# Non-classical selectivities in the reduction of alkenes by cobalt-mediated hydrogen atom transfer†
†Electronic supplementary information (ESI) available: Detailed experimental procedures and characterization data for all new compounds. See DOI: 10.1039/c5sc02476e


**DOI:** 10.1039/c5sc02476e

**Published:** 2015-08-21

**Authors:** Xiaoshen Ma, Seth B. Herzon

**Affiliations:** a Department of Chemistry , Yale University , New Haven , Connecticut 06520 , USA . Email: seth.herzon@yale.edu

## Abstract

Classical methods for alkene hydrogenation typically reduce less-substituted or more-strained alkenes, or those in proximity to a directing group, most rapidly. Here we describe a cobalt-mediated hydrogenation protocol that provides complementary selectivities in the reduction of several classes of olefins and alkynes. The selectivity of this reduction derives from a hydrogen atom transfer mechanism, which favors the generation of the more stable alkylradical intermediate. We also report the first alkene hydrobromination, hydroiodination, and hydroselenylation by a hydrogen atom transfer process.

## Abstract 2

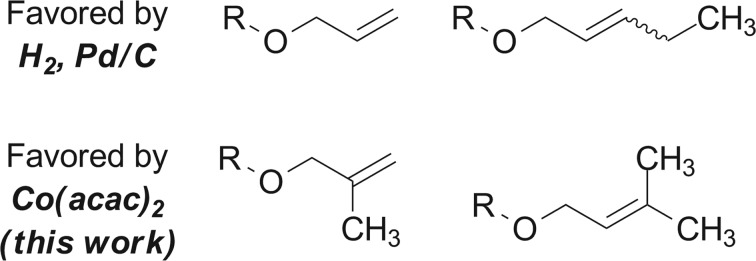
It is shown that the reduction of alkenes by hydrogen atom transfer provides selectivities that are distinct from classical hydrogenation catalysts. The first alkene hydrobromination, hydroiodination, and hydroselenylation reactions that proceed by hydrogen atom transfer processes are also reported.

## 

The application of transition metal hydrides as hydrogen atom donors to alkenes has been intensively studied.^1^,^2^ Early reports employed stoichiometric amounts of metal hydrides and activated alkenes.^3^–^13^ Recently a range of exceptionally useful alkene hydrofunctionalization reactions have been recorded by Mukaiyama, Carreira, Boger, Baran, and others using cobalt-, manganese-, and iron-based catalysts (Scheme 1a).^14^–^42^ Reports from our laboratory and Shenvi and co-workers detail methods for the reduction of alkenyl halides^43^,^44^ and unactivated alkenes^44^ by hydrogen atom transfer. Although prior examples of metal-catalyzed hydrogen atom transfer reduction had been described,^45^,^46^ these were the first to proceed with unactivated alkenes as substrates under mild conditions. In the reduction of alkenyl halides, the halogen substituent is thought to control selectivity by biasing the first hydrogen atom transfer toward the generation of a stabilized α-haloalkylradical intermediate (Scheme 1b). This mechanism avoids alkylmetal intermediates, which can lead to hydrodehalogenation products.^47^ Shenvi and co-workers subsequently reported a practical method for alkene isomerization and cycloisomerization by hydrogen atom transfer.^48^

**Scheme 1:**
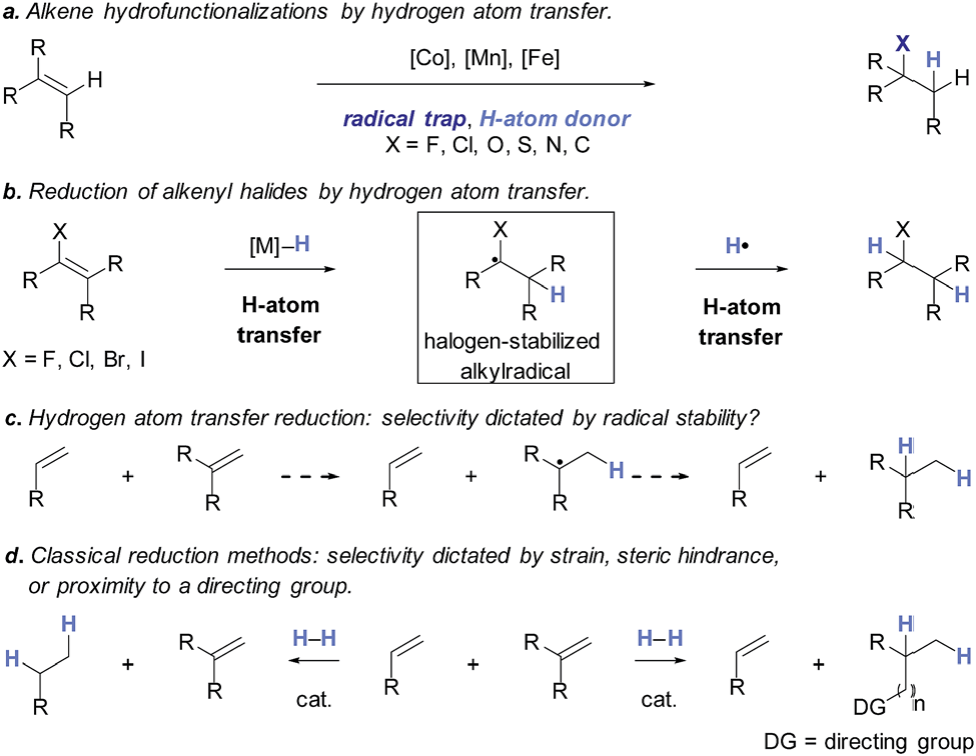
(a) Alkene hydrofunctionalization by hydrogen atom transfer; (b) the hydrogen atom transfer reduction of alkenyl halides to alkyl halides proceeds *via* selective addition to form a halogen-stabilized alkylradical intermediate; (c) proposed selectivity in hydrogen atom transfer reduction. (d) Classical trends in hydrogenation selectivity.

The rates of hydrogen atom transfer to alkenes depend upon the stability of the resulting alkylradical intermediate.^49^,^50^ These data and our alkenyl halide reduction led us to test whether the factors governing hydrogen atom transfer to alkenes could be exploited to obtain non-classical selectivities in alkene hydrogenation (Scheme 1c). Such selectivity would complement traditional approaches, which rely on the higher reactivity of strained and less-hindered alkenes^51^ or functional group coordination (Scheme 1d).^52^ However, for this approach to be successful, the steric encumbrance of the catalyst needs to be minimized to allow the radical-stabilizing effect to dominate.^53^

To facilitate analysis, 2-methylallyl 4-methoxybenzoate (**1a**) and an equimolar amount of allyl 4-methoxybenzyl ether (**1b**) were employed as substrates (Table 1). After some experimentation, we found that treatment of a solution of **1a** and **1b** in *n*-propanol with Co(acac)_2_ (1.0 equiv.), TBHP (2.0 equiv.), DHB (10 equiv.), and triethylsilane (10 equiv.) formed the products **2a** and **2b** in 71% and 14% yields after 135 min (5.1 : 1.0 ratio of **2a** : **2b**, entry 1). We attempted to improve the selectivity by decreasing the reaction temperature, but low conversion was observed (entry 2). Alternatively, when the amount of Co(acac)_2_ and TBHP were reduced to 50 mol%, the conversion of **1a** was 72%, but only 31% of **2a** was obtained, suggesting decomposition of the alkylradical (entry 3). The reactions in entries 1 and 2 were conducted under air in a flask sealed with a septum and pierced with a 16-gauge needle. Conducting the hydrogenation in an open flask enhanced the rate (30 *vs.* 135–180 min) but diminished selectivity (63% and 17% yield of **2a** and **2b**, respectively, entry 4). Interestingly, reducing the amount of Co(acac)_2_ and TBHP to 25 mol% decreased the conversion of **1b** but the major product was the alcohol **3** (69%, entry 5). The basis for the difference in product selectivity between entries 4 and 5 is not known, but the production of **3** is consistent with earlier reports describing the formal Markovnikov hydration of alkenes by Mukaiyama and co-workers.^15^,^19^ We posited that higher selectivity and yields could be achieved under an inert atmosphere, as the catalyst would be less activated and the alkylradical intermediate would be less likely to undergo decomposition. When the reaction was conducted under argon, useful selectivities were observed (4.9 : 1.0), but the conversion of **1a** was incomplete (81%, entry 6). Warming to 50 °C increased conversion with only a minor decrease in selectivity (4.4 : 1.0 ratio of **2a** : **2b**, entry 7). Slow addition of TBHP (syringe pump) provided a 91% yield of **2a** with 4.6 : 1.0 selectivity (entry 8). As the conditions of entry 1 provided the highest selectivity and those of entry 8 afforded the highest yield, both were employed in the investigation of the scope (referred to as conditions A and B, respectively). Other hydrogen atom donors were ineffective. Reduction using manganese tris(dipivaloylmethane)^44^ was non-selective (see Table S3†).

**Table 1:** Optimization of the reduction mediated by Co(acac)_2_^*a*^

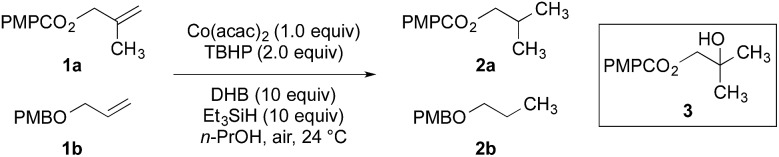							
Entry	Variation from above	Time	Conv. **1a**	Yield **2a**	Conv. **1b**	Yield **2b**	**2a** : **2b**
1	None	135 min	>95%	71%	14%	14%	5.1 : 1.0
2	0 °C	300 min	<5%	<1%	7%	<1%	—^*b*^
3	Co(acac)_2_, TBHP (50 mol% each)	180 min	72%	31%	18%	<1%	—^*b*^
4	Open flask	30 min	>95%	63%	56%	17%	3.7 : 1.0
5	Co(acac)_2_, TBHP (25 mol% each), open flask	180 min	75%	25% (69% of **3**)^*c*^	6%	<1%	—^*b*^
6	Argon	360 min	81%	69%	33%	14%	4.9 : 1.0
7	Argon, 50 °C	120 min	>95%	80%	25%	18%	4.4 : 1.0
8	TBHP (1.0 equiv., slow addition), argon, 40 °C	60 min	>95%	91%	28%	20%	4.6 : 1.0

^*a*^Reactions employed 250 μmol each of **1a** and **1b**. Conversions and yields were determined by ^1^H NMR spectroscopy using mesitylene or 1,3,5-trimethoxybenzene as an internal standard.

^*b*^The ratio of **2a** : **2b** could not be determined due to the absence of **2a** and/or **2b** in the ^1^H NMR spectrum of the unpurified product mixture.

^*c*^69% of **3** was isolated after purification by flash-column chromatography.

The experiments in Table 2 establish the relative reactivity of several alkene and alkene–alkyne pairs. For each substrate pair, the condition affording higher selectivities is shown (for complete data, see Table S2†). These data show that useful levels of selectivity can be obtained for eight pairs of unsaturated substrates. For example, entries 1 and 2 show that 2,2-disubstituted alkenes are reduced selectively over α-olefins, and that allylic substituents such as esters, bulky silyl ethers, or alkyl ethers do not significantly influence selectivity. The results in entries 3 and 4 show that bromo- and chloroalkenes are reduced more readily than α-olefins, which reflects the additional stabilization afforded by the halogen.^54^ It is noteworthy that reduction of the bromoalkene **1e** is complete within 20 min while ∼2 h are required to achieve conversion of the dialkyl-substituted alkene **1a**. The cyclic 2,2-disubstituted alkene **1f** was also reduced with comparable selectivity over the α-olefin **4a** (entry 5). In accord with these data and the mechanistic hypothesis shown in Scheme 1, 2, 2-disubstituted alkenes are reduced more readily than 1,2-disubstituted alkenes (entries 6 and 7). Heterogeneous hydrogenation catalysts typically reduce alkynes faster than alkenes,^51^ but this reduction method provides high levels of selectivity for 2,2-disubstituted alkenes over internal alkynes (entry 8). Trisubstituted alkenes are reduced with modest selectivity over α-olefins (entries 11 and 12), but are reduced with higher selectivities over *trans*- or *cis*-1,2-disubstituted alkenes (entries 13 and 14, respectively). Styrenyl and terminal arylalkynes undergo rapid decomposition to unidentified products (entries 15 and 16) and fluoroalkenes react slowly under these conditions (entry 17). To confirm that these conditions are effective in a polyfunctional setting, we evaluated the reduction of the diene **9** (Scheme 2). These conditions resulted in 72% reduction of the 2,2-disubstituted alkene and 9% reduction of the 1,2-disubstituted alkene (8.0 : 1.0 selectivity).

**Table 2:** Relative reactivity of different alkene or alkene–alkyne pairs toward reduction by Co(acac)_2_

Entry	Target substrate	Conditions and yield^*a*^	Competition substrate	Conversion^*b*^	Yield^*b*^	Ratio of reduction products
1	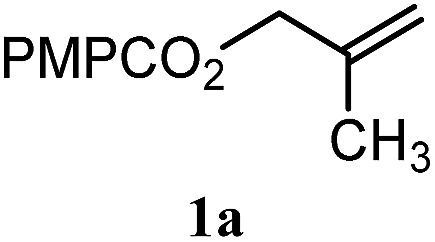	A: 79^*b*^%	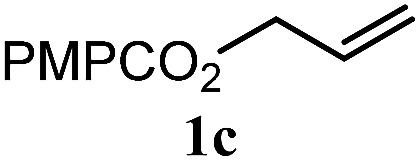	17%	14%	5.6 : 1.0
2	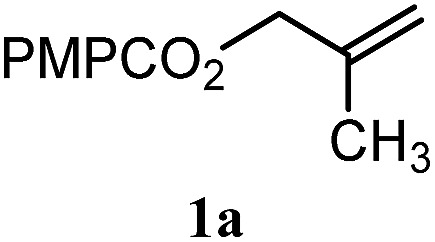	A: 86%	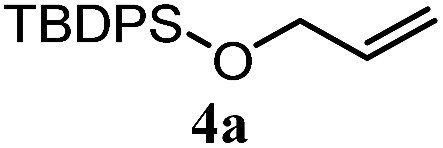	17%	12%	7.2 : 1.0
3	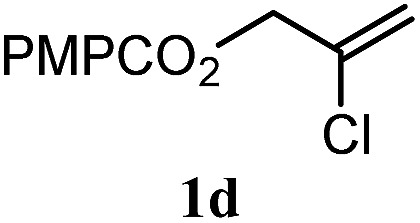	A: 79%	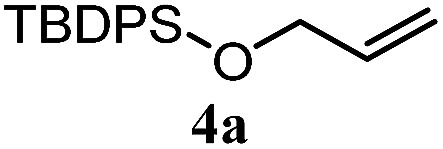	15%	15%	5.3 : 1.0
4	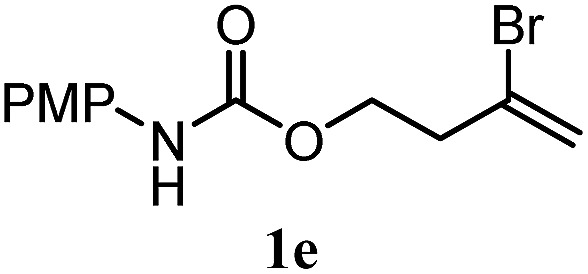	A: 71%	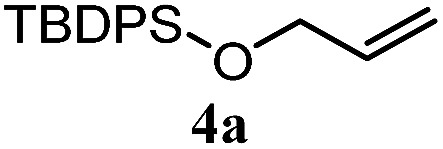	5%	—^*c*^	14 : 1.0^*d*^
5	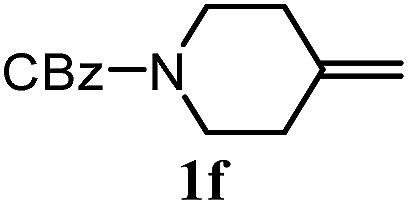	A: 78%	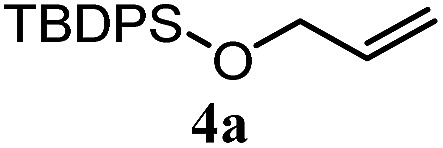	17%	17%	4.6 : 1.0
6	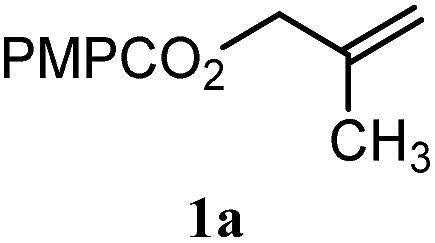	B: 96%	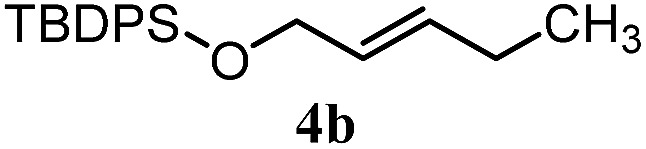	11%	11%	8.7 : 1.0
7	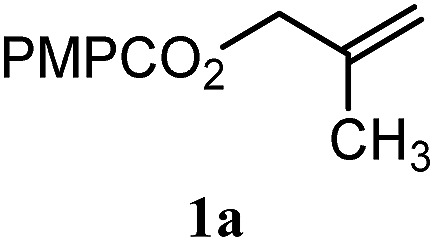	A: 70%	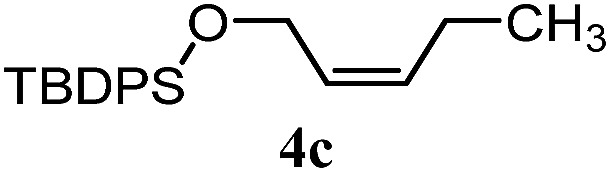	14%	8%	8.8 : 1.0
8	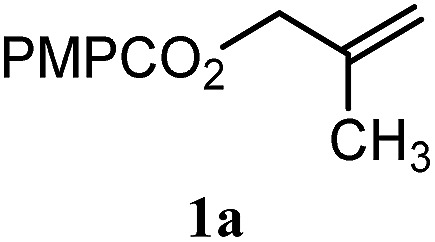	B: 93%	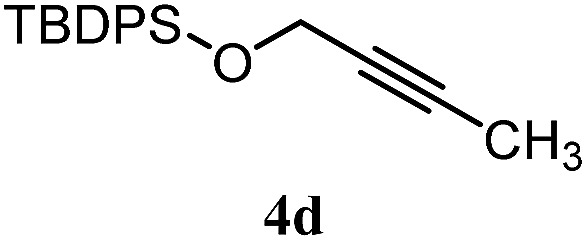	12%	—^*c*^	7.8 : 1.0^*d*^
9	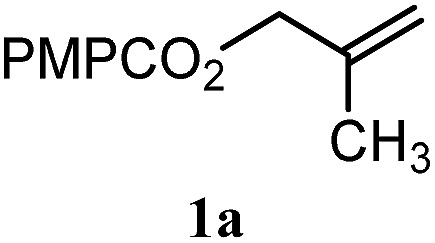	B: 89%	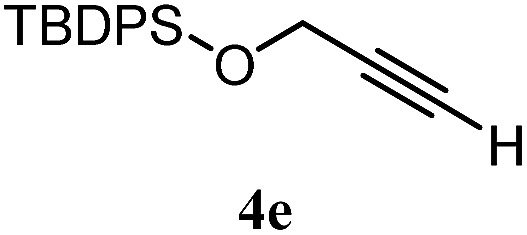	30%	—^*c*^	3.0 : 1.0^*d*^
10	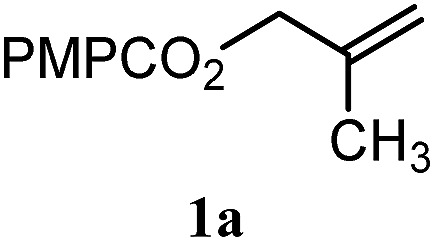	B: 90%	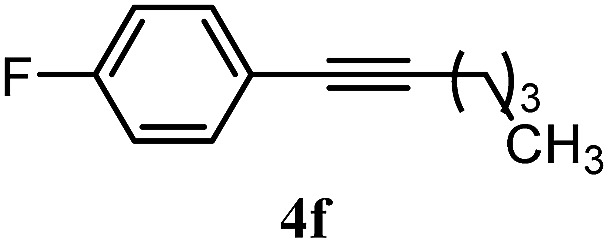	22^*e*^%	—^*c*^	4.1 : 1.0^*d*^
11	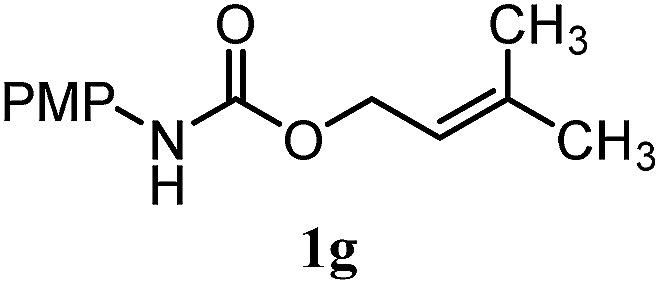	A: 92%	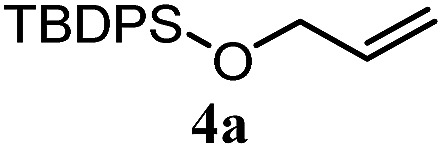	46%	46%	2.0 : 1.0
12	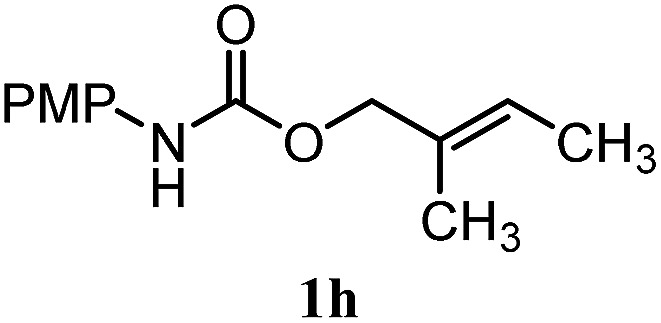	A: 95%	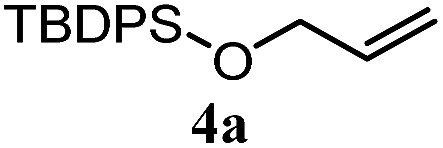	64%	64%	1.5 : 1.0
13	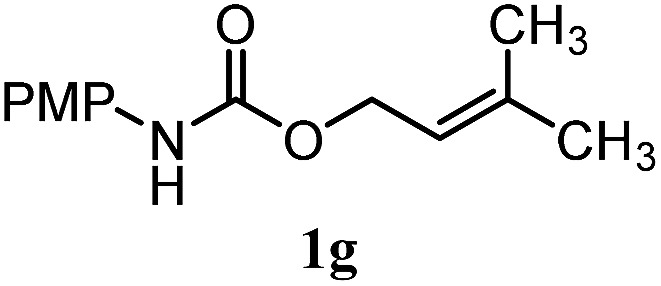	A: 86%	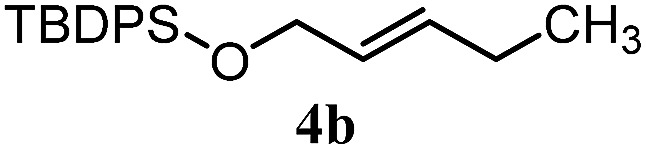	22%	18%	4.8 : 1.0
14	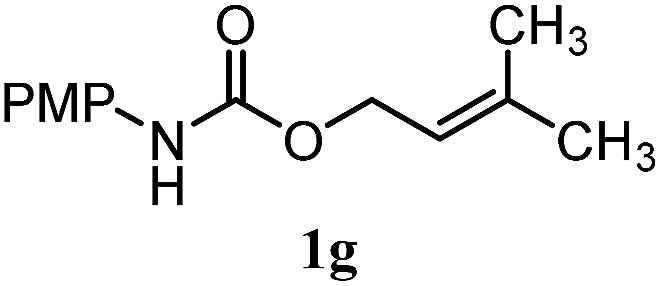	A: 90%	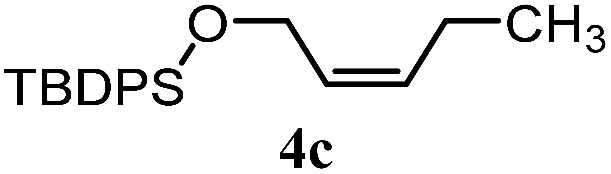	35%	28%	3.2 : 1.0
15	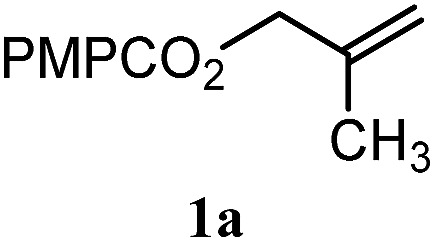	A: 95%	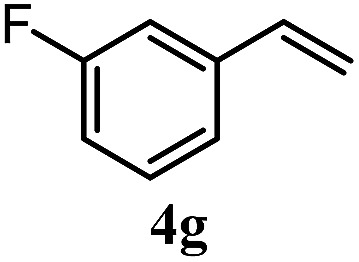	93^*e*^%	—^*f*^	1.0 : 1.0^*d*^
16	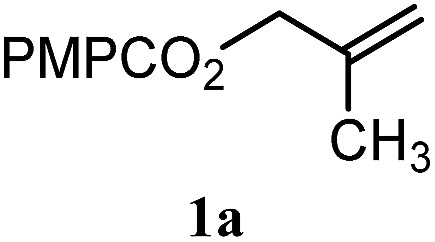	A: 55%	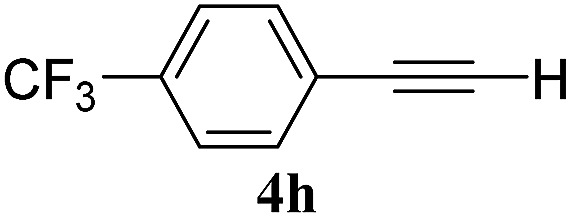	>95^*e*^%	—^*f*^	1.0 : 1.7^*d*^
17	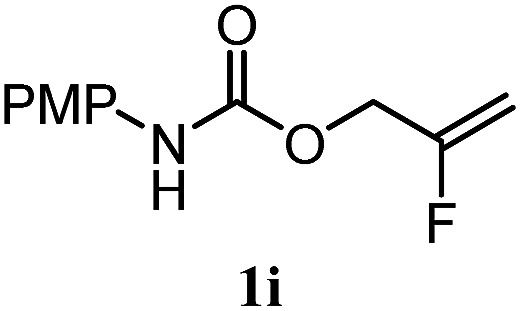	A: 62%	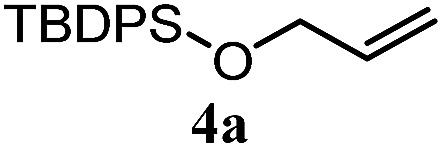	82%	83%	1.0 : 1.3

^*a*^Yields refer to purified products isolated by flash-column chromatography, unless otherwise noted. Condition A: Co(acac)_2_ (1 equiv.), TBHP (1–8 equiv.), 1,4-DHB (10 equiv.), Et_3_SiH (10 equiv.), *n*-PrOH (0.3 M), air, 24 °C. Condition B: Co(acac)_2_ (1 equiv.), TBHP (0.97–1.28 equiv., slow addition), 1,4-DHB (10 equiv.), Et_3_SiH (10 equiv.), *n*-PrOH (0.3 M), argon, 40 °C. The amount of TBHP varies among substrates, see the ESI.

^*b*^Determined by ^1^H NMR spectroscopy using mesitylene as an internal standard.

^*c*^Competition substrate was converted to unidentified products.

^*d*^Ratios are calculated as the yield of the target substrate *versus* the conversion of the competition substrate.

^*e*^Conversion determined by ^19^F NMR with hexafluorobenzene as an internal standard.

^*f*^Decomposition was observed.

**Scheme 2:**
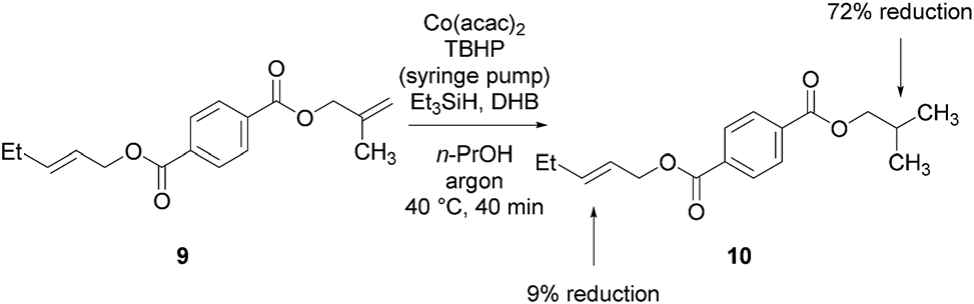
Hydrogenation of the diene **9**.

To benchmark these data, the relative reactivity of six classes of unsaturated substrates were examined under heterogeneous conditions (Table 3, for additional conditions, see Table S3†). As expected, the less-hindered alkene (or alkyne) was reduced preferentially. Thus, whereas classical hydrogenation conditions typically favor reaction of the most accessible (least-substituted) alkene, the hydrogen atom transfer reduction we have developed reverses this well-established trend.

**Table 3:** Reduction selectivities under classical and hydrogen atom transfer conditions^*a*^

	Co(acac)_2_	H_2_/Pd–C	
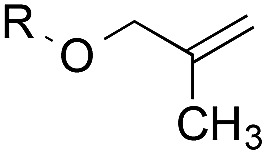	5.1 : 1.0	1.0 : 4.4	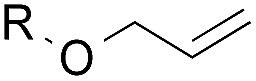
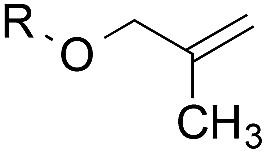	8.7 : 1.0	1.0 : 1.9	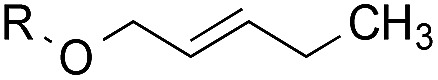
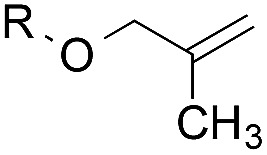	8.8 : 1.0	1.0 : 1.3	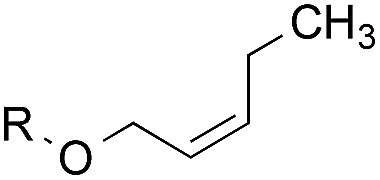
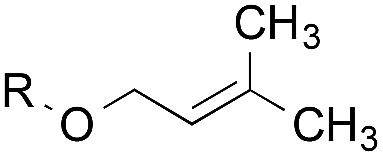	4.8 : 1.0	1.0 : 8.0	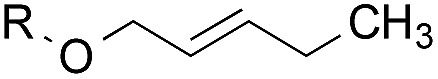
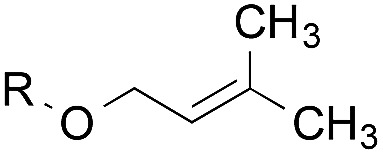	3.2 : 1.0	1.0 : 3.3	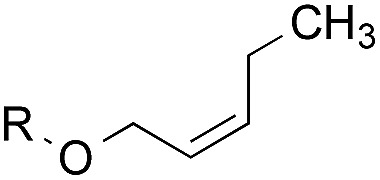
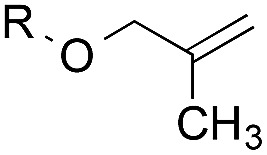	7.8 : 1.0	1.0 : 2.5	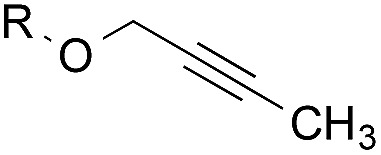

^*a*^For heterogeneous hydrogenation conditions, R = PMPCO_2_ (see the ESI).

Finally, we extended these studies toward the first hydrobromination, hydroiodination, and hydroselenation reactions that proceed by hydrogen atom transfer (Table 4). These experiments find important precedent in the work of Carreira and co-workers, who developed the first hydrochlorination of alkenes by hydrogen atom transfer.^32^ Here we evaluated a range of bromine, iodine, and selenium atom donors under our hydrogen atom transfer conditions (Table S4†). We found that addition of *p*-toluenesulfonyl bromide, diiodomethane, or *Se*-phenyl 4-methylbenzenesulfonoselenoate formed the desired hydrofunctionalization products. The hydrobromination and hydroselenation reactions provided high yields of products for α-, 2,2-, and trisubstituted olefins, but the hydroiodination of α- and trisubstituted alkenes did not proceed to completion. Application of the hydrobromination reaction to alkenyl halides formed the geminal dihalides **8a** and **8b** in high yield.

**Table 4:** Hydrobromination, hydroiodination, and hydroselenation of alkenes and alkenyl halides^*a*^

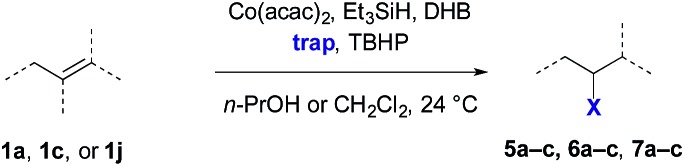			
Substrate	Products		
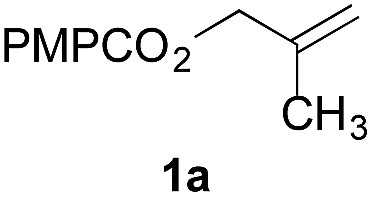	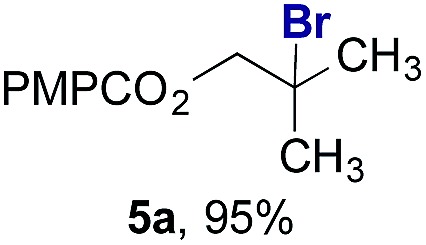	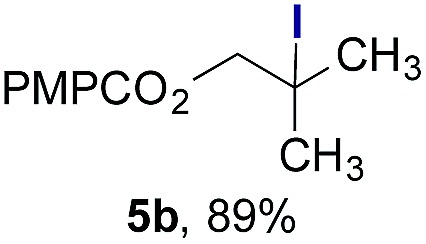	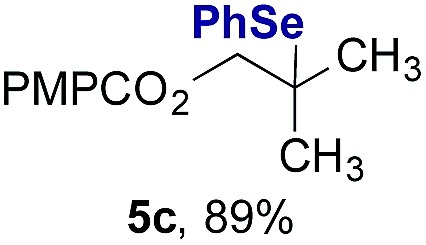
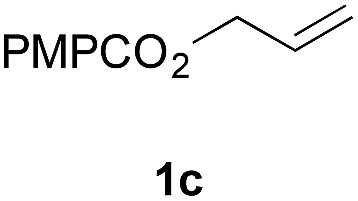	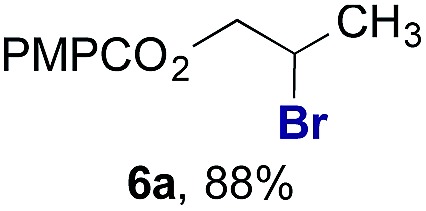	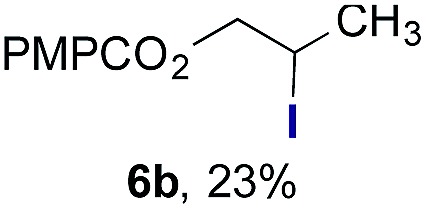	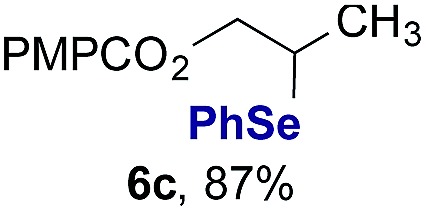
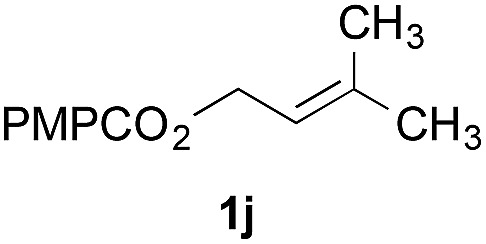	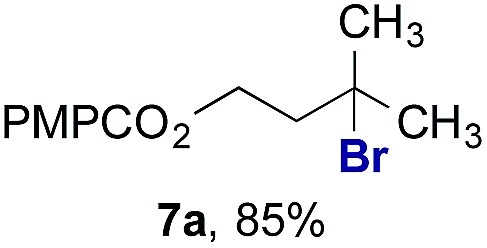	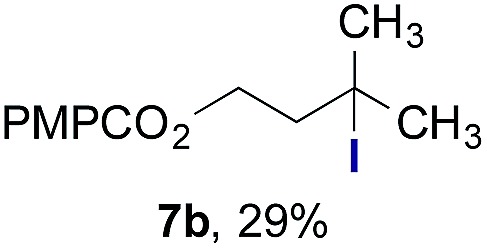	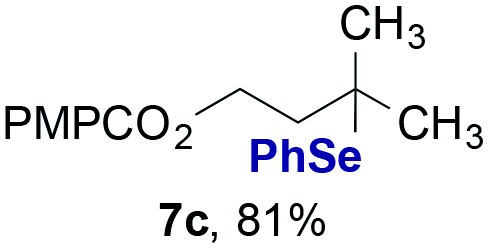
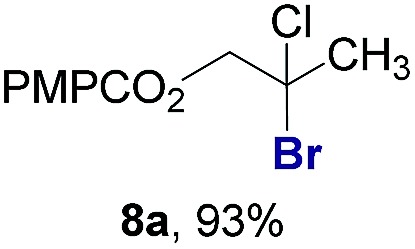		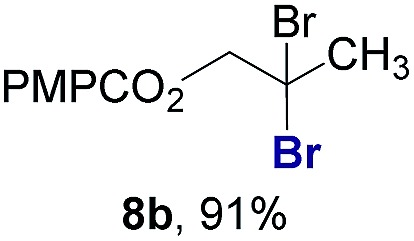	

^*a*^Yields refer to purified products isolated by flash-column chromatography. Hydrobromination: Co(acac)_2_ (1 equiv.), TBHP (1 equiv.), 1,4-DHB (3.75 equiv. for unfunctionalized alkenes, omitted for alkenyl halides), Et_3_SiH (10 equiv.), tosyl bromide (2.5 equiv.), *n*-PrOH (0.3 M) for unfunctionalized alkenes, DCM (0.3 M) for alkenyl halides, argon, 24 °C. Hydroiodination: Co(acac)_2_ (1 equiv.), TBHP (1 equiv.), 1,4-DHB (3.75 equiv.), Et_3_SiH (10 equiv.), diiodomethane (15 equiv.), DCM (0.3 M), argon, 24 °C. Hydroselenation: Co(acac)_2_ (1 equiv.), TBHP (1 equiv.), 1,4-DHB (3.75 equiv.), Et_3_SiH (10 equiv.), *Se*-phenyl 4-methylbenzenesulfonoselenoate (2.5 equiv.), *n*-PrOH (0.3 M), argon, 24 °C.

## Conclusions

In summary, we have shown that hydrogen atom transfer reduction provides selectivities that complement classical methods in the reduction of several alkene and alkene–alkyne pairs. In addition, we have described the first hydrobromination, hydroiodination, and hydroselenation of alkenes that proceed by hydrogen atom transfer. We believe that these methods constitute useful additions to the burgeoning area of practical hydrogen atom transfer reactions.

## Supplementary Material

Supplementary information
