# Supplementary material for: Non-classical selectivities in the reduction of alkenes by cobalt-mediated hydrogen atom transfer
Source: Chem Sci. 2015 Aug 21;6(11):6250–5. doi: 10.1039/c5sc02476e (PMC6054054; doi:10.1039/c5sc02476e)
Supplement: Supplementary file 1 [file SC-006-C5SC02476E-s001.pdf]

Electronic Supplementary Material (ESI) for Chemical Science.
This journal is © The Royal Society of Chemistry 2015

Non-Classical Selectivities in the Reduction of Alkenes by Cobalt-Mediated Hydrogen Atom Transfer

Xiaoshen Ma and Seth B. Herzon*

Department of Chemistry, Yale University, 225 Prospect Street, New Haven, CT 06520

Chem. Sci.

Supporting Information

Index

Table S1......S2
Table S2......S3
Table S3......S4
Table S4......S5
General Experimental Procedures......S6
Materials......S6
Instrumentation......S6
Synthetic Procedures......S8
Catalog of Nuclear Magnetic Resonance and Infrared Spectra of Purified Products......S79
^{1}H NMR Spectra of Unpurified Product Mixtures Containing Added Internal Standard......S160
Bibliography......S213

Table S1. The influence of hydrogen atom donor to the reaction.[a]

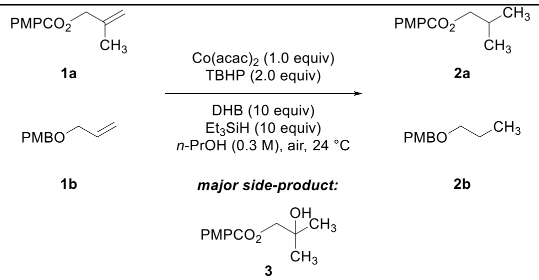

entry	variation from the conditions above	time	conv. **1a**	yield **2a**	conv. **1b**	yield **2b**	**2a**:**2b**
1	cyclohexa-2,5-diene-1-carboxylic acid (10 equiv) instead of DHB	280 min	>95%	13% (60% **3**)[c]	32%	3%	4.3:1.0
2	methyl cyclohexa-2,5-diene-1-carboxylate (10 equiv) instead of DHB	40 min	33%	15% (18% **3**)[c]	11%	4%	3.8:1.0
3	1-methoxycyclohexa-1,4-diene (10 equiv) instead of DHB	380 min	5%	5%	8%	3%	1.7:1.0

[a]Reactions employed 250 µmol each of **1a** and **1b**. All conversions and yields were calculated by ^{1}H NMR spectroscopy using mesitylene as an internal standard.

Table S2. Relative reactivity of different unsaturated substrates.

entry	target substrate	conditions and yield[a]	competition substrate	conversion[b]	yield[b]	ratio of reduction products
1	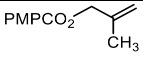 **1a**	A: 79%[b]	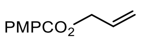 **1c**	17%	14%	5.6:1.0
		B: 91%[b]		29%	24%	3.8:1.0
2	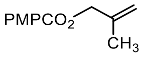 **1a**	A: 86%	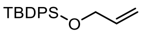 **4a**	17%	12%	7.2:1.0
		B: 92%		17%	17%	5.4:1.0
3	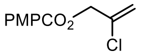 **1d**	A: 79%	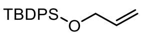 **4a**	15%	15%	5.3:1.0
		B: 87%		29%	28%	3.1:1.0
4	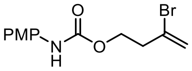 **1e**	A: 71%	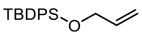 **4a**	5%	—[c]	14:1.0[d]
		B: 83%		38%	30%	2.8:1.0
5	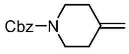 **1f**	A: 78%	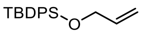 **4a**	17%	17%	4.6:1.0
		B: 88%		38%	39%	2.3:1.0
6	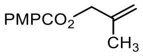 **1a**	A: 69%	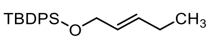 **4b**	12%	9%	7.7:1.0
		B: 96%		11%	11%	8.7:1.0
7	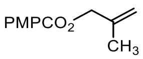 **1a**	A: 70%	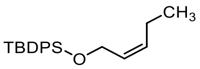 **4c**	14%	8%	8.8:1.0
		B: 92%		12%	11%	8.4:1.0
8	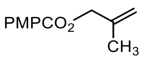 **1a**	A: 72%	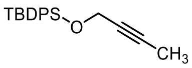 **4d**	12%	—[c]	6.0:1.0[d]
		B: 93%		12%	—[c]	7.8:1.0[d]
9	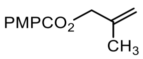 **1a**	A: 77%	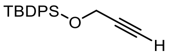 **4e**	26%	—[c]	3.0:1.0[d]
		B: 89%		30%	—[c]	3.0:1.0[d]
10	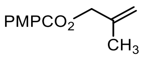 **1a**	A: 64%	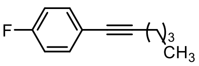 **4f**	31%[e]	—[c]	2.1:1.0[d]
		B: 90%		22%[e]	—[c]	4.1:1.0[d]
11	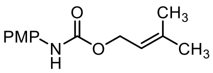 **1g**	A: 92%	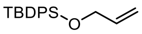 **4a**	46%	46%	2.0:1.0
		B: >99%		84%	82%	1.2:1.0
12	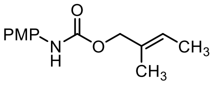 **1h**	A: 95%	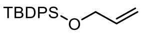 **4a**	64%	64%	1.5:1.0
13	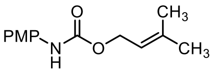 **1g**	A: 86%	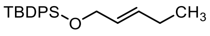 **4b**	22%	18%	4.8:1.0
		B: 92%		25%	25%	3.7:1.0
14	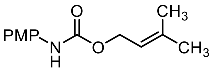 **1g**	A: 90%	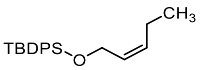 **4c**	35%	28%	3.2:1.0
		B: 89%		31%	29%	3.1:1.0
15	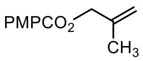 **1a**	A: 95%	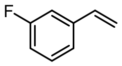 **4g**	93%[e]	—[f]	1.0:1.0[d]
		B: 96%		> 95%[e]	—[f]	1.0:1.0[d]
16	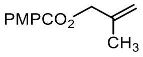 **1a**	A: 55%	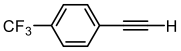 **4h**	> 95%[e]	—[f]	1.0:1.7[d]
17	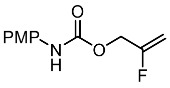 **1i**	A: 62%	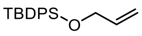 **4a**	82%	83%	1.0:1.3

[a]Reactions employed 250 μmol of each substrate. Yields refer to purified products isolated by flash-column chromatography, unless otherwise noted. [b]Determined by ^{1}H NMR spectroscopy using mesytilene as an internal standard. [c]Competition substrate was converted to unidentified products. [d]Ratios are calculated as the yield of the target substrate versus the conversion of the competition substrate. [e]Conversion determined by ^{19}F-NMR with hexafluorobenzene as an internal standard. [f]Decomposition was observed.

Table S3. Evaluation of heterogeneous, directed homogeneous, and hydrogen atom transfer reduction of **1a** and **1c**.[a]

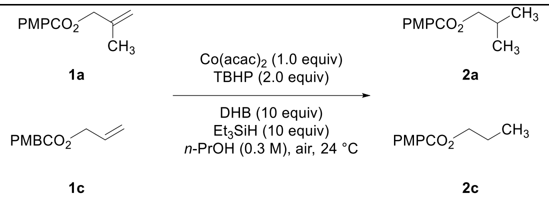

entry	conditions	time	conv. **1a**	yield **2a**	conv. **1c**	yield **2c**	**2a**:**2b**
1	1 mol% Pd/C, 1 atm H_2, CH_3OH, 24 ℃	5 min	13%	13%	70%	57%	1.0:4.4
2	0.5 mol% Pt/C, 1 atm H_2, CH_3OH, 24 ℃	3 min	24%	13%	56%	25%	1.0:1.9
3	2 mol% [Ir(COD)(PCy_3)py], 1 atm H_2, CH_2Cl_2, 24 ℃	180 min	8%	5%	43%	28%	1.0:5.6
4	Mn(dpm)$_3$, TBHP, $PhSiH_3$, *i*-PrOH, 24 ℃	10 min	59%	24%	53%	25%	1.0:1.0

[a]Reactions employed 250 µmol each of **1a** and **1b**. All conversions and yields were calculated by ^{1}H NMR spectroscopy using mesitylene as an internal standard.

Table S4. Optimization of hydrobromination, hydroiodination, and hydroselenation reactions.[a]

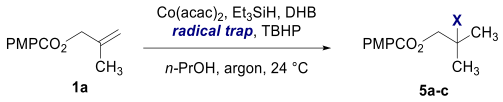

entry	radical trap	variation from conditions above	yield[b]	yield of reduction[c]
1	TsBr	DHB omitted	<1%[c]	<1%
2	TsBr	—	95%	<1%
3	I_2	DCM as solvent	<1%	<1%
4	TsI	DCM as solvent	<1%[c]	<1%
5	NIS	DCM as solvent	<1%[c]	<1%
6	$(CH_2I)_2$	DCM as solvent	<1%[c]	<1%
7	CH_2I_2	DCM as solvent, 15 equiv of CH_2I_2	89%	<1%
8	CH_2I_2	DCM as solvent, 7.5 equiv of CH_2I_2	52%[d]	<1%
9	ICH_2CO_2Et	DCM as solvent	49%[c]	<1%
10	ICH_2CN	DCM as solvent	36%[c]	<1%
11	TsSePh	—	89%	<1%

[a]Conditions: $Co(acac)_2$ (100 mol%), TBHP (100 mol%), DHB (3.75 equiv), Et_3SiH (10 equiv), radical trap (2.5 equiv), *n*-PrOH (0.3 M), argon, 24 °C. [b]Isolated yield after purification by flash-column chromatography. [c]Determined by ^1H NMR analysis against an internal standard. [d]The silyl ether (X = OTES) was isolated in 47% yield.

General Experimental Procedures. All reactions were performed in single-neck, flame-dried, round-bottomed flasks fitted with rubber septa under a positive pressure of argon, unless otherwise noted. Air- and moisture-sensitive liquids were transferred via syringe or stainless steel cannula, or were handled in a nitrogen-filled drybox (working oxygen level <5 ppm). Organic solutions were concentrated by rotary evaporation at 30–33 °C. Intermediates were purified using a Biotage Isolera system, employing polypropylene cartridges preloaded with silica gel (60 Å, 40–63 µm particle size, purchased from Silicycle, Quebec City, Canada). Alternatively, intermediates were purified using a Teledyne ISCO system, employing RediSep Rf High Performance Gold cartridges (RediSep Rf Gold Silica, 20–40 um spherical, purchased from Teledyne ISCO, Dallas, Texas). Samples were eluted using a flow rate of 12–50 mL/min, with detection by UV (254 nm). Analytical thin-layered chromatography (TLC) was performed using glass plates pre-coated with silica gel (0.25 mm, 60 Å pore size) impregnated with a fluorescent indicator (254 nm). TLC plates were visualized by exposure to ultraviolet light (UV) and/or submersion in aqueous ceric ammonium molybdate solution (CAM), or aqueous potassium permanganate solution ($KMnO_4$), followed by brief heating on a hot plate (120 °C, 10–15 s).

Materials. Commercial solvents and reagents were used as received with the following exceptions. Acetonitrile was purified according to the method of Pangborn et al.[1] Pyridine was distilled from calcium hydride under an atmosphere of nitrogen immediately before use. Commercial anhydrous *N*,*N*-dimethylformamide (Sigma-Aldrich Corporation, St. Louis, MO) was degassed by three freeze–pump–thaw cycles and stored over activated 4Å MS under an atmosphere of nitrogen before use. 1,4-Dioxane was degassed by three freeze-pump-thaw cycles, and was stored under an atmosphere of nitrogen before use. Tetrahydrofuran was distilled from sodium–benzophenone under an atmosphere of nitrogen immediately before use. Triethylamine was distilled from calcium hydride under an atmosphere of nitrogen immediately before use. *n*-Propanol was dried over calcium hydride for 12 h at 24 °C, degassed by three freeze–pump–thaw cycles, vacuum transferred, and stored under an atmosphere of argon before use. Triethylsilane was degassed by three freeze–pump–thaw cycles and stored under an atmosphere of argon before use. 1,4-Dihydrobenzene was degassed by three freeze–pump–thaw cycles, vacuum transferred, and stored under an atmosphere of argon at –10 °C before use. Cobalt bis(acetylacetonate) was dried by heating overnight in vacuo (70 °C, 200 mTorr), and stored under an atmosphere of argon before use. Benzyl 4-oxopiperidine-1-carboxylate,[2] (*E*)-2-methylbut-2-en-1-ol,[3] cyclohexa-2,5-diene-1-carboxylic acid,[4] methyl cyclohexa-2,5-diene-1-carboxylate,[4] manganese tris(dipivaloylmethane),[5] tosyl bromide,[6] tosyl iodide,[7] *Se*-phenyl 4-methylbenzenesulfonoselenoate,[8] *N*-(benzyloxy)-1-(phenylsulfonyl)methanimidoyl cyanide[9], and (phenylsulfonyl)methanal *O*-benzyl oxime[9] were prepared according to published procedures. *p*-Toluenesulfonyl chloride was recrystallized from chloroform–pentane immediately before use. *N*-Iodosuccinimide was recrystallized from 1,4-dioxane–tetrachloromethane immediately before use. 1,2-Diiodoethane was recrystallized from diethyl ether–pentane immediately before use. The concentration of *n*-butyllithium in hexanes was determined by titration against a standard solution of diphenylacetic acid (average of three determinations).[10]

Instrumentation. Proton nuclear magnetic resonance spectra (^1H NMR) were recorded at 400, 500, or 600 MHz at 24 °C, unless otherwise noted. Chemical shifts are expressed in parts per million (ppm, δ scale) downfield from tetramethylsilane and are referenced to residual protium in the NMR solvent ($CHCl_3$, δ 7.26; $CHDCl_2$, δ 5.32; C_6HD_5, δ 7.16). Data are represented as follows: chemical shift, multiplicity (s = singlet, d = doublet, t = triplet, q = quartet, m = multiplet and/or multiple resonances, br = broad), integration, coupling constant in Hertz, and assignment. Proton-decoupled carbon nuclear magnetic resonance spectra (^{13}C NMR) were recorded at 100, 125, or 150 MHz at 24 °C, unless otherwise noted. Chemical shifts are expressed in parts per million (ppm, δ scale) downfield from tetramethylsilane and are referenced to the carbon resonances of the solvent ($CDCl_3$, δ 77.0; CD_2Cl_2, δ 54.0; C_6D_6, δ 128.1). Distortionless enhancement by polarization transfer spectra [DEPT (135)] were recorded at 100, 125, or 150 MHz at 24 °C, unless otherwise noted. ^{13}C NMR and DEPT (135) data are combined and represented as follows: chemical shift, carbon type [obtained from DEPT (135) experiments]. Proton-decoupled

fluorine nuclear magnetic resonance spectra (^{19}F NMR) were recorded at 375 MHz or 470 MHz at 24 °C, unless otherwise noted. Chemical shifts are expressed in parts per million (ppm, δ scale) downfield from fluorotrichloromethane. Attenuated total reflectance Fourier transform infrared spectra (ATR-FTIR) were obtained using a Thermo Electron Corporation Nicolet 6700 FTIR spectrometer referenced to a polystyrene standard. Data are represented as follows: frequency of absorption (cm^{-1}), intensity of absorption (s = strong, m = medium, w = weak, br = broad). High-resolution mass spectrometry (HRMS) were obtained on a Waters UPLC/HRMS instrument equipped with a dual API/ESI high-resolution mass spectrometry detector and photodiode array detector. Unless otherwise noted, samples were eluted over a reverse-phase C18 column (1.7 µm particle size, 2.1 × 50 mm) with a linear gradient of 5% acetonitrile–water containing 0.1% formic acid→95% acetonitrile–water containing 0.1% formic acid over 1.6 min, followed by 100% acetonitrile containing 0.1% formic acid for 1 min, at a flow rate of 600 µL/min.

Synthetic Procedures.

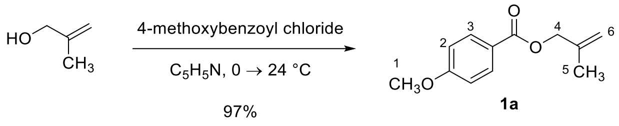

*Preparation of 2-methylallyl 4-methoxybenzoate (**1a**):*

4-Methoxybenzoyl chloride (1.50 g, 8.80 mmol, 1.10 equiv) was added dropwise via syringe to a solution of 2-methyl-2-propen-1-ol (576 mg, 8.00 mmol, 1 equiv) in pyridine (32 mL) at 0 °C. The reaction mixture was stirred for 30 min at 0 °C, and then the ice bath was removed. The reaction mixture was stirred for 24 h at 24 °C. The product mixture was transferred to a separatory funnel that had been charged with ethyl acetate (50 mL). The diluted product mixture was washed with saturated aqueous sodium bicarbonate solution (50 mL). The aqueous layer was isolated and the isolated aqueous layer was extracted with ethyl acetate (3 × 50 mL). The organic layers were combined and the combined organic layers were dried over sodium sulfate. The dried solution was filtered and the filtrate was concentrated. The residue obtained was purified by automated flash-column chromatography (eluting with 5% ethyl acetate–hexanes initially, grading to 10% ethyl acetate–hexanes, linear gradient) to afford 2-methylallyl 4-methoxybenzoate (**1a**) as a clear oil (1.60 g, 97%).

R_f = 0.55 (20% ethyl acetate–hexanes; UV, $KMnO_4$). ^1H NMR (600 MHz, $CDCl_3$) δ 8.02 (d, 2H, J = 8.4 Hz, H_3), 6.92 (d, 2H, J = 8.4 Hz, H_2), 5.05 (s, 1H, H_6), 4.96 (s, 1H, H_6), 4.71 (s, 2H, H_4), 3.87 (s, 3H, H_1), 1.82 (s, 3H, H_5). ^{13}C NMR (150 MHz, $CDCl_3$) δ 166.0 (C), 163.4 (C), 140.2 (C), 131.6 (CH), 122.6 (C), 113.6 (CH), 112.7 (CH_2), 67.8 (CH_2), 55.4 (CH_3), 19.6 (CH_3).

^1H and ^{13}C NMR data for 2-methylallyl 4-methoxybenzoate (**1a**) prepared in this way were in agreement with 46those previously described.[11]

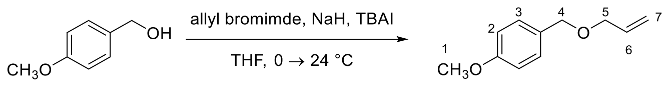

*Preparation of 1-((allyloxy)methyl)-4-methoxybenzene (**1b**):*

A 250-mL round-bottomed flask that had been fused to a Teflon-coated valve was charged with sodium hydride (300 mg, 7.50 mmol, 1.50 equiv). The reaction vessel was evacuated and refilled using a balloon of argon. This process was repeated twice. Tetrahydrofuran (24 mL) was added to the reaction vessel via syringe and the resulting mixture was cooled to 0 °C. A 250-mL round-bottomed flask was charged with 4-methoxybenzyl alcohol (691 mg, 5.00 mmol, 1 equiv). The vessel containing the starting material was evacuated and refilled using a balloon of argon. This process was repeated twice. Tetrahydrofuran (100 mL) was added to the vessel containing the starting material and the resulting solution was transferred via cannula to the sodium hydride suspension. The reaction mixture was stirred at 0 °C for 45 min. Tetrabutylammonium iodide (92.3 mg, 250 µmol, 0.0500 equiv) and allyl bromide (786 mg, 6.50 mmol, 1.30 equiv) were then added in sequence. The reaction mixture was allowed to warm over 30 min to 24 °C. The warmed reaction mixture was stirred for 12 h at 24 °C. The product mixture was filtered through a pad of silica gel and the pad was rinsed with ethyl acetate (50 mL). The filtrates were collected and combined and the combined filtrates were concentrated. The residue obtained was purified by automated flash-column chromatography (eluting with 5% ether–hexanes initially, grading to 10% ether–hexanes, linear gradient) to afford 1-((allyloxy)methyl)-4-methoxybenzene (**1b**) as a light yellow oil (890 mg, 99%).

R_f = 0.39 (20% ether–hexanes; UV, $KMnO_4$). ^{1}H NMR (600 MHz, $CDCl_3$) δ 7.27 (d, 2H, J = 7.8 Hz, H_3), 6.88 (d, 2H, J = 7.2 Hz, H_2), 5.94 (ddt, J = 16.8, 10.2, 5.4 Hz, 1H, H_6), 5.29 (d, J = 16.8 Hz, 1H, H_7), 5.19 (d, J = 10.2 Hz, 1H, H_7), 4.45 (s, 2H, H_4), 4.00 (d, J = 5.4 Hz, 2H, H_5), 3.80 (s, 3H, H_1). ^{13}C NMR (150 MHz, $CDCl_3$) δ 159.2 (C), 134.8 (CH), 130.3 (C), 129.3 (CH), 117.0 (CH_2), 113.7 (CH), 71.8 (CH_2), 70.8 (CH_2), 55.2 (CH_3).

^{1}H and ^{13}C NMR data for 1-((allyloxy)methyl)-4-methoxybenzene (**1b**) prepared in this way were in agreement with those previously described.[12]

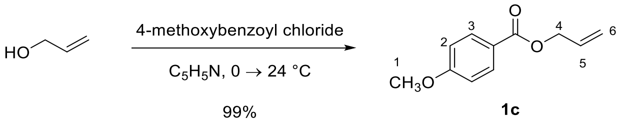

1c

*Preparation of allyl 4-methoxybenzoate (**1c**):*

4-Methoxybenzoyl chloride (1.50 g, 8.80 mmol, 1.10 equiv) was added dropwise via syringe to a solution of allyl alcohol (464 mg, 8.00 mmol, 1 equiv) in pyridine (32 mL) at 0 °C. The reaction mixture was stirred for 30 min at 0 °C, and then the ice bath was removed. The reaction mixture was stirred for 24 h at 24 °C. The product mixture was transferred to a separatory funnel that had been charged with ethyl acetate (50 mL). The diluted product mixture was washed with saturated aqueous sodium bicarbonate solution (50 mL). The aqueous layer was isolated and the isolated aqueous layer was extracted with ethyl acetate (3 × 50 mL). The organic layers were combined and the combined organic layers were dried over sodium sulfate. The dried solution was filtered and the filtrate was concentrated. The residue obtained was purified by automated flash-column chromatography (eluting with 5% ethyl acetate–hexanes initially, grading to 10% ethyl acetate–hexanes, linear gradient) to afford allyl 4-methoxybenzoate (**1c**) as a clear oil (1.54 g, 99%).

R_f = 0.55 (20% ethyl acetate–hexanes; UV, $KMnO_4$). ^{1}H NMR (600 MHz, $CDCl_3$) δ 8.02 (d, 2H, J = 8.4 Hz, H_3), 6.92 (d, 2H, J = 8.4 Hz, H_2), 6.03 (ddt, J = 16.8, 10.2, 4.2 Hz, 1H, H_5), 5.40 (d, J = 16.8 Hz, 1H, H_6), 5.27 (d, J = 10.2 Hz, 1H, H_6), 4.80 (d, J =4.2 Hz, 2H, H_4), 3.86 (s, 3H, H_1). ^{13}C NMR (150 MHz, $CDCl_3$) δ 166.0 (C), 163.4 (C), 132.5 (CH), 131.6 (CH), 122.5 (C), 117.9 (CH_2), 113.6 (CH), 65.2 (CH_2), 55.4 (CH_3).

^{1}H and ^{13}C NMR data for 2-methylallyl allyl 4-methoxybenzoate (**1c**) prepared in this way were in agreement with those previously described.[13]

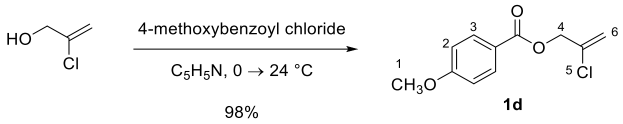

*Preparation of 2-chloroallyl 4-methoxybenzoate (**1d**):*

4-Methoxybenzoyl chloride (1.02 g, 5.96 mmol, 1.10 equiv) was added dropwise via syringe to a solution of 2-chloro-2-propen-1-ol (500 mg, 5.41 mmol, 1 equiv) in pyridine (22 mL) at 0 °C. The reaction mixture was stirred for 30 min at 0 °C, and then the ice bath was removed. The reaction mixture was stirred for 24 h at 24 °C. The product mixture was transferred to a separatory funnel that had been charged with ethyl acetate (20 mL). The diluted product mixture was washed with saturated aqueous sodium bicarbonate solution (20 mL). The aqueous layer was isolated and the isolated aqueous layer was extracted with ethyl acetate (3 × 20 mL). The organic layers were combined and the combined organic layers were dried over sodium sulfate. The dried solution was filtered and the filtrate was concentrated. The residue obtained was purified by automated flash-column chromatography (eluting with 5% ethyl acetate–hexanes initially, grading to 10% ethyl acetate–hexanes, linear gradient) to afford 2-chloroallyl 4-methoxybenzoate (**1d**) as a clear oil (1.20 g, 98%).

R_f = 0.52 (10% ethyl acetate–hexanes; UV, $KMnO_4$). ^1H NMR (500 MHz, $CDCl_3$) δ 8.04 (d, 2H, J = 9.0 Hz, H_3), 6.94 (d, 2H, J = 9.0 Hz, H_2), 5.55–5.53 (m, 1H, H_6), 5.45–5.42 (m, 1H, H_6), 4.87 (br s, 2H, H_4), 3.87 (s, 3H, H_1). ^{13}C NMR (150 MHz, $CDCl_3$) δ 165.0 (C), 163.4 (C), 135.9 (C), 131.6 (CH), 121.5 (C), 114.4 (CH_2), 113.5 (CH), 65.8 (CH_2), 55.2 (CH_3).

^1H and ^{13}C NMR data for 2-chloroallyl 4-methoxybenzoate (**1d**) prepared in this way were in agreement with those previously described.[14]

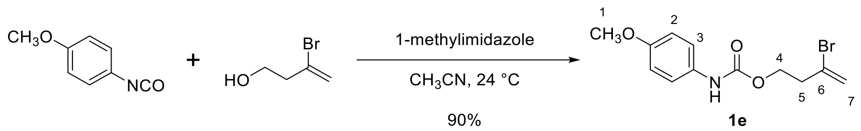

*Preparation of 3-bromobut-3-en-1-yl (4-methoxyphenyl)carbamate (**1e**):*

1-Methylimidazole (71.4 µL, 900 µmol, 0.300 equiv) and 4-methoxyphenyl isocyanate (386 µL, 3.00 mmol, 1.00 equiv) were added in sequence to a solution of 3-bromo-3-buten-1-ol (298 µL, 3.00 mmol, 1 equiv) in acetonitrile (7.5 mL) at 24 °C. The reaction mixture was stirred for 12 h at 24 °C. The product mixture was concentrated and the residue obtained was purified by automated flash-column chromatography (eluting with 10% ethyl acetate–hexanes initially, grading to 33% ethyl acetate–hexanes, linear gradient) to afford 3-bromobut-3-en-1-yl (4-methoxyphenyl)carbamate (**1e**) as a white solid (809 mg, 90%).

R_f = 0.34 (20% ethyl acetate–hexanes; UV, $KMnO_4$). ^{1}H NMR (600 MHz, $CDCl_3$) δ 7.34–7.26 (m, 2H, H_3), 6.85 (d, 2H, J = 9.0 Hz, H_2), 6.57 (br s, 1H, NH), 5.69 (br s, 1H, H_7), 5.52 (s, 1H, H_7), 4.34 (t, 2H, J = 6.3 Hz, H_4), 3.78 (s, 3H, H_1), 2.79 (t, 2H, J = 6.3 Hz, H_5). ^{13}C NMR (150 MHz, $CDCl_3$) δ 155.9 (C), 153.5 (C), 130.7 (C), 129.5 (C), 120.5 (CH), 119.1 (CH_2), 114.2 (CH), 62.3 (CH_2), 55.5 (CH_3), 40.8 (CH_2).

^{1}H and ^{13}C NMR data for 3-bromobut-3-en-1-yl (4-methoxyphenyl)carbamate (**1e**) prepared in this way were in agreement with those previously described.[14]

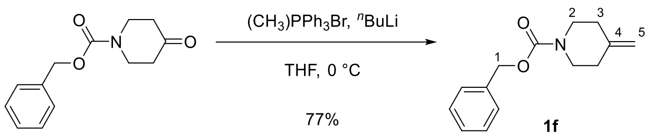

*Preparation of benzyl 4-methylenepiperidine-1-carboxylate (**1f**):*

A 250-mL round-bottomed flask that had been fused to a Teflon-coated valve was charged with methyltriphenylphosphonium bromide (4.29 g, 12.0 mmol, 1.20 equiv). The reaction vessel was evacuated and refilled using a balloon of argon. This process was repeated twice. Tetrahydrofuran (60 mL) was added to the reaction vessel via syringe and the resulting mixture was cooled to 0 °C. A solution of *n*-butyllithium in hexanes (2.42 M, 4.96 mL, 12.0 mmol, 1.20 equiv) was added dropwise via syringe to the cold mixture. The reaction mixture was stirred for 1 h at 0 °C. A 25-mL round-bottomed flask was charged with benzyl 4-oxopiperidine-1-carboxylate (2.33 g, 10.0 mmol, 1 equiv). The vessel containing the starting material was evacuated and refilled using a balloon of argon. This process was repeated twice. Tetrahydrofuran (10 mL) was added to the vessel containing the starting material and the resulting solution was transferred via cannula to the ylide solution. The reaction mixture was stirred at 0 °C for 1 h and then was allowed to warm over 30 min to 24 °C. The warmed product mixture was filtered through a pad of silica gel and the pad was rinsed with ethyl acetate (50 mL). The filtrates were combined and the combined filtrates were concentrated. The residue obtained was purified by automated flash-column chromatography (eluting with 5% ethyl acetate–hexanes initially, grading to 10% ethyl acetate–hexanes, linear gradient) to afford benzyl 4-methylenepiperidine-1-carboxylate (**1f**) as a clear oil (1.78 g, 77%).

R_f = 0.27 (20% ethyl acetate–hexanes; UV, $KMnO_4$). ^1H NMR (600 MHz, $CDCl_3$) δ 7.36–7.30 (m, 5H, ArH), 5.14 (s, 2H, H_5), 4.75 (s, 2H, H_1), 3.50 (br s, 4H, H_2), 2.19 (br s, 4H, H_3). ^{13}C NMR (150 MHz, $CDCl_3$) δ 155.1 (C), 144.8 (C), 136.8 (C), 128.4 (CH), 127.9 (CH), 127.8 (CH), 109.4 (CH_2), 67.1 (CH_2), 45.5 (CH_2), 34.5 (CH_2).

^1H and ^{13}C NMR data for benzyl 4-methylenepiperidine-1-carboxylate (**1f**) prepared in this way were in agreement with those previously described.[15]

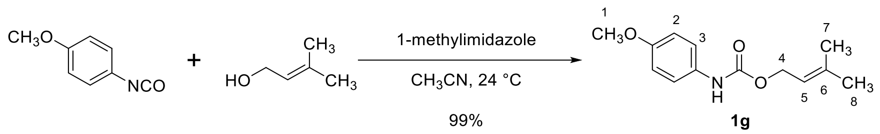

*Preparation of 3-methylbut-2-en-1-yl (4-methoxyphenyl)carbamate (**1g**):*

1-Methylimidazole (71.4 µL, 900 µmol, 0.300 equiv) and 4-methoxyphenyl isocyanate (386 µL, 3.00 mmol, 1.00 equiv) were added in sequence to a solution of 3-methylbut-2-en-1-ol (284 mg, 3.00 mmol, 1 equiv) in acetonitrile (7.5 mL) at 24 °C. The reaction mixture was stirred for 12 h at 24 °C. The product mixture was concentrated and the residue obtained was purified by automated flash-column chromatography (eluting with 10% ethyl acetate–hexanes initially, grading to 33% ethyl acetate–hexanes, linear gradient) to afford 3-methylbut-2-en-1-yl (4-methoxyphenyl)carbamate (**1g**) as a white solid (705 mg, 99%).

R_f = 0.25 (20% ethyl acetate–hexanes; UV, CAM). ^{1}H NMR (600 MHz, $CDCl_3$) δ 7.28–7.26 (m, 2H, H_3), 6.84 (d, 2H, J = 9.0 Hz, H_2), 6.49 (br s, NH), 5.41–5.37 (m, 1H, H_5), 4.65 (d, 2H, J = 7.0 Hz, H_4), 3.78 (s, 3H, H_1), 1.77 (s, 3H, H_7), 1.74 (s, 3H, H_8). ^{13}C NMR (150 MHz, $CDCl_3$) δ 155.6 (C), 153.7 (C), 138.9 (C), 130.8 (C), 120.4 (CH), 118.6 (CH), 114.0 (CH), 61.7 (CH_2), 55.2 (CH_3), 25.5 (CH_3), 17.8 (CH_3). IR (ATR-FTIR), cm^{-1}: 3315 (w), 1696 (s), 1511 (s), 1209 (s), 826 (s). HRMS-ESI (m/z): $[M + H]^+$ calcd for $C_{13}H_{18}NO_3$, 236.1287; found, 236.1278.

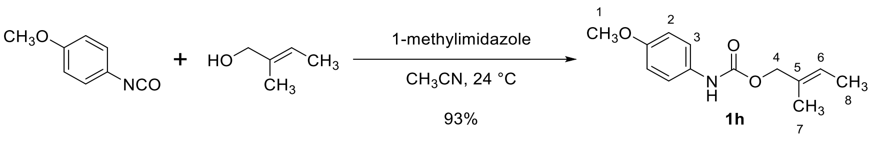

*Preparation of (E)-2-methylbut-2-en-1-yl (4-methoxyphenyl)carbamate (**1h**):*

1-Methylimidazole (71.4 µL, 900 µmol, 0.300 equiv) and 4-methoxyphenyl isocyanate (386 µL, 3.00 mmol, 1.00 equiv) were added in sequence to a solution of (*E*)-2-methylbut-2-en-1-ol (284 mg, 3.00 mmol, 1 equiv) in acetonitrile (7.5 mL) at 24 °C. The reaction mixture was stirred for 12 h at 24 °C. The product mixture was concentrated and the residue obtained was purified by automated flash-column chromatography (eluting with 10% ethyl acetate–hexanes initially, grading to 33% ethyl acetate–hexanes, linear gradient) to afford (*E*)-2-methylbut-2-en-1-yl (4-methoxyphenyl)carbamate (**1h**) as a white solid (654 mg, 93%).

R_f = 0.25 (20% ethyl acetate–hexanes; UV, CAM). ^{1}H NMR (400 MHz, CD_2Cl_2) δ 7.31 (d, 2H, J = 8.0 Hz, H_3), 6.87-6.85 (m, 3H, 2 × H_2, 1 × NH), 5.60 (t, 1H, J = 6.6 Hz, H_6), 4.54 (s, 2H, H_4), 3.78 (s, 3H, H_1), 1.70 (s, 3H, H_7), 1.66 (d, 3H, J = 6.8 Hz, H_8). ^{13}C NMR (150 MHz, CD_2Cl_2) δ 156.4 (C), 154.5 (C), 131.9 (C), 131.8 (C), 124.2 (CH), 121.1 (CH), 114.6 (CH), 71.2 (CH_2), 55.9 (CH_3), 13.9 (CH_3), 13.5 (CH_3). IR (ATR-FTIR), cm^{-1}: 3320 (s), 1689 (s), 1525 (s), 1232 (m), 1028 (m). HRMS-ESI (m/z): $[M + H]^+$ calcd for $C_{13}H_{18}NO_3$, 236.1287; found, 236.1284.

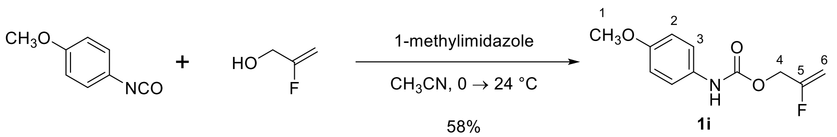

*Preparation of 2-fluoroallyl (4-methoxyphenyl)carbamate (**1i**):*

1-Methylimidazole (238 μL, 3.00 mmol, 0.300 equiv) and 4-methoxyphenyl isocyanate (1.29 mL, 10.0 mmol, 1.00 equiv) were added in sequence to a solution of 2-fluoroprop-2-en-1-ol (761 mg, 10.0 mmol, 1 equiv) in acetonitrile (25 mL) at 24 °C. The reaction mixture was stirred for 36 h at 24 °C. The product mixture was concentrated and the residue obtained was purified by automated flash-column chromatography (eluting with 8% ethyl acetate–hexanes initially, grading to 33% ethyl acetate–hexanes, linear gradient) to afford 2-fluoroallyl (4-methoxyphenyl)carbamate (**1i**) as a white solid (1.30 g, 58%).

R_f = 0.65 (33% ethyl acetate–hexanes; UV, $KMnO_4$). ^1H NMR (600 MHz, $CDCl_3$) δ 7.29 (d, 2H, J = 7.2 Hz, H_3), 6.94 (br s, 1H, NH), 6.84 (d, 2H, J = 8.1 Hz, H_2), 4.81 (dd, 1H, J = 15.9, 3.3 Hz, H_6), 4.71–4.60 (m, 3H, 2 × H_4, 1 × H_6), 3.77 (s, 3H, H_1). ^{19}F NMR (375 MHz, $CDCl_3$) δ –105.36.

^1H and ^{19}F NMR data for 2-fluoroallyl (4-methoxyphenyl)carbamate (**1i**) prepared in this way were in agreement with those previously described.[14]

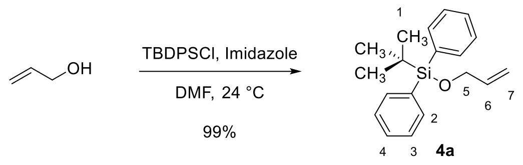

*Preparation of (allyloxy)(tert-butyl)diphenylsilane (**4a**):*

Imidazole (1.02 g, 15.0 mmol, 1.50 equiv) and *tert*-butyl(chloro)diphenylsilane (3.02 g, 11.0 mmol, 1.10 equiv) were added in sequence to a solution of allyl alcohol (580 mg, 10.0 mmol, 1 equiv) in *N,N*-dimethylformamide (8.0 mL) at 24 °C. The reaction mixture was stirred for 4 h at 24 °C. The product mixture was transferred to a separatory funnel that had been charged with ethyl acetate (50 mL). The diluted product mixture was washed with saturated aqueous ammonium chloride solution (50 mL). The aqueous layer was isolated and the isolated aqueous layer was extracted with ethyl acetate (3 × 20 mL). The organic layers were combined and the combined organic layers were dried over sodium sulfate. The dried solution was filtered and the filtrate was concentrated. The residue obtained was purified by automated flash-column chromatography (eluting with 1% ether–hexanes initially, grading to 5% ether–hexanes, linear gradient) to afford (allyloxy)(*tert*-butyl)diphenylsilane (**4a**) as a clear oil (2.96 g, 99%).

R_f = 0.75 (5% ether–hexanes; UV, $KMnO_4$). ^1H NMR (400 MHz, $CDCl_3$) δ 7.72–7.69 (m, 4H, H_3), 7.46–7.37 (m, 6H, 4 × H_2, 2 × H_4), 5.99–5.90 (m, 1H, H_6), 5.42–5.37 (m, 1H, H_7), 5.15–5.12 (m, 1H, H_7), 4.24–4.22 (m, 2H, H_5), 1.09 (s, 9H, H_1). ^{13}C NMR (100 MHz, $CDCl_3$) δ 137.0 (C), 135.5 (CH), 133.7 (CH), 129.6 (CH), 127.6 (CH), 113.9 (CH_2), 64.6 (CH_2), 26.8 (CH_3), 19.3 (C).

^1H and ^{13}C NMR data for (allyloxy)(*tert*-butyl)diphenylsilane (**4a**) prepared in this way were in agreement with those previously described.[16]

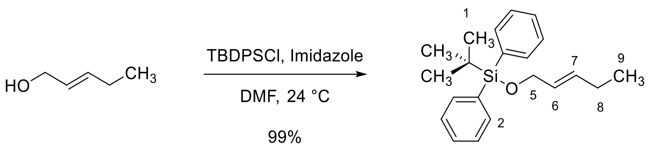

*Preparation of (E)-tert-butyl(pent-2-en-1-yloxy)diphenylsilane (**4b**):*

Imidazole (510 mg, 7.50 mmol, 1.50 equiv) and *tert*-butyl(chloro)diphenylsilane (1.51 g, 5.50 mmol, 1.10 equiv) were added in sequence to a solution of (*E*)-pent-2-en-1-ol (430 mg, 5.00 mmol, 1 equiv) in *N*,*N*-dimethylformamide (4.0 mL) at 24 °C. The reaction mixture was stirred for 4 h at 24 °C. The product mixture was transferred to a separatory funnel that had been charged with ethyl acetate (25 mL). The diluted product mixture was washed with saturated aqueous ammonium chloride solution (25 mL). The aqueous layer was isolated and the isolated aqueous layer was extracted with ethyl acetate (3 × 20 mL). The organic layers were combined and the combined organic layers were dried over sodium sulfate. The dried solution was filtered and the filtrate was concentrated. The residue obtained was purified by automated flash-column chromatography (eluting with 1% ether–hexanes initially, grading to 5% ether–hexanes, linear gradient) to afford (*E*)-*tert*-butyl(pent-2-en-1-yloxy)diphenylsilane (**4b**) as a clear oil (1.62 g, 99%).

R_f = 0.75 (5% ether–hexanes; UV, $KMnO_4$). ^1H NMR (400 MHz, $CDCl_3$) δ 7.75–7.72 (m, 4H, H_3), 7.45–7.40 (m, 6H, 4 × H_2, 2 × H_4), 5.75–5.70 (m, 1H, H_7), 5.61–5.59 (m, 1H, H_6), 4.22–4.19 (m, 2H, H_5), 2.11–2.05 (m, 2H, H_8), 1.12–1.09 (m, 9H, H_1), 1.06–0.99 (m, 3H, H_9). ^{13}C NMR (100 MHz, $CDCl_3$) δ 135.6 (CH), 133.9 (C), 132.9 (CH), 129.5 (CH), 127.7 (CH), 127.6 (CH), 64.7 (CH_2), 26.9 (CH_3), 25.2 (CH_2), 19.2 (C), 13.5 (CH_3). IR (ATR-FTIR), cm^{-1}: 2960 (m), 1428 (m), 1105 (s), 699 (s), 502 (m). HRMS-ESI (m/z): $[M + H]^+$ calcd for $C_{21}H_{29}OSi$, 325.1988; found, 325.1983.

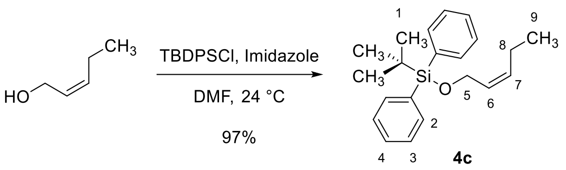

*Preparation of (Z)-tert-butyl(pent-2-en-1-yloxy)diphenylsilane (**4c**):*

Imidazole (510 mg, 7.50 mmol, 1.50 equiv) and *tert*-butyl(chloro)diphenylsilane (1.51 g, 5.50 mmol, 1.10 equiv) were added in sequence to a solution of (*Z*)-pent-2-en-1-ol (430 mg, 5.00 mmol, 1 equiv) in *N*,*N*-dimethylformamide (4.0 mL) at 24 °C. The reaction mixture was stirred for 4 h at 24 °C. The product mixture was transferred to a separatory funnel that had been charged with ethyl acetate (25 mL). The diluted product mixture was washed with saturated aqueous ammonium chloride solution (25 mL). The aqueous layer was isolated and the isolated aqueous layer was extracted with ethyl acetate (3 × 20 mL). The organic layers were combined and the combined organic layers were dried over sodium sulfate. The dried solution was filtered and the filtrate was concentrated. The residue obtained was purified by automated flash-column chromatography (eluting with 1% ether–hexanes initially, grading to 5% ether–hexanes, linear gradient) to afford (*Z*)-*tert*-butyl(pent-2-en-1-yloxy)diphenylsilane (**4c**) as a clear oil (1.57 g, 97%).

R_f = 0.75 (5% ether–hexanes; UV, $KMnO_4$). ^{1}H NMR (400 MHz, $CDCl_3$) δ 7.74–7.72 (m, 4H, H_3), 7.44–7.41 (m, 6H, 4 × H_2, 2 × H_4), 5.64–5.58 (m, 1H, H_7), 5.47–5.42 (m, 1H, H_6), 4.30–4.29 (m, 2H, H_5), 1.94–1.89 (m, 2H, H_8), 1.10–1.08 (m, 9H, H_1), 0.94–0.89 (m, 3H, H_9). ^{13}C NMR (100 MHz, $CDCl_3$) δ 135.6 (CH), 133.9 (C), 132.7 (CH), 129.5 (CH), 128.4 (CH), 127.6 (CH), 60.2 (CH_2), 26.8 (CH_3), 20.8 (CH_2), 19.1 (C), 14.2 (CH_3). IR (ATR-FTIR), cm^{-1}: 2961 (m), 1428 (s), 1070 (m), 698 (s), 502 (m). HRMS-ESI (m/z): $[M + H]^+$ calcd for $C_{21}H_{29}OSi$, 325.1988; found, 325.1986.

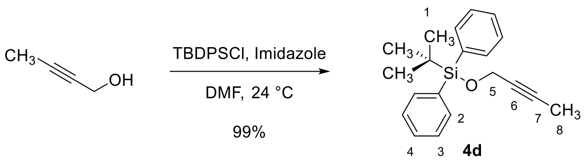

*Preparation of (but-2-yn-1-yloxy)(tert-butyl)diphenylsilane (**4d**):*

Imidazole (510 mg, 7.50 mmol, 1.50 equiv) and *tert*-butyl(chloro)diphenylsilane (1.51 g, 5.50 mmol, 1.10 equiv) were added in sequence to a solution of 2-butyn-1-ol (350 mg, 5.00 mmol, 1 equiv) in *N*,*N*-dimethylformamide (4.0 mL) at 24 °C. The reaction mixture was stirred for 4 h at 24 °C. The product mixture was transferred to a separatory funnel that had been charged with ethyl acetate (25 mL). The diluted product mixture was washed with saturated aqueous ammonium chloride solution (25 mL). The aqueous layer was isolated and the isolated aqueous layer was extracted with ethyl acetate (3 × 20 mL). The organic layers were combined and the combined organic layers were dried over sodium sulfate. The dried solution was filtered and the filtrate was concentrated. The residue obtained was purified by automated flash-column chromatography (eluting with 1% ether–hexanes initially, grading to 5% ether–hexanes, linear gradient) to afford (but-2-yn-1-yloxy)(*tert*-butyl)diphenylsilane (**4d**) as a clear oil (1.52 g, 99%).

R_f = 0.75 (5% ether–hexanes; UV, $KMnO_4$). ^{1}H NMR (400 MHz, $CDCl_3$) δ 7.73–7.72 (m, 4H, H_3), 7.44–7.39 (m, 6H, 4 × H_2, 2 × H_4), 4.30 (s, 2H, H_5), 1.81 (s, 3H, H_8), 1.07 (s, 9H, H_1). ^{13}C NMR (100 MHz, $CDCl_3$) δ 135.6 (CH), 133.3 (C), 129.7 (CH), 127.6 (CH), 81.2 (C), 77.5 (C), 52.9 (CH_2), 26.7 (CH_3), 19.2 (C), 3.6 (CH_3). IR (ATR-FTIR), cm^{-1}: 2930 (m), 1428 (s), 1062 (s), 698 (s), 500 (m). HRMS-ESI (m/z): $[M + Na]^+$ calcd for $C_{20}H_{24}NaOSi$, 331.1494; found, 331.1498.

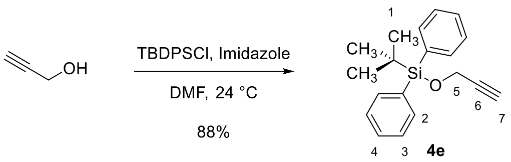

*Preparation of tert-butyldiphenyl(prop-2-yn-1-yloxy)silane (**4e**):*

Imidazole (510 mg, 7.5 mmol, 1.50 equiv) and *tert*-butyl(chloro)diphenylsilane (1.51 g, 5.50 mmol, 1.10 equiv) were added in sequence to a solution of propargyl alcohol (280 mg, 5.00 mmol, 1 equiv) in *N*,*N*-dimethylformamide (4.0 mL) at 24 °C. The reaction mixture was stirred for 4 h at 24 °C. The product mixture was transferred to a separatory funnel that had been charged with ethyl acetate (25 mL). The diluted product mixture was washed with saturated aqueous ammonium chloride solution (25 mL). The aqueous layer was isolated and the isolated aqueous layer was extracted with ethyl acetate (3 × 20 mL). The organic layers were combined and the combined organic layers were dried over sodium sulfate. The dried solution was filtered and the filtrate was concentrated. The residue obtained was purified by automated flash-column chromatography (eluting with 1% ether–hexanes initially, grading to 5% ether–hexanes, linear gradient) to afford *tert*-butyldiphenyl(prop-2-yn-1-yloxy)silane (**4e**) as a colorless solid (1.29 g, 88%).

R_f = 0.75 (5% ether–hexanes; UV, $KMnO_4$). ^1H NMR (600 MHz, $CDCl_3$) δ 7.74–7.72 (m, 4H, H_3), 7.47–7.40 (m, 6H, 4 × H_2, 2 × H_4), 4.33 (d, J = 2.4 Hz, 2H, H_5), 2.40 (t, J = 2.4 Hz, 1H, H_7), 1.09 (s, 9H, H_1). ^{13}C NMR (150 MHz, $CDCl_3$) δ 135.6 (CH), 132.9 (C), 129.8 (CH), 127.7 (CH), 82.0 (C), 73.0 (CH), 52.5 (CH_2), 26.7 (CH_3), 19.1 (C)

^1H and ^{13}C NMR data for *tert*-butyldiphenyl(prop-2-yn-1-yloxy)silane (**4e**) prepared in this way were in agreement with those previously described.[17]

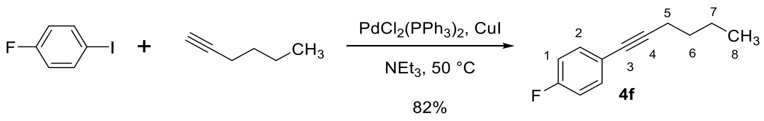

*Preparation of 1-fluoro-4-(hex-1-yn-1-yl)benzene (**4f**):*

A 25-mL round-bottomed flask that had been fused to a Teflon-coated valve was charged sequentially with 1-fluoro-4-iodobenzene (1.11 g, 5.00 mmol, 1 equiv), bis(triphenylphosphine)palladium dichloride (175 mg, 250 μmol, 0.0500 equiv), and copper iodide (95.2 mg, 500 μmol, 0.100 equiv). The reaction vessel was evacuated and refilled using a balloon of argon. This process was repeated twice. Triethylamine (10.0 mL) and 1-hexyne (860 μL, 7.50 mmol, 1.50 equiv) were added sequentially to the reaction vessel. The reaction vessel was sealed and the sealed vessel was placed in an oil bath that had been preheated to 50 °C. The reaction mixture was stirred and heated for 4 h at 50 °C. The product mixture was allowed to cool over 20 min to 24 °C. The cooled product mixture was filtered through a pad of silica gel and the pad was rinsed with ethyl acetate (100 mL). The filtrates were combined and the combined filtrates were concentrated. The residue obtained was purified by automated flash-column chromatography (eluting with hexanes, isocratic gradient) to afford 1-fluoro-4-(hex-1-yn-1-yl)benzene (**4f**) as a light yellow oil (723 mg, 82%).

R_f = 0.40 (hexanes; UV, $KMnO_4$). ^{1}H NMR (500 MHz, $CDCl_3$) δ 7.34 (dd, J = 8.4, 5.5 Hz, 2H, H_1), 6.98–6.47 (m, 2H, H_2), 2.39 (t, J = 7.3 Hz, 2H, H_5), 1.61–1.55 (m, 2H, H_7), 1.51–1.44 (m, 2H, H_6), 0.95 (t, J = 7.5 Hz, 3H, H_8). ^{19}F NMR (470 MHz, $CDCl_3$) δ -112.5.

^{1}H and ^{19}F NMR data for 1-fluoro-4-(hex-1-yn-1-yl)benzene (**4f**) prepared in this way were in agreement with those previously described.[18]

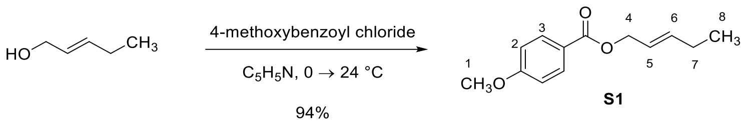

*Preparation of (E)-pent-2-en-1-yl 4-methoxybenzoate (**S1**):*

4-Methoxybenzoyl chloride (563 mg, 3.30 mmol, 1.10 equiv) was added dropwise via syringe to a solution of (*E*)-pent-2-en-1-ol (258 mg, 3.00 mmol, 1 equiv) in pyridine (12 mL) at 0 °C. The reaction mixture was stirred for 30 min at 0 °C, and then the ice bath was removed. The reaction mixture was stirred for 24 h at 24 °C. The product mixture was transferred to a separatory funnel that had been charged with ethyl acetate (20 mL). The diluted product mixture was washed with saturated aqueous sodium bicarbonate solution (20 mL). The aqueous layer was isolated and the isolated aqueous layer was extracted with ethyl acetate (3 × 20 mL). The organic layers were combined and the combined organic layers were dried over sodium sulfate. The dried solution was filtered and the filtrate was concentrated. The residue obtained was purified by automated flash-column chromatography (eluting with 5% ethyl acetate–hexanes initially, grading to 10% ethyl acetate–hexanes, linear gradient) to afford (*E*)-pent-2-en-1-yl 4-methoxybenzoate (**S1**) as a clear oil (621 mg, 94%).

R_f = 0.55 (20% ethyl acetate–hexanes; UV, $KMnO_4$). ^{1}H NMR (500 MHz, CD_2Cl_2) δ 7.99 (d, 2H, J = 8.5 Hz, H_3), 6.92 (d, 2H, J = 9.0 Hz, H_2), 5.93–5.88 (m, 1H, H_6), 5.71–5.66 (m, 1H, H_5), 4.72 (d, J = 6.5 Hz, 2H, H_4), 3.85 (s, 3H, H_1), 2.14–2.08 (m, 2H, H_7), 1.03 (t, J = 7.5 Hz, 3H, H_8). ^{13}C NMR (125 MHz, CD_2Cl_2) δ 166.4 (C), 164.0 (C), 138.1 (CH), 132.0 (CH), 123.8 (C), 123.5 (CH), 114.1 (CH), 65.9 (CH_2), 56.0 (CH_3), 25.9 (CH_2), 13.6 (CH_3). IR (ATR-FTIR), cm^{-1}: 2966 (m), 1708 (s), 1605 (s), 1511 (s), 1249 (s), 1096 (s). HRMS-ESI (m/z): $[M + Na]^+$ calcd for $C_{13}H_{16}NaO_3$, 243.0997; found, 243.0996.

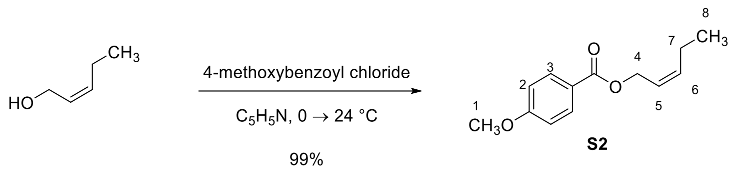

*Preparation of (Z)-pent-2-en-1-yl 4-methoxybenzoate (**S2**):*

4-Methoxybenzoyl chloride (563 mg, 3.30 mmol, 1.10 equiv) was added dropwise via syringe to a solution of (*Z*)-pent-2-en-1-ol (258 mg, 3.00 mmol, 1 equiv) in pyridine (12 mL) at 0 °C. The reaction mixture was stirred for 30 min at 0 °C, and then the ice bath was removed. The reaction mixture was stirred for 24 h at 24 °C. The product mixture was transferred to a separatory funnel that had been charged with ethyl acetate (20 mL). The diluted product mixture was washed with saturated aqueous sodium bicarbonate solution (20 mL). The aqueous layer was isolated and the isolated aqueous layer was extracted with ethyl acetate (3 × 20 mL). The organic layers were combined and the combined organic layers were dried over sodium sulfate. The dried solution was filtered and the filtrate was concentrated. The residue obtained was purified by automated flash-column chromatography (eluting with 5% ethyl acetate–hexanes initially, grading to 10% ethyl acetate–hexanes, linear gradient) to afford (*Z*)-pent-2-en-1-yl 4-methoxybenzoate (**S2**) as a clear oil (661 mg, 99%).

R_f = 0.55 (20% ethyl acetate–hexanes; UV, $KMnO_4$). ^1H NMR (500 MHz, CD_2Cl_2) δ 7.99 (d, 2H, J = 9.0 Hz, H_3), 6.94 (d, 2H, J = 9.0 Hz, H_2), 5.73–5.68 (m, 1H, H_6), 5.66–5.61 (m, 1H, H_5), 4.82 (d, J = 7.0 Hz, 2H, H_4), 3.86 (s, 3H, H_1), 2.23–2.17 (m, 2H, H_7), 1.03 (t, J = 7.5 Hz, 3H, H_8). ^{13}C NMR (125 MHz, CD_2Cl_2) δ 166.6 (C), 164.0 (C), 137.4 (CH), 132.0 (CH), 123.6 (C), 123.4 (CH), 114.1 (CH), 61.0 (CH_2), 56.0 (CH_3), 21.5 (CH_2), 14.5 (CH_3). IR (ATR-FTIR), cm^{-1}: 2965 (m), 1708 (m), 1605 (s), 1249 (s), 1096 (s). HRMS-ESI (m/z): $[M + Na]^+$ calcd for $C_{13}H_{16}NaO_3$, 243.0997; found, 243.1002.

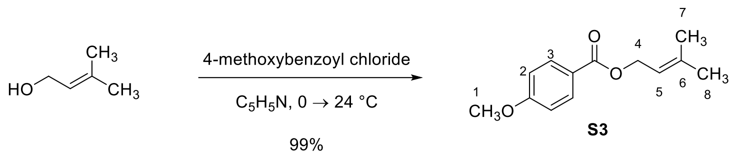

*Preparation of 3-methylbut-2-en-1-yl 4-methoxybenzoate (**S3**):*

4-Methoxybenzoyl chloride (563 mg, 3.30 mmol, 1.10 equiv) was added dropwise via syringe to a solution of 3-methylbut-2-en-1-ol (258 mg, 3.00 mmol, 1 equiv) in pyridine (12 mL) at 0 °C. The reaction mixture was stirred for 30 min at 0 °C, and then the ice bath was removed. The reaction mixture was stirred for 24 h at 24 °C. The product mixture was transferred to a separatory funnel that had been charged with ethyl acetate (20 mL). The diluted product mixture was washed with saturated aqueous sodium bicarbonate solution (20 mL). The aqueous layer was isolated and the isolated aqueous layer was extracted with ethyl acetate (3 × 20 mL). The organic layers were combined and the combined organic layers were dried over sodium sulfate. The dried solution was filtered and the filtrate was concentrated. The residue obtained was purified by automated flash-column chromatography (eluting with 5% ethyl acetate–hexanes initially, grading to 10% ethyl acetate–hexanes, linear gradient) to afford 3-methylbut-2-en-1-yl 4-methoxybenzoate (**S3**) as a clear oil (661 mg, 99%).

R_f = 0.55 (20% ethyl acetate–hexanes; UV, $KMnO_4$). ^{1}H NMR (500 MHz, CD_2Cl_2) δ 7.98 (d, 2H, J = 8.5 Hz, H_3), 6.94 (d, 2H, J = 9.0 Hz, H_2), 5.47 (t, J = 7.0 Hz, 1H, H_5), 4.78 (d, J = 7.5 Hz, 2H, H_4), 3.86 (s, 3H, H_1), 1.80 (s, 3H, H_7), 1.78 (s, 3H, H_8). ^{13}C NMR (125 MHz, CD_2Cl_2) δ 166.6 (C), 163.9 (C), 139.4 (C), 131.9 (CH), 123.6 (C), 119.5 (CH), 114.1 (CH), 62.0 (CH_2), 56.0 (CH_3), 26.0 (CH_3), 18.4 (CH_3). IR (ATR-FTIR), cm^{-1}: 2935 (w), 1707 (s), 1606 (s), 1251 (s), 1096 (s). HRMS-ESI (m/z): $[M + Na]^+$ calcd for $C_{12}H_{12}NaO_3$, 243.0997; found, 243.1003.

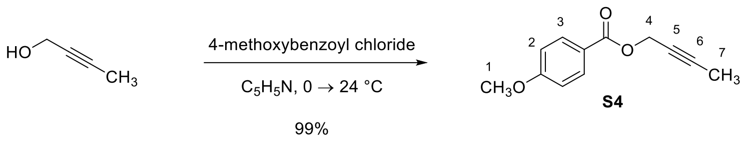

*Preparation of but-2-yn-1-yl 4-methoxybenzoate (**S4**):*

4-Methoxybenzoyl chloride (563 mg, 3.30 mmol, 1.10 equiv) was added dropwise via syringe to a solution of but-2-yn-1-ol (210 mg, 3.00 mmol, 1 equiv) in pyridine (12 mL) at 0 °C. The reaction mixture was stirred for 30 min at 0 °C, and then the ice bath was removed. The reaction mixture was stirred for 24 h at 24 °C. The product mixture was transferred to a separatory funnel that had been charged with ethyl acetate (20 mL). The diluted product mixture was washed with saturated aqueous sodium bicarbonate solution (20 mL). The aqueous layer was isolated and the isolated aqueous layer was extracted with ethyl acetate (3 × 20 mL). The organic layers were combined and the combined organic layers were dried over sodium sulfate. The dried solution was filtered and the filtrate was concentrated. The residue obtained was purified by automated flash-column chromatography (eluting with 5% ethyl acetate–hexanes initially, grading to 10% ethyl acetate–hexanes, linear gradient) to afford but-2-yn-1-yl 4-methoxybenzoate (**S4**) as a clear oil (612 mg, 99%).

R_f = 0.55 (20% ethyl acetate–hexanes; UV, $KMnO_4$). ^{1}H NMR (500 MHz, CD_2Cl_2) δ 7.99 (d, 2H, J = 9.0 Hz, H_3), 6.94 (d, 2H, J = 9.0 Hz, H_2), 4.83 (s, 2H, H_4), 3.86 (s, 3H, H_1), 1.87 (s, 3H, H_7). ^{13}C NMR (125 MHz, CD_2Cl_2) δ 166.0 (C), 164.2 (C), 132.2 (CH), 122.7 (C), 114.2 (CH), 83.4 (C), 74.0 (C), 56.0 (CH_3), 53.4 (CH_2), 3.9 (CH_3). IR (ATR-FTIR), cm^{-1}: 2938 (w), 1710 (m), 1605 (s), 1248 (s), 1091 (s). HRMS-ESI (m/z): $[M + Na]^+$ calcd for $C_{12}H_{12}NaO_3$, 227.0684; found, 227.0685.

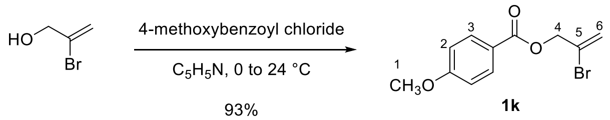

*Preparation of 2-bromoallyl 4-methoxybenzoate (**1k**):*

4-Methoxybenzoyl chloride (938 mg, 5.50 mmol, 1.10 equiv) was added dropwise via syringe to a solution of 2-bromoallyl alcohol (685 mg, 5.00 mmol, 1 equiv) in pyridine (20 mL) at 0 °C. The reaction mixture was stirred for 30 min at 0 °C, and then the ice bath was removed. The reaction mixture was stirred for 24 h at 24 °C. The product mixture was transferred to a separatory funnel that had been charged with ethyl acetate (20 mL). The diluted product mixture was washed with saturated aqueous sodium bicarbonate solution (20 mL). The aqueous layer was isolated and the isolated aqueous layer was extracted with ethyl acetate (3 × 20 mL). The organic layers were combined and the combined organic layers were dried over sodium sulfate. The dried solution was filtered and the filtrate was concentrated. The residue obtained was purified by automated flash-column chromatography (eluting with 5% ethyl acetate–hexanes initially, grading to 10% ethyl acetate–hexanes, linear gradient) to afford 2-bromoallyl 4-methoxybenzoate (**1k**) as a clear oil (1.26g, 93%).

R_f = 0.51 (20% ethyl acetate–hexanes; UV, $KMnO_4$). ^{1}H NMR (400 MHz, CD_2Cl_2) δ 8.03 (d, 2H, J = 8.8 Hz, H_3), 6.96 (d, 2H, J = 8.8 Hz, H_2), 6.00 (br s, 1H, H_6), 5.70 (br s, 1H, H_6), 4.92 (s, 2H, H_4), 3.86 (s, 3H, H_1). ^{13}C NMR (100 MHz, CD_2Cl_2) δ 165.6 (C), 164.3 (C), 132.2 (CH), 127.2 (C), 122.4 (C), 119.3 (CH_2), 114.3 (CH), 68.1 (CH_2), 56.0 (CH_3). IR (ATR-FTIR), cm^{-1}: 1714 (s), 1604 (s), 1510 (m), 1249 (s), 1165 (s). HRMS-ESI (m/z): $[M + H]^+$ calcd for $C_{11}H_{12}{}^{79/81}BrO_3$, 270.9970/272.9949; found, 270.9975/272.9943.

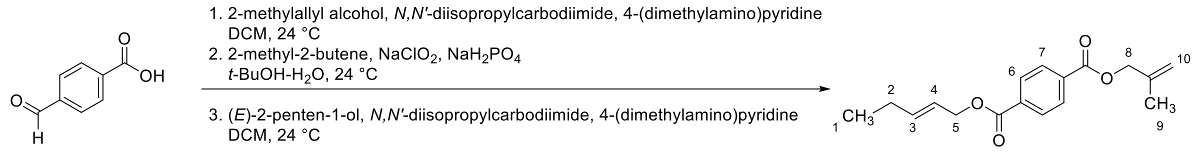

9

*Preparation of (E)-2-methylallyl pent-2-en-1-yl terephthalate (**9**):*

N,N'-Diisopropylcarbodiimide (1.55 mL, 10.0 mmol, 2.00 equiv) and 4-dimethylaminopyridine (122 mg, 1.00 mmol, 0.200 equiv) were added sequentially to a suspension of 2-methylallyl alcohol (371 mg, 5.00 mmol, 1 equiv) and 4-formylbenzoic acid (750 mg, 5.00 mmol, 1 equiv) in dichloromethane (25 mL) at 24 °C. The reaction mixture was stirred for 2 h at 24 °C. The product mixture was concentrated and the residue obtained was diluted with a mixture of ethyl acetate and hexanes (1:4, v/v, 100 mL). The diluted product mixture was filtered through a pad of celite and the pad was rinsed with a mixture of ethyl acetate and hexanes (1:4, v/v, 100 mL). The filtrates were collected and combined. The combined filtrate was concentrated. The 2-methylallyl 4-formylbenzoate prepared in this way was immediately used in the following step without further purification.

2-Methyl-2-butene (6.36mL, 60.0 mmol, 12.0 equiv) and a solution of sodium chlorite (3.00 g, 33.3 mmol, 6.65 equiv) and sodium phosphate monobasic (3.68 g, 26.7 mmol, 5.34 equiv) in water (22.7 mL) were added sequentially to a suspension of 2-methylallyl 4-formylbenzoate (nominally, 5.00 mmol) in *t*-butanol (71.4 mL) at 24 °C. The reaction mixture was stirred for 30 min at 24 °C. The product mixture was transferred to a separatory funnel that had been charged with ethyl acetate (250 mL). The diluted product mixture was washed with saturated ammonium chloride (3 × 50 mL). The organic layer were combined and the combined organic layers were dried over sodium sulfate. The dried solution was filtered and the filtrate was concentrated. The 4-(((2-methylallyl)oxy)carbonyl)benzoic acid prepared in this way was immediately used in the following step without further purification.

N,N'-Diisopropylcarbodiimide (1.55 mL, 10.0 mmol, 2.00 equiv) and 4-dimethylaminopyridine (122 mg, 1.00 mmol, 0.200 equiv) were added sequentially to a suspension of (*E*)-2-penten-1-ol (517 mg, 6.00 mmol, 1.20 equiv) and 4-(((2-methylallyl)oxy)carbonyl)benzoic acid (nominally, 5.00 mmol) in dichloromethane (25 mL) at 24 °C. The reaction mixture was stirred for 2 h at 24 °C. The product mixture was concentrated and the residue obtained was diluted with a mixture of ethyl acetate and hexanes (1:4, v/v, 200 mL). The diluted product mixture was filtered through a pad of celite and the pad was rinsed with a mixture of ethyl acetate and hexanes (1:4, v/v, 250 mL). The filtrates were collected and combined. The combined filtrate was concentrated and the residue obtained was purified by flash-column chromatography (eluting with 5% ethyl acetate–hexanes, isocratic gradient) to afford (*E*)-2-methylallyl pent-2-en-1-yl terephthalate (**9**) as a light yellow oil (579 mg, 40%)

R_f = 0.47 (10% ethyl acetate–hexanes; UV, $KMnO_4$). ^1H NMR (500 MHz, $CDCl_3$) δ 8.11–8.10 (m, 4H, 2 × H_6, 2 × H_7), 5.94–5.87 (m, 1H, H_3), 5.70–5.63 (m, 1H, H_4), 5.07 (s, 1H, H_{10}), 4.98 (s, 1H, H_{10}), 4.78–4.75 (m, 4H, 2 × H_5, 2 × H_8), 2.11–2.08 (m, 2H, H_2), 1.83 (s, 3H, H_1), 1.01 (t, 3H, J = 10.0 Hz, H_9). ^{13}C NMR (125 MHz, $CDCl_3$) δ 165.5 (C), 165.3 (C), 139.6 (C), 138.4 (CH), 134.2 (C), 133.8 (C), 129.5 (CH), 129.4 (CH), 122.5 (CH), 113.2 (CH), 68.4 (CH_2), 66.1 (CH_2), 25.2 (CH_2), 19.5 (CH_3), 13.0 (CH_3). IR (ATR-FTIR), cm^{-1}: 2964 (w), 1717 (s), 1262 (s), 1242 (s), 1099 (s). HRMS-ESI (m/z): $[M + H]^+$ calcd for $C_{17}H_{21}O_4$, 289.1440; found, 289.1439.

Condition Optimization for the Hydrogenation (Table 1, Entry 1).

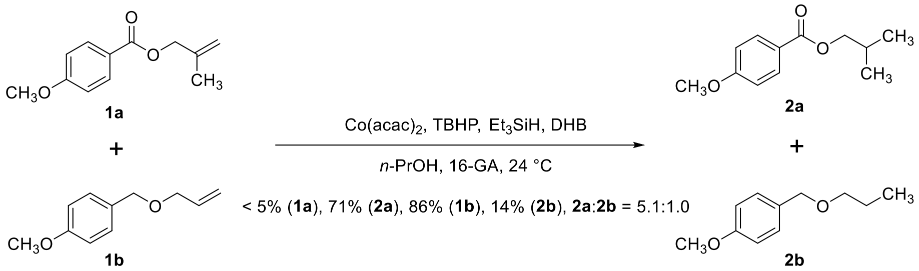

A 25-mL round-bottomed flask fitted with a rubber septum was charged sequentially with 2-methylallyl 4-methoxybenzoate (**1a**, 51.6 mg, 250 μmol, 1 equiv), 1-((allyloxy)methyl)-4-methoxybenzene (**1b**, 44.5 mg, 250 μmol, 1 equiv), and cobalt bis(acetylacetonate) (64.3 mg, 250 μmol, 1.00 equiv). A 16-gauge needle was penetrated through the septum. *n*-Propanol (830 μL), 1,4-dihydrobenzene (238 μL, 2.50 mmol, 10.0 equiv), a solution of *tert*-butyl hydroperoxide in nonane (~5.5 M, 45.5 μL, 250 μmol, 1.00 equiv), and triethylsilane (400 μL, 2.50 mmol, 10.0 equiv) were added sequentially to the reaction vessel via syringe. The reaction mixture was stirred at 24 °C and *tert*-butyl hydroperoxide in nonane (~5.5 M, 22.8 μL, 125 μmol, 0.500 equiv) was added every hour until the consumption of **1a** was complete (as determined by TLC analysis, 135 min for **1a**, 2.00 equiv of TBHP in total). The product mixture was filtered through a short column of silica gel and the short column was rinsed with 20% ethyl acetate–hexanes (100 mL). The filtrates were combined and the combined filtrates were concentrated. ^{1}H NMR analysis of the unpurified product mixture using mesitylene as an internal standard (25.4 mg, 212 μmol, 0.848 equiv) revealed a < 5% yield of 2-methylallyl 4-methoxybenzoate (**1a**), a 71% yield of isobutyl 4-methoxybenzoate (**2a**), an 86% yield of 1-((allyloxy)methyl)-4-methoxybenzene (**1b**), and a 14% yield of 1-methoxy-4-(propoxymethyl)benzene (**2b**).

Condition Optimization for the Hydrogenation (Table 1, Entry 2).

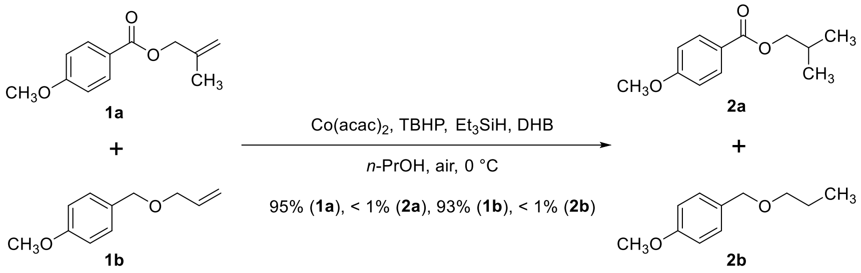

A 25-mL round-bottomed flask fitted with a rubber septum was charged sequentially with 2-methylallyl 4-methoxybenzoate (**1a**, 51.6 mg, 250 μmol, 1 equiv), 1-((allyloxy)methyl)-4-methoxybenzene (**1b**, 44.5 mg, 250 μmol, 1 equiv), and cobalt bis(acetylacetonate) (64.3 mg, 250 μmol, 1.00 equiv). The reaction vessel was equipped with a balloon of dry air and the reaction vessel was cooled to 0 °C. *n*-Propanol (830 μL), 1,4-dihydrobenzene (238 μL, 2.50 mmol, 10.0 equiv), a solution of *tert*-butyl hydroperoxide in nonane (~5.5 M, 45.5 μL, 250 μmol, 1.00 equiv), and triethylsilane (400 μL, 2.50 mmol, 10.0 equiv) were added sequentially to the reaction vessel via syringe. The reaction mixture was stirred at 0 °C for 300 min. The product mixture was filtered through a short column of silica gel and the short column was rinsed with 20% ethyl acetate–hexanes (100 mL). The filtrates were combined and the combined filtrates were concentrated. ^{1}H NMR analysis of the unpurified product mixture using 1,3,5-trimethoxybenzene as an internal standard (35.7 mg, 241 μmol, 0.964 equiv) revealed a 95% yield of 2-methylallyl 4-methoxybenzoate (**1a**), a <5% yield of isobutyl 4-methoxybenzoate (**2a**), a 93% yield of 1-((allyloxy)methyl)-4-methoxybenzene (**1b**), and a < 1% yield of 1-methoxy-4-(propoxymethyl)benzene (**2b**).

Condition Optimization for the Hydrogenation (Table 1, Entry 3).

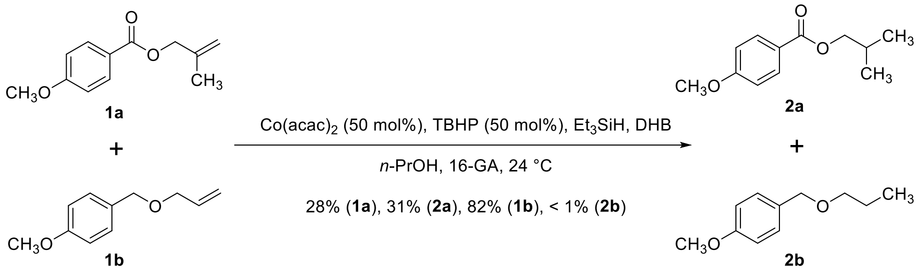

A 25-mL round-bottomed flask fitted with a rubber septum was charged sequentially with 2-methylallyl 4-methoxybenzoate (**1a**, 51.6 mg, 250 µmol, 1 equiv), 1-((allyloxy)methyl)-4-methoxybenzene (**1b**, 44.5 mg, 250 µmol, 1 equiv), and cobalt bis(acetylacetonate) (64.3 mg, 250 µmol, 1.00 equiv). A 16-gauge needle was penetrated through the septum. *n*-Propanol (830 µL), 1,4-dihydrobenzene (238 µL, 2.50 mmol, 10.0 equiv), a solution of *tert*-butyl hydroperoxide in nonane (~5.5 M, 45.5 µL, 250 µmol, 1.00 equiv), and triethylsilane (400 µL, 2.50 mmol, 10.0 equiv) were added sequentially to the reaction vessel via syringe. The reaction mixture was stirred at 24 °C and *tert*-butyl hydroperoxide in nonane (~5.5 M, 22.8 µL, 125 µmol, 0.500 equiv) was added every hour until the consumption of **1a** was complete (as determined by TLC analysis, 180 min for **1a**, 2.50 equiv of TBHP in total). The product mixture was filtered through a short column of silica gel and the short column was rinsed with 20% ethyl acetate–hexanes (100 mL). The filtrates were combined and the combined filtrates were concentrated. ^{1}H NMR analysis of the unpurified product mixture using 1,3,5-trimethoxybenzene as an internal standard (46.8 mg, 278 µmol, 1.11 equiv) revealed a 28% yield of 2-methylallyl 4-methoxybenzoate (**1a**), a 31% yield of isobutyl 4-methoxybenzoate (**2a**), an 82% yield of 1-((allyloxy)methyl)-4-methoxybenzene (**1b**), and a < 1% yield of 1-methoxy-4-(propoxymethyl)benzene (**2b**).

Condition Optimization for the Hydrogenation (Table 1, Entry 4).

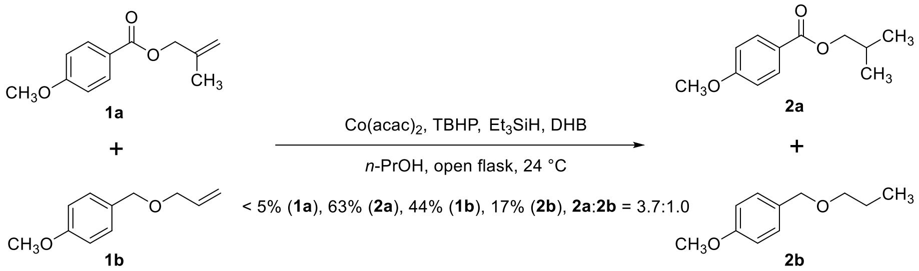

A 25-mL round-bottomed flask fitted with a rubber septum was charged sequentially with 2-methylallyl 4-methoxybenzoate (**1a**, 51.6 mg, 250 µmol, 1 equiv), 1-((allyloxy)methyl)-4-methoxybenzene (**1b**, 44.5 mg, 250 µmol, 1 equiv), and cobalt bis(acetylacetonate) (64.3 mg, 250 µmol, 1.00 equiv). The reaction vessel was open to the atmosphere. *n*-Propanol (830 µL), 1,4-dihydrobenzene (238 µL, 2.50 mmol, 10.0 equiv), a solution of *tert*-butyl hydroperoxide in nonane (~5.5 M, 45.5 µL, 250 µmol, 1.00 equiv), and triethylsilane (400 µL, 2.50 mmol, 10.0 equiv) were added sequentially to the reaction vessel via syringe. The reaction mixture was stirred at 24 °C for 30 min. The product mixture was filtered through a short column of silica gel and the short column was rinsed with 20% ethyl acetate–hexanes (100 mL). The filtrates were combined and the combined filtrates were concentrated. ^{1}H NMR analysis of the unpurified product mixture using mesitylene as an internal standard (32.2 mg, 268 µmol, 1.07 equiv) revealed a < 5% yield of 2-methylallyl 4-methoxybenzoate (**1a**), a 63% yield of isobutyl 4-methoxybenzoate (**2a**), an 44% yield of 1-((allyloxy)methyl)-4-methoxybenzene (**1b**), and a 17% yield of 1-methoxy-4-(propoxymethyl)benzene (**2b**).

Condition Optimization for the Hydrogenation (Table 1, Entry 5).

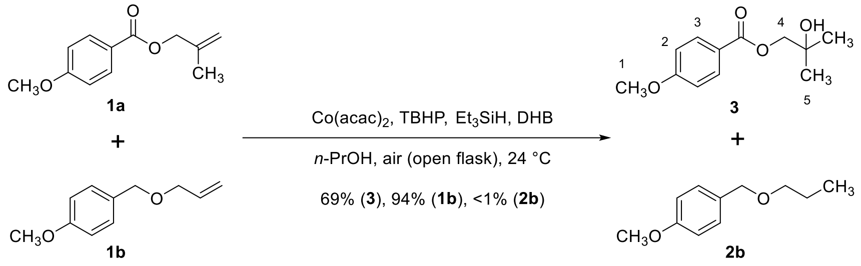

A 25-mL round-bottomed flask fitted with a rubber septum was charged sequentially with 2-methylallyl 4-methoxybenzoate (**1a**, 51.6 mg, 250 μmol, 1 equiv), 1-((allyloxy)methyl)-4-methoxybenzene (**1b**, 44.5 mg, 250 μmol, 1 equiv), and cobalt bis(acetylacetonate) (16.1 mg, 62.5 μmol, 0.250 equiv). The reaction vessel was left open to air. *n*-Propanol (830 μL), 1,4-dihydrobenzene (238 μL, 2.50 mmol, 10.0 equiv), a solution of *tert*-butyl hydroperoxide in nonane (~5.5 M, 11.4 μL, 62.5 μmol, 0.250 equiv), and triethylsilane (400 μL, 2.50 mmol, 10.0 equiv) were added sequentially to the reaction vessel via syringe. The reaction mixture was stirred at 24 °C until the consumption of **1a** was complete (as determined by TLC analysis, 180 min for **1a**). The product mixture was filtered through a short column of silica gel and the short column was rinsed with 20% ethyl acetate–hexanes (100 mL). The filtrates were combined and the combined filtrates were concentrated. ^{1}H NMR analysis of the unpurified product mixture using mesitylene as an internal standard (21.9 mg, 183 μmol, 0.730 equiv) revealed a 94% yield of 1-((allyloxy)methyl)-4-methoxybenzene (**1b**), and a <1% yield of 1-methoxy-4-(propoxymethyl)benzene (**2b**). The NMR sample was concentrated to dryness and the residue obtained was purified by automated flash-column chromatography (eluting with 2% ether–hexanes initially, grading to 20% ether–hexanes, linear gradient) to afford 2-hydroxy-2-methylpropyl 4-methoxybenzoate (**3**, clear oil, 38.6 mg, 69%).

R_f = 0.29 (20% ether–hexanes; UV). ^{1}H NMR (400 MHz, CD_2Cl_2) δ 9.46 (s, 1H, OH), 8.03 (d, J = 8,8 Hz, 2H, H_3), 6.96 (d, J = 9.2 Hz, 2H, H_2), 4.36 (s, 2H, H_4), 3.87 (s, 3H, H_1), 1.27 (s, 6H, H_5). ^{13}C NMR (100 MHz, CD_2Cl_2) δ 168.4 (C), 164.5 (C), 132.5 (CH), 122.4 (C), 114.3 (CH), 82.1 (C), 67.0 (CH_2), 56.1 (CH_3), 21.7 (CH_3). IR (ATR-FTIR), cm^{-1}: 3342 (w), 2983 (m), 1688 (m), 1255 (m), 1167 (s). HRMS-ESI (m/z): $[M + Na]^+$ calcd for $C_{12}H_{16}NaO_4$, 247.0946; found, 247.0942.

Condition Optimization for the Hydrogenation (Table 1, Entry 6).

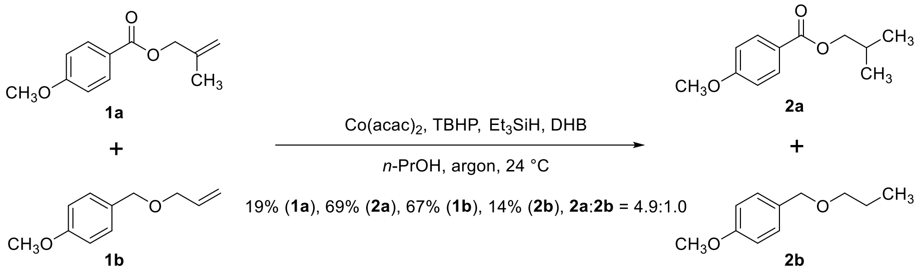

A 25-mL round-bottomed flask fitted with a rubber septum was charged sequentially with 2-methylallyl 4-methoxybenzoate (**1a**, 51.6 mg, 250 μmol, 1 equiv), 1-((allyloxy)methyl)-4-methoxybenzene (**1b**, 44.5 mg, 250 μmol, 1 equiv), and cobalt bis(acetylacetonate) (64.3 mg, 250 μmol, 1.00 equiv). The reaction vessel was evacuated and refilled using a balloon of argon. This process was repeated twice. *n*-Propanol (830 μL), 1,4-dihydrobenzene (238 μL, 2.50 mmol, 10.0 equiv), a solution of *tert*-butyl hydroperoxide in nonane (~5.5 M, 45.5 μL, 250 μmol, 1.00 equiv), and triethylsilane (400 μL, 2.50 mmol, 10.0 equiv) were added sequentially to the reaction vessel via syringe. The reaction mixture was stirred at 24 °C and *tert*-butyl hydroperoxide in nonane (~5.5 M, 22.8 μL, 125 μmol, 0.500 equiv) was added every hour until the consumption of **1a** was complete (as determined by TLC analysis, 360 min for **1a**, 3.50 equiv of TBHP in total). The product mixture was filtered through a short column of silica gel and the short column was rinsed with 20% ethyl acetate–hexanes (100 mL). The filtrates were combined and the combined filtrates were concentrated. ^{1}H NMR analysis of the unpurified product mixture using 1,3,5-trimethoxybenzene as an internal standard (41.4 mg, 246 μmol, 0.948 equiv) revealed a 19% yield of 2-methylallyl 4-methoxybenzoate (**1a**), a 69% yield of isobutyl 4-methoxybenzoate (**2a**), an 67% yield of 1-((allyloxy)methyl)-4-methoxybenzene (**1b**), and a 14% yield of 1-methoxy-4-(propoxymethyl)benzene (**2b**).

Condition Optimization for the Hydrogenation (Table 1, Entry 7).

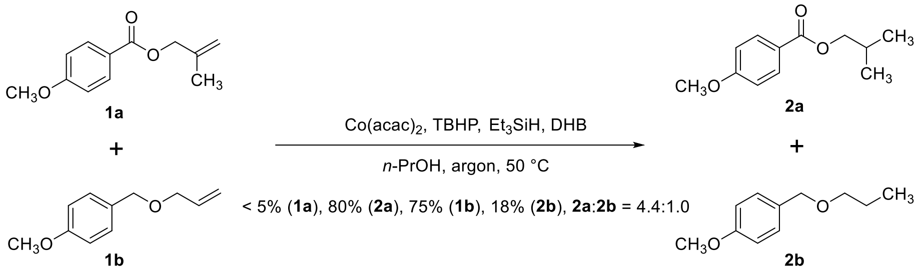

A 25-mL round-bottomed flask fitted with a rubber septum was charged sequentially with 2-methylallyl 4-methoxybenzoate (**1a**, 51.6 mg, 250 µmol, 1 equiv), 1-((allyloxy)methyl)-4-methoxybenzene (**1b**, 44.5 mg, 250 µmol, 1 equiv), and cobalt bis(acetylacetonate) (64.3 mg, 250 µmol, 1.00 equiv). The reaction vessel was evacuated and refilled using a balloon of argon. This process was repeated twice. *n*-Propanol (830 µL), 1,4-dihydrobenzene (238 µL, 2.50 mmol, 10.0 equiv), a solution of *tert*-butyl hydroperoxide in nonane (~5.5 M, 45.5 µL, 250 µmol, 1.00 equiv), and triethylsilane (400 µL, 2.50 mmol, 10.0 equiv) were added sequentially to the reaction vessel via syringe. The reaction vessel was placed in an oil bath that had been preheated to 50 °C. The reaction mixture was stirred at 50 °C and *tert*-butyl hydroperoxide in nonane (~5.5 M, 22.8 µL, 125 µmol, 0.500 equiv) was added every hour until the consumption of **1a** was complete (as determined by TLC analysis, 120 min for **1a**, 1.50 equiv of TBHP in total). The product mixture was filtered through a short column of silica gel and the short column was rinsed with 20% ethyl acetate–hexanes (100 mL). The filtrates were combined and the combined filtrates were concentrated. ^{1}H NMR analysis of the unpurified product mixture using mesitylene as an internal standard (29.3 mg, 244 µmol, 0.977 equiv) revealed a < 5% yield of 2-methylallyl 4-methoxybenzoate (**1a**), a 80% yield of isobutyl 4-methoxybenzoate (**2a**), an 75% yield of 1-((allyloxy)methyl)-4-methoxybenzene (**1b**), and a 18% yield of 1-methoxy-4-(propoxymethyl)benzene (**2b**).

Condition Optimization for the Hydrogenation (Table 1, Entry 8).

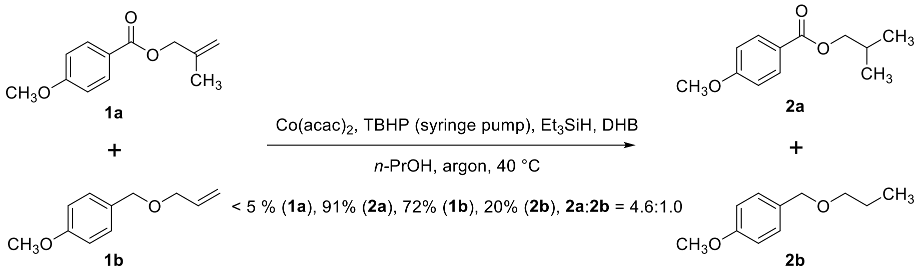

A 10-mL two-neck round-bottomed flask fitted with a rubber septum and a reflux condenser was charged sequentially with 2-methylallyl 4-methoxybenzoate (**1a**, 51.6 mg, 250 µmol, 1 equiv), 1-((allyloxy)methyl)-4-methoxybenzene (**1b**, 44.5 mg, 250 µmol, 1 equiv), and cobalt bis(acetylacetonate) (64.3 mg, 250 µmol, 1.00 equiv). The reaction vessel was evacuated and refilled using a balloon of argon. This process was repeated twice. *n*-Propanol (830 µL), 1,4-dihydrobenzene (238 µL, 2.5 mmol, 10.0 equiv), and triethylsilane (400 µL, 2.50 mmol, 10.0 equiv) were added sequentially to the reaction vessel via syringe. The reaction vessel was placed in an oil bath that had been preheated to 40 °C. A solution of *tert*-butyl hydroperoxide in nonane (~5.5 M, 45.5 µL, 250 µmol, 1.00 equiv) was added dropwise over 60 min via syringe pump to the reaction vessel. The reaction mixture was stirred and heated at 40 °C until the consumption of **1a** was complete (as determined by TLC analysis, 60 min for **1a**). The product mixture was filtered through a short column of silica gel and the short column was rinsed with 20% ethyl acetate–hexanes (100 mL). The filtrates were combined and the combined filtrates were concentrated. ^{1}H NMR analysis of the unpurified product mixture using mesitylene as an internal standard (28.7 mg, 239 µmol, 0.956 equiv) revealed a < 5% yield of 2-methylallyl 4-methoxybenzoate (**1a**), a 91% yield of isobutyl 4-methoxybenzoate (**2a**), a 72% yield of 1-((allyloxy)methyl)-4-methoxybenzene (**1b**), and a 20% yield of 1-methoxy-4-(propoxymethyl)benzene (**2b**).

Condition Optimization for the Hydrogenation (Table S1, Entry 1).

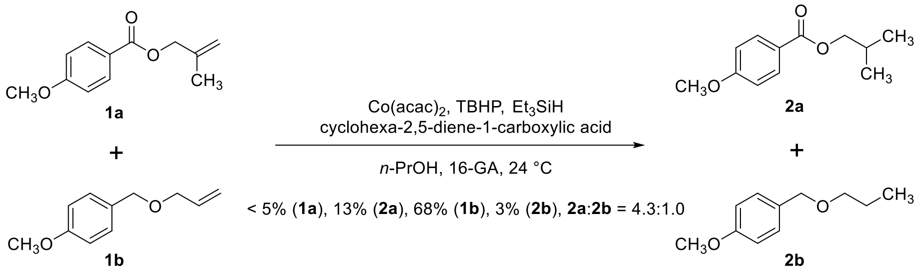

A 25-mL round-bottomed flask fitted with a rubber septum was charged sequentially with 2-methylallyl 4-methoxybenzoate (**1a**, 51.6 mg, 250 µmol, 1 equiv), 1-((allyloxy)methyl)-4-methoxybenzene (**1b**, 44.5 mg, 250 µmol, 1 equiv), and cobalt bis(acetylacetonate) (64.3 mg, 250 µmol, 1.00 equiv). A 16-gauge needle was penetrated through the septum. *n*-Propanol (830 µL), cyclohexa-2,5-diene-1-carboxylic acid (310 mg, 2.50 mmol, 10.0 equiv), a solution of *tert*-butyl hydroperoxide in nonane (~5.5 M, 45.5 µL, 250 µmol, 1.00 equiv), and triethylsilane (400 µL, 2.50 mmol, 10.0 equiv) were added sequentially to the reaction vessel via syringe. The reaction mixture was stirred at 24 °C and *tert*-butyl hydroperoxide in nonane (~5.5 M, 22.8 µL, 125 µmol, 0.500 equiv) was added every hour until the consumption of **1a** was complete (as determined by TLC analysis, 280 min for **1a**, 3.50 equiv of TBHP in total). The product mixture was filtered through a short column of silica gel and the short column was rinsed with 20% ethyl acetate–hexanes (100 mL). The filtrates were combined and the combined filtrates were concentrated. ^{1}H NMR analysis of the unpurified product mixture using mesitylene as an internal standard (61.9 mg, 516 µmol, 2.06 equiv) revealed a < 5% yield of 2-methylallyl 4-methoxybenzoate (**1a**), a 13% yield of isobutyl 4-methoxybenzoate (**2a**), an 68% yield of 1-((allyloxy)methyl)-4-methoxybenzene (**1b**), and a 3% yield of 1-methoxy-4-(propoxymethyl)benzene (**2b**). The NMR sample was concentrated to dryness and the residue obtained was purified by automated flash-column chromatography (eluting with 2% ether–hexanes initially, grading to 20% ether–hexanes, linear gradient) to afford 2-hydroxy-2-methylpropyl 4-methoxybenzoate (**3**, clear oil, 33.6 mg, 60%).

Condition Optimization for the Hydrogenation (Table S1, Entry 2).

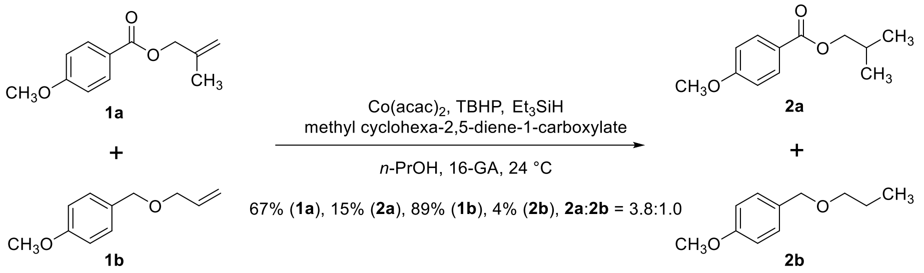

A 25-mL round-bottomed flask fitted with a rubber septum was charged sequentially with 2-methylallyl 4-methoxybenzoate (**1a**, 51.6 mg, 250 µmol, 1 equiv), 1-((allyloxy)methyl)-4-methoxybenzene (**1b**, 44.5 mg, 250 µmol, 1 equiv), and cobalt bis(acetylacetonate) (64.3 mg, 250 µmol, 1.00 equiv). A 16-gauge needle was penetrated through the septum. *n*-Propanol (830 µL), methyl cyclohexa-2,5-diene-1-carboxylate (345 mg, 2.50 mmol, 10.0 equiv), a solution of *tert*-butyl hydroperoxide in nonane (~5.5 M, 45.5 µL, 250 µmol, 1.00 equiv), and triethylsilane (400 µL, 2.50 mmol, 10.0 equiv) were added sequentially to the reaction vessel via syringe. The reaction mixture was stirred at 24 °C 40 min. The product mixture was filtered through a short column of silica gel and the short column was rinsed with 20% ethyl acetate–hexanes (100 mL). The filtrates were combined and the combined filtrates were concentrated. ^{1}H NMR analysis of the unpurified product mixture using mesitylene as an internal standard (29.9 mg, 249 µmol, 1.06 equiv) revealed a 67% yield of 2-methylallyl 4-methoxybenzoate (**1a**), a 15% yield of isobutyl 4-methoxybenzoate (**2a**), an 89% yield of 1-((allyloxy)methyl)-4-methoxybenzene (**1b**), and a 4% yield of 1-methoxy-4-(propoxymethyl)benzene (**2b**). The NMR sample was concentrated to dryness and the residue obtained was purified by automated flash-column chromatography (eluting with 2% ether–hexanes initially, grading to 20% ether–hexanes, linear gradient) to afford 2-hydroxy-2-methylpropyl 4-methoxybenzoate (**3**, clear oil, 10.0 mg, 18%).

Condition Optimization for the Hydrogenation (Table S1, Entry 3).

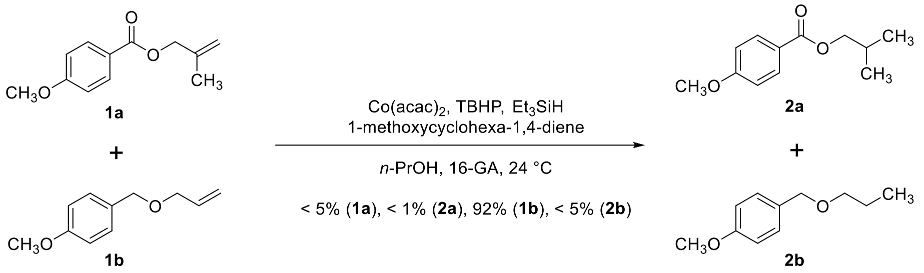

A 25-mL round-bottomed flask fitted with a rubber septum was charged sequentially with 2-methylallyl 4-methoxybenzoate (**1a**, 51.6 mg, 250 µmol, 1 equiv), 1-((allyloxy)methyl)-4-methoxybenzene (**1b**, 44.5 mg, 250 µmol, 1 equiv), and cobalt bis(acetylacetonate) (64.3 mg, 250 µmol, 1.00 equiv). A 16-gauge needle was penetrated through the septum. *n*-Propanol (830 µL), 1-methoxycyclohexa-1,4-diene (275 mg, 2.50 mmol, 10.0 equiv), a solution of *tert*-butyl hydroperoxide in nonane (~5.5 M, 45.5 µL, 250 µmol, 1.00 equiv), and triethylsilane (400 µL, 2.50 mmol, 10.0 equiv) were added sequentially to the reaction vessel via syringe. The reaction mixture was stirred at 24 °C and *tert*-butyl hydroperoxide in nonane (~5.5 M, 22.8 µL, 125 µmol, 0.500 equiv) was added every hour until the consumption of **1a** was complete (as determined by TLC analysis, 380 min for **1a**, 4.00 equiv of TBHP in total). The product mixture was filtered through a short column of silica gel and the short column was rinsed with 20% ethyl acetate–hexanes (100 mL). The filtrates were combined and the combined filtrates were concentrated. ^1H NMR analysis of the unpurified product mixture using mesitylene as an internal standard (29.1 mg, 243 µmol, 0.970 equiv) revealed a < 5% yield of 2-methylallyl 4-methoxybenzoate (**1a**), a < 1% yield of isobutyl 4-methoxybenzoate (**2a**), a 92% yield of 1-((allyloxy)methyl)-4-methoxybenzene (**1b**), and a < 5% yield of 1-methoxy-4-(propoxymethyl)benzene (**2b**).

Representative Procedure for Competition Experiments (Table S2, Condition A).

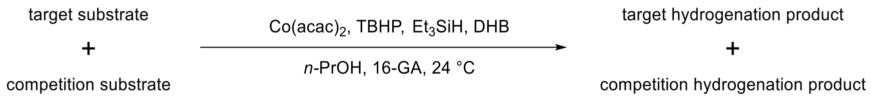

A 25-mL round-bottomed flask fitted with a rubber septum was charged sequentially with the target substrate (250 µmol, 1 equiv), the competition substrate (250 µmol, 1 equiv), and cobalt bis(acetylacetonate) (64.3 mg, 250 µmol, 1.00 equiv). A 16-gauge needle was penetrated through the septum. *n*-Propanol (830 µL), 1,4-dihydrobenzene (238 µL, 2.50 mmol, 10.0 equiv), a solution of *tert*-butyl hydroperoxide in nonane (~5.5 M, 45.5 µL, 250 µmol, 1.00 equiv), and triethylsilane (400 µL, 2.50 mmol, 10.0 equiv) were added sequentially to the reaction vessel via syringe. The reaction mixture was stirred at 24 °C and a solution of *tert*-butyl hydroperoxide in nonane (~5.5 M, 22.8 µL, 125 µmol, 0.50 equiv) was added every hour until the consumption of the target substrate was complete (as determined by TLC analysis). The product mixture was filtered through a short column of silica gel and the short column was rinsed with 20% ethyl acetate–hexanes (100 mL). The filtrates were combined and the combined filtrates were concentrated. The conversion of the competition substrate and the yield of the competition product were determined by ^1H NMR analysis of the unpurified product mixture using mesitylene as an internal standard. The NMR sample was concentrated to dryness and the residue obtained was purified by automated flash-column chromatography to afford the target hydrogenation product.

Representative Procedure for Competition Experiments (Table S2, Condition B).

target substrate + competition substrate $\xrightarrow[n\text{-PrOH, argon, 40 °C}]{\text{Co(acac)}_2\text{, TBHP (syringe pump), Et}_3\text{SiH, DHB}}$ target hydrogenation product + competition hydrogenation product

A 10-mL round-bottomed flask fitted with a reflux condenser was charged sequentially with the target substrate (250 μmol, 1 equiv), the competition substrate (250 μmol, 1 equiv), and cobalt bis(acetylacetonate) (64.3 mg, 250 μmol, 1.00 equiv). The reaction vessel was evacuated and refilled using a balloon of argon. This process was repeated twice. *n*-Propanol (830 μL), 1,4-dihydrobenzene (238 μL, 2.5 mmol, 10.0 equiv), and triethylsilane (400 μL, 2.50 mmol, 10.0 equiv) were added sequentially to the reaction vessel via syringe. The reaction vessel was placed in an oil bath that had been preheated to 40 °C. A solution of *tert*-butyl hydroperoxide in nonane (~5.5 M, 45.5 μL, 250 μmol, 1.00 equiv) was added dropwise via syringe pump to the reaction vessel. The reaction mixture was stirred and heated at 40 °C until the consumption of the target substrate was complete (as determined by TLC analysis). The product mixture was filtered through a short column of silica gel and the short column was rinsed with 20% ethyl acetate–hexanes (100 mL). The filtrates were combined and the combined filtrates were concentrated. The conversion of the competition substrate and the yield of the competition product were determined by ^{1}H NMR analysis of the unpurified product mixture using mesitylene as an internal standard. The NMR sample was concentrated to dryness and the residue obtained was purified by automated flash-column chromatography to afford the target hydrogenation product.

2-Methylallyl 4-methoxybenzoate (1a) versus allyl 4-methoxybenzoate (1c)

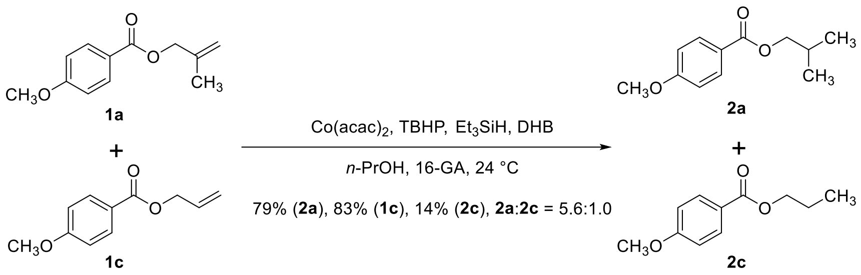

Condition A

Following the general procedure A using 2-methylallyl 4-methoxybenzoate (**1a**, 51.6 mg, 250 μmol, 1 equiv) and allyl 4-methoxybenzoate (**1c**, 48.1 mg, 250 μmol, 1 equiv). Reaction time was 135 min and 2.00 equiv of TBHP were employed. ^{1}H NMR analysis of the unpurified product mixture using mesitylene as an internal standard (24.1 mg, 201 μmol, 0.803 equiv) revealed a 79% yield of isobutyl 4-methoxybenzoate, an 83% yield of allyl 4-methoxybenzoate (**1c**) and a 14% yield of propyl 4-methoxybenzoate (**2c**).

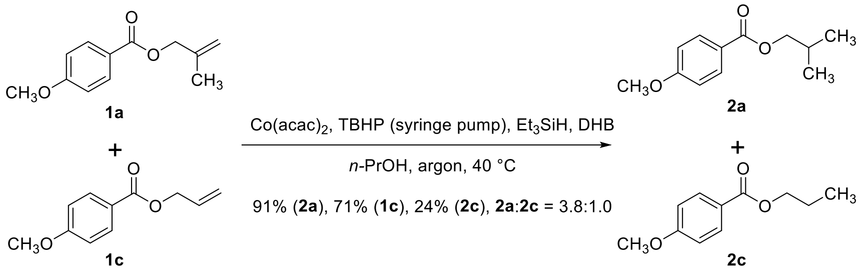

Condition B:

Following the general procedure B using 2-methylallyl 4-methoxybenzoate (**1a**, 51.6 mg, 250 μmol, 1 equiv) and allyl 4-methoxybenzoate (**1c**, 48.1 mg, 250 μmol, 1 equiv). Reaction time was 60 min and 1.00 equiv of TBHP was added in total. ^{1}H NMR analysis of the unpurified product mixture with mesitylene as an internal standard (28.7 mg, 239 μmol, 0.957 equiv) revealed a 91% yield of isobutyl 4-methoxybenzoate (**2a**), a 71% yield of allyl 4-methoxybenzoate (**1c**) and a 24% yield of propyl 4-methoxybenzoate (**2c**).

2-Methylallyl 4-methoxybenzoate (1a) versus (allyloxy)(tert-butyl)diphenylsilane (4a)

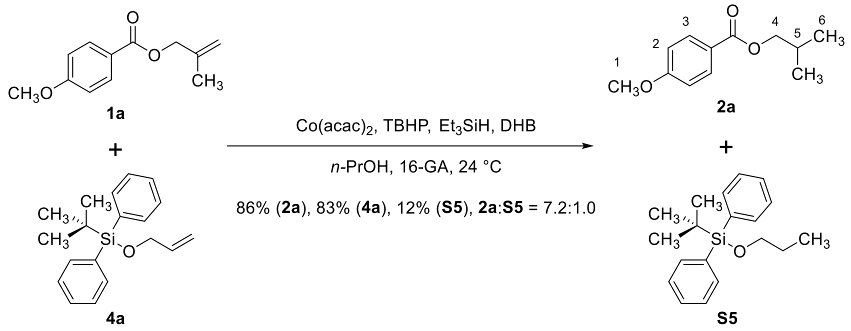

Condition A

Following the general procedure A using 2-methylallyl 4-methoxybenzoate (**1a**, 51.6 mg, 250 μmol, 1 equiv) and (allyloxy)(*tert*-butyl)diphenylsilane (**4a**, 74.0 mg, 250 μmol, 1 equiv). Reaction time was 135 min and 2.00 equiv of TBHP were employed. ^{1}H NMR analysis of the unpurified product mixture using mesitylene as an internal standard (24.7 mg, 206 μmol, 0.823 equiv) revealed an 83% yield of (allyloxy)(*tert*-butyl)diphenylsilane (**4a**) and a 12% yield of *tert*-butyldiphenyl(propoxy)silane (**S5**). The NMR sample was concentrated to dryness and the residue obtained was purified by automated flash-column chromatography (eluting with 2% ether–hexanes initially, grading to 5% ether–hexanes, linear gradient) to afford isobutyl 4-methoxybenzoate (**2a**, clear oil, 44.6 mg, 86%).

Isobutyl 4-methoxybenzoate (**2a**): R_f = 0.54 (20% ethyl acetate–hexanes; UV). ^{1}H NMR (400 MHz, $CDCl_3$) δ 8.01 (d, 2H, J = 8.8 Hz, H_3), 6.92 (d, 2H, J = 8.8 Hz, H_2), 4.07 (d, J = 6.8 Hz, 2H, H_4), 3.86 (s 3H, H_1), 2.12–2.02 (m, H, H_5), 1.02 (d, J = 6.8 Hz, 6H, H_6). ^{13}C NMR (100 MHz, $CDCl_3$) δ 166.4 (C), 163.2 (C), 131.5 (CH), 123.0 (C), 113.5 (CH), 70.7 (CH_2), 55.4 (CH_3), 27.9 (CH), 19.2 (CH_3).

^{1}H and ^{13}C NMR data for isobutyl 4-methoxybenzoate (**2a**) prepared in this way were in agreement with those previously described.[19]

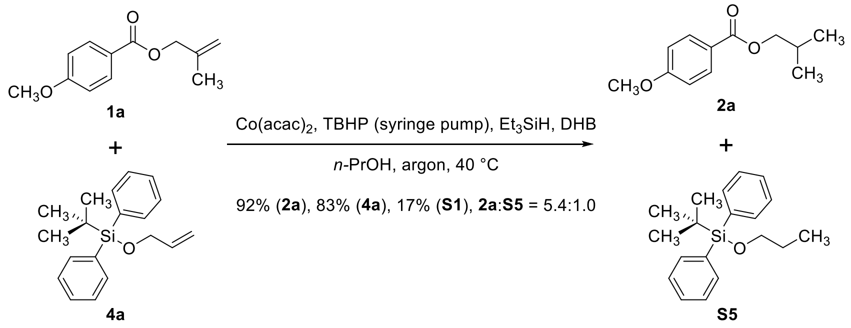

Condition B:

Following the general procedure B using 2-methylallyl 4-methoxybenzoate (**1a**, 51.6 mg, 250 μmol, 1 equiv) and (allyloxy)(*tert*-butyl)diphenylsilane (**4a**, 74.0 mg, 250 μmol, 1 equiv). Reaction time was 60 min and 1.00 equiv of TBHP was added in total. ^{1}H NMR analysis of the unpurified product mixture with mesitylene as an internal standard (30.8 mg, 257 μmol, 1.03 equiv) revealed a 83% yield of (allyloxy)(*tert*-butyl)diphenylsilane (**4a**) and a 17% yield of *tert*-butyldiphenyl(propoxy)silane (**S5**). The NMR sample was concentrated to dryness and the residue obtained was purified by automated flash-column chromatography (eluting with 2% ether–hexanes initially, grading to 5% ether–hexanes, linear gradient) to afford isobutyl 4-methoxybenzoate (**2a**, clear oil, 47.8 mg, 92%).

2-Chloroallyl 4-methoxybenzoate (1d) versus (allyloxy)(tert-butyl)diphenylsilane (4a)

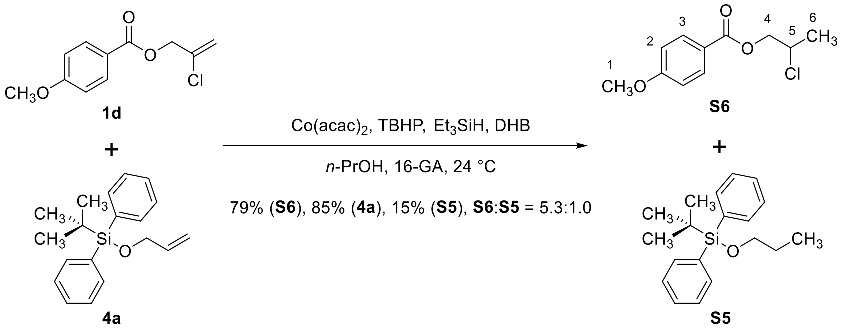

Condition A:

Following the general procedure A using 2-chloroallyl 4-methoxybenzoate (**1d**, 56.7 mg, 250 μmol, 1 equiv) and (allyloxy)(*tert*-butyl)diphenylsilane (**4a**, 74.0 mg, 250 μmol, 1 equiv). Reaction time was 40 min and 1.00 equiv of TBHP were employed. ^{1}H NMR analysis of the unpurified product mixture using mesitylene as an internal standard (28.5 mg, 238 μmol, 0.950 equiv) revealed an 85% yield of (allyloxy)(*tert*-butyl)diphenylsilane (**4a**) and a 15% yield of *tert*-butyldiphenyl(propoxy)silane (**S5**). Purification by automated flash-column chromatography (eluting with 2% ether–hexanes initially, grading to 5% ether–hexanes, linear gradient) afforded 2-chloropropyl 4-methoxybenzoate (**S6**) as a clear oil (45.3 mg, 79%).

R_f = 0.52 (20% ethyl acetate–hexanes; UV). ^{1}H NMR (500 MHz, $CDCl_3$) δ 8.02 (d, 2H, J = 9.0 Hz, H_3), 6.93 (d, 2H, J = 9.0 Hz, H_2), 4.42–4.38 (m, 2H, H_4), 4.34–4.27 (m, 1H, H_5), 3.87 (s, 3H, H_1), 1.60 (d, 3H, J = 6.5 Hz, H_6). ^{13}C NMR (150 MHz, $CDCl_3$) δ 165.6 (C), 163.4 (C), 131.7 (CH), 121.9 (C), 113.6 (CH), 68.6 (CH_2), 55.3 (CH_3), 54.1 (CH), 21.5 (CH_3).

^{1}H and ^{13}C NMR data for 2-chloroallyl 4-methoxybenzoate (**S6**) prepared in this way were in agreement with those previously described.[14]

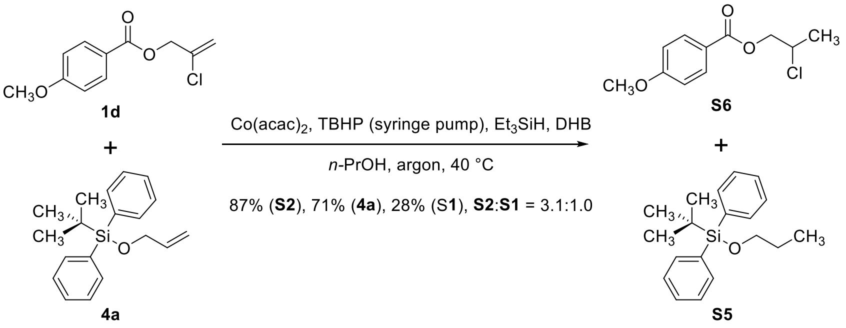

Condition B:

Following the general procedure B using 2-chloroallyl 4-methoxybenzoate (**1d**, 56.7 mg, 250 μmol, 1 equiv) and (allyloxy)(*tert*-butyl)diphenylsilane (**4a**, 74.0 mg, 250 μmol, 1 equiv). Reaction time was 60 min and 1.00 equiv of TBHP was added in total. ^{1}H NMR analysis of the unpurified product mixture with mesitylene as an internal standard (28.7 mg, 239 μmol, 0.957 equiv) revealed a 71% yield of (allyloxy)(*tert*-butyl)diphenylsilane (**4a**) and a 28% yield of *tert*-butyldiphenyl(propoxy)silane (**S5**). Purification by automated flash-column chromatography (eluting with 2% ether–hexanes initially, grading to 5% ether–hexanes, linear gradient) afforded 2-chloropropyl 4-methoxybenzoate (**S6**) as a clear oil (49.8 mg, 87%)

3-Bromobut-3-en-1-yl (4-methoxyphenyl)carbamate (1e) versus (allyloxy)(tert-butyl)diphenylsilane (4a)

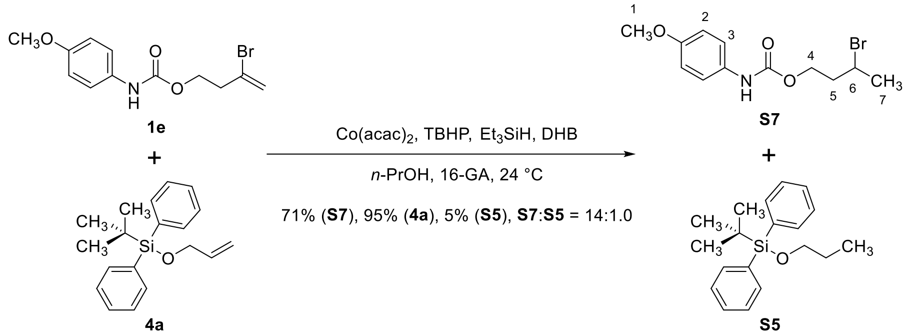

Condition A:

Following the general procedure A using 3-bromobut-3-en-1-yl (4-methoxyphenyl)carbamate (**1e**, 75.0 mg, 250 μmol, 1 equiv) and (allyloxy)(*tert*-butyl)diphenylsilane (**4a**, 74.0 mg, 250 μmol, 1 equiv). Reaction time was 20 min and 1.00 equiv of TBHP were employed. ^{1}H NMR analysis of the unpurified product mixture using mesitylene as an internal standard (33.2 mg, 277 μmol, 1.11 equiv) revealed a 95% yield of (allyloxy)(*tert*-butyl)diphenylsilane (**4a**). Purification by automated flash-column chromatography (eluting with 2% ethyl acetate–hexanes initially, grading to 10% ethyl acetate–hexanes, linear gradient) afforded 3-bromobutyl (4-methoxyphenyl)carbamate (**S7**) as a white solid (53.6 mg, 71%).

R_f = 0.34 (33% ether–hexanes; UV, CAM). ^{1}H NMR (400 MHz, $CDCl_3$) δ 7.33–7.22 (m, 2H, H_3), 6.83 (d, 2H, J = 8.8 Hz, H_2), 6.74 (br s, 1H, NH), 4.40–4.31 (m, 1H, H_4), 4.30–4.15 (m, 2H, H_6, 1 × H_4), 3.77 (s, 3H, H_1), 2.19–2.05 (m, 2H, H_5), 1.74 (d, 3H, J = 6.8 Hz, H_7). ^{13}C NMR (100 MHz, $CDCl_3$) δ 155.8 (C), 153.6 (C), 130.7 (C), 120.5 (CH), 114.1 (CH), 63.1 (CH_2), 55.4 (CH_3), 47.0 (CH), 39.9 (CH_2), 26.4 (CH_3).

^{1}H and ^{13}C NMR data for 3-bromobutyl (4-methoxyphenyl)carbamate (**S7**) prepared in this way were in agreement with those previously described.[14]

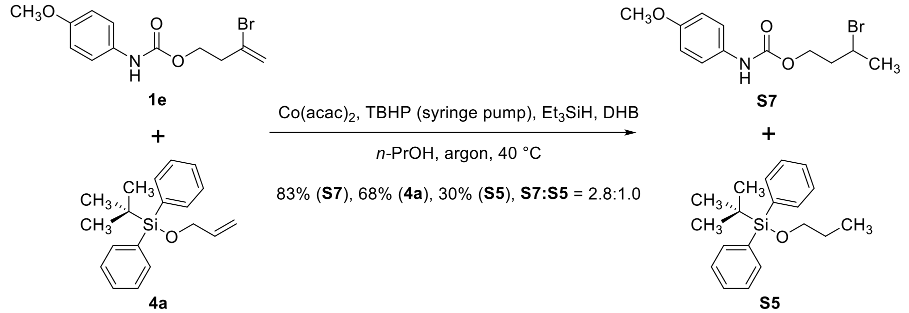

Condition B:

Following the general procedure B using 2-chloroallyl 4-methoxybenzoate (**1e**, 75.0 mg, 250 μmol, 1 equiv) and (allyloxy)(*tert*-butyl)diphenylsilane (**4a**, 74.0 mg, 250 μmol, 1 equiv). Reaction time was 60 min and 1.00 equiv of TBHP was added in total. ^{1}H NMR analysis of the unpurified product mixture with mesitylene as an internal standard (29.1 mg, 243 μmol, 0.971 equiv) revealed a 62% yield of (allyloxy)(*tert*-butyl)diphenylsilane (**4a**) and a 30% yield of *tert*-butyldiphenyl(propoxy)silane (**S5**). Purification by automated flash-column chromatography (eluting with 2% ether–hexanes initially, grading to 10% ether–hexanes, linear gradient) afforded 3-bromobutyl (4-methoxyphenyl)carbamate (**S7**) as a white solid (62.8 mg, 83%).

Benzyl 4-methylenepiperidine-1-carboxylate (1f) versus (allyloxy)(tert-butyl)diphenylsilane (4a)

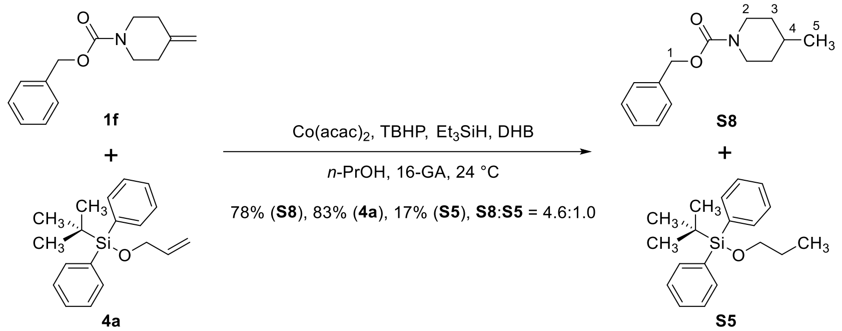

Condition A:

Following the general procedure A using benzyl 4-methylenepiperidine-1-carboxylate (**1f**, 57.8 mg, 250 μmol, 1 equiv) and (allyloxy)(*tert*-butyl)diphenylsilane (**4a**, 74.0 mg, 250 μmol, 1 equiv). Reaction time was 120 min and 1.50 equiv of TBHP were employed. ^{1}H NMR analysis of the unpurified product mixture using mesitylene as an internal standard (30.3 mg, 253 μmol, 1.01 equiv) revealed an 83% yield of (allyloxy)(*tert*-butyl)diphenylsilane (**4a**) and a 17% yield of *tert*-butyldiphenyl(propoxy)silane (**S5**). Purification by automated flash-column chromatography (eluting with 2% ethyl acetate–hexanes initially, grading to 10% ethyl acetate–hexanes, linear gradient) afforded benzyl 4-methylpiperidine-1-carboxylate (**S8**) as a clear oil (45.4 mg, 78%).

R_f = 0.29 (20% ethyl acetate–hexanes; UV, CAM). ^{1}H NMR (400 MHz, CD_2Cl_2) δ 7.37–7.29 (m, 5H, ArH), 5.11 (s, 2H, H_1), 4.12 (br d, J = 12.4 Hz, 2H, H_2), 2.77 (br s, 2H, H_2), 1.63 (br d, J = 12.4 Hz, 2H, H_3), 1.57–1.52 (m, 1H, H_4), 1.10 (qd, J = 12.4, 4.0 Hz, 2H, H_3), 0.95 (d, 3H, J = 6.8 Hz, H_5). ^{13}C NMR (100 MHz, CD_2Cl_2) δ 155.6 (C), 138.0 (C), 128.9 (CH), 128.3 (CH), 128.2 (CH), 67.2 (CH_2), 44.8 (CH_2), 34.5 (CH_2), 31.5 (CH), 22.2 (CH_3).

^{1}H and ^{13}C NMR data for benzyl 4-methylpiperidine-1-carboxylate (**S8**) prepared in this way were in agreement with those previously described.[20]

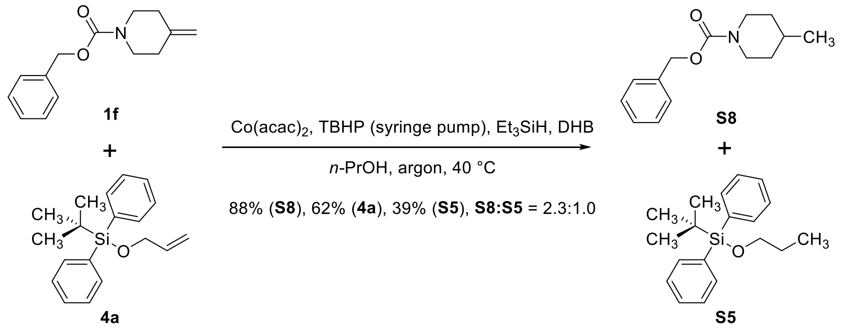

Condition B:

Following the general procedure B using benzyl 4-methylenepiperidine-1-carboxylate (**1f**, 57.8 mg, 250 μmol, 1 equiv) and (allyloxy)(*tert*-butyl)diphenylsilane (**4a**, 74.0 mg, 250 μmol, 1 equiv). Reaction time was 90 min and 1.52 equiv of TBHP was added in total. ^{1}H NMR analysis of the unpurified product mixture with mesitylene as an internal standard (37.9 mg, 316 μmol, 1.26 equiv) revealed a 62% yield of (allyloxy)(*tert*-butyl)diphenylsilane (**4a**) and a 39% yield of *tert*-butyldiphenyl(propoxy)silane (**S5**). Purification by automated flash-column chromatography (eluting with 2% ethyl acetate–hexanes initially,

grading to 10% ethyl acetate–hexanes, linear gradient) afforded benzyl 4-methylpiperidine-1-carboxylate (**S8**) as a white solid (51.3 mg, 88%).

2-Methallyl 4-methoxybenzoate (1a) versus (E)-tert-butyl(pent-2-en-1-yloxy)diphenylsilane (4b)

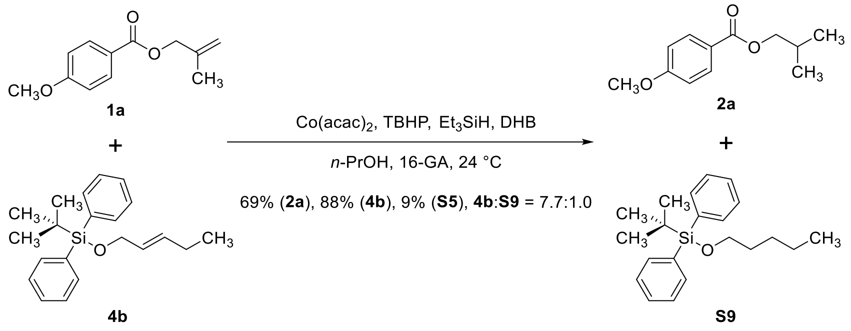

Condition A:
Following the general procedure A using 2-methylallyl 4-methoxybenzoate (**1a**, 51.6 mg, 250 μmol, 1 equiv) and (*E*)-*tert*-butyl(pent-2-en-1-yloxy)diphenylsilane (**4b**, 81.1 mg, 250 μmol, 1 equiv). Reaction time was 135 min and 2.00 equiv of TBHP was added in total. ^{1}H NMR analysis of the unpurified product mixture with mesitylene as an internal standard (27.7 mg, 231 μmol, 0.923 equiv) revealed a 88% yield of (*E*)-*tert*-butyl(pent-2-en-1-yloxy)diphenylsilane (**4b**) and a 9% yield of *tert*-butyl(pentyloxy)diphenylsilane (**S9**). Purification by automated flash-column chromatography (eluting with 2% ether–hexanes initially, grading to 10% ether–hexanes, linear gradient) afforded isobutyl 4-methoxybenzoate (**2a**) as a clear oil (35.8 mg, 69%).

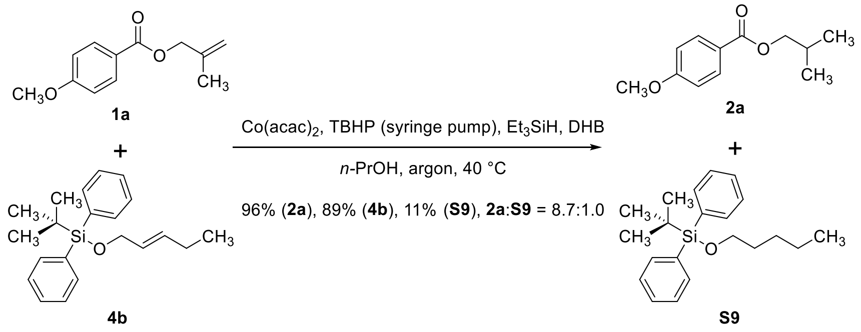

Condition B:
Following the general procedure B using 2-methylallyl 4-methoxybenzoate (**1a**, 51.6 mg, 250 μmol, 1 equiv) and (*E*)-*tert*-butyl(pent-2-en-1-yloxy)diphenylsilane (**4b**, 81.1 mg, 250 μmol, 1 equiv). Reaction time was 60 min and 1.00 equiv of TBHP were employed. ^{1}H NMR analysis of the unpurified product mixture using mesitylene as an internal standard (27.7 mg, 231 μmol, 0.923 equiv) revealed an 89% yield of (*E*)-*tert*-butyl(pent-2-en-1-yloxy)diphenylsilane (**4b**) and an 11% yield of *tert*-butyl(pentyloxy)diphenylsilane (**S9**). Purification by automated flash-column chromatography (eluting with 2% ethyl acetate–hexanes initially, grading to 10% ethyl acetate–hexanes, linear gradient) afforded isobutyl 4-methoxybenzoate (**2a**) as a clear oil (50.2 mg, 96%).

2-Methallyl 4-methoxybenzoate (1a) versus (Z)-tert-butyl(pent-2-en-1-yloxy)diphenylsilane (4c)

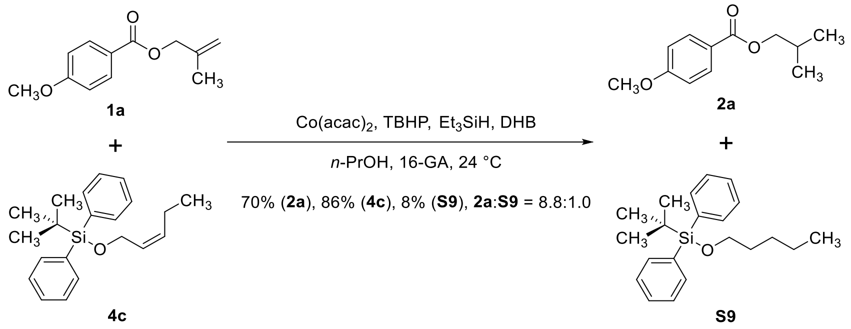

Condition A:

Following the general procedure A using 2-methylallyl 4-methoxybenzoate (**1a**, 51.6 mg, 250 µmol, 1 equiv) and (*Z*)-*tert*-butyl(pent-2-en-1-yloxy)diphenylsilane (**4c**, 81.1 mg, 250 µmol, 1 equiv). Reaction time was 135 min and 2.00 equiv of TBHP were employed. ^{1}H NMR analysis of the unpurified product mixture using mesitylene as an internal standard (27.7 mg, 231 µmol, 0.923 equiv) revealed an 86% yield of (*Z*)-*tert*-butyl(pent-2-en-1-yloxy)diphenylsilane (**4c**) and an 8% yield of *tert*-butyl(pentyloxy)diphenylsilane (**S9**). Purification by automated flash-column chromatography (eluting with 2% ether–hexanes initially, grading to 10% ether–hexanes, linear gradient) afforded isobutyl 4-methoxybenzoate (**2a**) as a clear oil (36.5 mg, 70%).

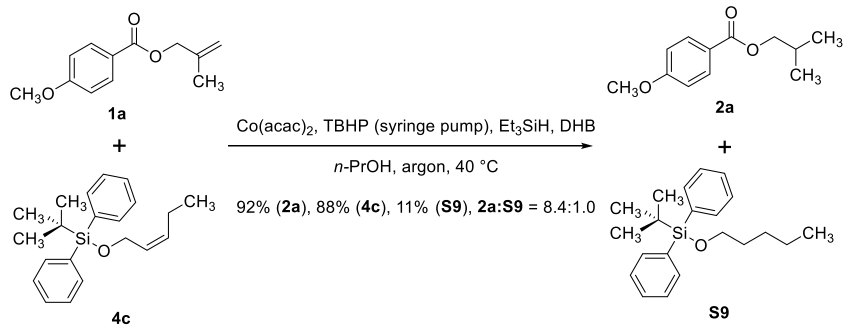

Condition B:

Following the general procedure B using 2-methylallyl 4-methoxybenzoate (**1a**, 51.6 mg, 250 µmol, 1 equiv) and (*Z*)-*tert*-butyl(pent-2-en-1-yloxy)diphenylsilane (**4c**, 81.1 mg, 250 µmol, 1 equiv). Reaction time was 60 min and 1.00 equiv of TBHP was added in total. ^{1}H NMR analysis of the unpurified product mixture with mesitylene as an internal standard (27.9 mg, 233 µmol, 0.930 equiv) revealed a 88% yield of (*Z*)-*tert*-butyl(pent-2-en-1-yloxy)diphenylsilane (**4c**) and a 11% yield of *tert*-butyl(pentyloxy)diphenylsilane (**S9**). Purification by automated flash-column chromatography (eluting with 2% ethyl acetate–hexanes initially, grading to 10% ethyl acetate–hexanes, linear gradient) afforded isobutyl 4-methoxybenzoate (**2a**) as a clear oil (47.8 mg, 92%).

2-Methallyl 4-methoxybenzoate (1a) versus (but-2-yn-1-yloxy)(tert-butyl)diphenylsilane (4d)

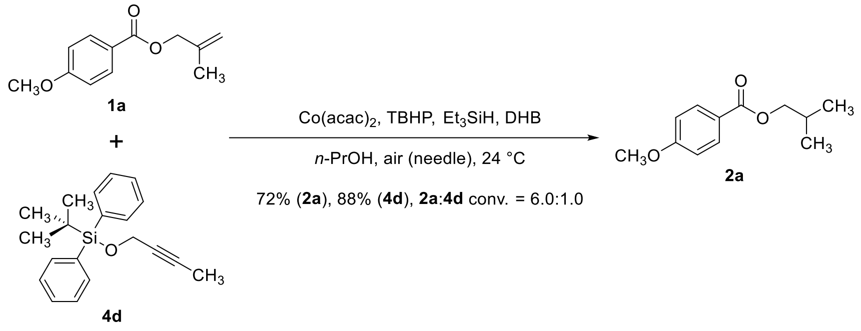

Condition A:

Following the general procedure A using 2-methylallyl 4-methoxybenzoate (**1a**, 51.6 mg, 250 μmol, 1 equiv) and (but-2-yn-1-yloxy)(*tert*-butyl)diphenylsilane (**4d**, 77.1 mg, 250 μmol, 1 equiv). Reaction time was 135 min and 2.00 equiv of TBHP was added in total. ^{1}H NMR analysis of the unpurified product mixture with mesitylene as an internal standard (29.0 mg, 242 μmol, 0.967 equiv) revealed a 88% yield of (but-2-yn-1-yloxy)(*tert*-butyl)diphenylsilane (**4d**). Purification by automated flash-column chromatography (eluting with 2% ether–hexanes initially, grading to 10% ether–hexanes, linear gradient) afforded isobutyl 4-methoxybenzoate (**2a**) as a clear oil (37.4 mg, 72%).

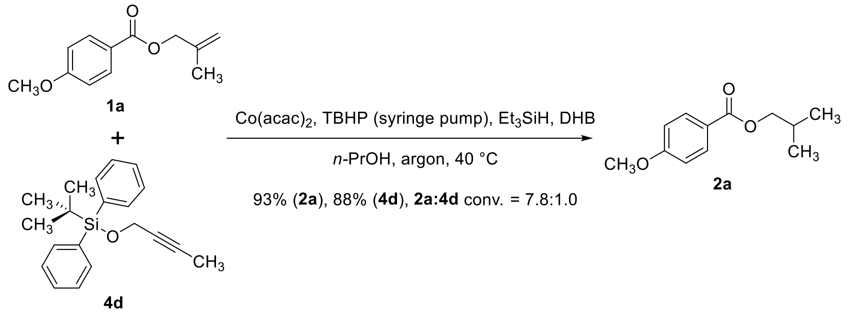

Condition B:

Following the general procedure B using 2-methylallyl 4-methoxybenzoate (**1a**, 51.6 mg, 250 μmol, 1 equiv) and (but-2-yn-1-yloxy)(*tert*-butyl)diphenylsilane (**4d**, 77.1 mg, 250 μmol, 1 equiv). Reaction time was 58 min and 0.97 equiv of TBHP were employed. ^{1}H NMR analysis of the unpurified product mixture using mesitylene as an internal standard (30.7 mg, 256 μmol, 1.02 equiv) revealed an 88% yield of (but-2-yn-1-yloxy)(*tert*-butyl)diphenylsilane (**4d**). Purification by automated flash-column chromatography (eluting with 2% ethyl acetate–hexanes initially, grading to 10% ethyl acetate–hexanes, linear gradient) afforded isobutyl 4-methoxybenzoate (**2a**) as a clear oil (48.4 mg, 93%).

2-Methallyl 4-methoxybenzoate (1a) versus tert-butyldiphenyl(prop-2-yn-1-yloxy)silane (4e)

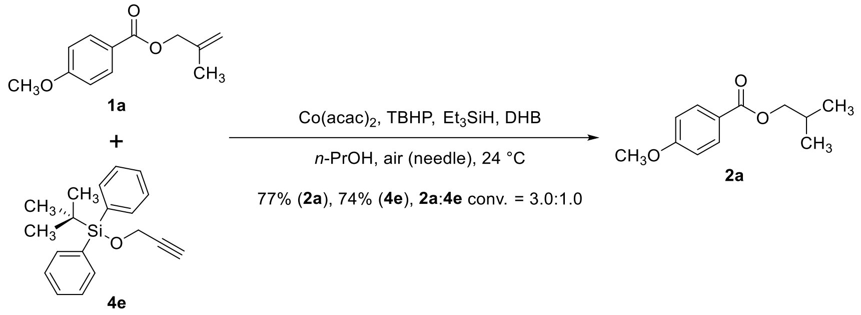

Condition A:

Following the general procedure A using 2-methylallyl 4-methoxybenzoate (**1a**, 51.6 mg, 250 μmol, 1 equiv) and *tert*-butyldiphenyl(prop-2-yn-1-yloxy)silane (**4e**, 73.6 mg, 250 μmol, 1 equiv). Reaction time was 135 min and 2.00 equiv of TBHP was added in total. ^{1}H NMR analysis of the unpurified product mixture with mesitylene as an internal standard (28.9 mg, 241 μmol, 0.963 equiv) revealed a 74% yield of *tert*-butyldiphenyl(prop-2-yn-1-yloxy)silane (**4e**). Purification by automated flash-column chromatography (eluting with 2% ether–hexanes initially, grading to 10% ether–hexanes, linear gradient) afforded isobutyl 4-methoxybenzoate (**2a**) as a clear oil (40.1 mg, 77%).

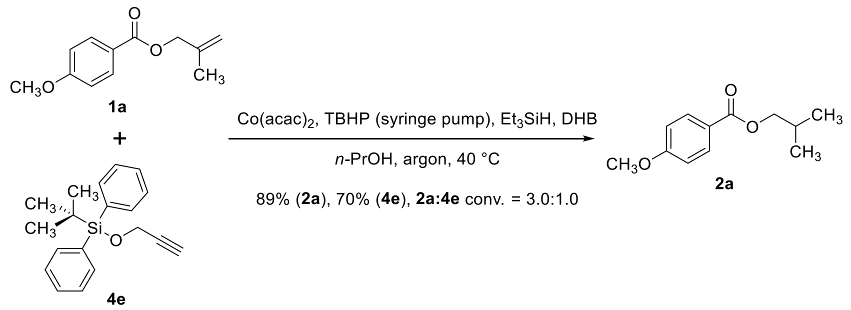

Condition B:

Following the general procedure B using 2-methylallyl 4-methoxybenzoate (**1a**, 51.6 mg, 250 μmol, 1 equiv) and *tert*-butyldiphenyl(prop-2-yn-1-yloxy)silane (**4e**, 73.6 mg, 250 μmol, 1 equiv). Reaction time was 100 min and 1.28 equiv of TBHP were employed. ^{1}H NMR analysis of the unpurified product mixture using mesitylene as an internal standard (30.9 mg, 258 μmol, 1.03 equiv) revealed a 70% yield of *tert*-butyldiphenyl(prop-2-yn-1-yloxy)silane (**4e**). Purification by automated flash-column chromatography (eluting with 2% ethyl acetate–hexanes initially, grading to 10% ethyl acetate–hexanes, linear gradient) afforded isobutyl 4-methoxybenzoate (**2a**) as a clear oil (46.3 mg, 89%).

2-Methallyl 4-methoxybenzoate (1a) versus 1-fluoro-4-(hex-1-yn-1-yl)benzene (4f)

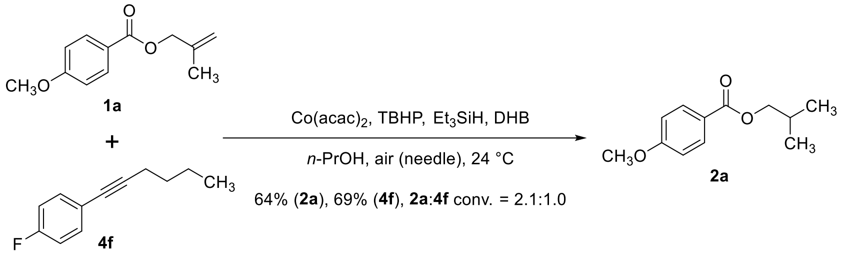

Condition A:

Following the general procedure A using 2-methylallyl 4-methoxybenzoate (**1a**, 51.6 mg, 250 μmol, 1 equiv) and 1-fluoro-4-(hex-1-yn-1-yl)benzene (**4f**, 44.1 mg, 250 μmol, 1 equiv). Reaction time was 135 min and 2.00 equiv of TBHP was added in total. ^{19}F NMR analysis of the unpurified product mixture with hexafluorobenzene as an internal standard (46.6 mg, 251 μmol, 1.00 equiv) revealed a 69% yield of 1-fluoro-4-(hex-1-yn-1-yl)benzene (**4f**). Purification by automated flash-column chromatography (eluting with 2% ether–hexanes initially, grading to 10% ether–hexanes, linear gradient) afforded isobutyl 4-methoxybenzoate (**2a**) as a clear oil (33.3 mg, 64%).

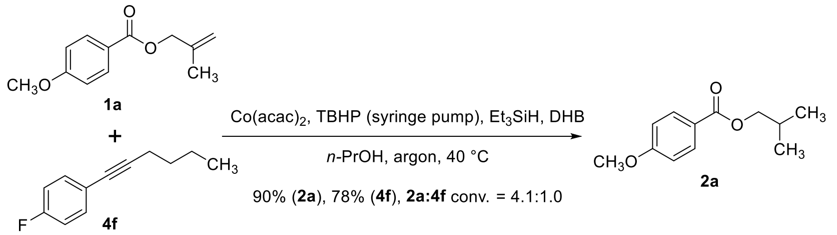

Condition B:

Following the general procedure B using 2-methylallyl 4-methoxybenzoate (**1a**, 51.6 mg, 250 μmol, 1 equiv) and 1-fluoro-4-(hex-1-yn-1-yl)benzene (**4f**, 44.1 mg, 250 μmol, 1 equiv). Reaction time was 70 min and 1.00 equiv of TBHP were employed. ^{19}F NMR analysis of the unpurified product mixture using hexafluorobenzene as an internal standard (46.3 mg, 249 μmol, 0.996 equiv) revealed a 78% yield of 1-fluoro-4-(hex-1-yn-1-yl)benzene (**4f**). Purification by automated flash-column chromatography (eluting with 2% ethyl acetate–hexanes initially, grading to 10% ethyl acetate–hexanes, linear gradient) afforded isobutyl 4-methoxybenzoate (**2a**) as a clear oil (46.8 mg, 90%).

3-Methylbut-2-en-1-yl (4-methoxyphenyl)carbamate (1g) versus (allyloxy)(tert-butyl)diphenylsilane (4a)

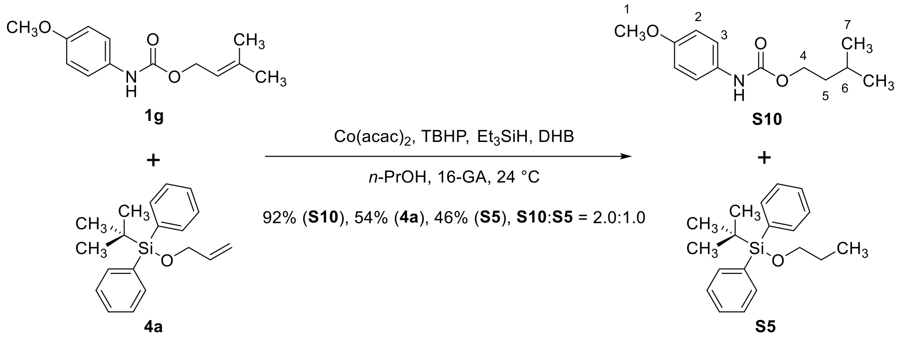

Condition A:

Following the general procedure A using 3-methylbut-2-en-1-yl (4-methoxyphenyl)carbamate (**1g**, 57.8 mg, 250 µmol, 1 equiv) and (allyloxy)(*tert*-butyl)diphenylsilane (**4a**, 74.0 mg, 250 µmol, 1 equiv). A solution of *tert*-butyl hydroperoxide in nonane (~5.5 M, 22.8 µL, 125 µmol, 0.50 equiv) was added every half an hour. Reaction time was 420 min and 8.00 equiv of TBHP were employed. ^{1}H NMR analysis of the unpurified product mixture using mesitylene as an internal standard (29.9 mg, 249 µmol, 0.998 equiv) revealed a 54% yield of (allyloxy)(*tert*-butyl)diphenylsilane (**4a**) and a 46% yield of *tert*-butyldiphenyl(propoxy)silane (**S5**). Purification by automated flash-column chromatography (eluting with 2% ethyl acetate–hexanes initially, grading to 10% ethyl acetate–hexanes, linear gradient) afforded isopentyl (4-methoxyphenyl)carbamate (**S10**) as a white solid (54.4 mg, 92%).

R_f = 0.52 (33% ethyl acetate–hexanes; UV, CAM). ^{1}H NMR (400 MHz, CD_2Cl_2) δ 7.28 (d, J = 8.4 Hz, 2H, H_3), 6.89 (d, J = 8.8 Hz, 2H, H_2), 6.60 (br s, 1H, NH), 4.16 (t, J = 7.0 Hz, 2H, H_4), 3.77 (s, 3H, H_1), 1.76–1.69 (m, 1H, H_6), 1.60–1.53 (m, 2H, H_5), 0.94 (d, 6H, J = 6.4 Hz, H_7). ^{13}C NMR (150 MHz, CD_2Cl_2) δ 156.4 (C), 154.5 (C), 131.8 (C), 121.0 (CH), 114.6 (CH), 64.2 (CH_2), 56.0 (CH_3), 38.2 (CH_2), 25.6 (CH), 22.8 (CH_3). IR (ATR-FTIR), cm^{-1}: 3317 (w), 2959 (m), 1698 (s), 1512 (s), 1213 (s). HRMS-ESI (m/z): $[M + H]^+$ calcd for $C_{13}H_{19}NO_3$, 238.1443; found, 238.1441.

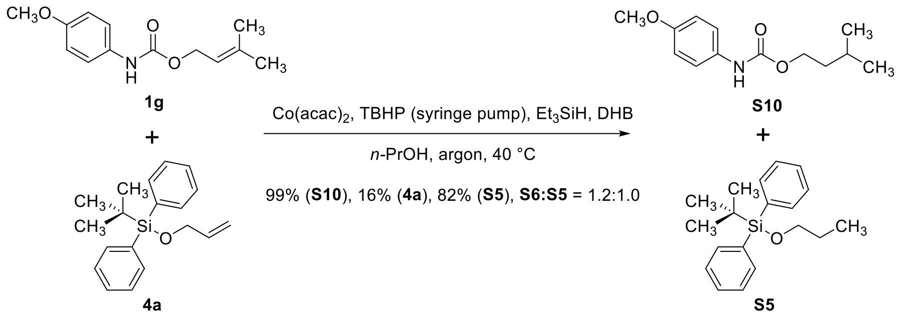

Condition B:

Following the general procedure B using 3-methylbut-2-en-1-yl (4-methoxyphenyl)carbamate (**1g**, 57.8 mg, 250 µmol, 1 equiv) and (allyloxy)(*tert*-butyl)diphenylsilane (**4a**, 74.0 mg, 250 µmol, 1 equiv). Reaction time was 180 min and 3.00 equiv of TBHP was added in total. ^{1}H NMR analysis of the unpurified product mixture with mesitylene as an internal standard (32.4 mg, 270 µmol, 1.08 equiv) revealed a 16% yield of (allyloxy)(*tert*-butyl)diphenylsilane (**4a**) and a 82% yield of *tert*-butyldiphenyl(propoxy)silane (**S5**). Purification by automated flash-column chromatography (eluting with 2% ethyl acetate–hexanes initially, grading to 10% ethyl acetate–hexanes, linear gradient) afforded isopentyl (4-methoxyphenyl)carbamate (**S10**) as a white solid (59.5 mg, >99%).

(E)-2-methylbut-2-en-1-yl (4-methoxyphenyl)carbamate (1h) versus (allyloxy)(tert-butyl)diphenylsilane (4a)

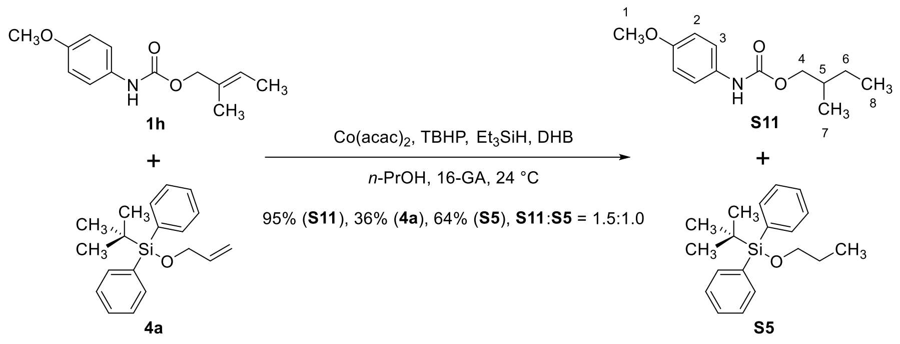

Condition A:

Following the general procedure A using (*E*)-2-methylbut-2-en-1-yl (4-methoxyphenyl)carbamate (**1h**, 57.8 mg, 250 μmol) and (allyloxy)(*tert*-butyl)diphenylsilane (**4a**, 74.0 mg, 250 μmol, 1 equiv). A solution of *tert*-butyl hydroperoxide in nonane (~5.5 M, 22.8 μL, 125 μmol, 0.50 equiv) was added every half an hour. Reaction time was 420 min and 8.00 equiv of TBHP were employed. ^{1}H NMR analysis of the unpurified product mixture using mesitylene as an internal standard (33.8 mg, 282 μmol, 1.13 equiv) revealed a 36% yield of (allyloxy)(*tert*-butyl)diphenylsilane (**4a**) and a 64% yield of *tert*-butyldiphenyl(propoxy)silane (**S5**). Purification by automated flash-column chromatography (eluting with 2% ethyl acetate–hexanes initially, grading to 10% ethyl acetate–hexanes, linear gradient) afforded 2-methylbutyl (4-methoxyphenyl)carbamate (**S11**) as a white solid (55.9 mg, 95%).

R_f = 0.52 (33% ethyl acetate–hexanes; UV, CAM). ^{1}H NMR (400 MHz, $CDCl_3$) δ 7.29 (br d, J = 8.4 Hz, 2H, H_3), 6.85 (d, J = 8.8 Hz, 2H, H_2), 6.48 (br s, 1H, NH), 4.07–4.02 (m, 1H, 1 × H_4), 3.97–3.93 (m, 1H, 1 × H_4), 3.79 (s, 3H, H_1), 1.78–1.70 (m, 1H, H_5), 1.50–1.43 (m, 1H, 1 × H_6), 1.26–1.15 (m, 1H, 1 × H_6), 0.96-0.91 (m, 6H, 3 × H_7, 3 × H_8). ^{13}C NMR (150 MHz, $CDCl_3$) δ 155.9 (C), 154.1 (C), 131.0 (C), 120.5 (CH), 114.2 (CH), 69.8 (CH_2), 565.5 (CH_3), 34.4 (CH), 26.0 (CH_2), 16.4 (CH_3), 11.2 (CH_3). IR (ATR-FTIR), cm^{-1}: 3315 (w), 2961 (m), 1699 (s), 1512 (s), 1216 (s). HRMS-ESI (m/z): $[M + H]^+$ calcd for $C_{13}H_{19}NO_3$, 238.1443; found, 238.1432.

3-Methylbut-2-en-1-yl (4-methoxyphenyl)carbamate (1g) versus (E)-tert-butyl(pent-2-en-1-yloxy)diphenylsilane (4b)

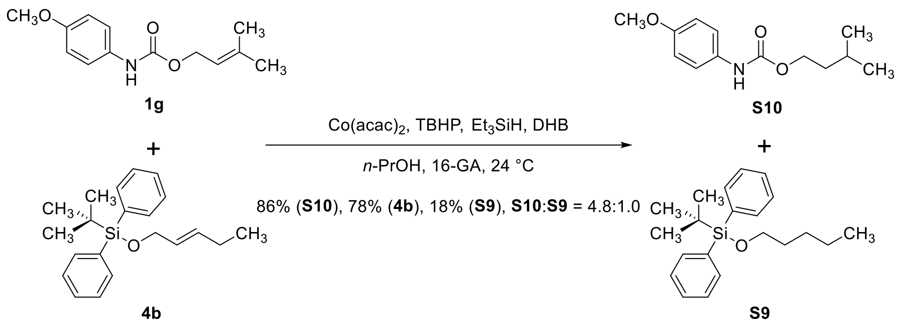

Condition A:

Following the general procedure A using 3-methylbut-2-en-1-yl (4-methoxyphenyl)carbamate (**1g**, 57.8 mg, 250 µmol, 1 equiv) and (*E*)-*tert*-butyl(pent-2-en-1-yloxy)diphenylsilane (**4b**, 81.1 mg, 250 µmol, 1 equiv). A solution of *tert*-butyl hydroperoxide in nonane (~5.5 M, 22.8 µL, 125 µmol, 0.50 equiv) was added every half an hour. Reaction time was 360 min and 5.00 equiv of TBHP were employed. Purification by automated flash-column chromatography (eluting with 2% ethyl acetate–hexanes initially, grading to 10% ethyl acetate–hexanes, linear gradient) afforded isopentyl (4-methoxyphenyl)carbamate (**S10**) as a white solid (51.1 mg, 86%). All the other fractions are combined and concentrated. ^{1}H NMR analysis of the residue using mesitylene as an internal standard (24.5 mg, 204 µmol, 0.817 equiv) revealed a 78% yield of (*E*)-*tert*-butyl(pent-2-en-1-yloxy)diphenylsilane (**4b**) and an 18% yield of *tert*-butyl(pentyloxy)diphenylsilane (**S9**).

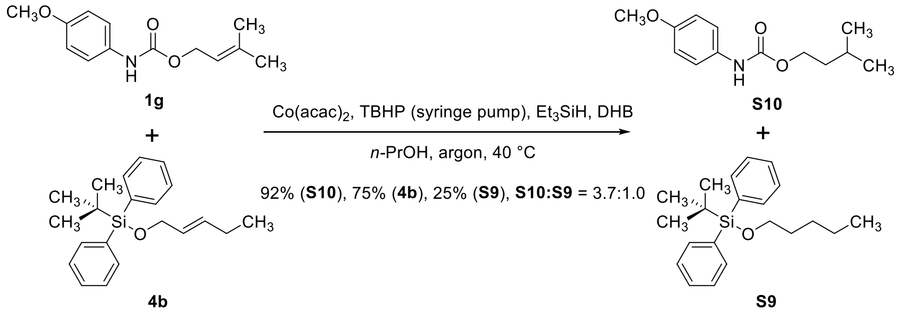

Condition B:

Following the general procedure B using 3-methylbut-2-en-1-yl (4-methoxyphenyl)carbamate (**1g**, 57.8 mg, 250 µmol, 1 equiv) and (*E*)-*tert*-butyl(pent-2-en-1-yloxy)diphenylsilane (**4b**, 81.1 mg, 250 µmol, 1 equiv). Reaction time was 135 min and 2.20 equiv of TBHP was added in total. Purification by automated flash-column chromatography (eluting with 2% ethyl acetate–hexanes initially, grading to 10% ethyl acetate–hexanes, linear gradient) afforded isopentyl (4-methoxyphenyl)carbamate (**S10**) as a white solid (54.5 mg, 92%). All the other fractions are combined and concentrated. ^{1}H NMR analysis of the residue with mesitylene as an internal standard (22.1 mg, 184 µmol, 0.737 equiv) revealed a 75% yield of (*E*)-*tert*-butyl(pent-2-en-1-yloxy)diphenylsilane (**4b**) and a 25% yield of *tert*-butyl(pentyloxy)diphenylsilane (**S9**).

3-Methylbut-2-en-1-yl (4-methoxyphenyl)carbamate (1g) versus (Z)-tert-butyl(pent-2-en-1-yloxy)diphenylsilane (4c)

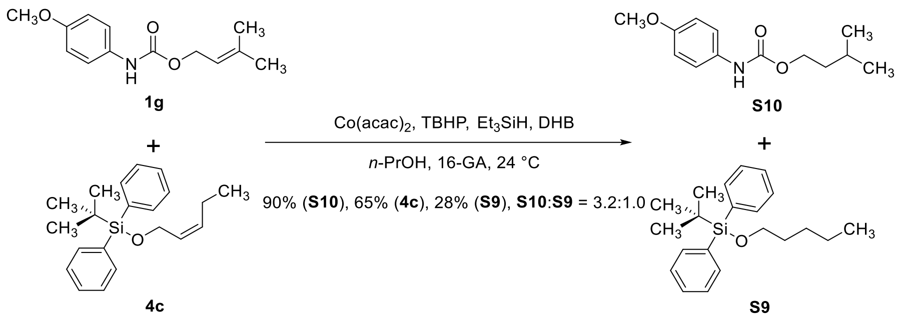

Condition A:

Following the general procedure A using 3-methylbut-2-en-1-yl (4-methoxyphenyl)carbamate (**1g**, 57.8 mg, 250 μmol, 1 equiv) and (*Z*)-*tert*-butyl(pent-2-en-1-yloxy)diphenylsilane (**4c**, 81.1 mg, 250 μmol, 1 equiv). A solution of *tert*-butyl hydroperoxide in nonane (~5.5 M, 22.8 μL, 125 μmol, 0.50 equiv) was added every half an hour. Reaction time was 360 min and 6.50 equiv of TBHP were employed. ^{1}H NMR analysis of the unpurified product mixture using mesitylene as an internal standard (28.6 mg, 238 μmol, 0.953 equiv) revealed a 65% yield of (*Z*)-*tert*-butyl(pent-2-en-1-yloxy)diphenylsilane (**4c**) and a 28% yield of *tert*-butyl(pentyloxy)diphenylsilane (**S9**). Purification by automated flash-column chromatography (eluting with 2% ethyl acetate–hexanes initially, grading to 10% ethyl acetate–hexanes, linear gradient) afforded isopentyl (4-methoxyphenyl)carbamate (**S10**) as a white solid (53.2 mg, 90%).

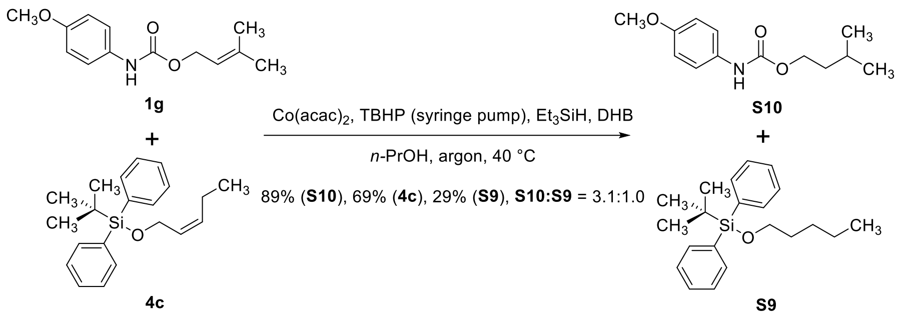

Condition B:

Following the general procedure B using 3-methylbut-2-en-1-yl (4-methoxyphenyl)carbamate (**1g**, 57.8 mg, 250 μmol, 1 equiv) and (*Z*)-*tert*-butyl(pent-2-en-1-yloxy)diphenylsilane (**4c**, 81.1 mg, 250 μmol, 1 equiv). Reaction time was 135 min and 2.20 equiv of TBHP was added in total. ^{1}H NMR analysis of the unpurified product mixture with mesitylene as an internal standard (51.3 mg, 428 μmol, 1.71 equiv) revealed a 69% yield of (*Z*)-*tert*-butyl(pent-2-en-1-yloxy)diphenylsilane (**4c**) and a 29% yield of *tert*-butyl(pentyloxy)diphenylsilane (**S9**). Purification by automated flash-column chromatography (eluting with 2% ethyl acetate–hexanes initially, grading to 10% ethyl acetate–hexanes, linear gradient) afforded isopentyl (4-methoxyphenyl)carbamate (**S10**) as a white solid (52.9 mg, 89%).

2-Methallyl 4-methoxybenzoate (1a) versus 1-fluoro-3-vinylbenzene (4g)

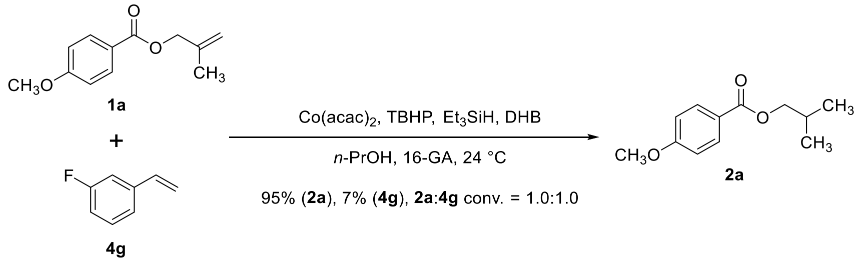

Condition A:

Following the general procedure A using 2-methylallyl 4-methoxybenzoate (**1a**, 51.6 mg, 250 μmol, 1 equiv) and 1-fluoro-3-vinylbenzene (**4g**, 30.5 mg, 250 μmol, 1 equiv). Reaction time was 135 min and 2.00 equiv of TBHP were employed. ^{19}F NMR analysis of the unpurified product mixture using hexafluorobenzene as an internal standard revealed (34.0 mg, 183 μmol, 0.732 equiv) a 7% yield of 1-fluoro-3-vinylbenzene (**4g**). Purification by automated flash-column chromatography (eluting with 2% ether–hexanes initially, grading to 10% ether–hexanes, linear gradient) afforded isobutyl 4-methoxybenzoate (**2a**) as a clear oil (49.6 mg, 95%).

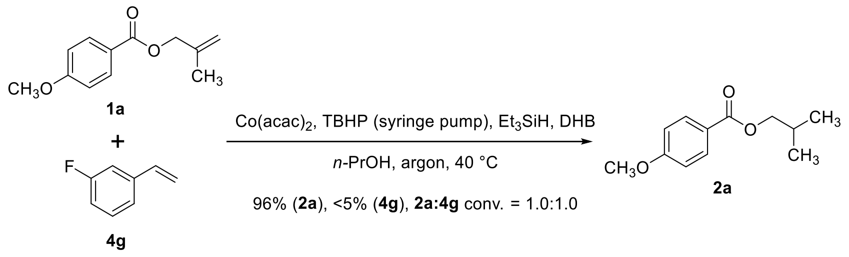

Conditioni B:

Following the general procedure B using 2-methylallyl 4-methoxybenzoate (**1a**, 51.6 mg, 250 μmol, 1 equiv) and 1-fluoro-3-vinylbenzene (**4g**, 30.5 mg, 250 μmol, 1 equiv). Reaction time was 70 min and 1.00 equiv of TBHP was added in total. ^{19}F NMR analysis of the unpurified product mixture with hexafluorobenzene as an internal standard (39.0 mg, 210 μmol, 0.839 equiv) revealed a <5% yield of 1-fluoro-3-vinylbenzene (**4g**). Purification by automated flash-column chromatography (eluting with 2% ethyl acetate–hexanes initially, grading to 10% ethyl acetate–hexanes, linear gradient) afforded isobutyl 4-methoxybenzoate (**2a**) as a clear oil (50.1 mg, 96%).

2-Methallyl 4-methoxybenzoate (1a) versus 1-ethynyl-4-(trifluoromethyl)benzene (4h)

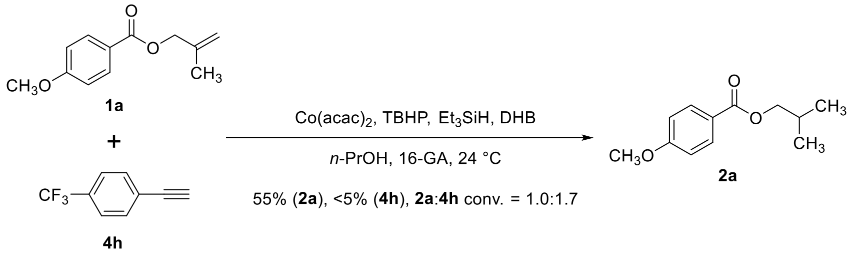

Condition A:

Following the general procedure A using 2-methylallyl 4-methoxybenzoate (**1a**, 51.6 mg, 250 µmol, 1 equiv) and 1-ethynyl-4-(trifluoromethyl)benzene (**4h**, 42.5 mg, 250 µmol, 1 equiv). Reaction time was 135 min and 2.00 equiv of TBHP were employed. ^{19}F NMR analysis of the unpurified product mixture using hexafluorobenzene as an internal standard (49.8 mg, 268 µmol, 1.07 equiv) revealed a <5% yield of 1-ethynyl-4-(trifluoromethyl)benzene (**4h**). Purification by automated flash-column chromatography (eluting with 2% ether–hexanes initially, grading to 10% ether–hexanes, linear gradient) afforded isobutyl 4-methoxybenzoate (**2a**) as a clear oil (28.6 mg, 55%).

2-Fluoroallyl (4-methoxyphenyl)carbamate (1i) versus (allyloxy)(tert-butyl)diphenylsilane (4a)

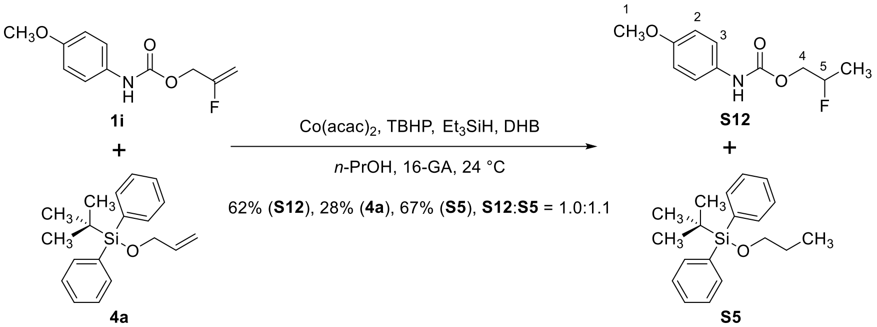

Condition A:

Following the general procedure A using 2-fluoroallyl (4-methoxyphenyl)carbamate (**1i**, 56.3 mg, 250 μmol) and (allyloxy)(*tert*-butyl)diphenylsilane (**4a**, 74.0 mg, 250 μmol, 1 equiv). Reaction time was 19 h but only 1.00 equiv of TBHP was added to initially activate the catalyst. ^{1}H NMR analysis of the unpurified product mixture using mesitylene as an internal standard revealed a 28% yield of (allyloxy)(*tert*-butyl)diphenylsilane (**4a**) and a 67% yield of *tert*-butyldiphenyl(propoxy)silane (**S5**). Purification by automated flash-column chromatography (eluting with 2% ethyl acetate–hexanes initially, grading to 10% ethyl acetate–hexanes, linear gradient) afforded 2-fluoropropyl (4-methoxyphenyl)carbamate (**S12**) as a white solid (35.5 mg, 62%).

R_f = 0.58 (33% ethyl acetate–hexanes; UV, CAM). ^{1}H NMR (400 MHz, $CDCl_3$) δ 7.27 (br d, 2H, J = 8.0 Hz, H_3), 6.85 (d, 2H, J = 8.8 Hz, H_2), 6.74 (br s, 1H, NH), 4.99–4.78 (m, 1H, H_5), 4.35–4.11 (m, 2H, H_4), 3.78 (s, 3H, H_1), 1.37 (dd, 3H, J = 23.6, 6.4 Hz, H_6). ^{19}F NMR (375 MHz, $CDCl_3$) δ –47.71.

^{1}H and ^{19}F NMR data for 2-fluoropropyl (4-methoxyphenyl)carbamate (**S12**) prepared in this way were in agreement with those previously described.[20]

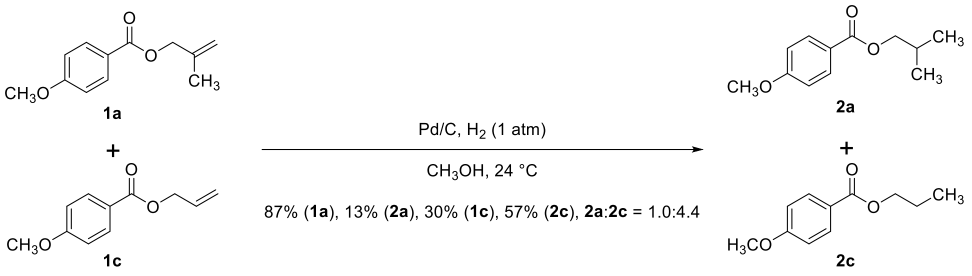

Competitive hydrogenation employing palladium on carbon (Table S3, Entry 1 and Table 3, Entry 2, H_2/Pd-C)

Palladium on carbon (10 wt. % loading, 1.1 mg, 1.0 μmol, 0.01 equiv) was added to a solution of 2-methylallyl 4-methoxybenzoate (**1a**, 20.6 mg, 100 μmol, 1 equiv) and allyl 4-methoxybenzoate (**1c**, 19.2 mg, 100 μmol, 1 equiv) in methanol (10 mL) at 24 °C. The reaction vessel was evacuated and re-filled using a balloon of dihydrogen. This process was repeated four times. The reaction mixture was stirred for 5 min at 24 °C. The product mixture was filtered through a short column of celite and the short column was rinsed with ether (100 mL). The filtrates were combined and the combined filtrates were concentrated. ^{1}H NMR analysis of the unpurified product mixture using mesitylene as an internal standard (5.0 mg, 42.0 μmol , 0.420 equiv) revealed an 87% yield of 2-methallyl 4-methoxybenzoate (**1a**), a 13% yield of isobutyl 4-methoxybenzoate (**2a**), a 30% yield of allyl 4-methoxybenzoate (**1c**), and a 57% yield of propyl 4-methoxybenzoate (**2c**).

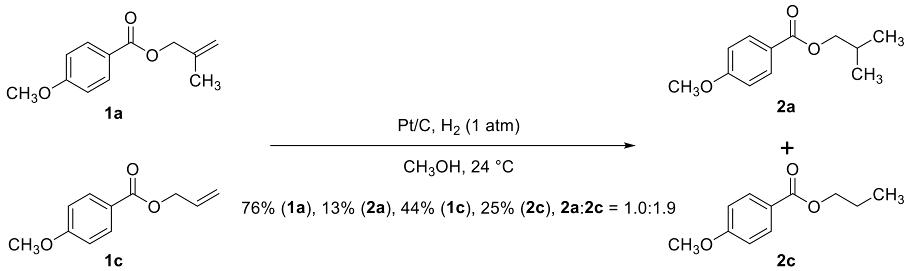

Competitive hydrogenation employing platinum on carbon (Table S3, Entry 2)

Palladium on carbon (10 wt. % loading, 1.0 mg, 0.5 µmol, 0.005 equiv) was added to a solution of 2-methylallyl 4-methoxybenzoate (**1a**, 20.6 mg, 100 µmol, 1 equiv) and allyl 4-methoxybenzoate (**1c**, 19.2 mg, 100 µmol, 1 equiv) in methanol (10 mL) at 24 °C. The reaction vessel was evacuated and re-filled using a balloon of dihydrogen. This process was repeated four times. The reaction mixture was stirred for 3 min at 24 °C. The product mixture was filtered through a short column of celite and the short column was rinsed with ether (100 mL). The filtrates were combined and the combined filtrates were concentrated. ^{1}H NMR analysis of the unpurified product mixture using mesitylene as an internal standard (7.7 mg, 6.43 µmol , 0.643 equiv) revealed an 76% yield of 2-methallyl 4-methoxybenzoate (**1a**), a 13% yield of isobutyl 4-methoxybenzoate (**2a**), a 44% yield of allyl 4-methoxybenzoate (**1c**), and a 25% yield of propyl 4-methoxybenzoate (**2c**).

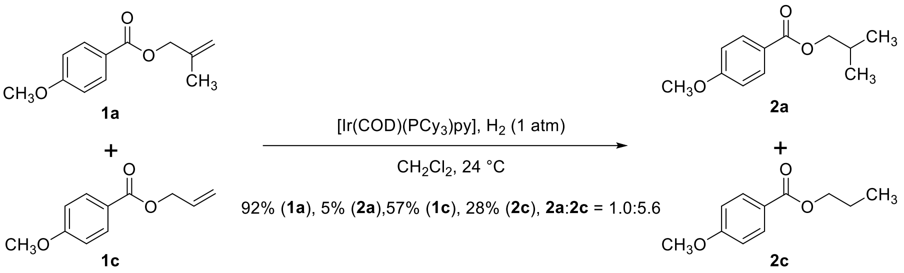

Competitive hydrogenation employing Crabtree's catalyst (Table S3, Entry 3)

Crantree's catalyst (2.5 mol%, 2.0 mg, 0.25 μmol, 0.0025 equiv) was added to a solution of 2-methylallyl 4-methoxybenzoate (**1a**, 20.6 mg, 100 μmol, 1 equiv) and allyl 4-methoxybenzoate (**1c**, 19.2 mg, 100 μmol, 1 equiv) in dichloromethane (500 μL) at 24 °C. The reaction vessel was evacuated and re-filled using a balloon of dihydrogen. This process was repeated four times. The reaction mixture was stirred for 3 h at 24 °C. The product mixture was filtered through a short column of silica gel and the short column was rinsed with dichloromethane (200 mL). The filtrates were combined and the combined filtrates were concentrated. ^{1}H NMR analysis of the unpurified product mixture using mesitylene as an internal standard (6.9 mg, 5.73 μmol , 0.573 equiv) revealed a 92% yield of 2-methallyl 4-methoxybenzoate (**1a**), a 5% yield of isobutyl 4-methoxybenzoate (**2a**), a 57% yield of allyl 4-methoxybenzoate (**1c**), and a 28% yield of propyl 4-methoxybenzoate (**2c**).

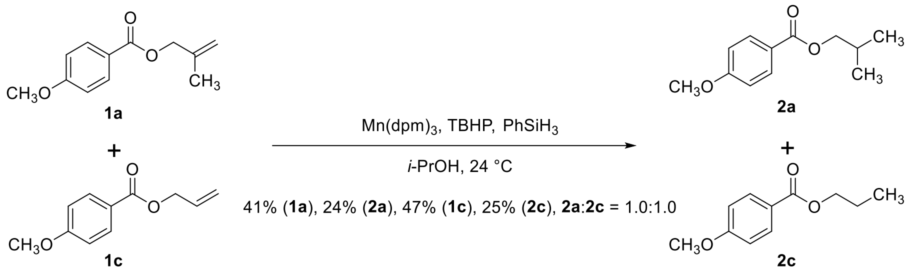

Competitive hydrogenation employing manganese tris(dipivaloylmethane) (Table S3, Entry 4)

This experiment followed the procedure of Shenvi and co-workers.[21] Phenylsilane (64.5 µL, 600 µmol, 6.00 equiv) and a solution of *tert*-butyl hydroperoxide in nonane (~5.5 M, 36.4 µL, 200 µmol, 2.00 equiv) were added sequentially to a solution of 2-methylallyl 4-methoxybenzoate (**1a**, 20.6 mg, 100 µmol, 1 equiv), allyl 4-methoxybenzoate (**1c**, 19.2 mg, 100 µmol, 1 equiv) in *i*-propanol (200 µL) under argon at 24 °C. The resulting mixture was degassed by bubbling argon through the solution for 10 min. Manganese tris(dipivaloylmethane) (9.0 mg, 15.0 µmol, 0.15 equiv) was added and the resulting mixture was degassed by bubbling argon through the solution for 30 s. The reaction mixture was stirred for 10 min at 24 °C. The reaction was quenched by filtering the product mixture through a plug of silica gel and the plug was rinsed with 20% ethyl acetate–hexanes (100 mL). The filtrates were combined and the combined filtrates were concentrated. ^{1}H NMR analysis of the unpurified product mixture with mesitylene as an internal standard (9.4 mg, 78.4 µmol, 0.784 equiv) revealed a 41% yield of 2-methylallyl 4-methoxybenzoate (**1a**), a 24% yield of isobutyl 4-methoxybenzoate (**2a**), a 47% yield of allyl 4-methoxybenzoate (**1c**), and a 25% yield of propyl 4-methoxybenzoate (**2c**).

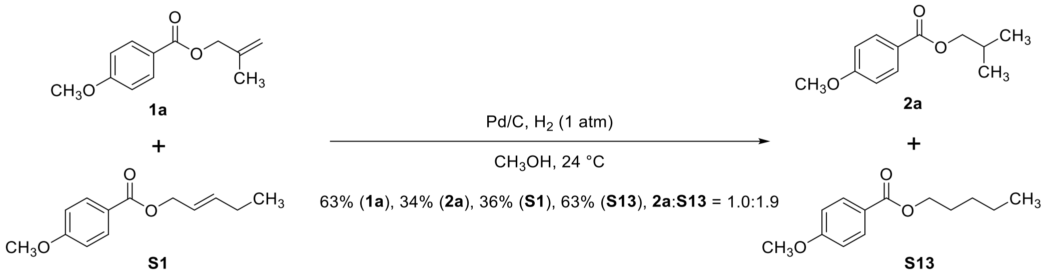

Competitive hydrogenation employing palladium on carbon (Table 3, Entry 2, H_2/Pd-C)

Palladium on carbon (10 wt. % loading, 1.1 mg, 1.0 μmol, 0.01 equiv) was added to a solution of 2-methylallyl 4-methoxybenzoate (**1a**, 20.6 mg, 100 μmol, 1 equiv) and (*E*)-pent-2-en-1-yl 4-methoxybenzoate (**S1**, 22.6 mg, 100 μmol, 1 equiv) in methanol (10 mL) at 24 °C. The reaction vessel was evacuated and re-filled using a balloon of dihydrogen. This process was repeated four times. The reaction mixture was stirred for 5 min at 24 °C. The product mixture was filtered through a short column of celite and the short column was rinsed with ether (100 mL). The filtrates were combined and the combined filtrates were concentrated. ^{1}H NMR analysis of the unpurified product mixture using mesitylene as an internal standard (9.8 mg, 82.0 μmol, 0.820 equiv) revealed a 63% yield of 2-methylallyl 4-methoxybenzoate (**1a**), a 34% yield of isobutyl 4-methoxybenzoate (**2a**), a 36% yield of (*E*)-pent-2-en-1-yl 4-methoxybenzoate (**S1**), and a 63% yield of pentyl 4-methoxybenzoate (**S13**).

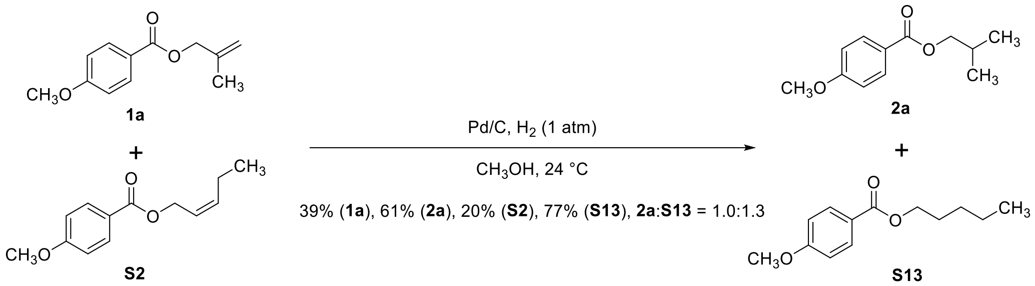

Competitive hydrogenation employing palladium on carbon (Table 3, Entry 3, H_2/Pd-C)

Palladium on carbon (10 wt. % loading, 1.1 mg, 1.0 μmol, 0.01 equiv) was added to a solution of 2-methylallyl 4-methoxybenzoate (**1a**, 20.6 mg, 100 μmol, 1 equiv) and (*Z*)-pent-2-en-1-yl 4-methoxybenzoate (**S2**, 22.6 mg, 100 μmol, 1 equiv) in methanol (10 mL) at 24 °C. The reaction vessel was evacuated and re-filled using a balloon of dihydrogen. This process was repeated four times. The reaction mixture was stirred for 5 min at 24 °C. The product mixture was filtered through a short column of celite and the short column was rinsed with ether (100 mL). The filtrates were combined and the combined filtrates were concentrated. ^{1}H NMR analysis of the unpurified product mixture using mesitylene as an internal standard (15.6 mg, 130 μmol, 1.30 equiv) revealed a 39% yield of 2-methylallyl 4-methoxybenzoate (**1a**), a 61% yield of isobutyl 4-methoxybenzoate (**2a**), a 20% yield of (*Z*)-pent-2-en-1-yl 4-methoxybenzoate (**S2**), and a 77% yield of pentyl 4-methoxybenzoate (**S13**).

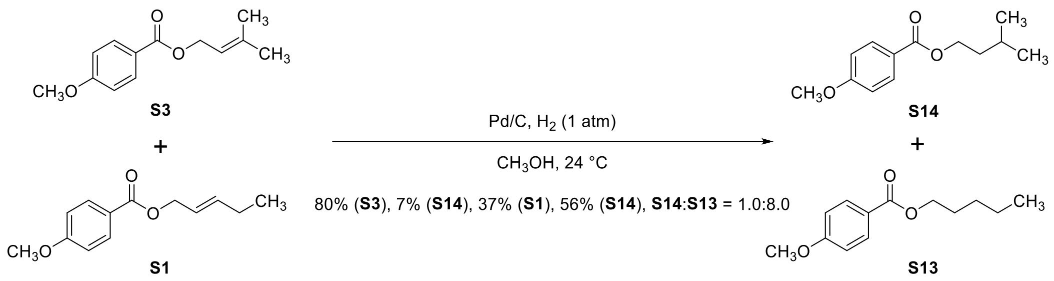

Competitive hydrogenation employing palladium on carbon (Table 3, Entry 4, H_2/Pd-C)

Palladium on carbon (10 wt. % loading, 1.1 mg, 1.0 µmol, 0.01 equiv) was added to a solution of 3-methylbut-2-en-1-yl 4-methoxybenzoate (**S3**, 22.6 mg, 100 µmol, 1 equiv) and (*E*)-pent-2-en-1-yl 4-methoxybenzoate (**S1**, 22.6 mg, 100 µmol, 1 equiv) in methanol (10 mL) at 24 °C. The reaction vessel was evacuated and re-filled using a balloon of dihydrogen. This process was repeated four times. The reaction mixture was stirred for 5 min at 24 °C. The product mixture was filtered through a short column of celite and the short column was rinsed with ether (100 mL). The filtrates were combined and the combined filtrates were concentrated. ^{1}H NMR analysis of the unpurified product mixture using mesitylene as an internal standard (10.9 mg, 90.3 µmol, 0.903 equiv) revealed a 80% yield of 3-methylbut-2-en-1-yl 4-methoxybenzoate (**S3**), a 7% yield of isopentyl 4-methoxybenzoate (**S14**), a 37% yield of (*E*)-pent-2-en-1-yl 4-methoxybenzoate (**S1**), and a 56% yield of pentyl 4-methoxybenzoate (**S13**).

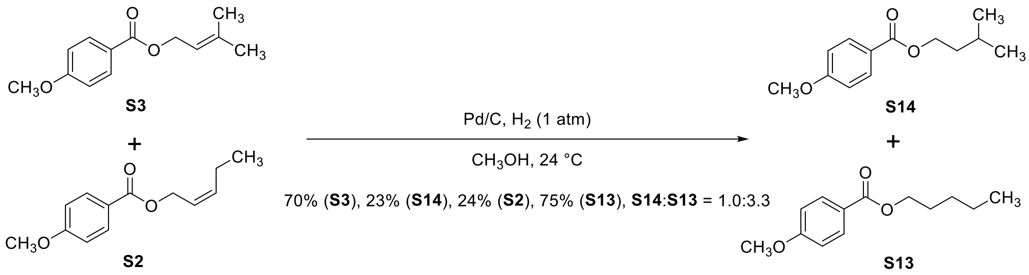

Competitive hydrogenation employing palladium on carbon (Table 3, Entry 5, H_2/Pd-C)

Palladium on carbon (10 wt. % loading, 1.1 mg, 1.0 μmol, 0.01 equiv) was added to a solution of 3-methylbut-2-en-1-yl 4-methoxybenzoate (**S3**, 22.6 mg, 100 μmol, 1 equiv) and (*E*)-pent-2-en-1-yl 4-methoxybenzoate (**S2**, 22.6 mg, 100 μmol, 1 equiv) in methanol (10 mL) at 24 °C. The reaction vessel was evacuated and re-filled using a balloon of dihydrogen. This process was repeated four times. The reaction mixture was stirred for 5 min at 24 °C. The product mixture was filtered through a short column of celite and the short column was rinsed with ether (100 mL). The filtrates were combined and the combined filtrates were concentrated. ^{1}H NMR analysis of the unpurified product mixture using mesitylene as an internal standard (9.7 mg, 80.3 μmol, 0.803 equiv) revealed a 70% yield of 3-methylbut-2-en-1-yl 4-methoxybenzoate (**S3**), a 23% yield of isopentyl 4-methoxybenzoate (**S14**), a 24% yield of (*E*)-pent-2-en-1-yl 4-methoxybenzoate (**S2**), and a 75% yield of pentyl 4-methoxybenzoate (**S13**).

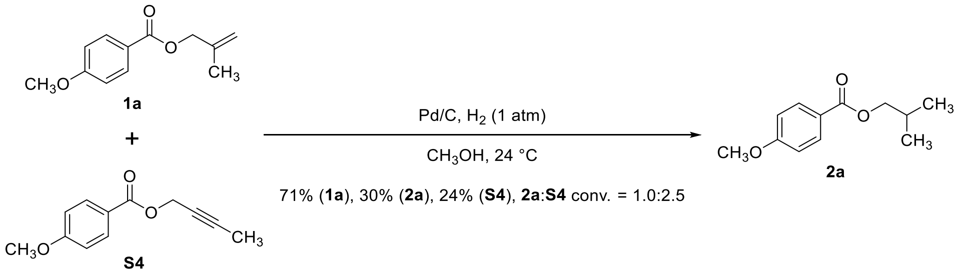

Competitive hydrogenation employing palladium on carbon (Table 3, Entry 6, H_2/Pd-C)

Palladium on carbon (10 wt. % loading, 1.1 mg, 1.0 μmol, 0.01 equiv) was added to a solution of 2-methylallyl 4-methoxybenzoate (**1a**, 22.6 mg, 100 μmol, 1 equiv) and but-2-yn-1-yl 4-methoxybenzoate (**S4**, 20.4 mg, 100 μmol, 1 equiv) in methanol (10 mL) at 24 °C. The reaction vessel was evacuated and re-filled using a balloon of dihydrogen. This process was repeated four times. The reaction mixture was stirred for 5 min at 24 °C. The product mixture was filtered through a short column of celite and the short column was rinsed with ether (100 mL). The filtrates were combined and the combined filtrates were concentrated. ^{1}H NMR analysis of the unpurified product mixture using mesitylene as an internal standard (11.5 mg, 96.0 μmol, 0.960 equiv) revealed a 71% yield of 2-methylallyl 4-methoxybenzoate (**1a**), a 30% yield of isobutyl 4-methoxybenzoate (**2a**), and a 24% yield of but-2-yn-1-yl 4-methoxybenzoate (**S4**).

Hydrobromination of 2-methylallyl 4-methoxybenzoate (1a) to 2-bromo-2-methylpropyl 4-methoxybenzoate (5a, Table S4, entry 2)

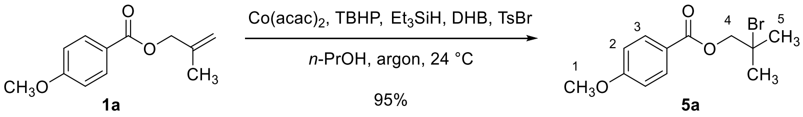

A 10 mL round-bottomed flask fitted with a rubber septum was charged sequentially with 2-methylallyl 4-methoxybenzoate (**1a**, 51.6 mg, 250 µmol, 1 equiv), tosyl bromide (147 mg, 625 µmol, 2.50 equiv), and cobalt bis(acetylacetonate) (64.3 mg, 250 µmol, 1.00 equiv). The reaction vessel was evacuated and refilled using a balloon of argon. This process was repeated twice. *n*-Propanol (830 µL), 1,4-dihydrobenzene (86.0 µL, 938 µmol, 3.75 equiv), a solution of *tert*-butyl hydroperoxide in nonane (~5.5 M, 45.5 µL, 250 µmol, 1.00 equiv), and triethylsilane (400 µL, 2.50 mmol, 10.0 equiv) were added sequentially to the reaction vessel via syringe. The reaction mixture was stirred for 4.0 h at 24 °C. The product mixture was filtered through a short column of silica gel and the short column was rinsed with 20% ethyl acetate–hexanes (100 mL). The filtrates were combined and the combined filtrates were concentrated. The residue obtained was purified by flash-column chromatography (eluting with 2% ethyl acetate–hexanes initially, grading to 7% ethyl acetate–hexanes, linear gradient) to 2-bromo-2-methylpropyl 4-methoxybenzoate (**5a**) as a clear oil (71.5 mg, 95%).

R_f = 0.47 (20% ethyl acetate–hexanes; UV). ^{1}H NMR (500 MHz, CD_2Cl_2) δ 8.03 (d, J = 8.5 Hz, 2H, H_3), 6.96 (d, J = 8.5 Hz, 2H, H_2), 4.40 (s, 2H, H_4), 3.87 (s, 3H, H_1), 1.85 (s, 6H, H_5). ^{13}C NMR (125 MHz, CD_2Cl_2) δ 165.9 (C), 164.2 (C), 132.2 (CH), 122.7 (C), 114.3 (CH), 73.5 (CH_2), 62.3 (C), 56.1 (CH_3), 31.4 (CH_3). IR (ATR-FTIR), cm^{-1}: 2970 (w), 1713 (m), 1605 (m), 1252 (s), 1096 (s). HRMS-ESI (m/z): [M + H]$^+$ calcd for $C_{12}H_{16}{}^{79/81}BrO_3$, 287.0283/289.0262; found, 287.0280/289.0261.

Hydroiodination of 2-methylallyl 4-methoxybenzoate (1a) to 2-iodo-2-methylpropyl 4-methoxybenzoate (5b, Table S4, entry 7)

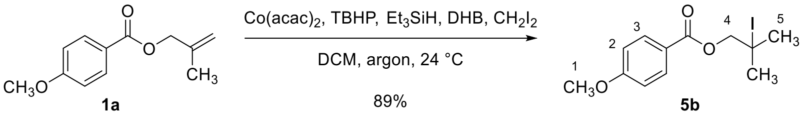

A 10 mL round-bottomed flask fitted with a rubber septum was charged sequentially with 2-methylallyl 4-methoxybenzoate (**1a**, 51.6 mg, 250 µmol, 1 equiv) and cobalt bis(acetylacetonate) (64.3 mg, 250 µmol, 1.00 equiv). The reaction vessel was evacuated and refilled using a balloon of argon. This process was repeated twice. The reaction vessel was protected from light with aluminum foil. Dichoromethane (830 µL), diiodomethane (302µL, 3.75 mmol, 15.0 equiv), 1,4-dihydrobenzene (115 µL, 1.25 mmol, 5.00 equiv), a solution of *tert*-butyl hydroperoxide in nonane (~5.5 M, 45.5 µL, 250 µmol, 1.00 equiv), and triethylsilane (400 µL, 2.50 mmol, 10.0 equiv) were added sequentially to the reaction vessel via syringe. The reaction mixture was stirred for 50 h at 24 °C. The product mixture was concentrated and the residue obtained was purified by flash-column chromatography (eluting with 2% ethyl acetate–hexanes initially, grading to 7% ethyl acetate–hexanes, linear gradient) to 2-iodo-2-methylpropyl 4-methoxybenzoate (**5b**) as a clear oil (74.3 mg, 89%).

R_f = 0.47 (20% ethyl acetate–hexanes; UV). ^{1}H NMR (400 MHz, CD_2Cl_2) δ 8.04 (d, J = 8.8 Hz, 2H, H_3), 6.96 (d, J = 8.8 Hz, 2H, H_2), 4.32 (s, 2H, H_4), 3.87 (s, 3H, H_1), 2.01 (s, 6H, H_5). ^{13}C NMR (100 MHz, CD_2Cl_2) δ 165.8 (C), 164.3 (C), 132.2 (CH), 122.7 (C), 114.3 (CH), 76.1 (CH_2), 56.1 (CH_3), 43.5 (C), 34.8 (CH_3). IR (ATR-FTIR), cm^{-1}: 2963 (w), 1714 (m), 1605 (m), 1254 (s), 1099 (s). HRMS-ESI (m/z): $[M + H]^+$ calcd for $C_{12}H_{16}IO_3$,335.0144; found, 335.0148.

Hydroiodination of 2-methylallyl 4-methoxybenzoate (1a) to 2-iodo-2-methylpropyl 4-methoxybenzoate (5b, Table S4, entry 9)

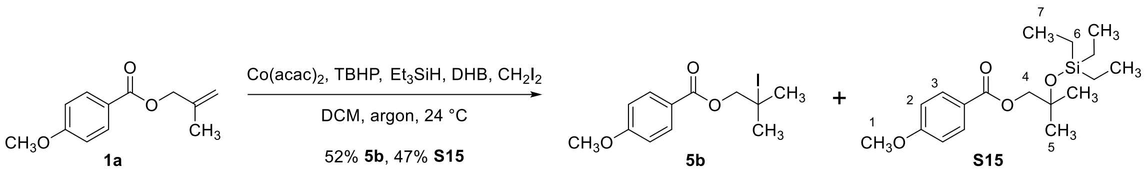

A 10 mL round-bottomed flask fitted with a rubber septum was charged sequentially with 2-methylallyl 4-methoxybenzoate (**1a**, 51.6 mg, 250 µmol, 1 equiv) and cobalt bis(acetylacetonate) (64.3 mg, 250 µmol, 1.00 equiv). The reaction vessel was evacuated and refilled using a balloon of argon. This process was repeated twice. The reaction vessel was protected from light with aluminum foil. Dichoromethane (830 µL), diiodomethane (156µL, 1.88 mmol, 7.50 equiv), 1,4-dihydrobenzene (115 µL, 1.25 mmol, 5.00 equiv), a solution of *tert*-butyl hydroperoxide in nonane (~5.5 M, 45.5 µL, 250 µmol, 1.00 equiv), and triethylsilane (400 µL, 2.50 mmol, 10.0 equiv) were added sequentially to the reaction vessel via syringe. The reaction mixture was stirred for 50 h at 24 °C. The product mixture was concentrated and the residue obtained was purified by flash-column chromatography (eluting with hexanes initially, grading to 7% ethyl acetate–hexanes, linear gradient) to 2-iodo-2-methylpropyl 4-methoxybenzoate (**5b**) as a clear oil (43.4 mg, 52%) and 2-methyl-2-((triethylsilyl)oxy)propyl 4-methoxybenzoate (**S15**) as a clear oil (39.7 mg, 47%).

2-Methyl-2-((triethylsilyl)oxy)propyl 4-methoxybenzoate (**S15**) R_f = 0.57 (20% ethyl acetate–hexanes; UV). ^{1}H NMR (400 MHz, CD_2Cl_2) δ 8.00 (d, J = 8.8 Hz, 2H, H_3), 6.94 (d, J = 8.8 Hz, 2H, H_2), 4.30 (s, 2H, H_4), 3.86 (s, 3H, H_1), 1.30 (s, 6H, H_5), 0.97 (t, J = 8.0 Jz, 9H, H_7), 0.68 (q, J = 8.0 Hz,6H, H_6). ^{13}C NMR (100 MHz, CD_2Cl_2) δ 166.4 (C), 164.0 (C), 132.0 (CH), 123.4 (C), 114.1 (CH), 81.7 (C), 67.9 (CH_2), 56.0 (CH_3), 22.3 (CH_3), 7.1 (CH_3), 4.3 (CH_2). IR (ATR-FTIR), cm^{-1}: 2955(w), 1714 (m), 1275 (m), 1253 (s), 1101 (s). HRMS-ESI (m/z): $[M + H]^+$ calcd for $C_{18}H_{31}SiO_4$, 339.1992; found, 339.1989.

General procedure for the hydroselenation of 2-methylallyl 4-methoxybenzoate (1a) to 2-methyl-2-(phenylselanyl)propyl 4-methoxybenzoate (5c, Table S4, entry 11)

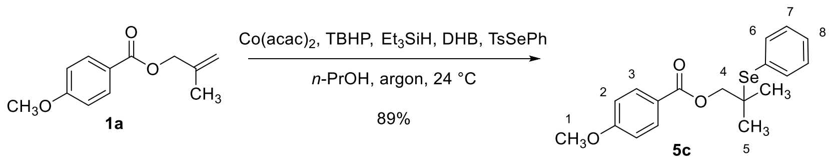

A 10 mL round-bottomed flask fitted with a rubber septum was charged sequentially with 2-methylallyl 4-methoxybenzoate (**1a**, 51.6 mg, 250 µmol, 1 equiv), *Se*-phenyl 4-methylbenzenesulfonoselenoate (195 mg, 625 µmol, 2.50 equiv), and cobalt bis(acetylacetonate) (64.3 mg, 250 µmol, 1.00 equiv). The reaction vessel was evacuated and refilled using a balloon of argon. This process was repeated twice. *n*-Propanol (830 µL), 1,4-dihydrobenzene (86.0 µL, 938 µmol, 3.75 equiv), a solution of *tert*-butyl hydroperoxide in nonane (~5.5 M, 45.5 µL, 250 µmol, 1.00 equiv), and triethylsilane (400 µL, 2.50 mmol, 10.0 equiv) were added sequentially to the reaction vessel via syringe. The reaction mixture was stirred for 4.0 h at 24 °C. The product mixture was filtered through a short column of silica gel and the short column was rinsed with 20% ethyl acetate–hexanes (100 mL). The filtrates were combined and the combined filtrates were concentrated. The residue obtained was purified by flash-column chromatography (eluting with 2% ethyl acetate–hexanes initially, grading to 6% ethyl acetate–hexanes, linear gradient) to 2-methyl-2-(phenylselanyl)propyl 4-methoxybenzoate (**5c**) as a clear oil (83.9 mg, 89%).

R_f = 0.47 (20% ethyl acetate–hexanes; UV, CAM). ^{1}H NMR (400 MHz, CD_2Cl_2) δ 7.98–7.95 (m, 2H, H_3), 7.69–7.66 (m, 2H, H_7), 7.42–7.38 (m, 1H, H_8), 7.34–7.30 (m, 2H, H_6), 6.95–6.92 (m, 2H, H_2), 4.24 (s, 2H, H_4), 3.86 (s, 3H, H_1), 1.46 (s, 6H, H_5). ^{13}C NMR (100 MHz, CD_2Cl_2) δ 166.2 (C), 164.1 (C), 138.9 (CH), 132.1 (CH), 129.4 (CH), 129.4 (CH), 127.4 (C), 123.1 (C), 114.2 (CH), 72.7 (CH_2), 56.0 (CH_3), 45.0 (C), 27.0 (CH_3). IR (ATR-FTIR), cm^{-1}: 2960 (w), 1712 (m), 1606 (m), 1256 (s), 1167 (m). HRMS-ESI (m/z): $[M + H]^+$ calcd for $C_{18}H_{21}SeO_3$, 365.0656; found, 365.0648.

2-Bromopropyl 4-methoxybenzoate (6a)

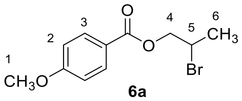

6a

Following the general hydrobromination procedure using allyl 4-methoxybenzoate (**1c**, 48.1 mg, 250μmol, 1 equiv). Reaction time was 4.0 h. Purification by flash-column chromatography (eluting with 2% ethyl acetate–hexanes initially, grading to 7% ethyl acetate–hexanes, linear gradient) afforded 2-bromoropropyl 4-methoxybenzoate (**6a**) as a clear oil (60.1 mg, 88%).

R_f = 0.48 (20% ethyl acetate–hexanes; UV). ^{1}H NMR (500 MHz, CD_2Cl_2) δ 8.01 (d, J = 9.0 Hz, 2H, H_3), 6.95 (d, J = 9.0 Hz, 2H, H_2), 4.49–4.35 (m, 3H, 2 × H_4, 1 × H_5), 3.86 (s, 3H, H_1), 1.77 (d, J = 6.5 Hz, 3H, H_6). ^{13}C NMR (125 MHz, CD_2Cl_2) δ 166.0 (C), 164.3 (C), 132.2 (CH), 122.7 (C), 114.3 (CH), 69.5 (CH_2), 56.1 (CH_3), 46.1 (CH), 23.0 (CH_3). IR (ATR-FTIR), cm^{-1}: 2931 (w), 1714 (m), 1605 (m), 1254 (s), 1167 (s). HRMS-ESI (m/z): $[M + Na]^+$ calcd for $C_{11}H_{13}{}^{79/81}BrNaO_3$, 294.9946/296.9925; found, 294.9944/296.9909.

2-Iodopropyl 4-methoxybenzoate (6b)

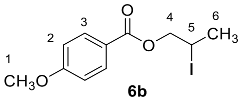

6b

Following the general hydroiodination procedure using allyl 4-methoxybenzoate (**1c,** 48.1 mg, 250μmol, 1 equiv). Reaction time was 168 h. The product mixture was filtered through a short column of silica gel and the short column was rinsed with 20% ethyl acetate–hexanes (100 mL). The filtrates were combined and the combined filtrates were concentrated. ^{1}H NMR analysis of the unpurified product mixture using mesitylene as an internal standard revealed a 69% yield of allyl 4-methoxybenzoate (**9c**) and a 23% yield of 2-iodopropyl 4-methoxybenzoate (**6b**). An inseparable 1.2:1.0 mixture of **1c** versus **6b** was obtained for characterization by flash-column chromatography (eluting with 2% ethyl acetate–hexanes initially, grading to 6% ethyl acetate–hexanes, linear gradient).

R_f = 0.55 (20% ethyl acetate–hexanes; UV). ^{1}H NMR (500 MHz, C_6D_6) δ 8.18–8.15 (m, 2H, H_3), 6.64–6.62 (m, 2H, H_2), 4.24–4.22 (m, 1H, 1 × H_4), 4.12–4.08 (m, 1H, 1 × H_4), 3.88–3.84(m, 1H, H_5), 3.15–3.13 (m, 3H, H_1), 1.45 (d, J = 7.0 Hz, 3H, H_6). ^{13}C NMR (125 MHz, C_6D_6) δ 165.3 (C), 164.9 (C), 131.8 (CH), 122.4 (C), 113.7 (CH), 70.2 (CH_2), 54,5 (CH_3), 24.1 (CH_3), 21.8 (CH). IR (ATR-FTIR), cm^{-1}: 2958 (w), 1713 (m), 1606 (m), 1254 (s), 1167 (s). HRMS-ESI (m/z): $[M + H]^+$ calcd for $C_{11}H_{13}INaO_3$, 342.9807; found, 342.9805.

2-(Phenylselanyl)propyl 4-methoxybenzoate (6c)

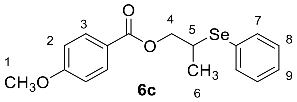

6c

Following the general hydroselenation procedure using allyl 4-methoxybenzoate (**1c**, 48.1 mg, 250μmol, 1 equiv). Reaction time was 4.0 h. Purification by flash-column chromatography (eluting with 2% ethyl acetate–hexanes initially, grading to 7% ethyl acetate–hexanes, linear gradient) afforded 2-(phenylselanyl)propyl 4-methoxybenzoate (**6c**) as a clear oil (75.9 mg, 87%).

R_f = 0.48 (20% ethyl acetate–hexanes; UV, CAM). ^{1}H NMR (400 MHz, CD_2Cl_2) δ 7.92 (d, J = 8.8 Hz, 2H, H_3), 7.63–7.60 (m, 2H, H_8), 7.31–7.29 (m, 3H, 2 × H_7, 1 × H_9), 6.91 (d, J = 8.8 Hz, 2H, H_2), 4.46–4.41 (m, 1H, 1 × H_4), 4.31–4.26 (m, 1H, 1 × H_4), 3.85 (s, 3H, H_1), 3.67–3.57 (m, 1H, H_5), 1.50 (d, J = 6.8 Hz, 3H, H_6). ^{13}C NMR (10 MHz, CD_2Cl_2) δ 166.2 (C), 164.0 (C), 135.5 (CH), 132.0 (CH), 129.6 (CH), 128.8 (C), 128.3 (CH), 123.0 (C), 114.1 (CH), 69.3 (CH_2), 56.0 (CH_3), 37.3 (CH), 19.0 (CH_3). IR (ATR-FTIR), cm^{-1}: 2963 (w), 1709 (m), 1605 (m), 1510 (w), 1253 (s). HRMS-ESI (m/z): $[M + H]^+$ calcd for $C_{17}H_{18}SeO_3$, 351.0499; found, 351.0513.

3-Bromo-3-methylbutyl 4-methoxybenzoate (7a)

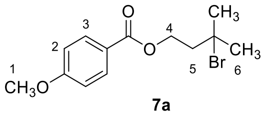

7a

Following the general hydrobromination procedure using 3-methylbut-2-en-1-yl 4-methoxybenzoate (**1j**, 55.0 mg, 250μmol, 1 equiv). Reaction time was 4.0 h. Purification by flash-column chromatography (eluting with 2% ethyl acetate–hexanes initially, grading to 7% ethyl acetate–hexanes, linear gradient) afforded 3-bomo-3-methylbutyl 4-methoxybenzoate (**7a**) as a clear oil (63.7 mg, 88%).

R_f = 0.46 (20% ethyl acetate–hexanes; UV). ^{1}H NMR (500 MHz, CD_2Cl_2) δ 7.98 (d, J = 9.0 Hz, 2H, H_3), 6.94 (d, J = 9.0 Hz, 2H, H_2), 4.52 (t, J = 6.8 Hz, 2H, H_4), 3.85 (s, 3H, H_1), 2.31 (t, J = 6.8 Hz, 2H, H_5), 1.77 (s, 6H, H_6). ^{13}C NMR (125 MHz, CD_2Cl_2) δ 166.5 (C), 164.0 (C), 132.0 (CH), 123.2 (C), 114.2 (CH), 65.6 (C), 63.3 (CH_2), 56.0 (CH_3), 46.0 (CH_2), 35.1 (CH_3). IR (ATR-FTIR), cm^{-1}: 2965 (w), 1710 (m), 1605 (m), 1253 (s), 1166 (m). HRMS-ESI (m/z): $[M + Na]^+$ calcd for $C_{13}H_{17}{}^{79/81}BrNaO_3$, 323.0259/325.0238; found, 323.0266/325.0243.

3-iodo-3-methylbutyl 4-methoxybenzoate (7b)

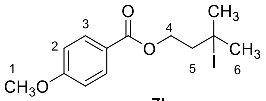

7b

Following the general hydroiodination procedure using 3-methylbut-2-en-1-yl 4-methoxybenzoate (**1j**, 55.0 mg, 250μmol, 1 equiv). Reaction time was 125 h. The product mixture was filtered through a short column of silica gel and the short column was rinsed with 20% ethyl acetate–hexanes (100 mL). The filtrates were combined and the combined filtrates were concentrated. ^{1}H NMR analysis of the unpurified product mixture using mesitylene as an internal standard revealed a 58% yield of 3-methylbut-2-en-1-yl 4-methoxybenzoate (**1j**) and a 29% yield of 3-iodo-3-methylbutyl 4-methoxybenzoatee (**7b**). An analytically pure sample of **7b** was obtained for characterization by flash-column chromatography (eluting with 2% ethyl acetate–hexanes initially, grading to 5% ethyl acetate–hexanes, linear gradient).

R_f = 0.49 (20% ethyl acetate–hexanes; UV). ^{1}H NMR (500 MHz, C_6D_6) δ 8.15 (d, J = 8.0 Hz, 2H, H_3), 6.67 (d, J = 8.0 Hz, 2H, H_2), 4.45 (t, J = 6.8 Hz, 2H, H_4), 3.16(m, 3H, H_1), 1.81 (t, J = 6.8 Hz, 2H, H_5), 1.63 (s, 6H, H_6). ^{13}C NMR (125 MHz, C_6D_6) δ 165.9 (C), 163.8 (C), 132.0 (CH), 123.4 (C), 114.0 (CH), 64.7 (CH_2), 54.9 (CH_3), 48.5 (CH_2), 46.2 (C), 38.3 (CH_3). IR (ATR-FTIR), cm^{-1}: 2962 (w), 1710 (m), 1605 (m), 1256 (s), 1167 (m). HRMS-ESI (m/z): $[M + Na]^+$ calcd for $C_{13}H_{17}INaO_3$, 371.0120; found, 371.0131.

3-Methyl-3-(phenylselanyl)butyl 4-methoxybenzoate (7c)

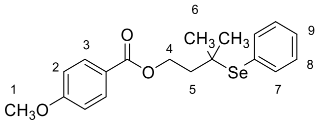

7c

Following the general hydroselenation procedure using 3-methylbut-2-en-1-yl 4-methoxybenzoate (**1j**, 55.0 mg, 250μmol, 1 equiv). Reaction time was 4.0 h. Purification by flash-column chromatography (eluting with 2% ethyl acetate–hexanes initially, grading to 7% ethyl acetate–hexanes, linear gradient) afforded 3-methyl-3-(phenylselanyl)butyl 4-methoxybenzoate (**7c**) as a clear oil (76.4 mg, 81%).

R_f = 0.48 (20% ethyl acetate–hexanes; UV, CAM). ^{1}H NMR (500 MHz, CD_2Cl_2) δ 7.98 (d, J = 7.5 Hz, 2H, H_3), 7.68–7.66 (m, 2H, H_8), 7.41–7.39 (m, 1H, H_9), 7.35–7.32 (m, 2H, H_7), 6.94 (d, J = 7.5 Hz, 2H, H_2), 4.51 (t, J = 6.8 Hz, 2H, H_4), 3.87 (s, 3H, H_1), 2.01 (t, J = 6.8 Hz, 2H, H_5), 1.47 (s, 6H, H_6). ^{13}C NMR (125MHz, CD_2Cl_2) δ 166.6 (C), 164.0 (C), 138.8 (CH), 132.0 (CH), 129.3 (CH), 129.2 (CH), 128.2 (C), 123.4 (C), 114.1 (CH), 63.1 (CH_2), 56.0 (CH_3), 45.7 (C), 42.1 (CH_2), 30.5 (CH_3). IR (ATR-FTIR), cm^{-1}: 2960 (w), 1708 (m), 1605 (m), 1511 (w), 1254 (s). HRMS-ESI (m/z): $[M + K]^+$ calcd for $C_{19}H_{22}SeKO_3$, 417.0371; found, 417.0366.

Hydrobromination of 2-chloroallyl 4-methoxybenzoate (1d) to 2-bromo-2-chloropropyl 4-methoxybenzoate (8a)

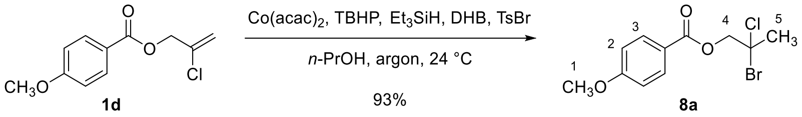

A 10 mL round-bottomed flask fitted with a rubber septum was charged sequentially with 2-chloroallyl 4-methoxybenzoate (**1d**, 28.3 mg, 125 µmol, 1 equiv), tosyl bromide (73.5 mg, 313 µmol, 2.50 equiv), and cobalt bis(acetylacetonate) (32.1 mg, 125 µmol, 1.00 equiv). The reaction vessel was evacuated and refilled using a balloon of argon. This process was repeated twice. *n*-Propanol (400 µL), 1,4-dihydrobenzene (43.0 µL, 469 µmol, 3.75 equiv), a solution of *tert*-butyl hydroperoxide in nonane (~5.5 M, 22.8 µL, 125 µmol, 1.00 equiv), and triethylsilane (200 µL, 1.25 mmol, 10.0 equiv) were added sequentially to the reaction vessel via syringe. The reaction mixture was stirred for 3.0 h at 24 °C. The product mixture was filtered through a short column of silica gel and the short column was rinsed with 20% ethyl acetate–hexanes (100 mL). The filtrates were combined and the combined filtrates were concentrated. The residue obtained was purified by flash-column chromatography (eluting with 2% ethyl acetate–hexanes initially, grading to 5% ethyl acetate–hexanes, linear gradient) to 2-bromo-2-chloropropyl 4-methoxybenzoate (**8a**) as a clear oil (35.6 mg, 93%).

R_f = 0.53 (20% ethyl acetate–hexanes; UV). ^{1}H NMR (500 MHz, CD_2Cl_2) δ 8.05 (d, J = 8.5 Hz, 2H, H_3), 6.97 (d, J = 8.5 Hz, 2H, H_2), 4.73–4.68 (m, 2H, H_4), 3.87 (s, 3H, H_1), 2.38 (s, 3H, H_5). ^{13}C NMR (150 MHz, CD_2Cl_2) δ 165.4 (C), 164.6 (C), 132.4 (CH), 122.1 (C), 114.4 (CH), 74.9 (C), 73.8 (CH_2), 56.1 (CH_3), 36.2 (CH_3). IR (ATR-FTIR), cm^{-1}: 2931 (w), 1721 (m), 1606 (m), 1255 (m), 557 (s). HRMS-ESI (m/z): [M + Na]$^+$ calcd for $C_{11}H_{12}{}^{35/37}Cl^{79/81}BrNaO_3$, 328.9556/330.9536/330.9527/332.9506; found, 328.9560/330.9528/330.9518/332.9510.

Hydrobromination of 2-bromoallyl 4-methoxybenzoate (1k) to 2,2-dibromopropyl 4-methoxybenzoate (8b)

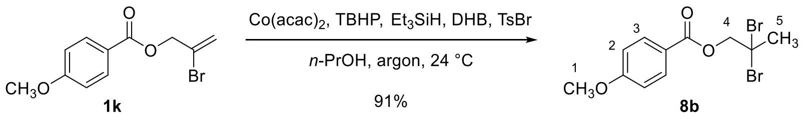

A 10 mL round-bottomed flask fitted with a rubber septum was charged sequentially with 2-bromoallyl 4-methoxybenzoate (**1k**, 33.9 mg, 125 μmol, 1 equiv), tosyl bromide (147 mg, 625 μmol, 2.50 equiv), and cobalt bis(acetylacetonate) (64.3 mg, 250 μmol, 1.00 equiv). The reaction vessel was evacuated and refilled using a balloon of argon. This process was repeated twice. *n*-Propanol (830 μL), 1,4-dihydrobenzene (86.0 μL, 938 μmol, 3.75 equiv), a solution of *tert*-butyl hydroperoxide in nonane (~5.5 M, 45.5 μL, 250 μmol, 1.00 equiv), and triethylsilane (400 μL, 2.50 mmol, 10.0 equiv) were added sequentially to the reaction vessel via syringe. The reaction mixture was stirred for 5.0 h at 24 °C. The product mixture was filtered through a short column of silica gel and the short column was rinsed with 20% ethyl acetate–hexanes (100 mL). The filtrates were combined and the combined filtrates were concentrated. The residue obtained was purified by flash-column chromatography (eluting with 2% ethyl acetate–hexanes initially, grading to 5% ethyl acetate–hexanes, linear gradient) to 2,2-dibromopropyl 4-methoxybenzoate (**8b**) as a clear oil (39.9 mg, 93%).

R_f = 0.51 (20% ethyl acetate–hexanes; UV). ^{1}H NMR (500 MHz, CD_2Cl_2) δ 8.05 (d, J = 8.5 Hz, 2H, H_3), 6.97 (d, J = 8.5 Hz, 2H, H_2), 4.75 (s, 2H, H_4), 3.87 (s, 3H, H_1), 2.56 (s, 3H, H_5). ^{13}C NMR (125 MHz, CD_2Cl_2) δ 165.3 (C), 164.5 (C), 132.4 (CH), 122.1 (C), 114.4 (CH), 74.7 (CH_2), 61.7 (C), 56.1 (CH_3), 37.8 (CH_3). IR (ATR-FTIR), cm^{-1}: 2933 (w), 1719 (m), 1605 (m), 1254 (m), 1091 (m). HRMS-ESI (m/z): [M + H]$^+$ calcd for $C_{11}H_{12}{}^{79/81}Br_2O_3$, 350.9231/352.9211/354.9191; found, 350.9240/352.9212/354.9185.

Intramolecular Competition Experiment (Scheme 2):

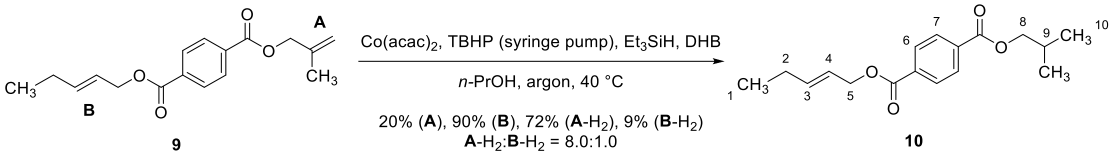

Following the general procedure B using (*E*)-2-methylallyl pent-2-en-1-yl terephthalate (**9**, 72.0 mg, 250 μmol, 1 equiv). Reaction time was 40 min and 0.667 equiv of TBHP were employed. ^{1}H NMR analysis of the unpurified product mixture using mesitylene as an internal standard (28.2 mg, 235 μmol, 0.940 equiv) revealed a 20% yield of alkene (A), a 90% yield of alkene (B), a 72% yield of alkene (A) hydrogenation, and a 9% yield of alkene (B) hydrogenation. An analytically pure sample of (*E*)-isobutyl pent-2-en-1-yl terephthalate (**10**) was obtained by automated flash-column chromatography (eluting with 2% ether–hexanes initially, grading to 10% ether–hexanes, linear gradient) followed by five iterations of preparative thin-layer chromatography on silver nitrate impregnated silica gel plates.

R_f = 0.47 (10% ethyl acetate–hexanes; UV, $KMnO_4$). ^{1}H NMR (500 MHz, $CDCl_3$) δ 8.13–8.08 (m, 4H, 2 × H_6, 2 × H_7), 5.95–5.89 (m, 1H, H_3), 5.71–5.65 (m, 1H, H_4), 4.79 (d, 2H, J = 5 Hz, H_5), 4.12 (d, 2H, J = 5 Hz, H_8), 2.14–2.06 (m, 3H, 2 × H_2, 1 × H_9), 1.04–1.01 (m, 9H, 3 × H_1, 9 × H_{10}). ^{13}C NMR (125 MHz, $CDCl_3$) δ 165.8 (C), 165.6 (C),138.5 (CH), 134.2 (C), 134.1 (C), 129.6 (CH), 129.4 (CH), 122.5 (CH), 71.4 (CH_2), 66.2 (CH_2), 27.9 (CH), 25.2 (CH_2), 19.2 (CH_3), 13.1 (CH_3). IR (ATR-FTIR), cm^{-1}: 2964 (w), 1716 (S), 1263 (s), 1244 (s), 1115 (s). HRMS-ESI (m/z): $[M + H]^+$ calcd for $C_{17}H_{23}O_4$, 291.1596; found, 291.1587.

Catalog of Nuclear Magnetic Resonance and Infrared Spectra.

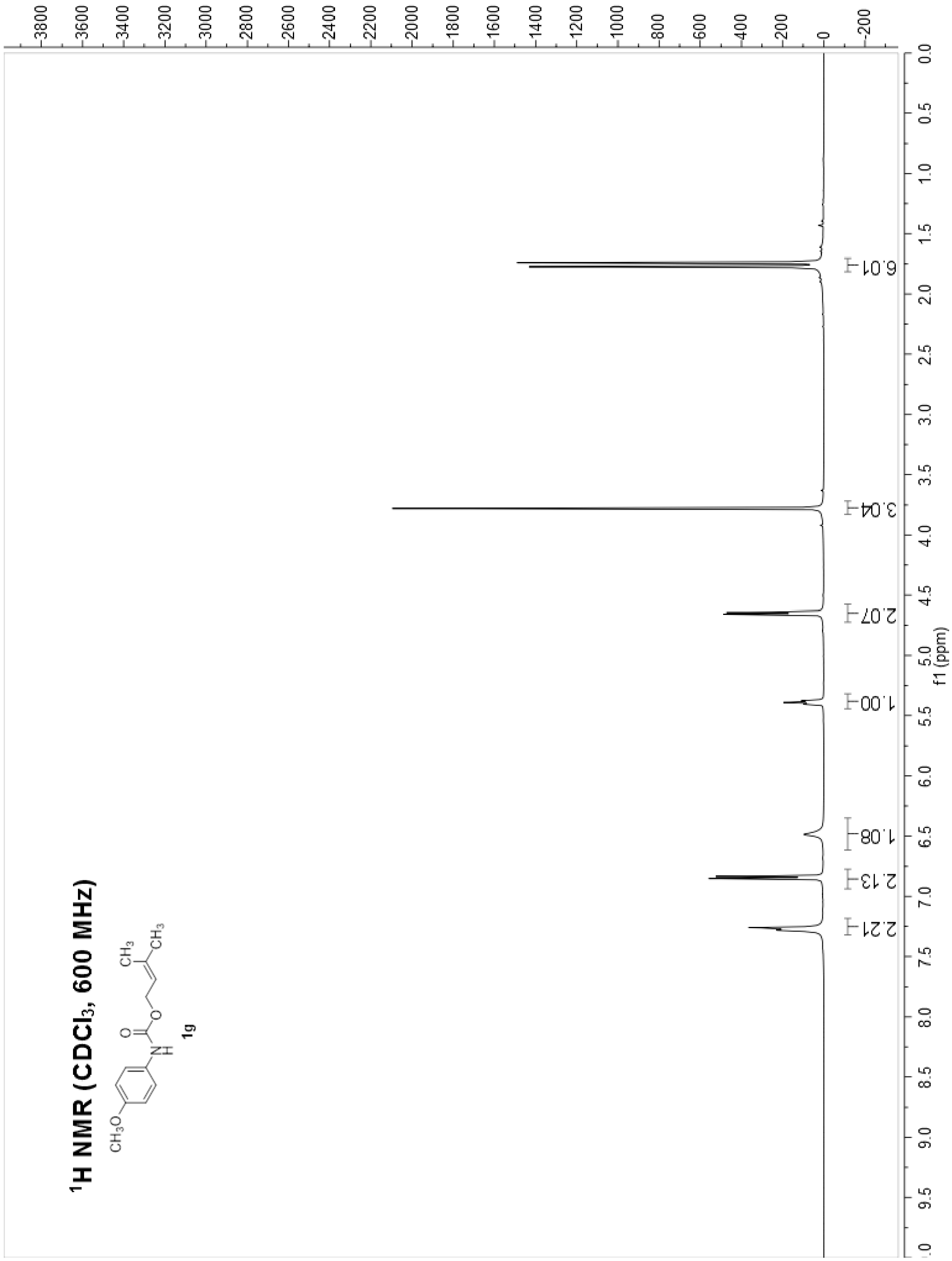

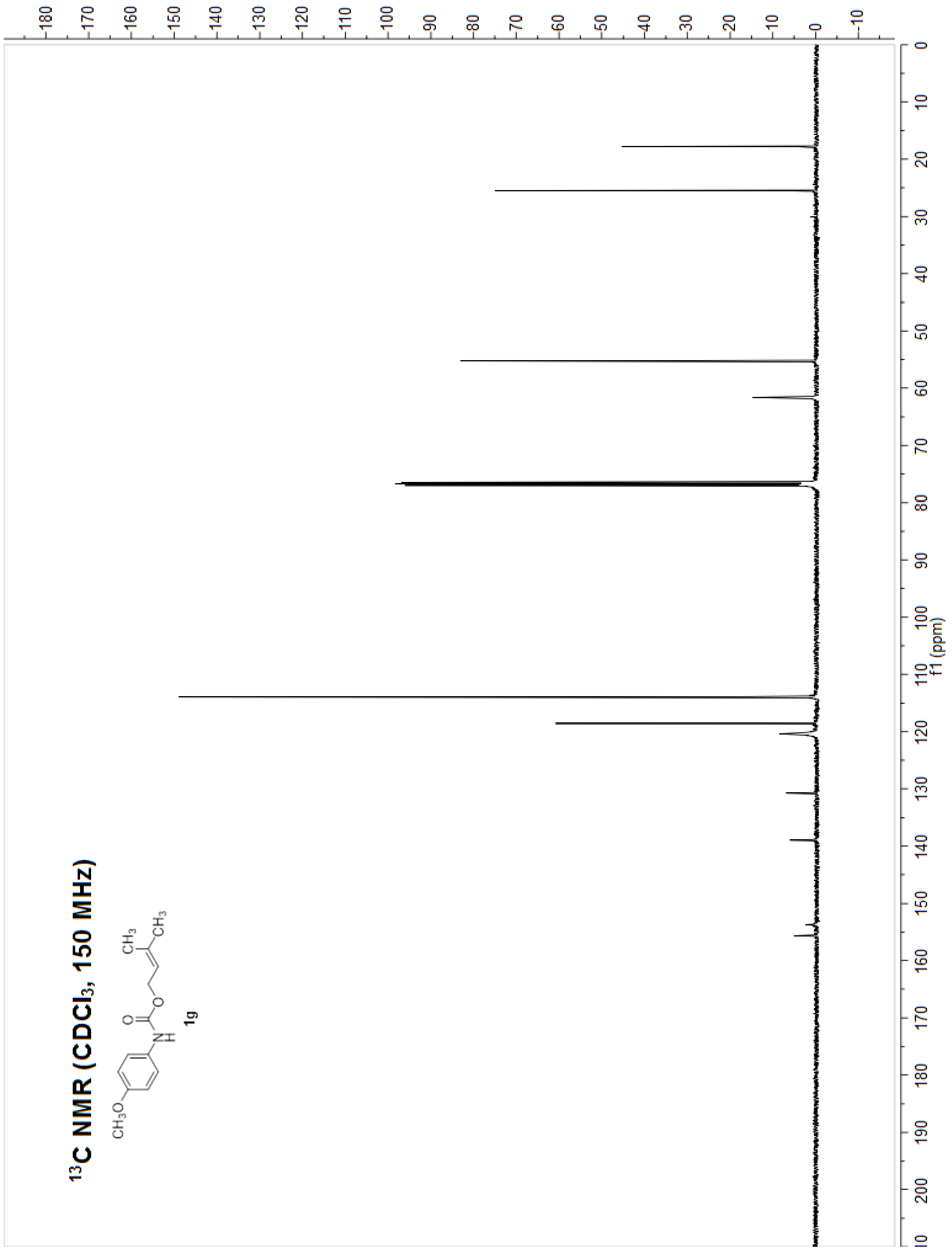
13C NMR (CDCl3, 150 MHz)
CH3O
N
H
O
O
CH3
CH3
1g
f1 (ppm)

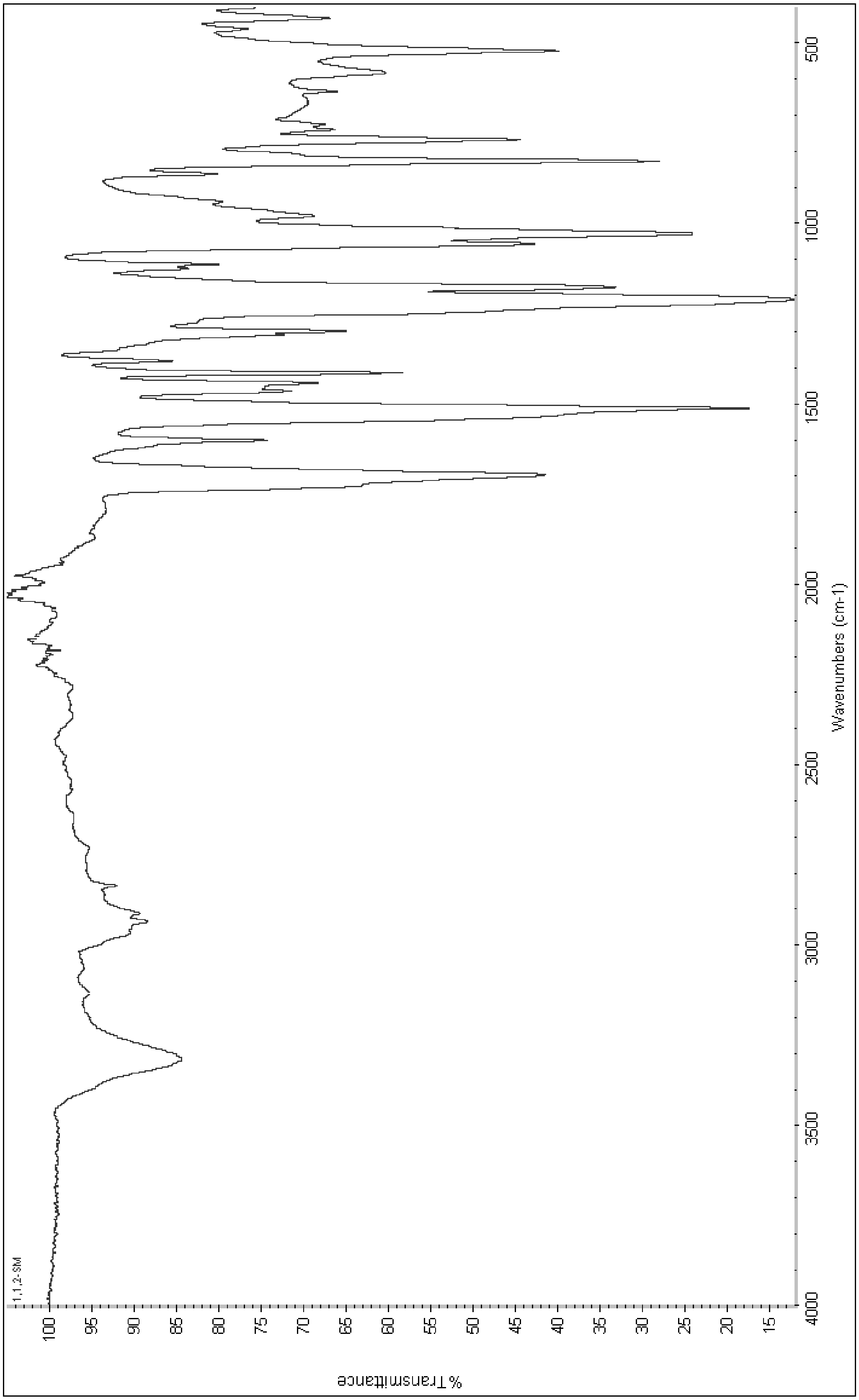
1,1,2-SM
%Transmittance
Wavenumbers (cm-1)

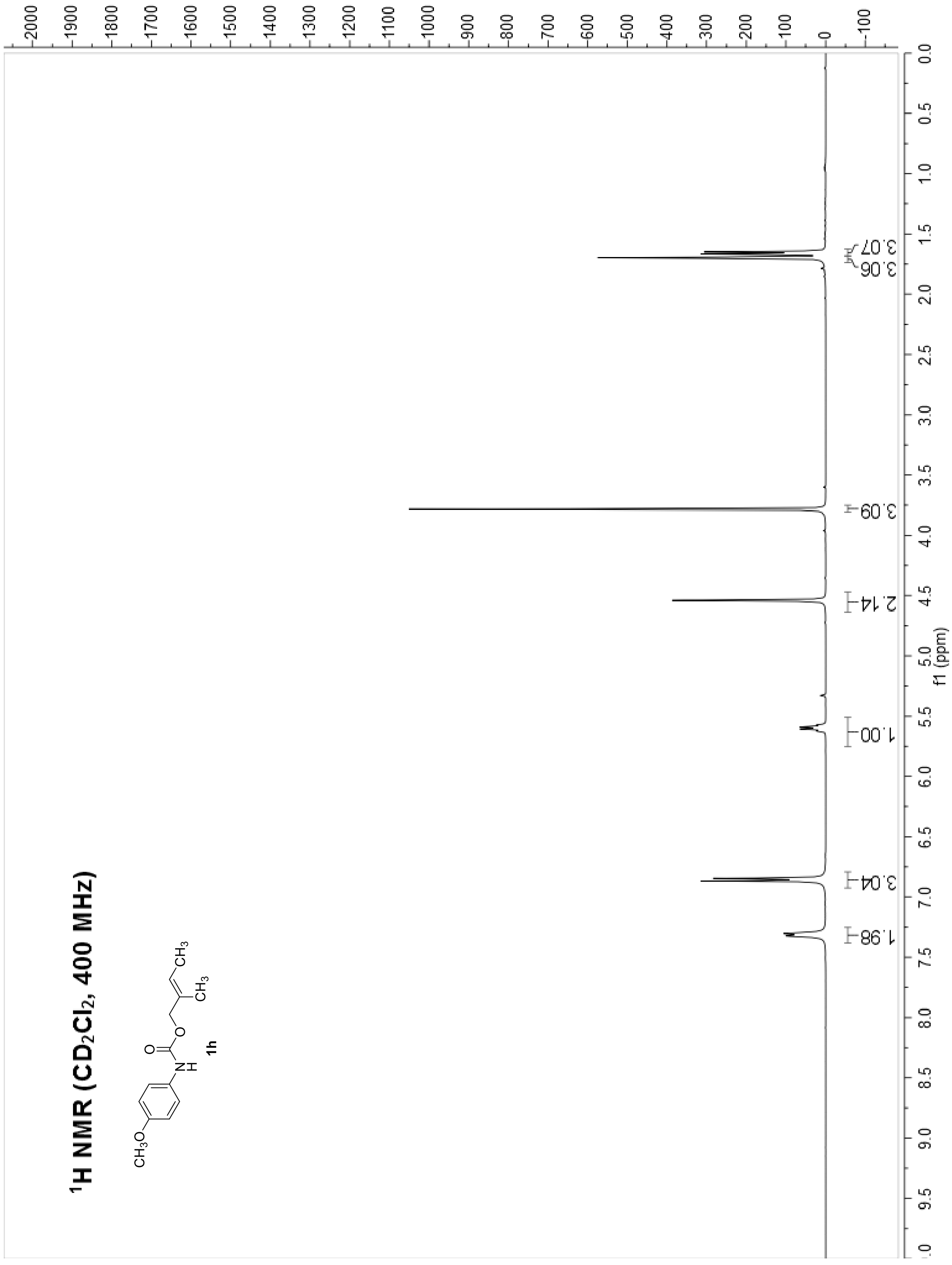
1H NMR (CD2Cl2, 400 MHz)
1h
f1 (ppm)
3.07
3.06
3.09
2.14
1.00
3.04
1.98

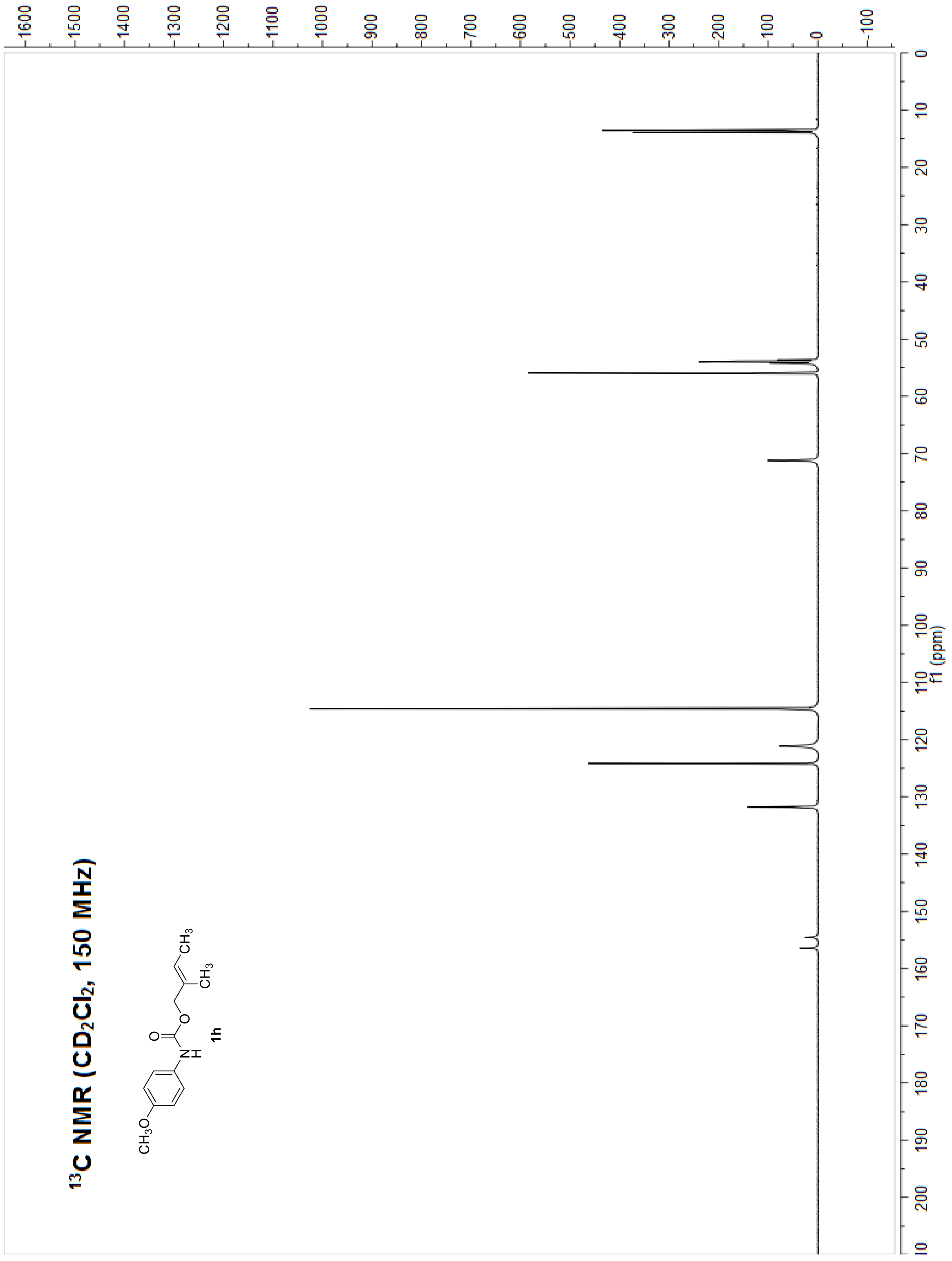
13C NMR (CD2Cl2, 150 MHz)
1h
f1 (ppm)

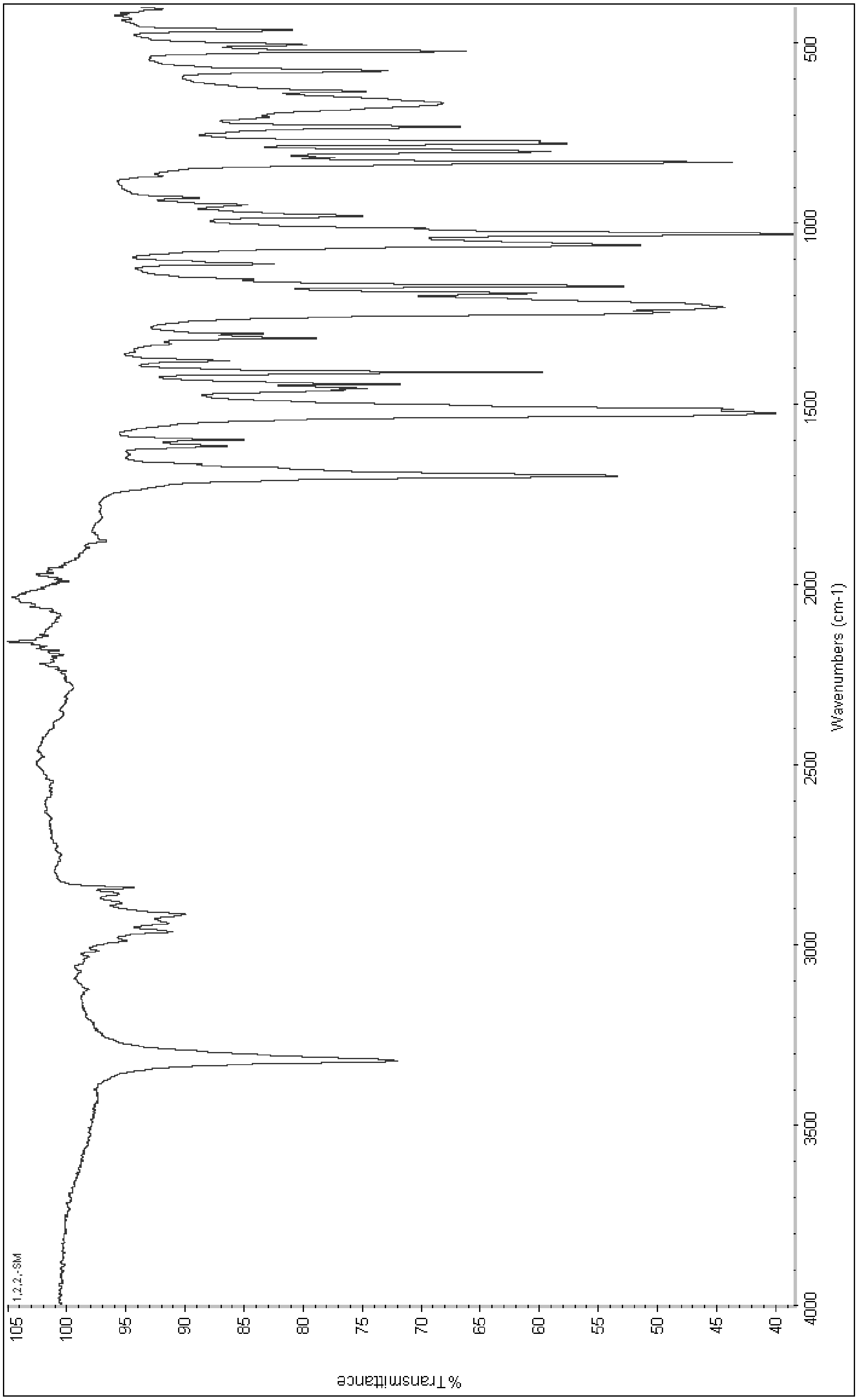
%Transmittance
Wavenumbers (cm-1)

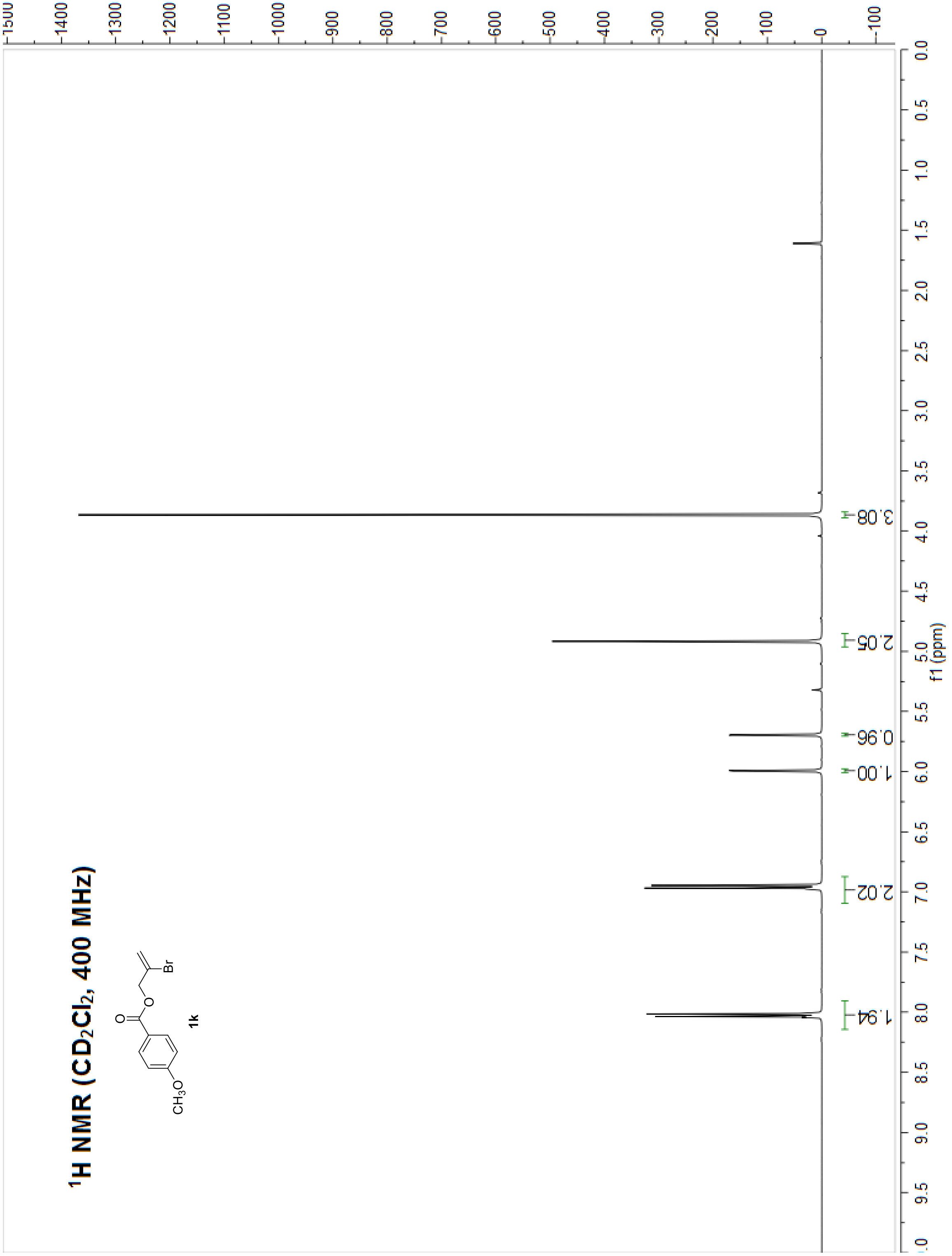
1H NMR (CD2Cl2, 400 MHz)
1k
f1 (ppm)

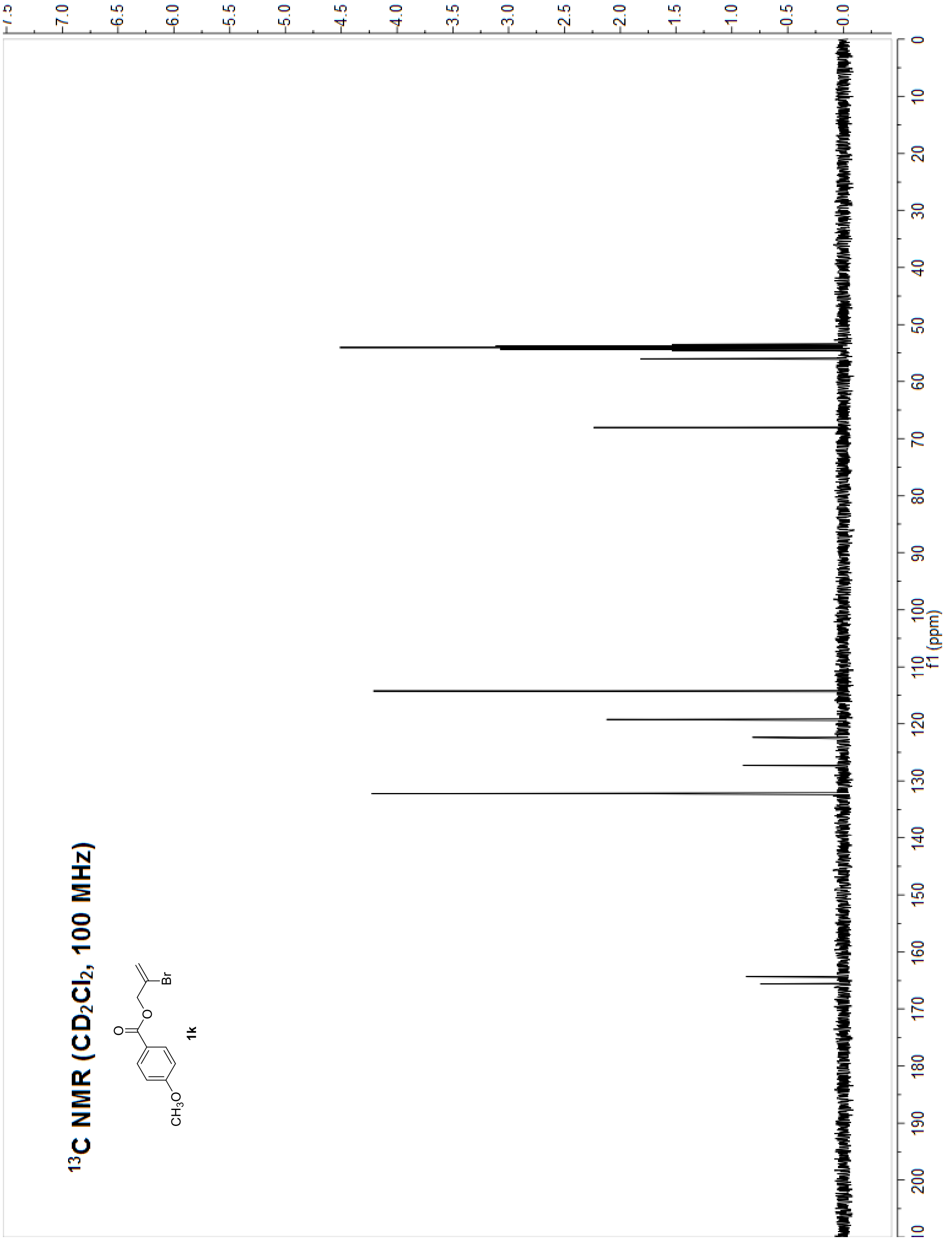
13C NMR (CD2Cl2, 100 MHz)
Br
O
O
CH3O
1k
f1 (ppm)

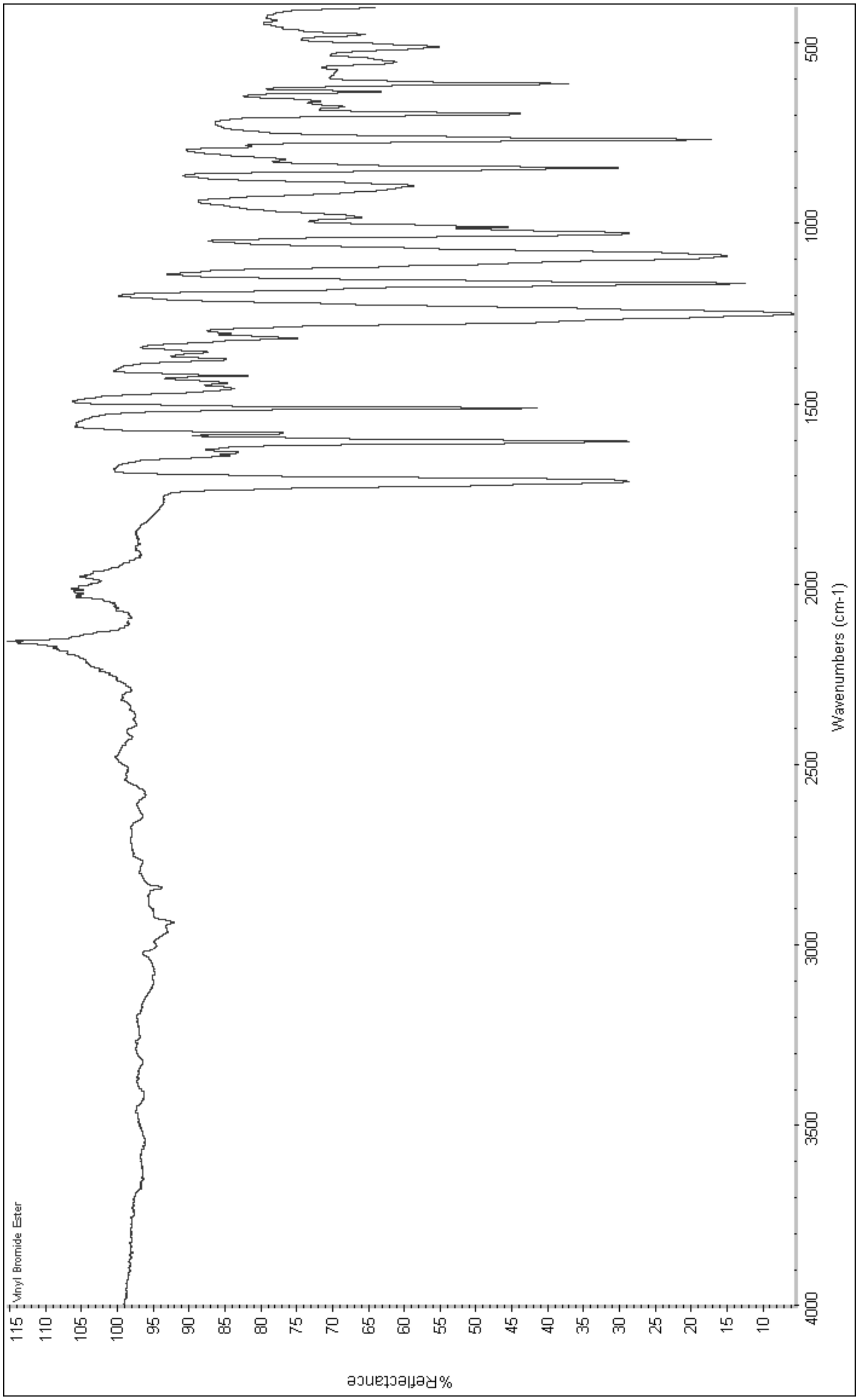
Vinyl Bromide Ester
%Reflectance
Wavenumbers (cm-1)

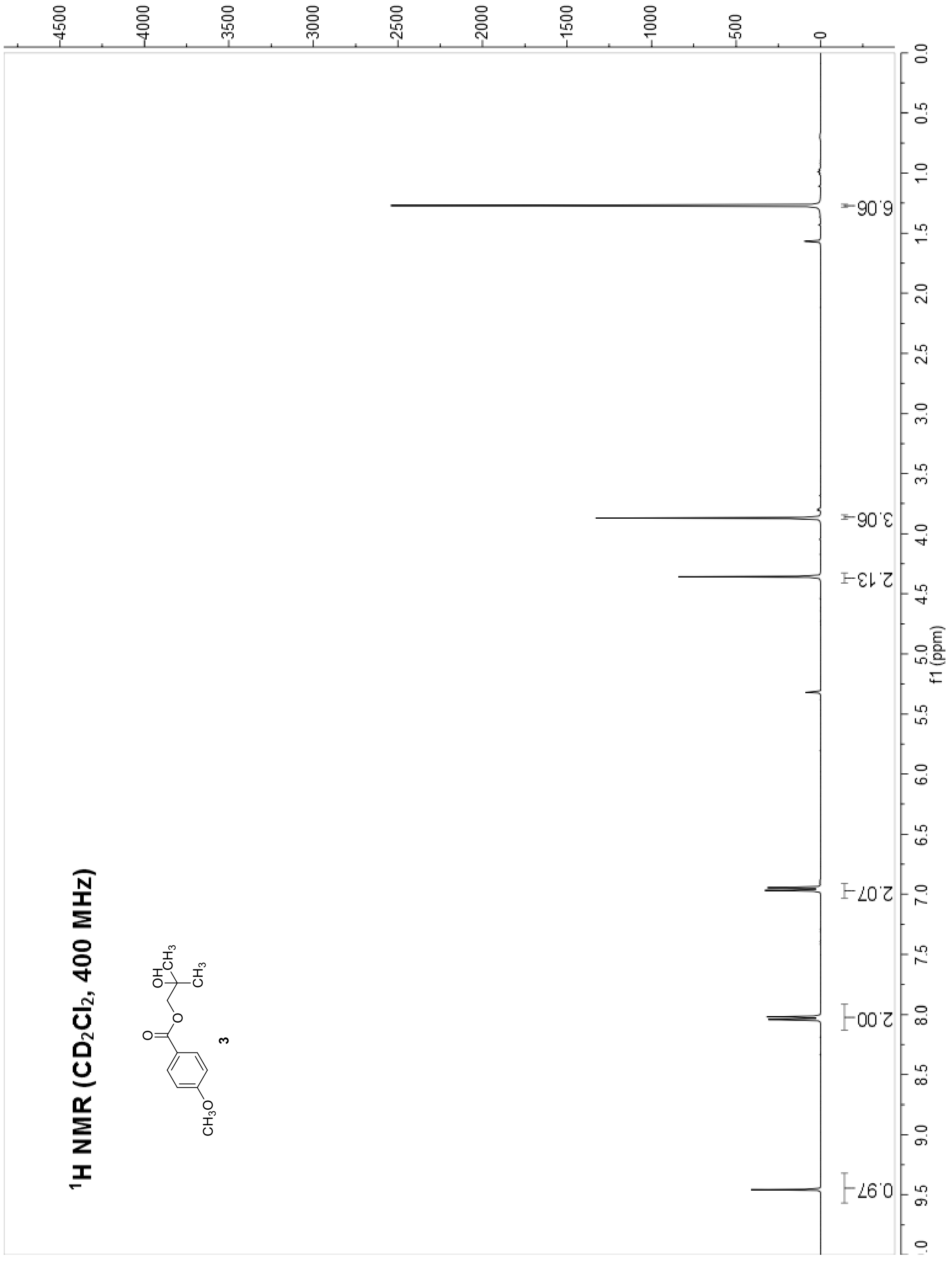
¹H NMR (CD₂Cl₂, 400 MHz)
3
f1 (ppm)
6.06
3.06
2.13
2.07
2.00
0.97

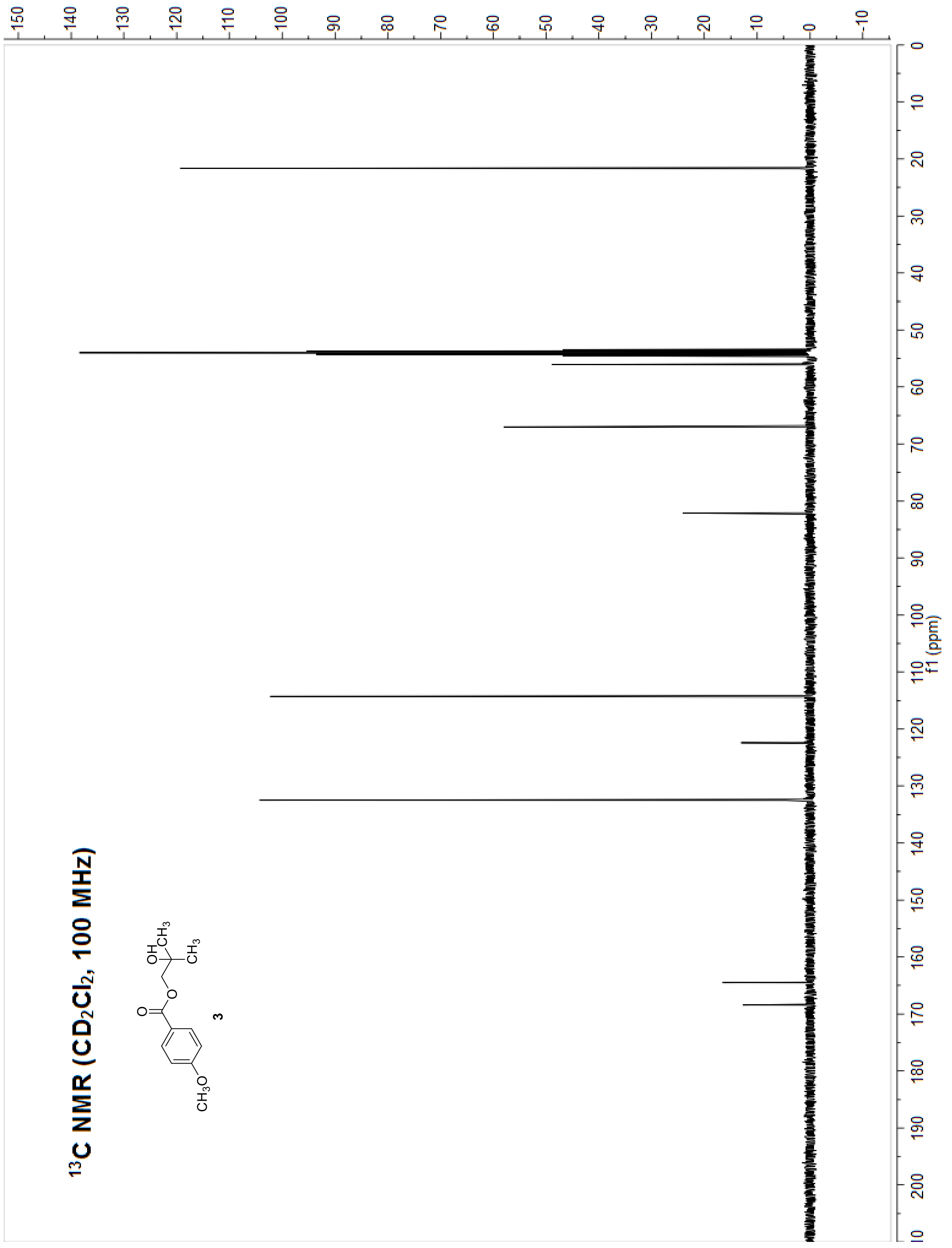
^{13}C NMR (CD_2Cl_2, 100 MHz)
3
f1 (ppm)

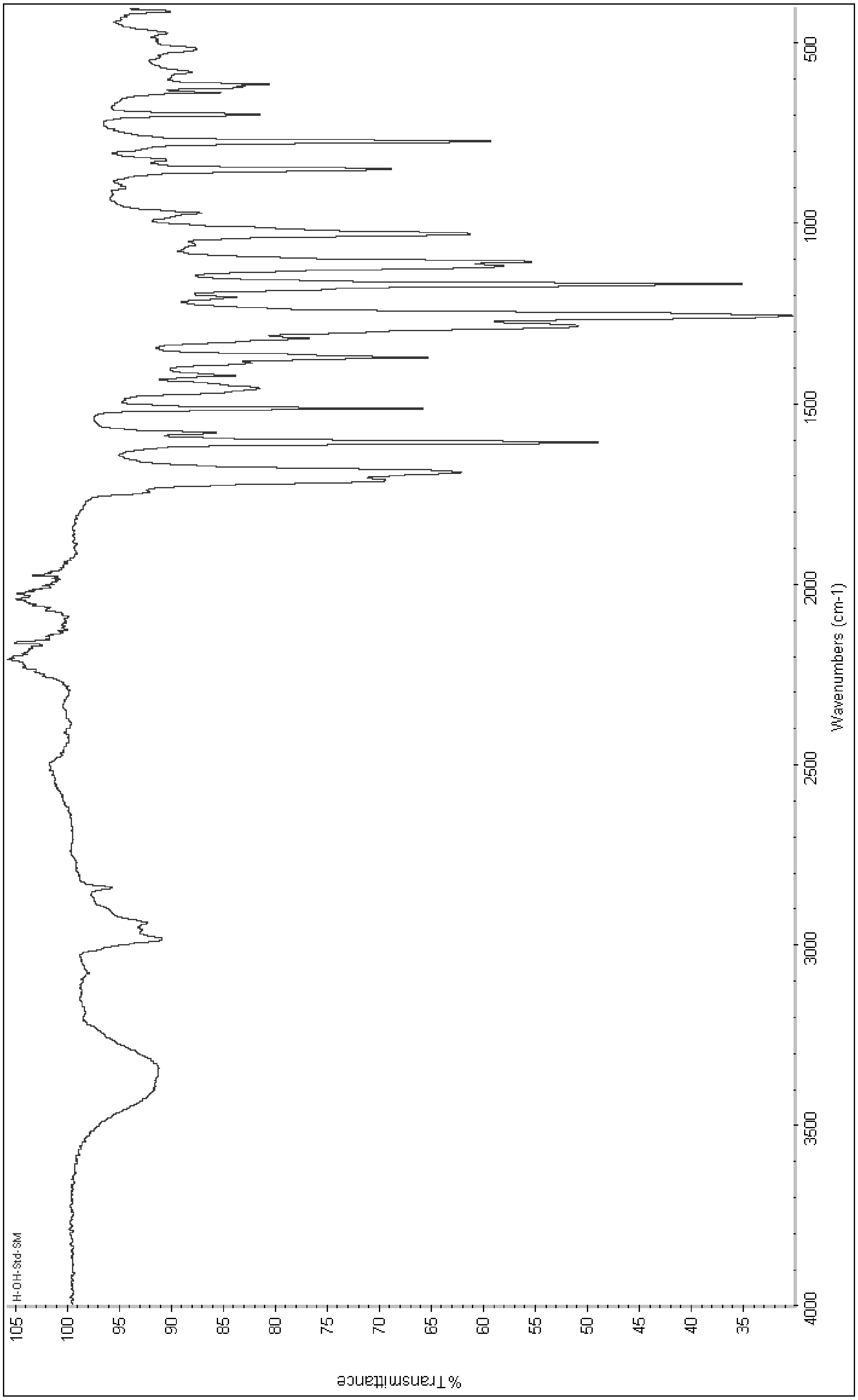
H-OH-Std-SM
%Transmittance
Wavenumbers (cm-1)
105
100
95
90
85
80
75
70
65
60
55
50
45
40
35
4000
3500
3000
2500
2000
1500
1000
500

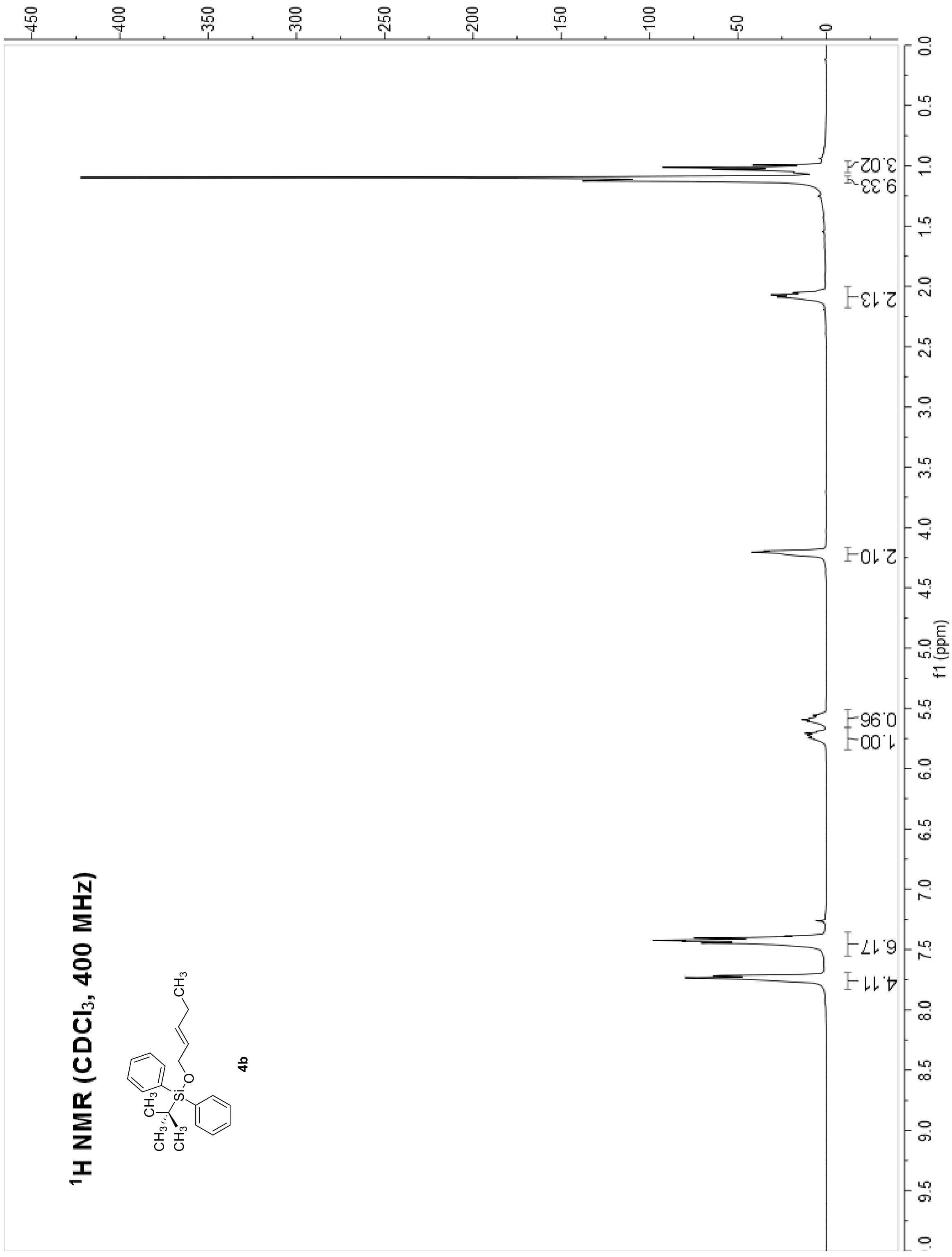

1H NMR (CDCl3, 400 MHz)
4b
3.02
9.33
2.13
2.10
0.96
1.00
6.17
4.11
f1 (ppm)

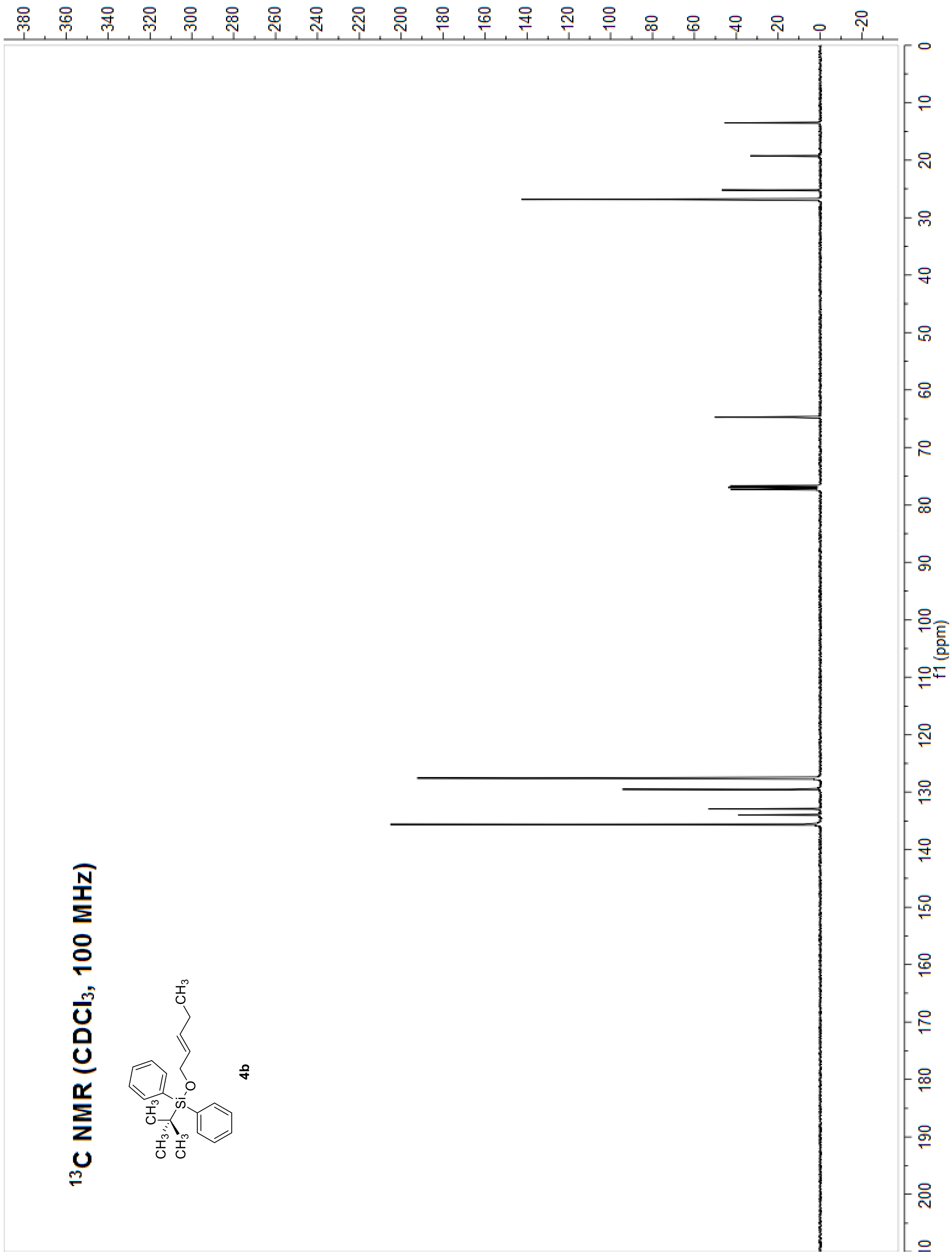

^{13}C NMR ($CDCl_3$, 100 MHz)
4b
f1 (ppm)

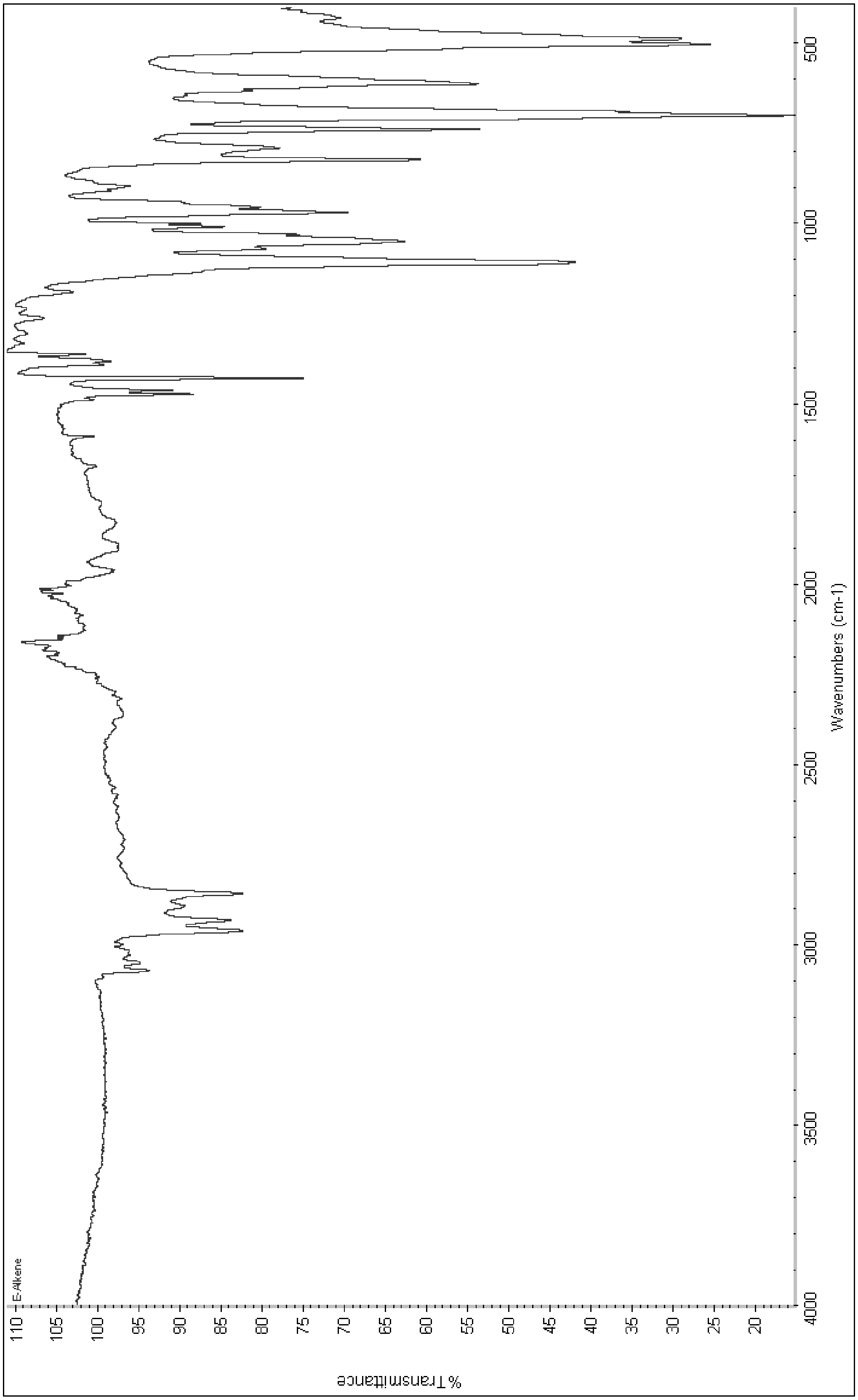
E-Alkene
%Transmittance
Wavenumbers (cm-1)
110
105
100
95
90
85
80
75
70
65
60
55
50
45
40
35
30
25
20
4000
3500
3000
2500
2000
1500
1000
500

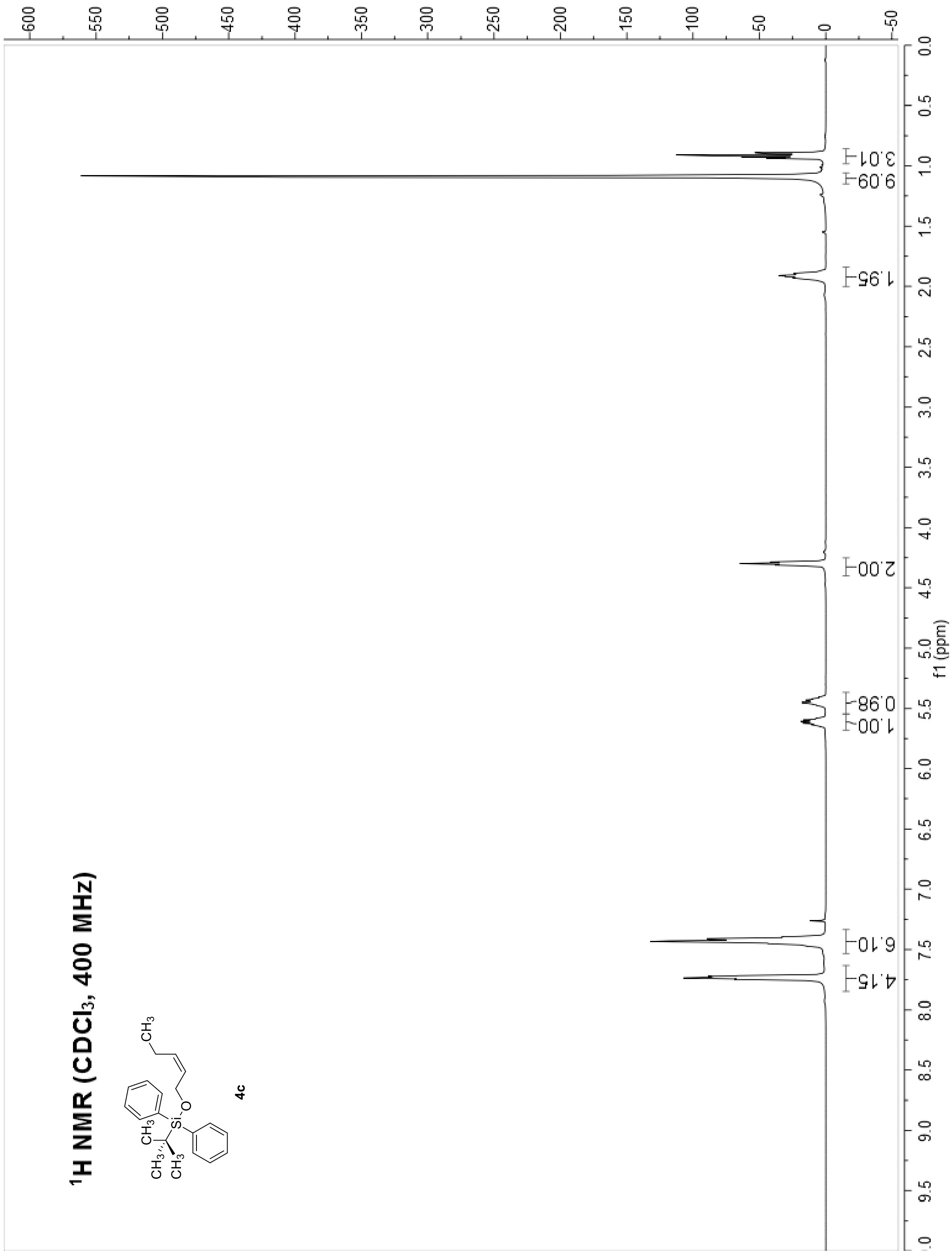

1H NMR (CDCl3, 400 MHz)
CH3
CH3
CH3
Si
O
CH3
4c
3.01
9.09
1.95
2.00
0.98
1.00
6.10
4.15
f1 (ppm)

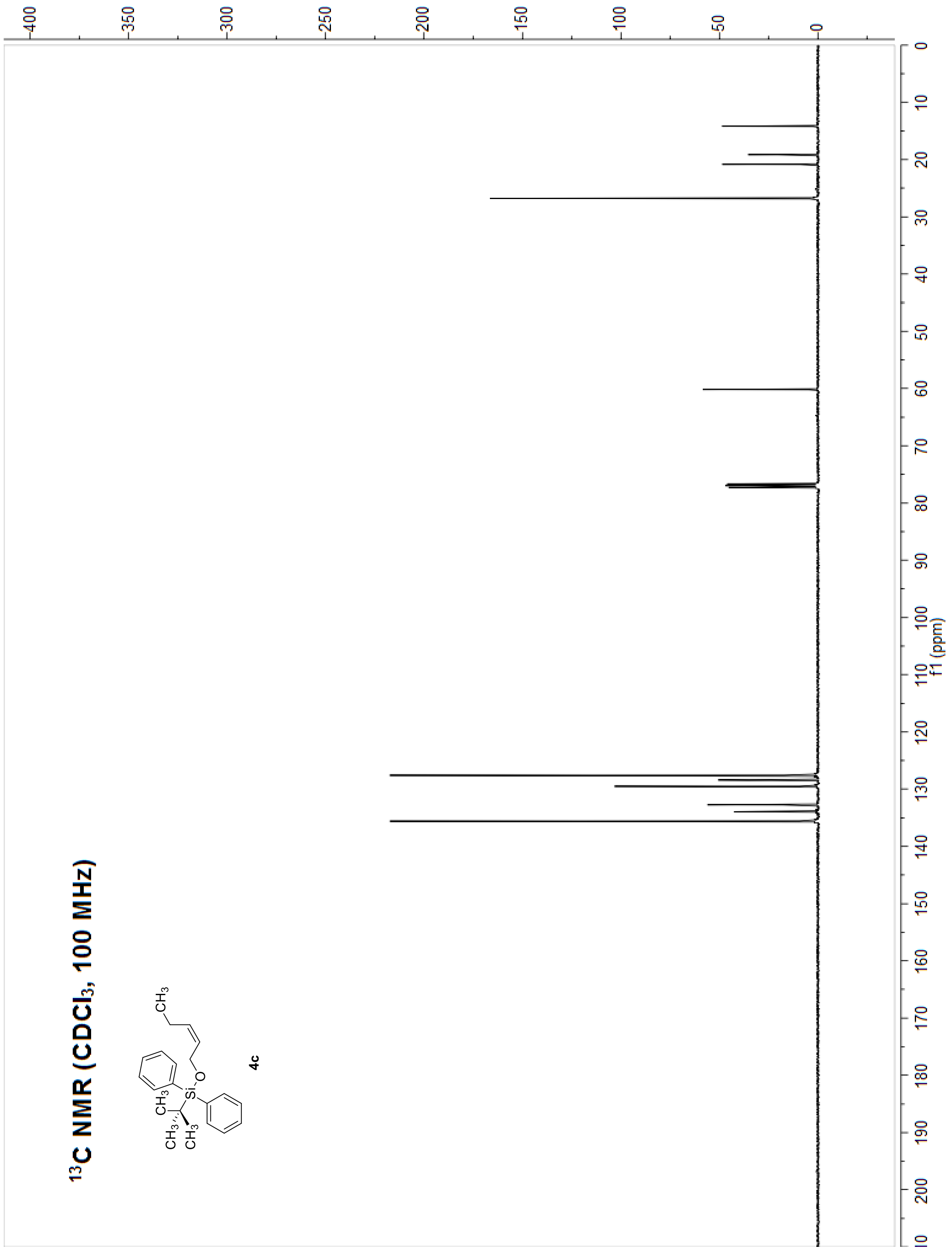
13C NMR (CDCl3, 100 MHz)
4c
f1 (ppm)

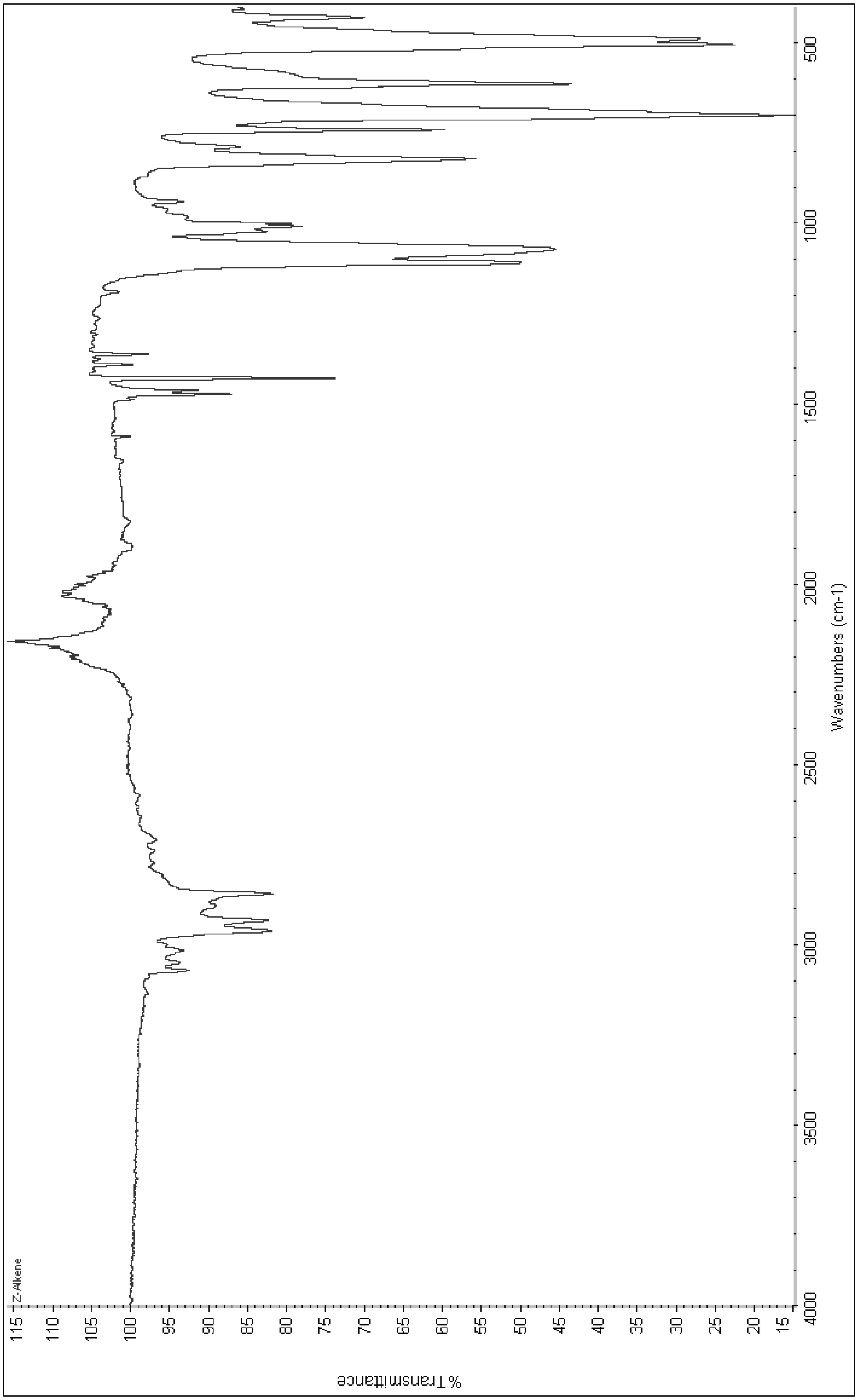
Z-Alkene
%Transmittance
Wavenumbers (cm-1)
115
110
105
100
95
90
85
80
75
70
65
60
55
50
45
40
35
30
25
20
15
4000
3500
3000
2500
2000
1500
1000
500

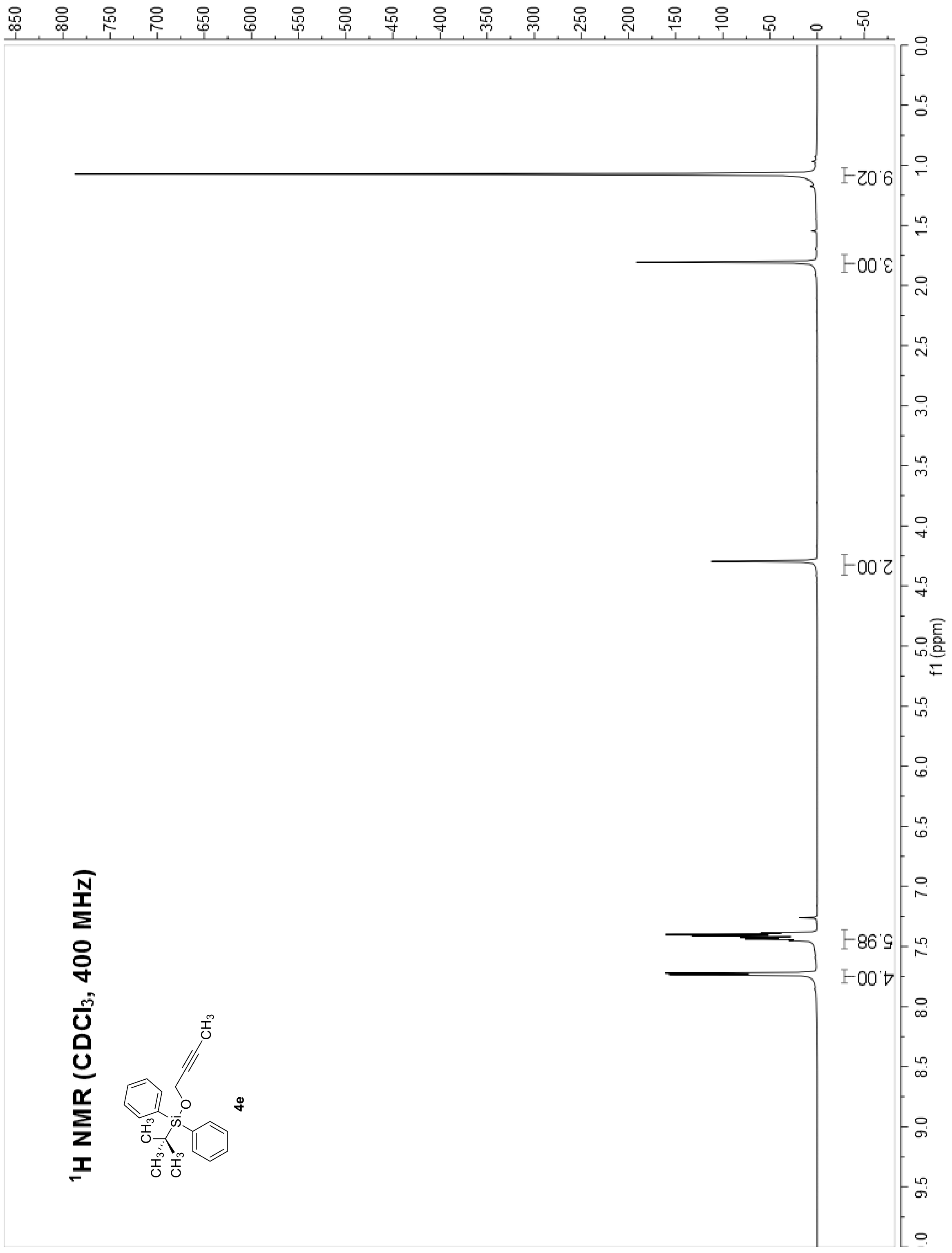

1H NMR (CDCl3, 400 MHz)
4e
f1 (ppm)
9.02
3.00
2.00
5.98
4.00

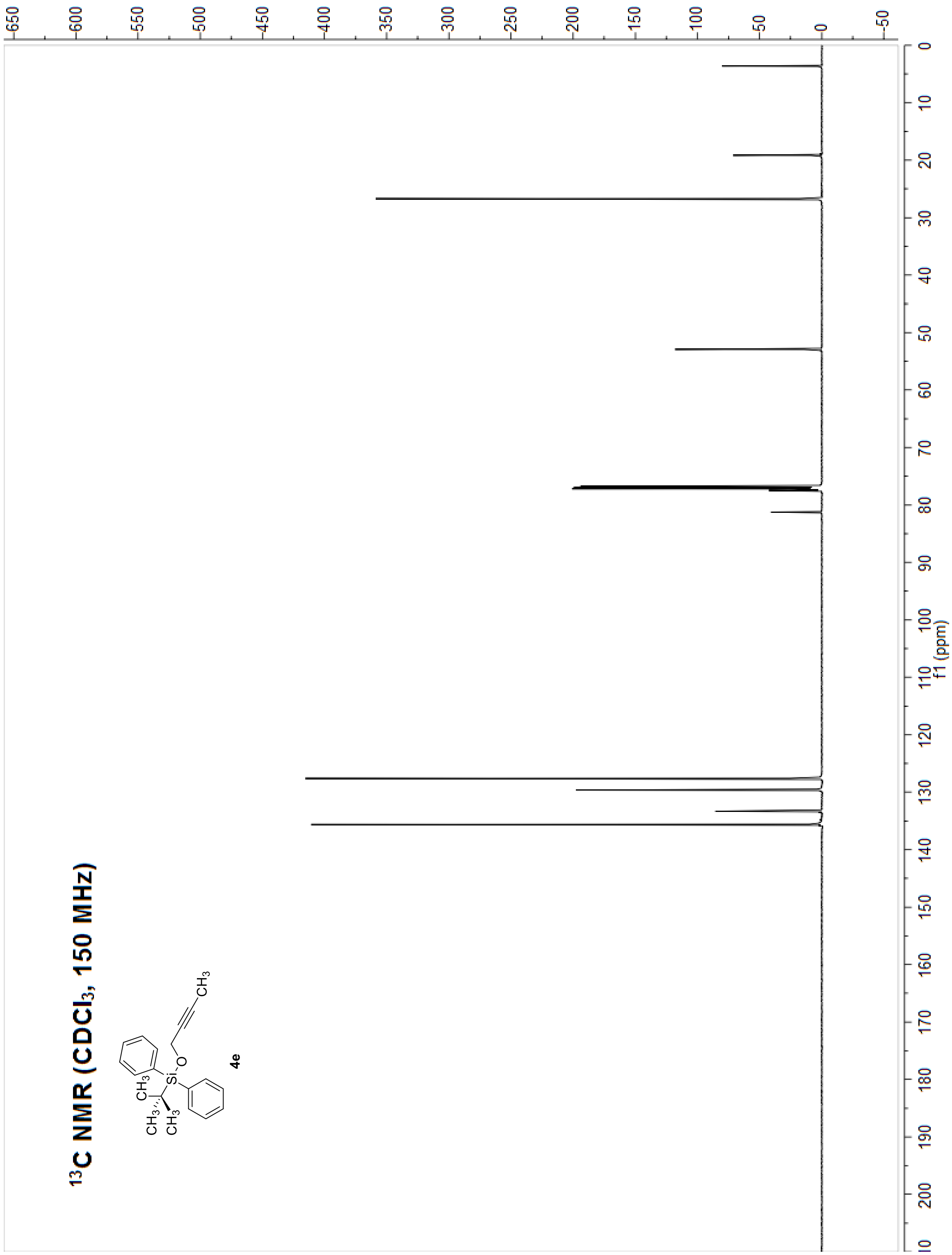
13C NMR (CDCl3, 150 MHz)
4e
f1 (ppm)

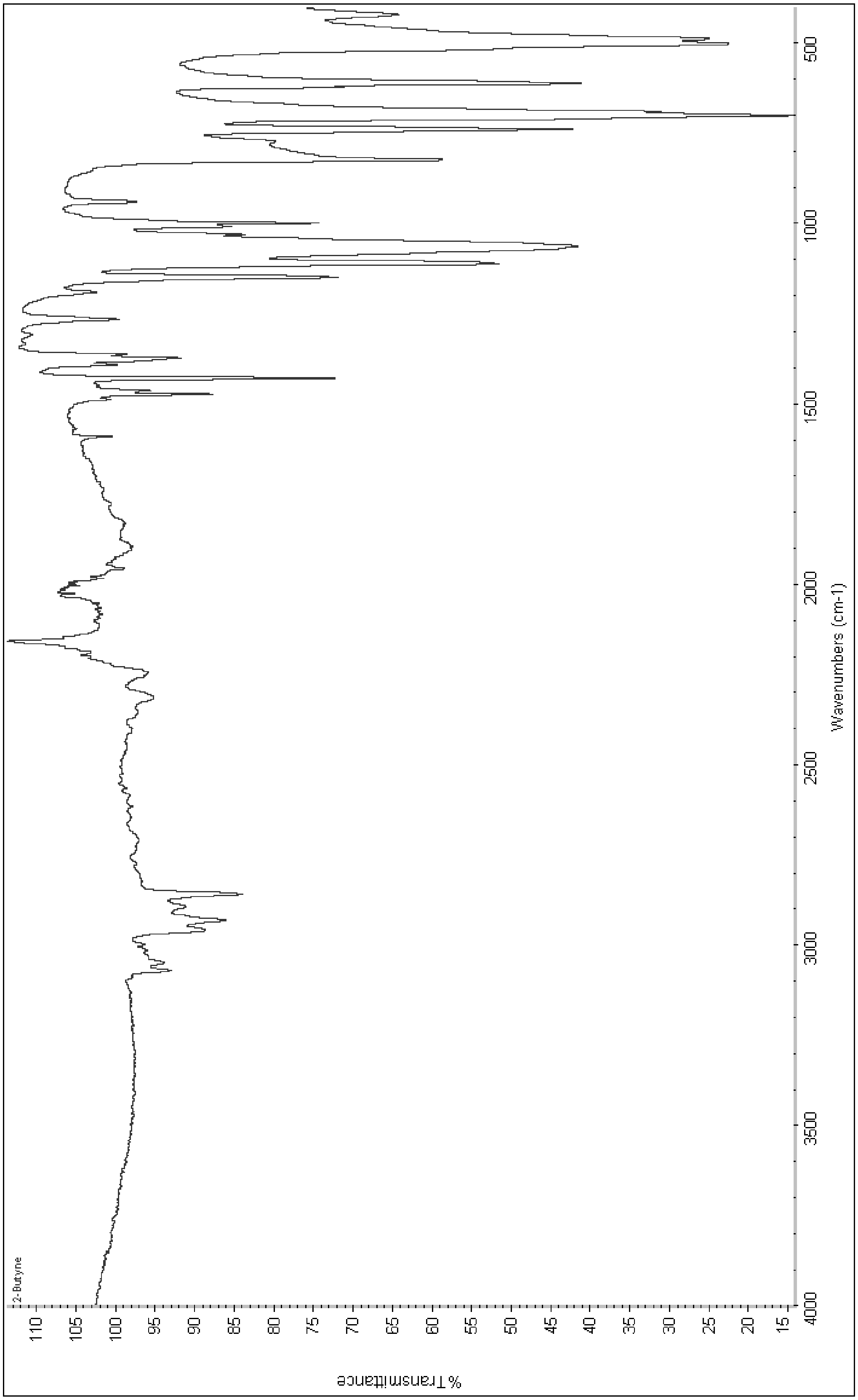
2-Butyne
%Transmittance
Wavenumbers (cm-1)
110
105
100
95
90
85
80
75
70
65
60
55
50
45
40
35
30
25
20
15
4000
3500
3000
2500
2000
1500
1000
500

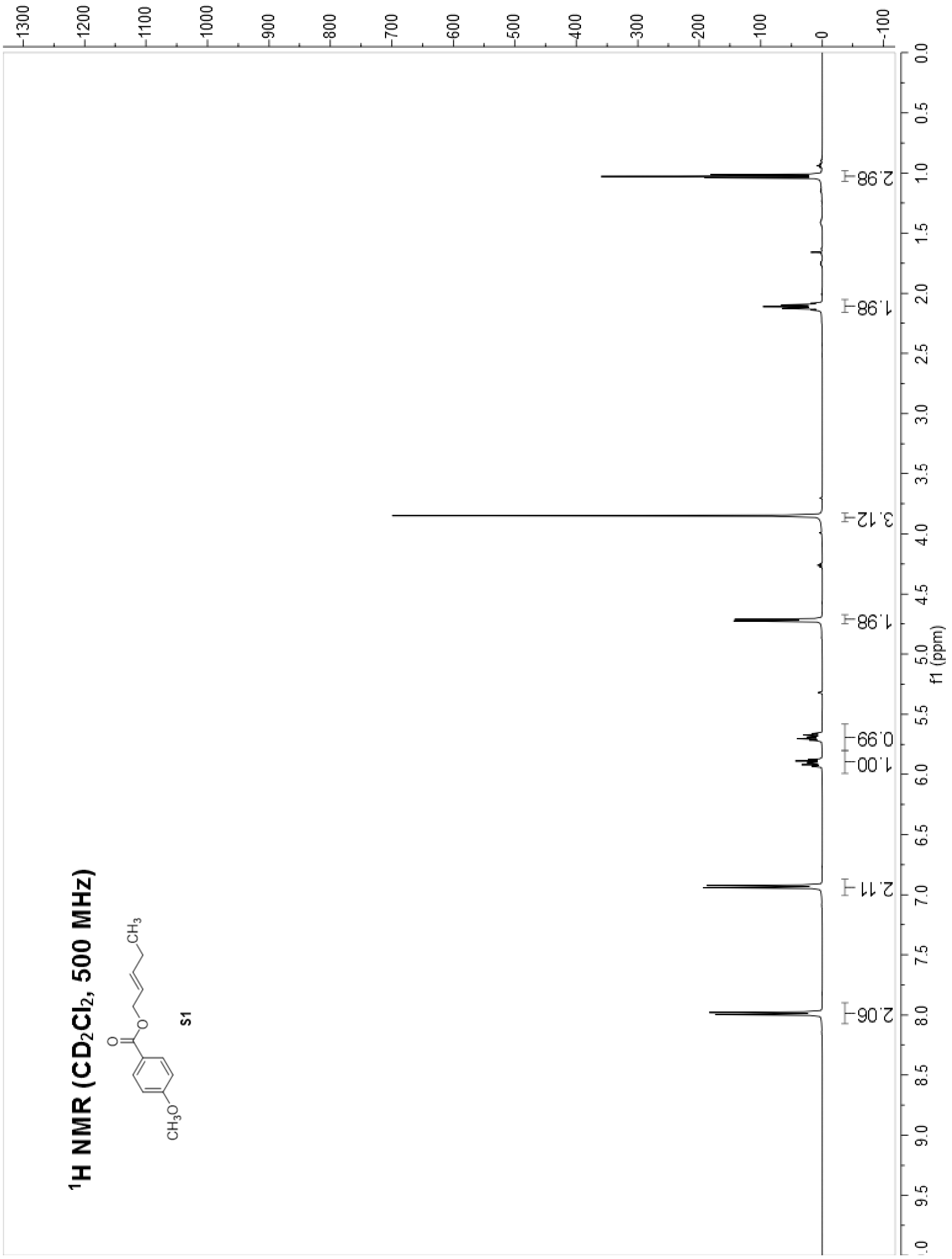

1H NMR (CD2Cl2, 500 MHz)
S1
f1 (ppm)

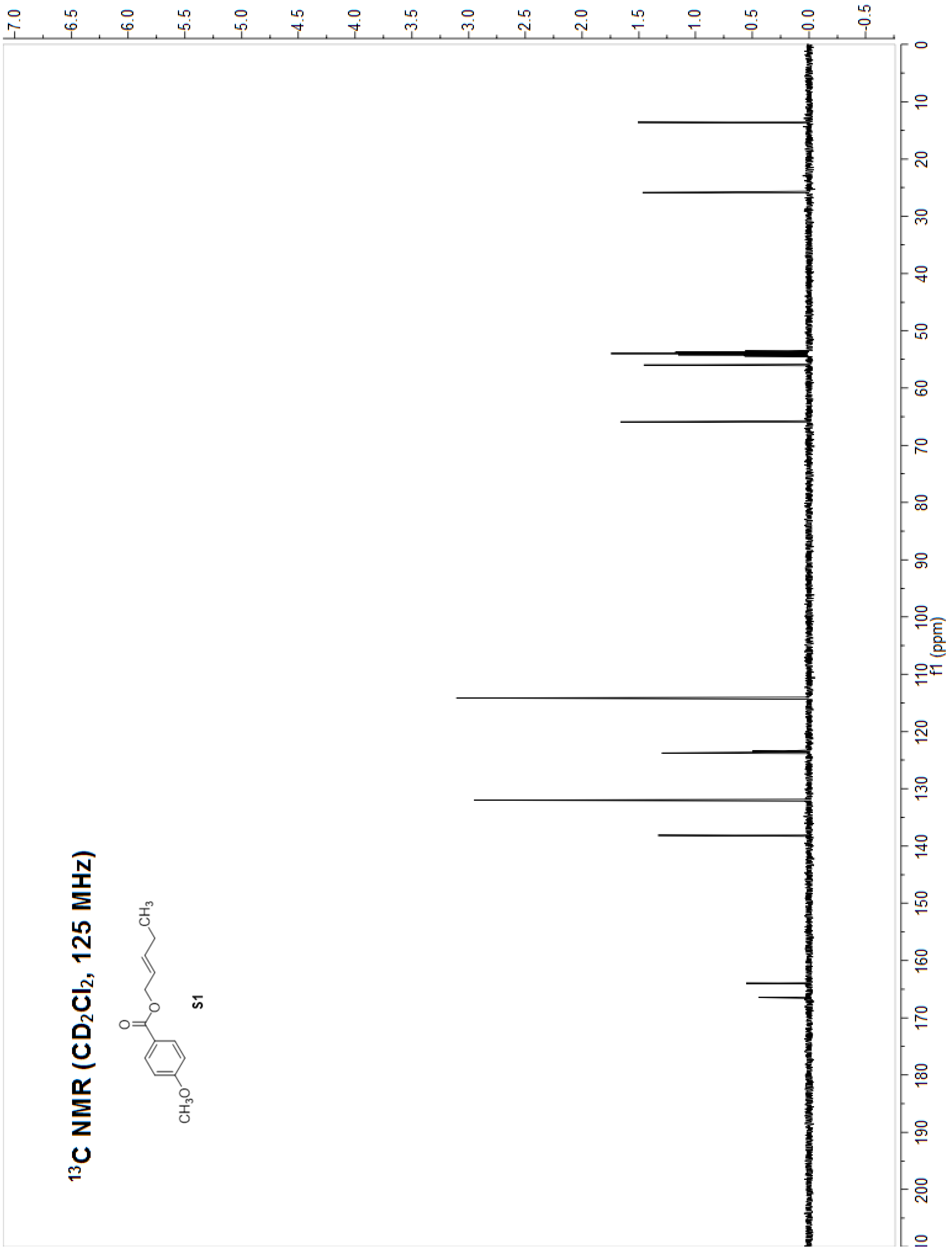

¹³C NMR (CD₂Cl₂, 125 MHz)
S1
f1 (ppm)

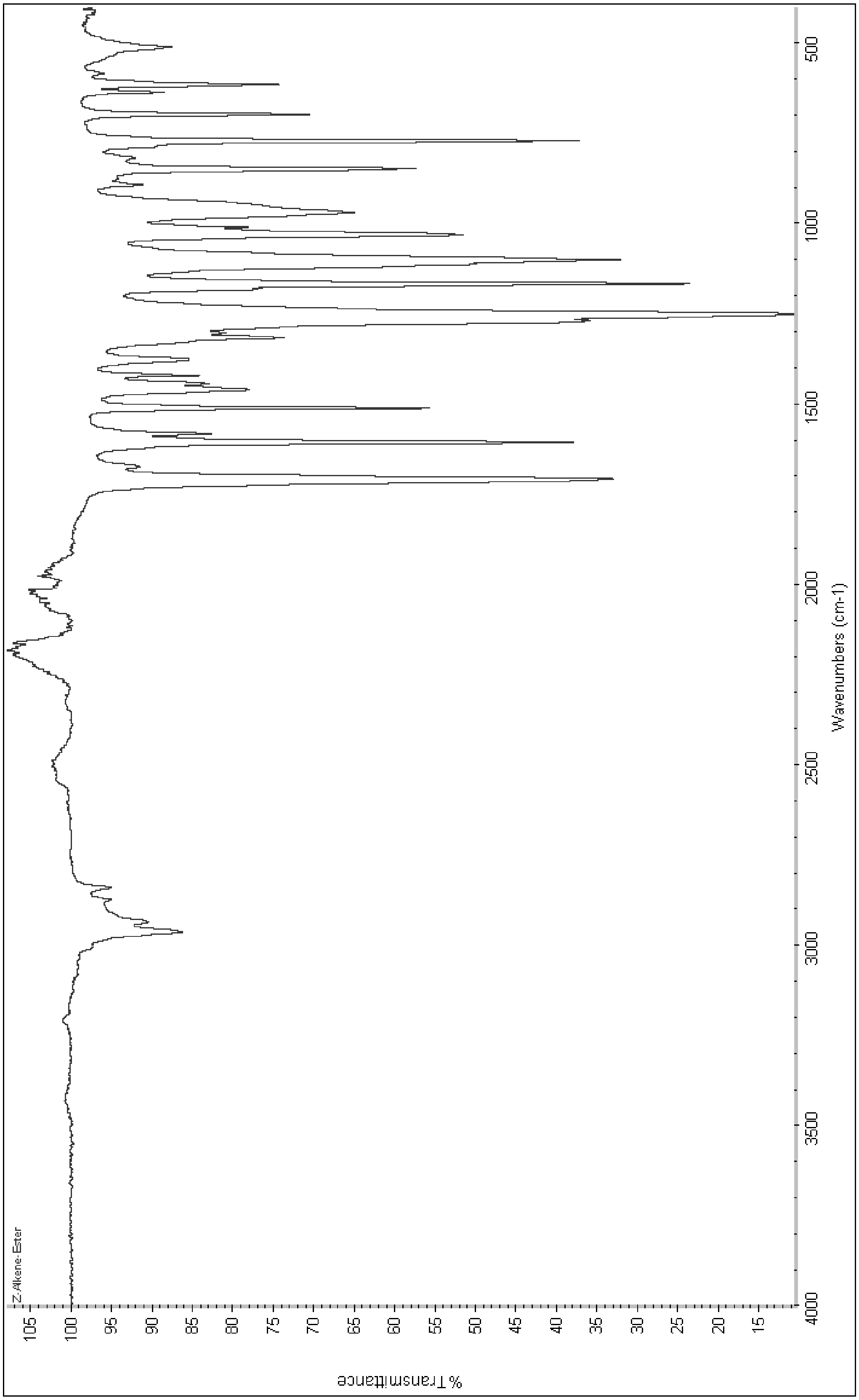
Z-Alkene-Ester
%Transmittance
Wavenumbers (cm-1)
105
100
95
90
85
80
75
70
65
60
55
50
45
40
35
30
25
20
15
4000
3500
3000
2500
2000
1500
1000
500

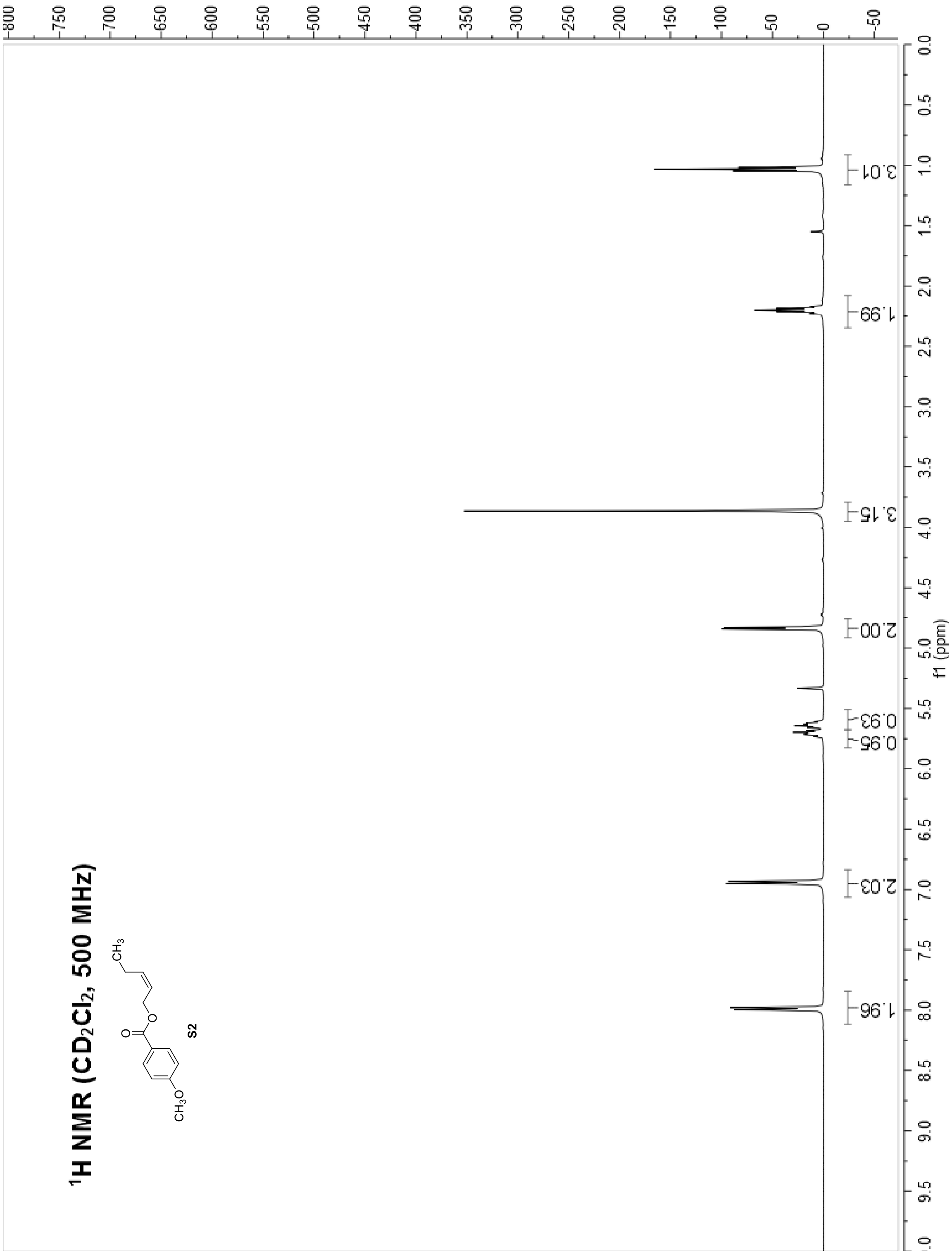

¹H NMR (CD₂Cl₂, 500 MHz)
S2
f1 (ppm)

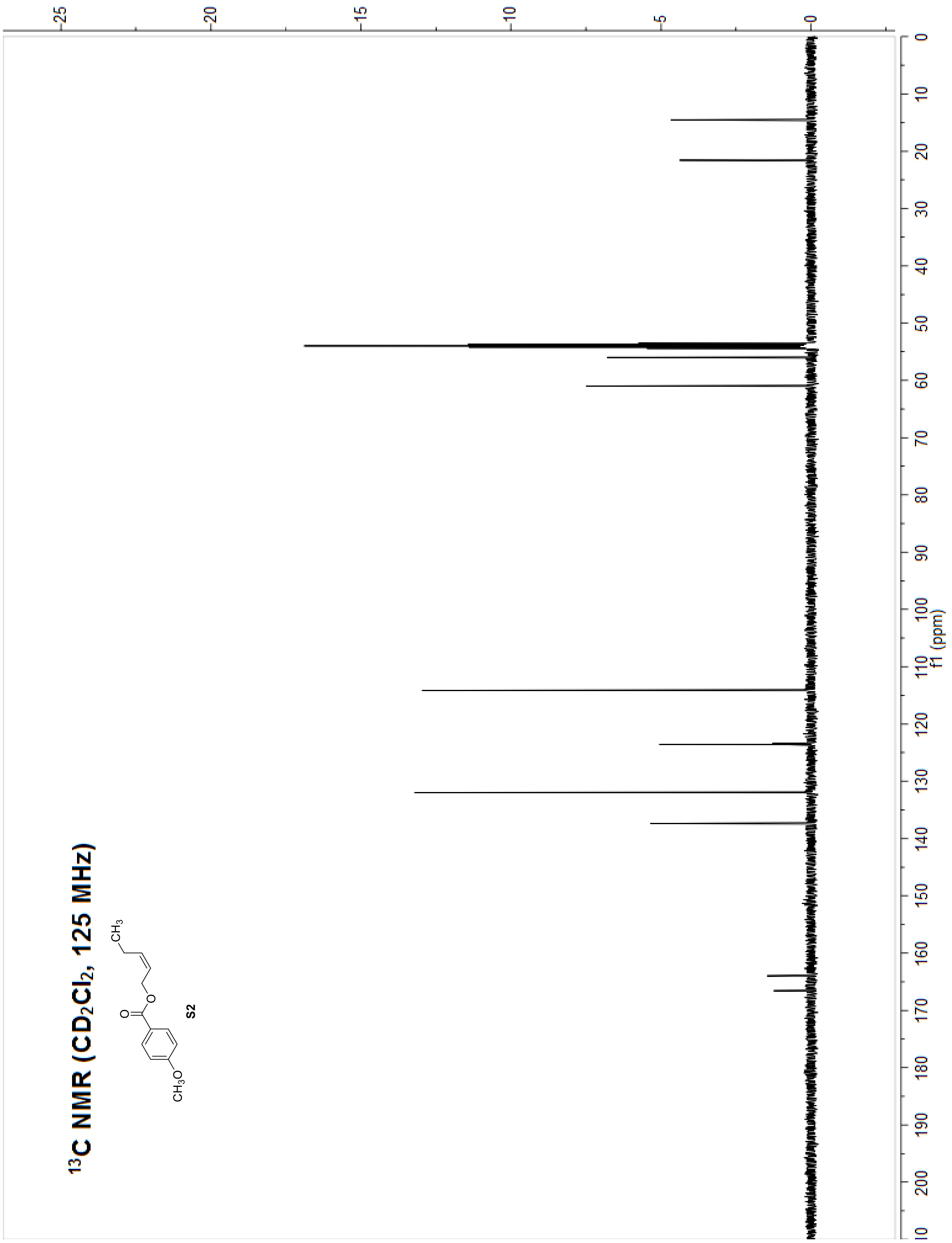

13C NMR (CD2Cl2, 125 MHz)
S2
f1 (ppm)

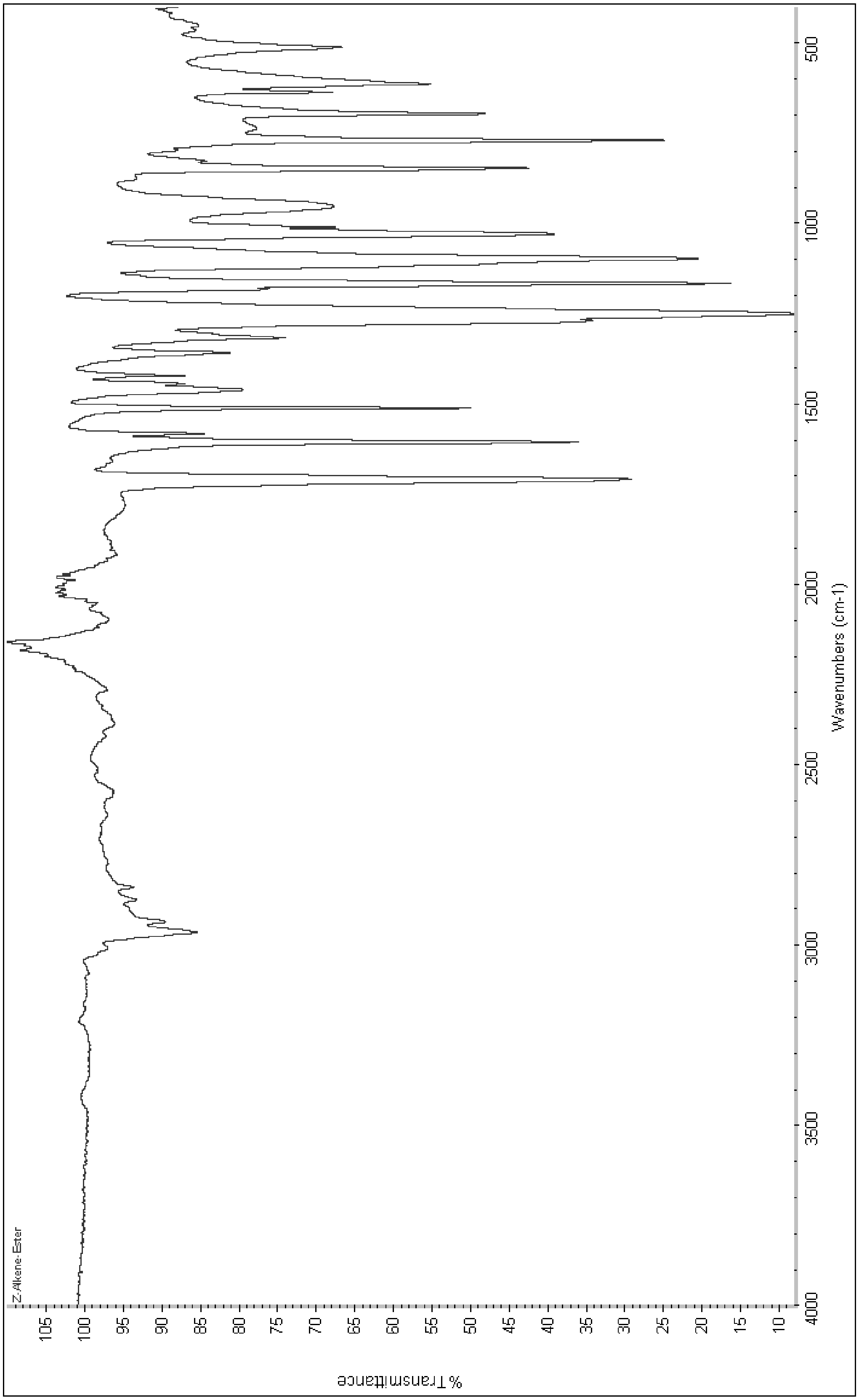
Z-Alkene-Ester
%Transmittance
Wavenumbers (cm-1)

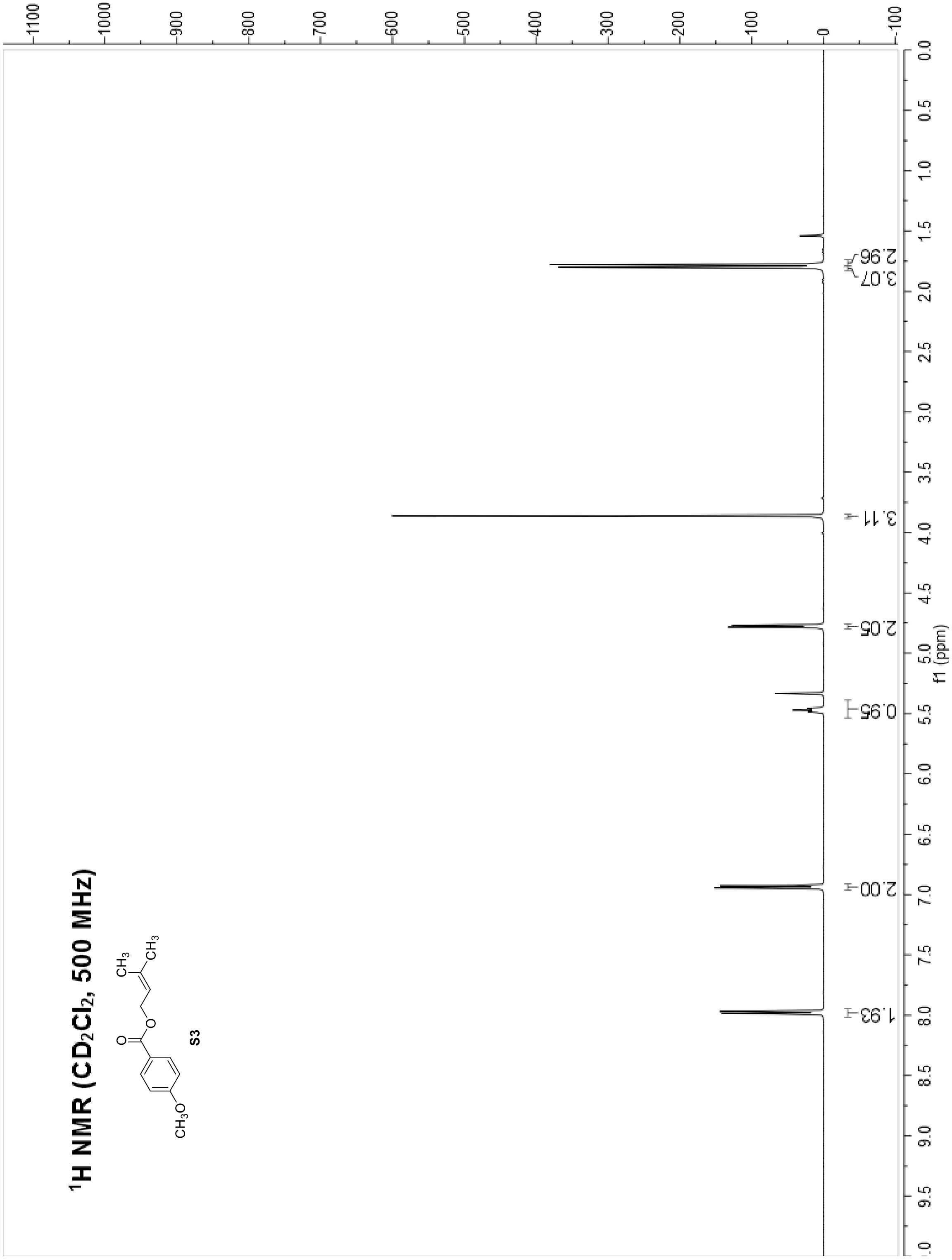
1H NMR (CD2Cl2, 500 MHz)
S3
CH3O
O
CH3
CH3
2.96
3.07
3.11
2.05
0.95
2.00
1.93
f1 (ppm)

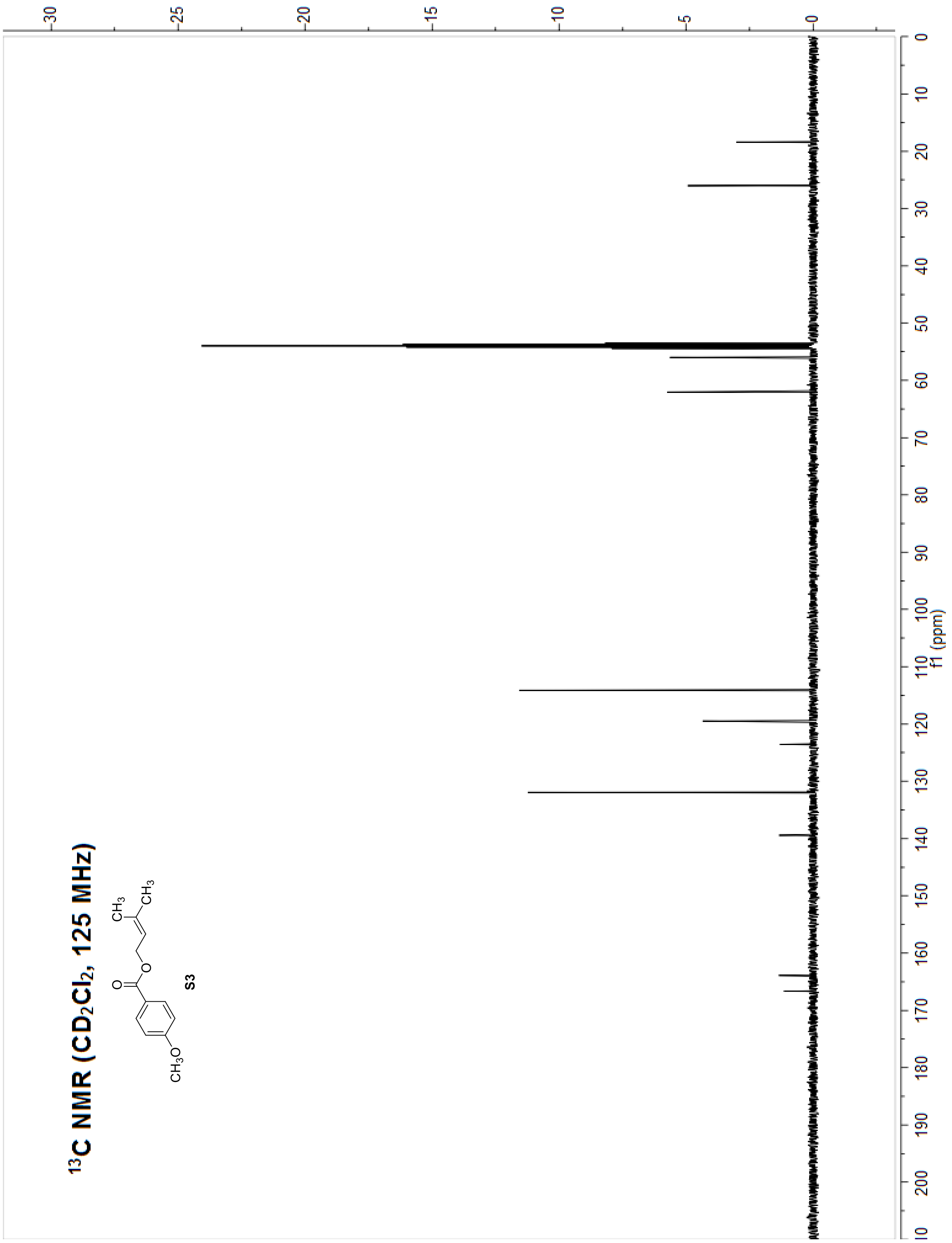
13C NMR (CD2Cl2, 125 MHz)
S3
f1 (ppm)

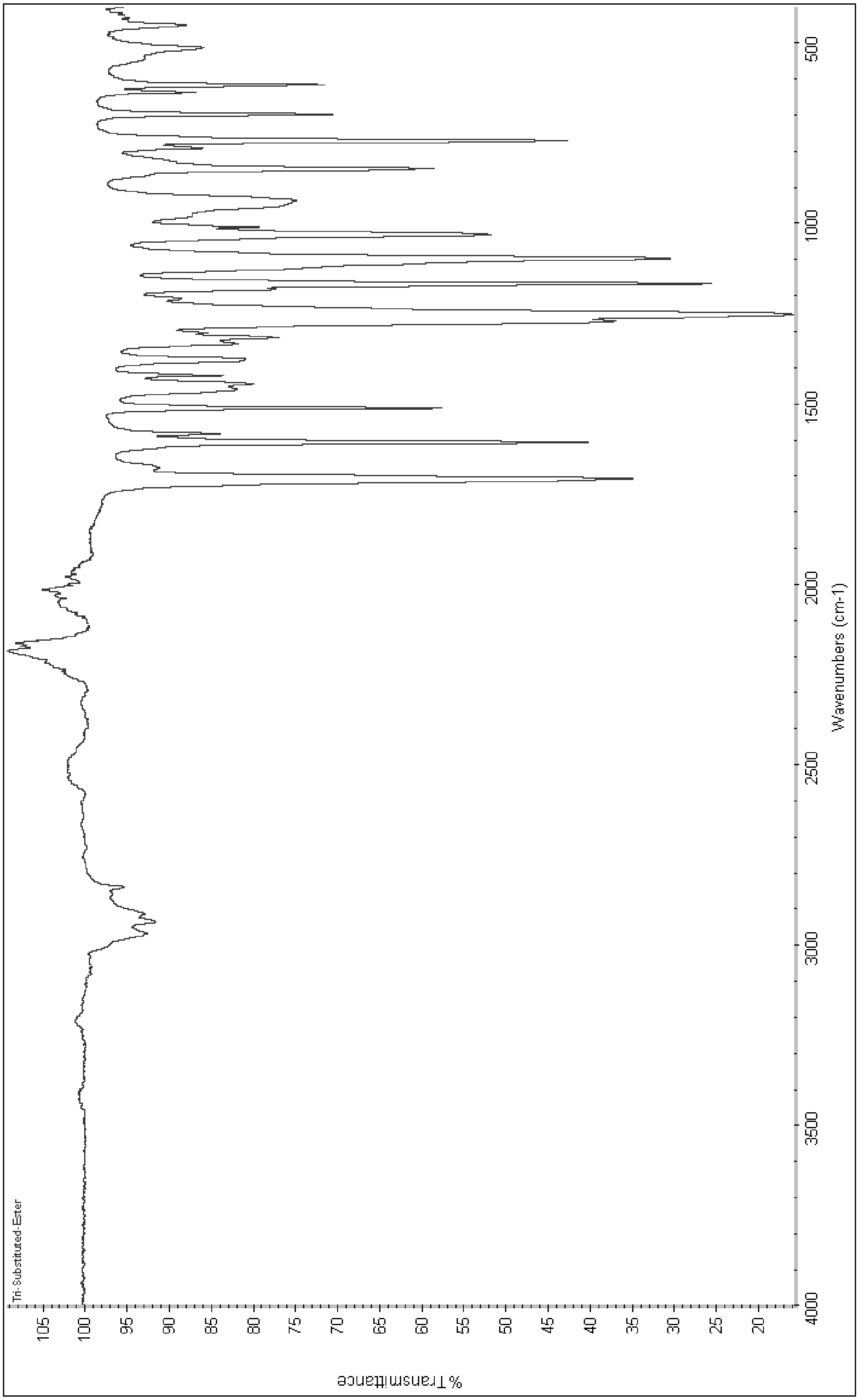
Tri-Substituted-Ester
%Transmittance
Wavenumbers (cm-1)
105
100
95
90
85
80
75
70
65
60
55
50
45
40
35
30
25
20
4000
3500
3000
2500
2000
1500
1000
500

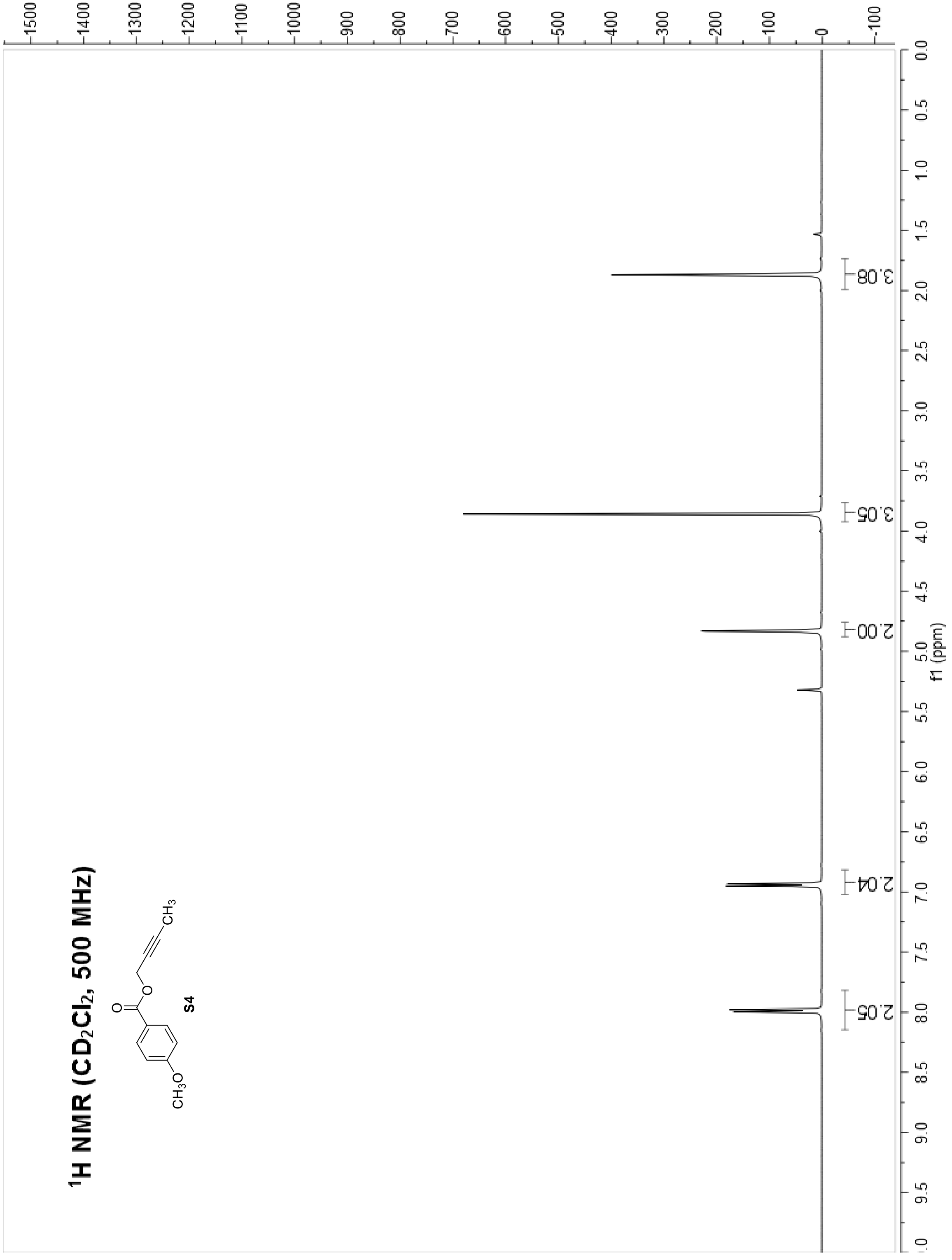

1H NMR (CD2Cl2, 500 MHz)
S4
CH3O
O
CH3
f1 (ppm)
3.08
3.05
2.00
2.04
2.05

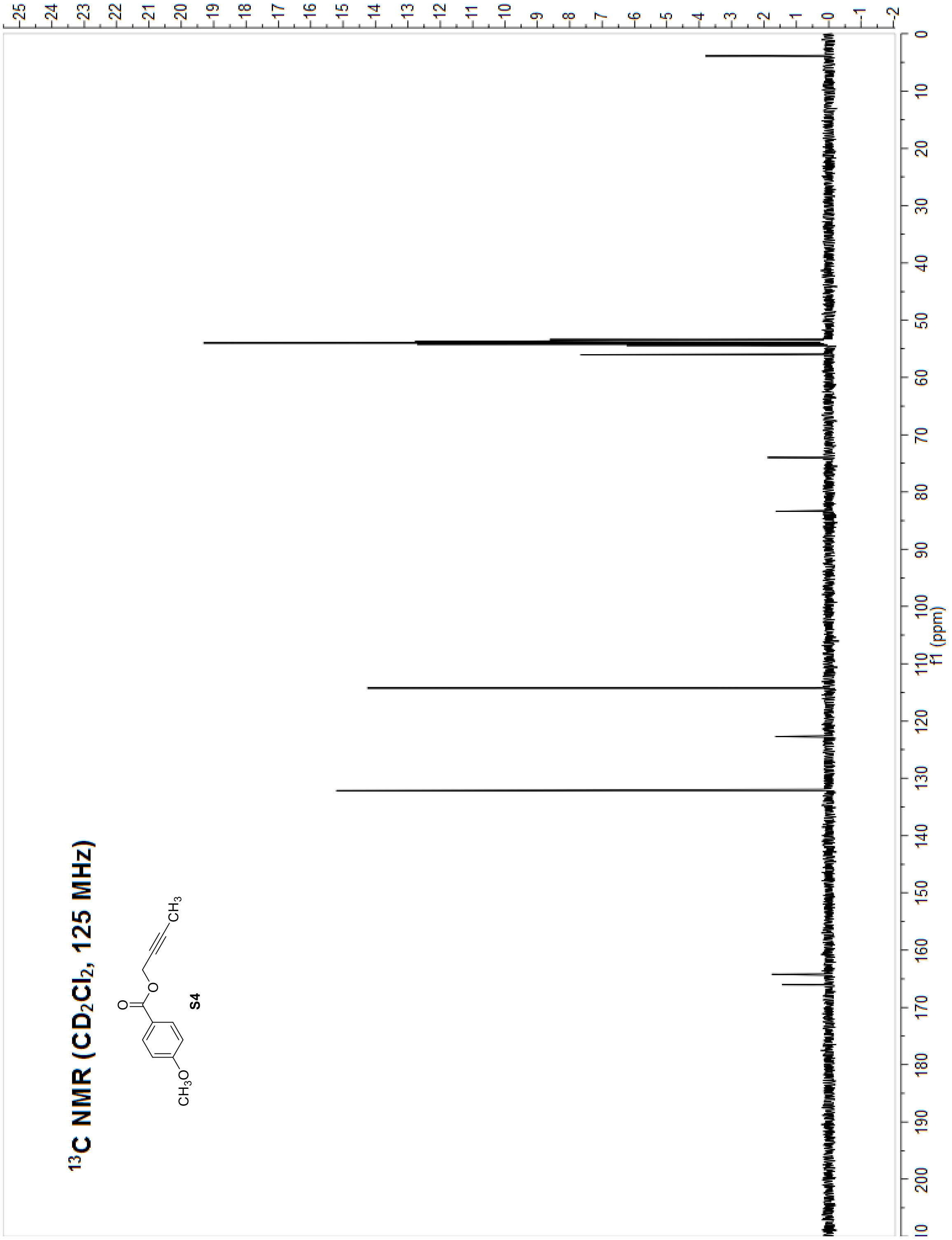

^{13}C NMR (CD$_2$Cl$_2$, 125 MHz)
CH$_3$O
O
O
CH$_3$
S4
f1 (ppm)

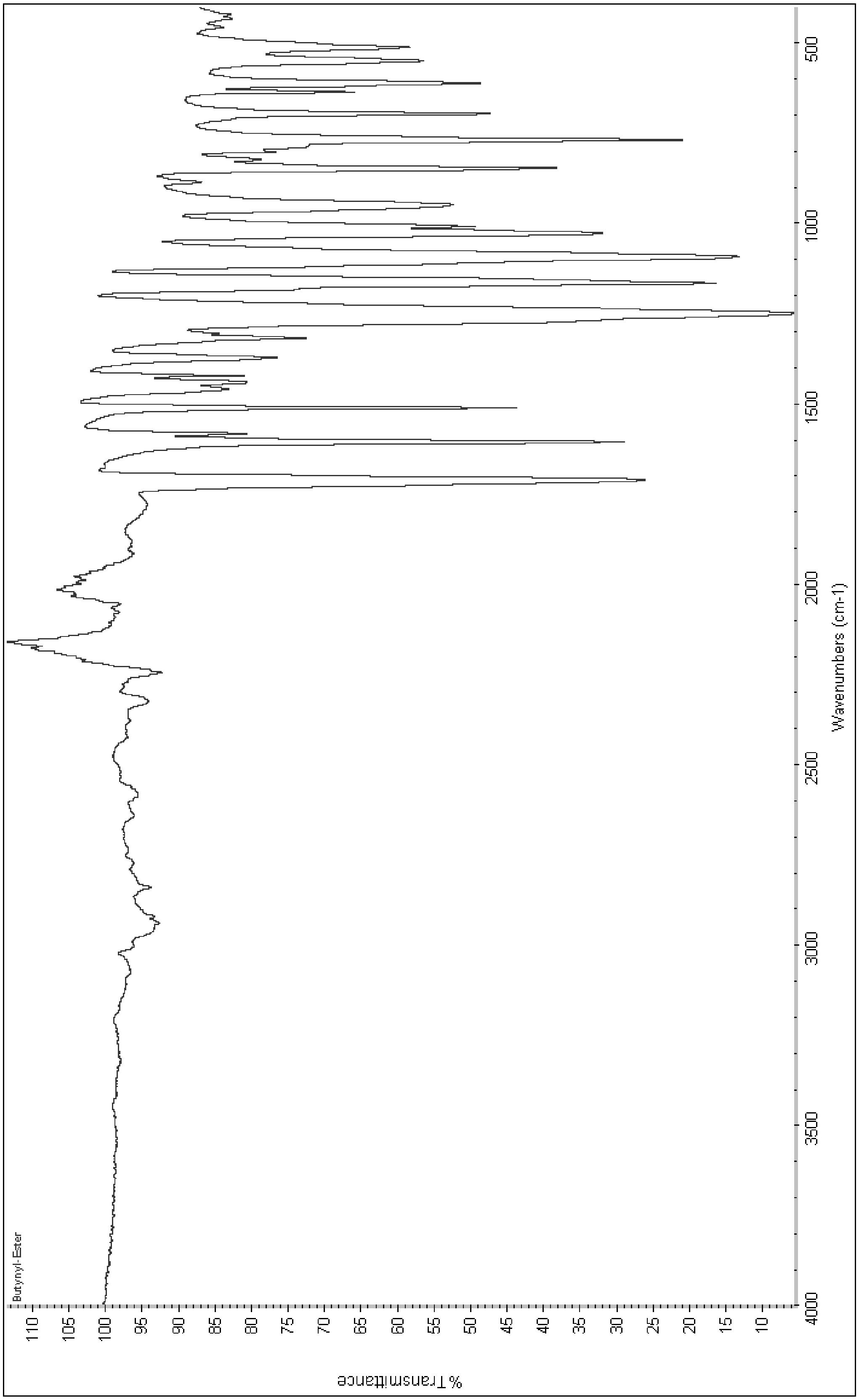
Butynyl-Ester
%Transmittance
Wavenumbers (cm-1)
110
105
100
95
90
85
80
75
70
65
60
55
50
45
40
35
30
25
20
15
10
4000
3500
3000
2500
2000
1500
1000
500

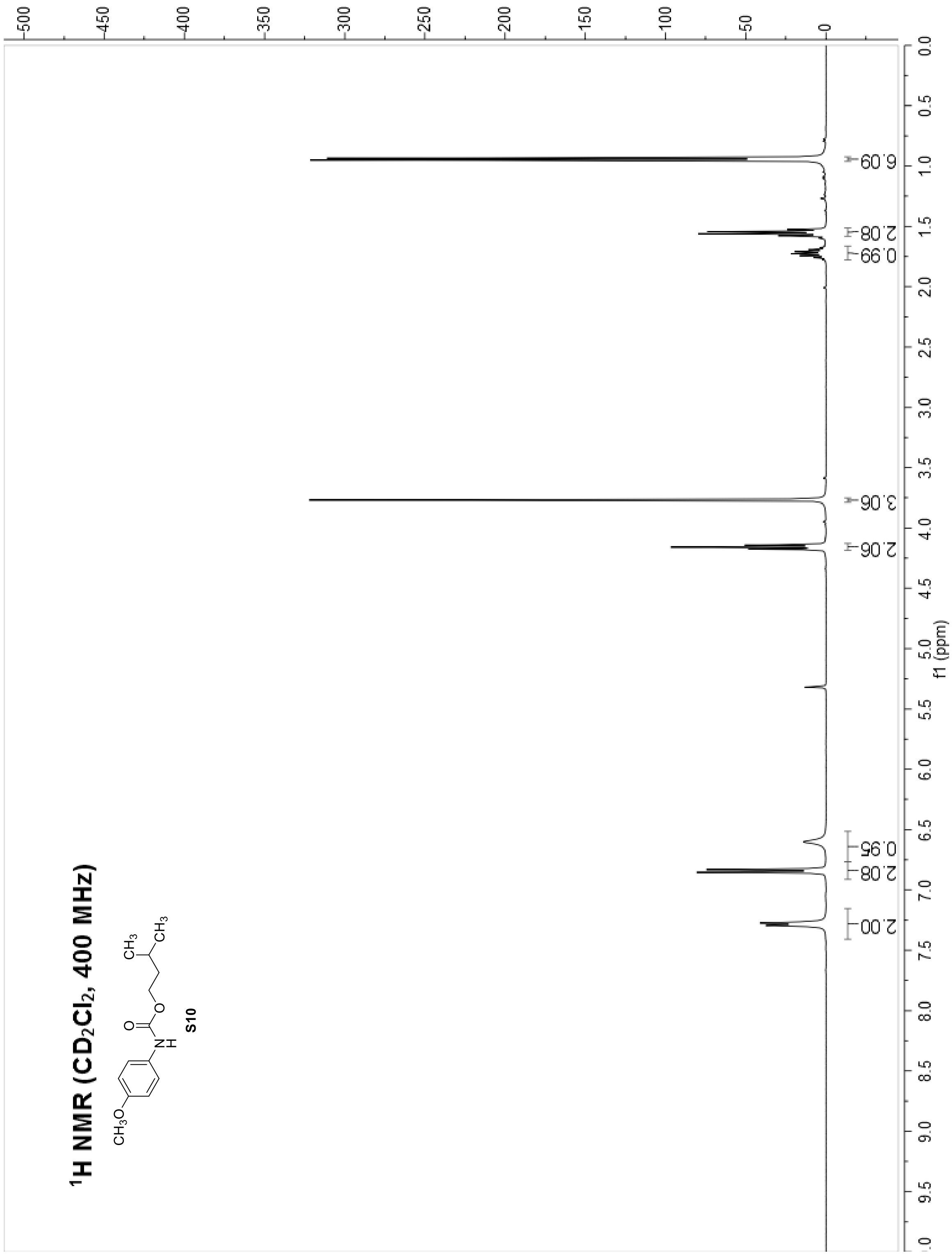
1H NMR (CD2Cl2, 400 MHz)
S10
f1 (ppm)
6.09
2.08
0.99
3.06
2.06
0.95
2.08
2.00

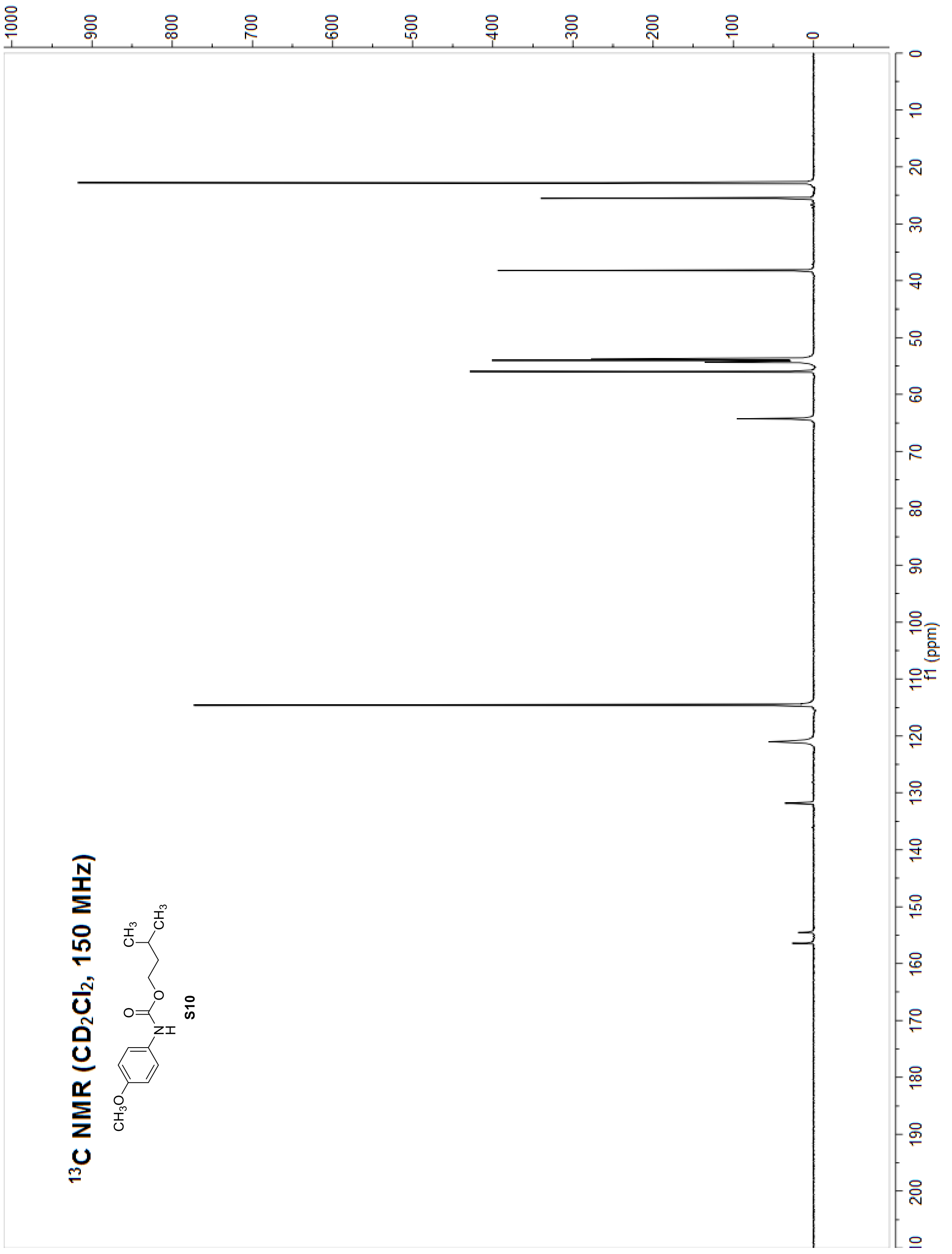

13C NMR (CD2Cl2, 150 MHz)
CH3O
N
H
O
O
CH3
CH3
S10
f1 (ppm)

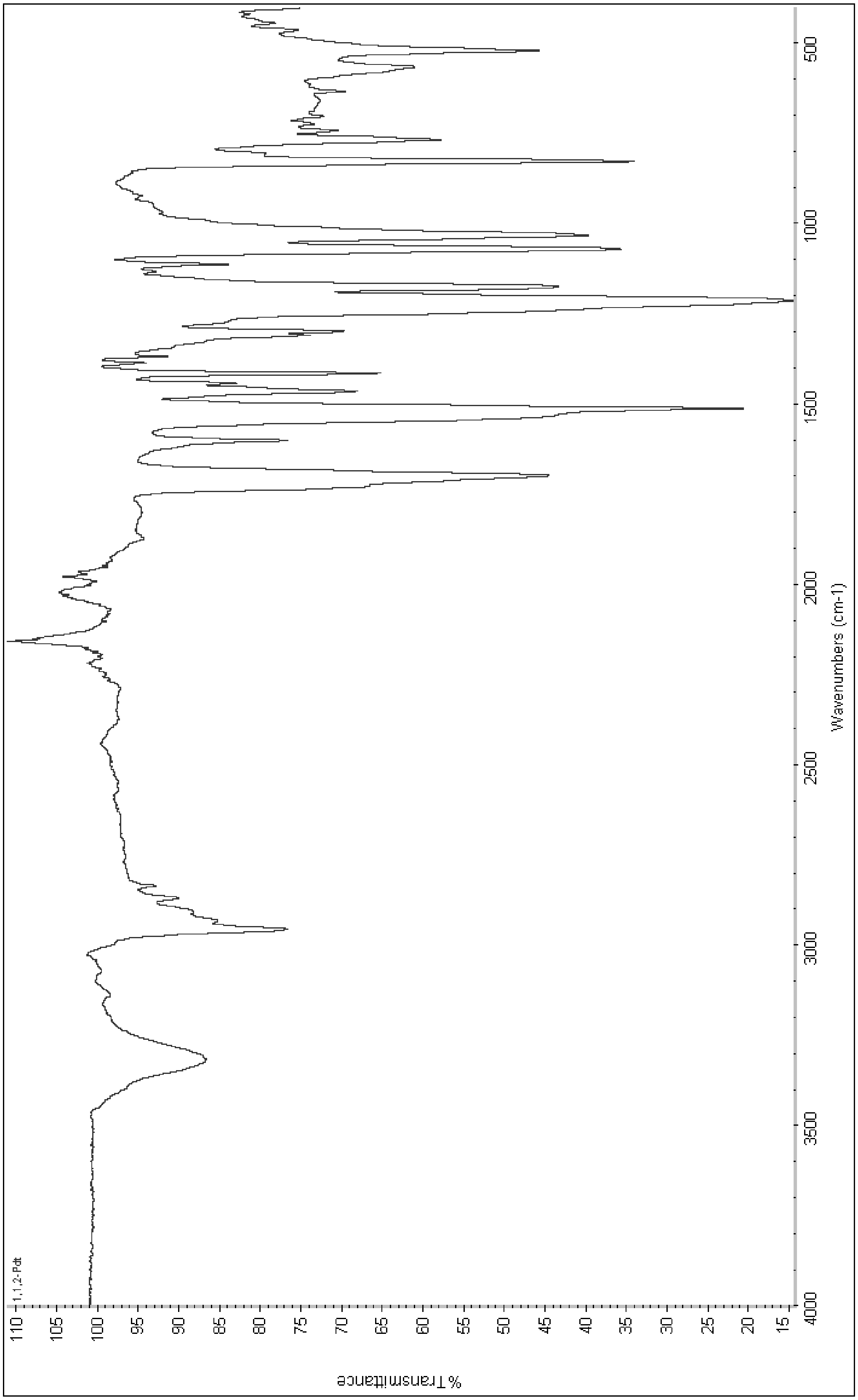
1,1,2-Pdt
%Transmittance
Wavenumbers (cm-1)
110 105 100 95 90 85 80 75 70 65 60 55 50 45 40 35 30 25 20 15
4000 3500 3000 2500 2000 1500 1000 500

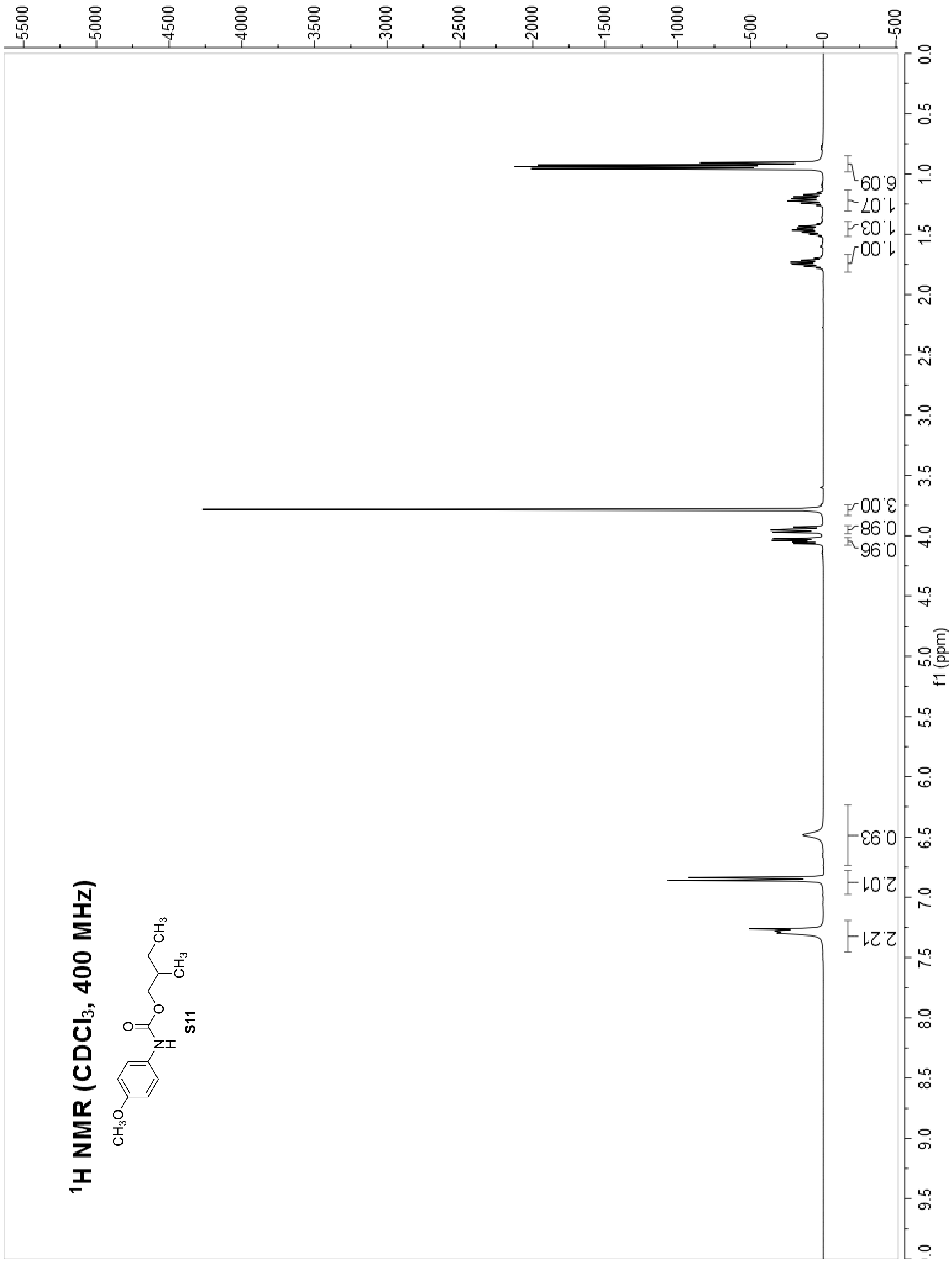

1H NMR (CDCl3, 400 MHz)
S11
f1 (ppm)
6.09
1.07
1.03
1.00
3.00
0.98
0.96
0.93
2.01
2.21

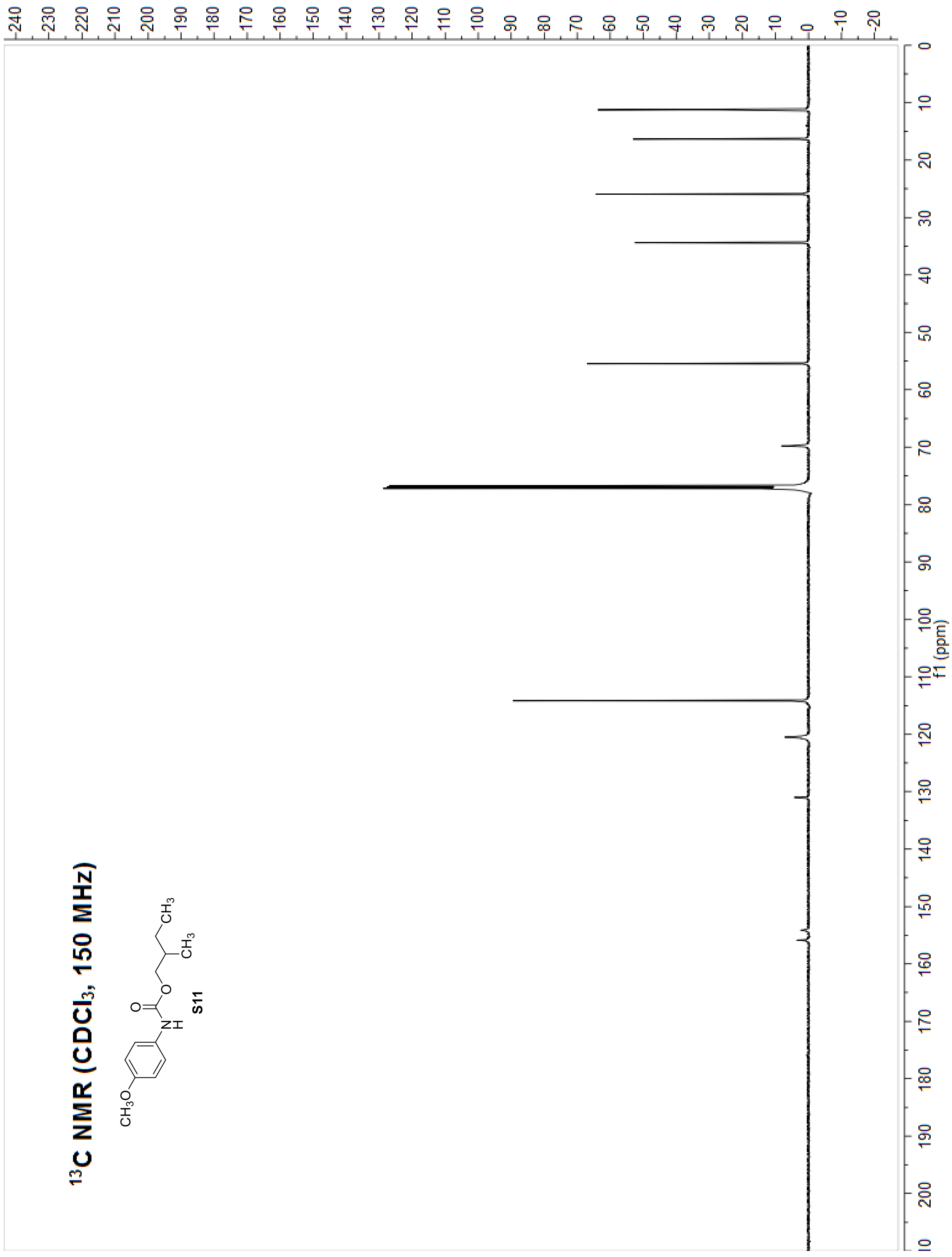
13C NMR (CDCl3, 150 MHz)
CH3O
N
H
O
O
CH3
CH3
S11
f1 (ppm)

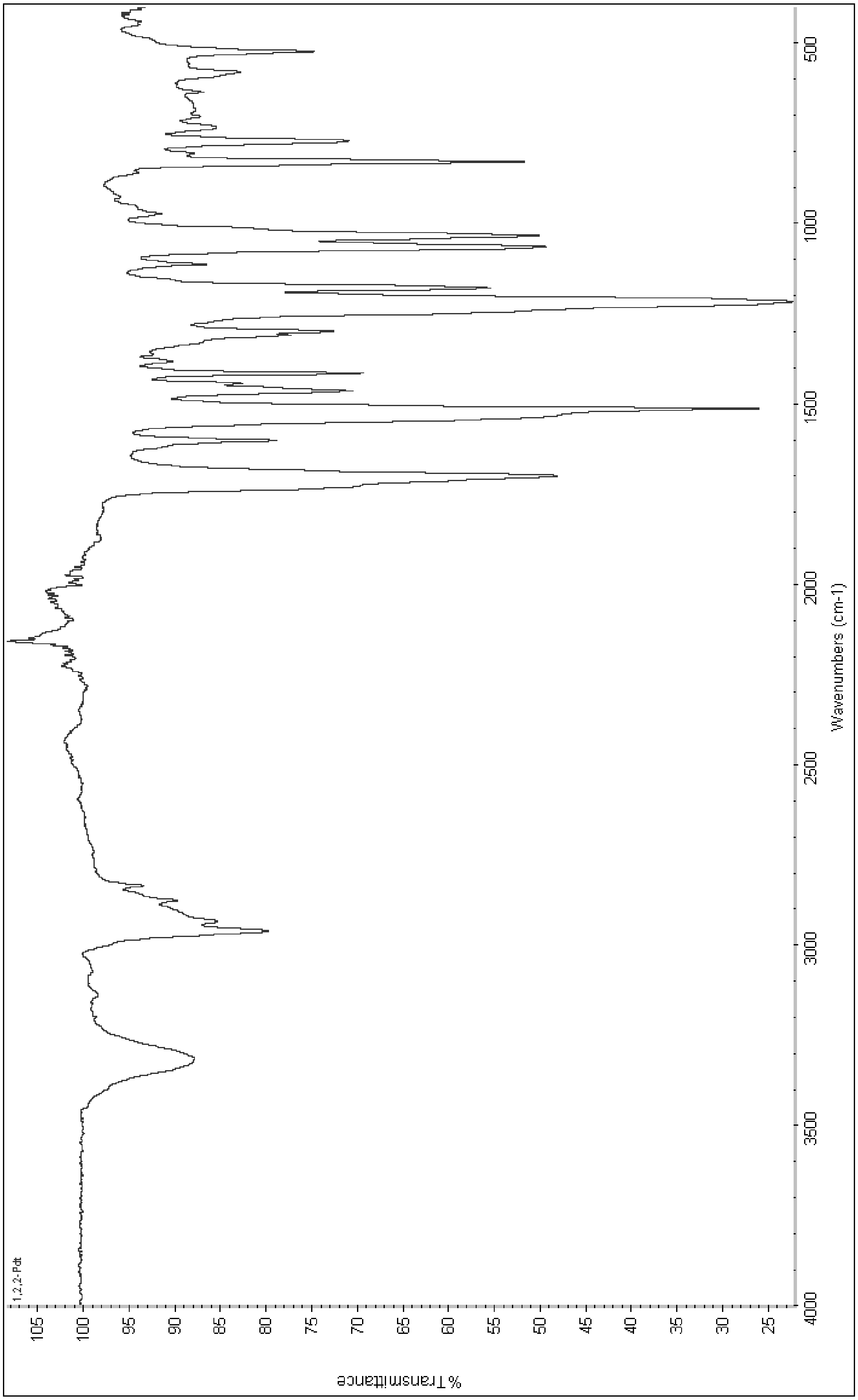
1,2,2-Pdt
%Transmittance
Wavenumbers (cm-1)
105
100
95
90
85
80
75
70
65
60
55
50
45
40
35
30
25
4000
3500
3000
2500
2000
1500
1000
500

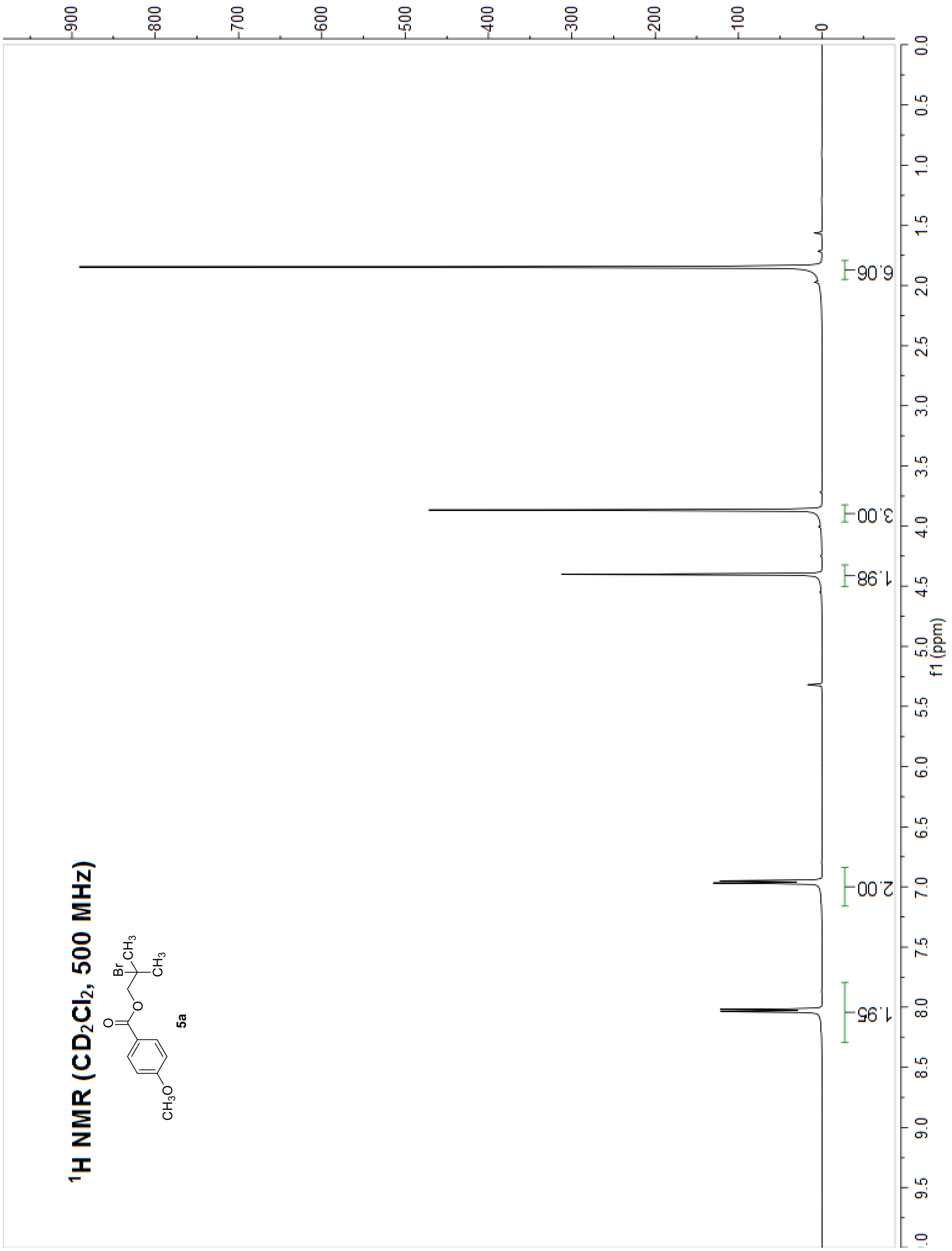
1H NMR (CD2Cl2, 500 MHz)
5a
6.06
3.00
1.98
2.00
1.95
f1 (ppm)

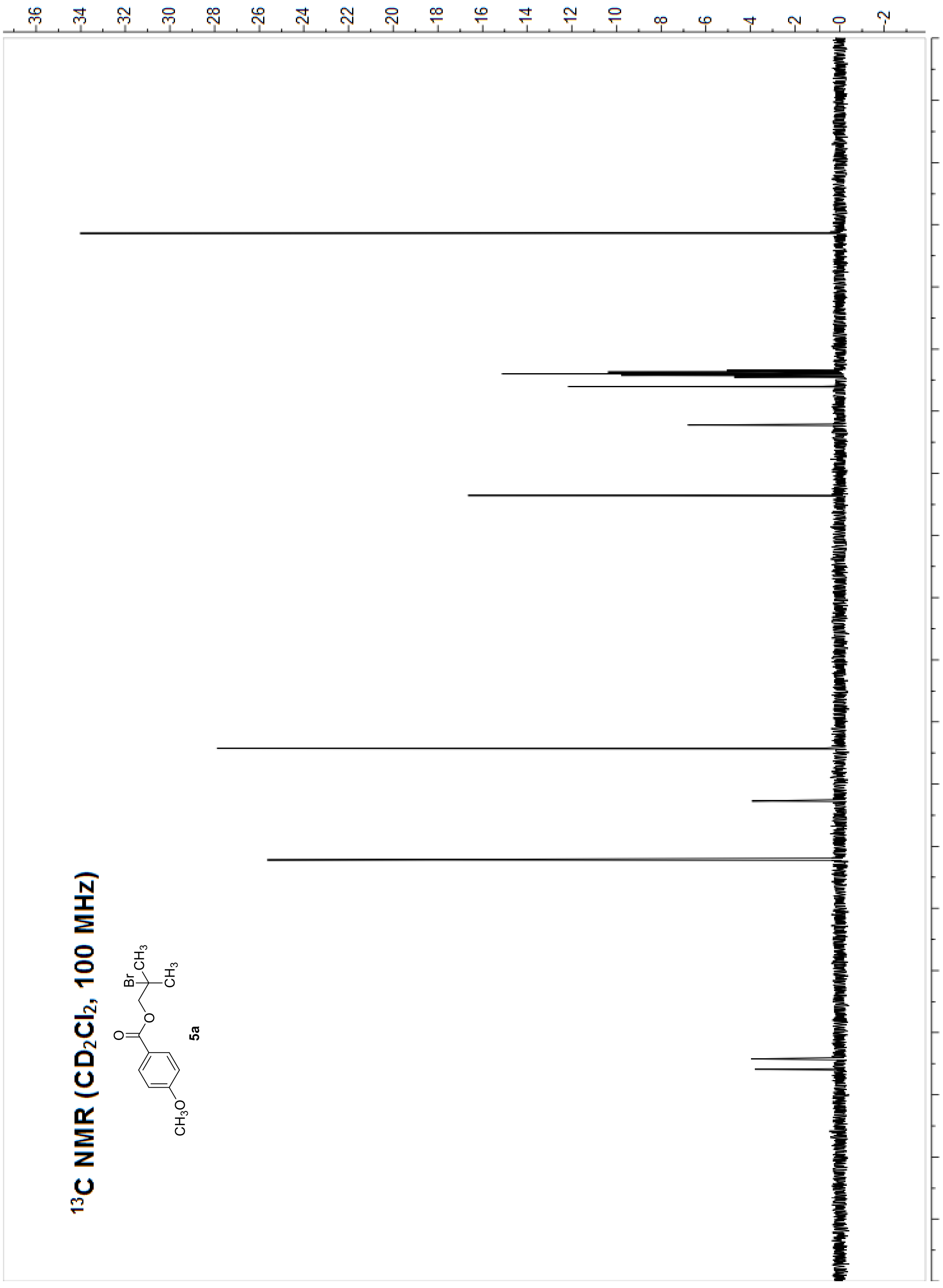

13C NMR (CD2Cl2, 100 MHz)
5a
f1 (ppm)

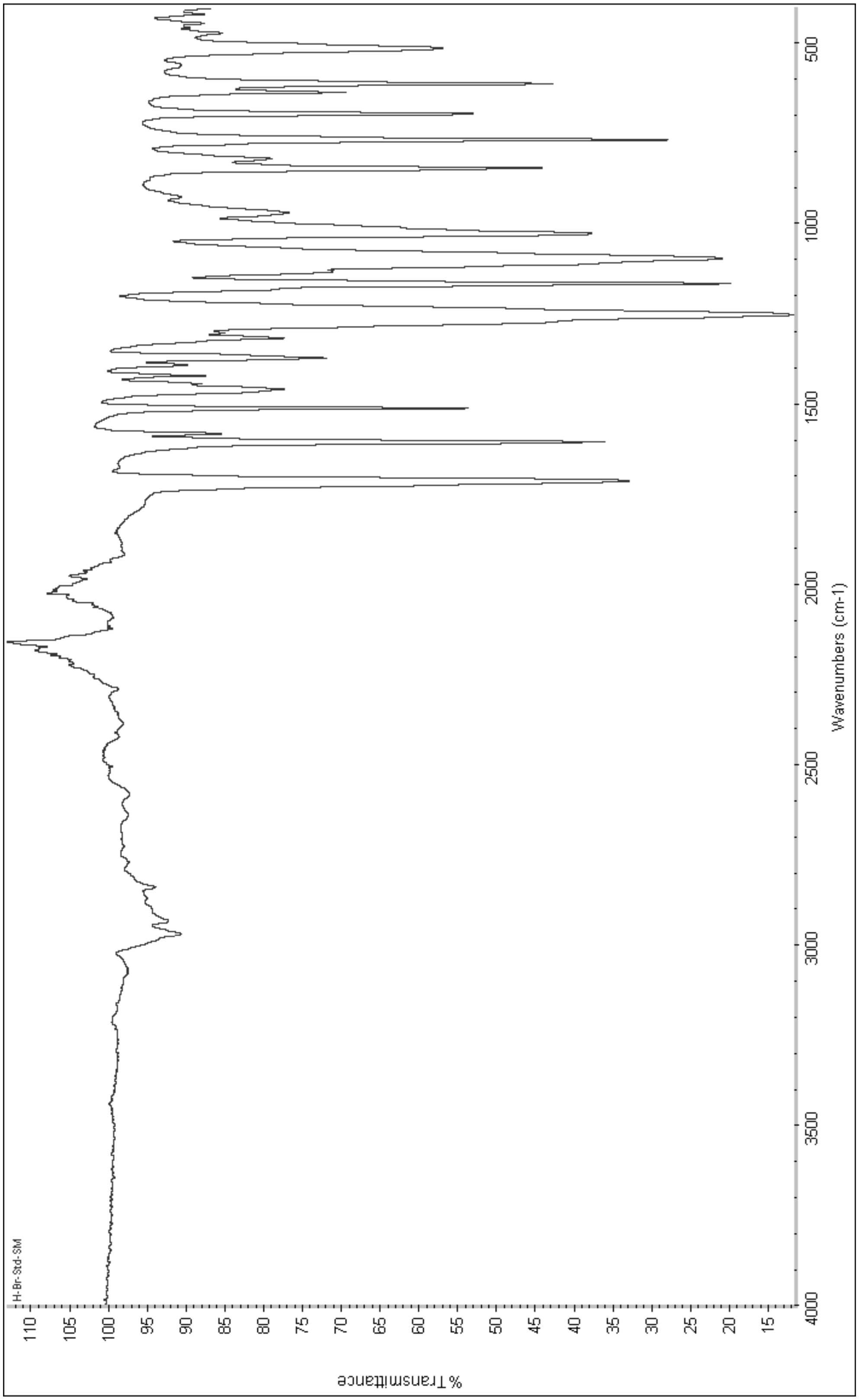
H-Br-Std-SM
%Transmittance
Wavenumbers (cm-1)
110 105 100 95 90 85 80 75 70 65 60 55 50 45 40 35 30 25 20 15
4000 3500 3000 2500 2000 1500 1000 500

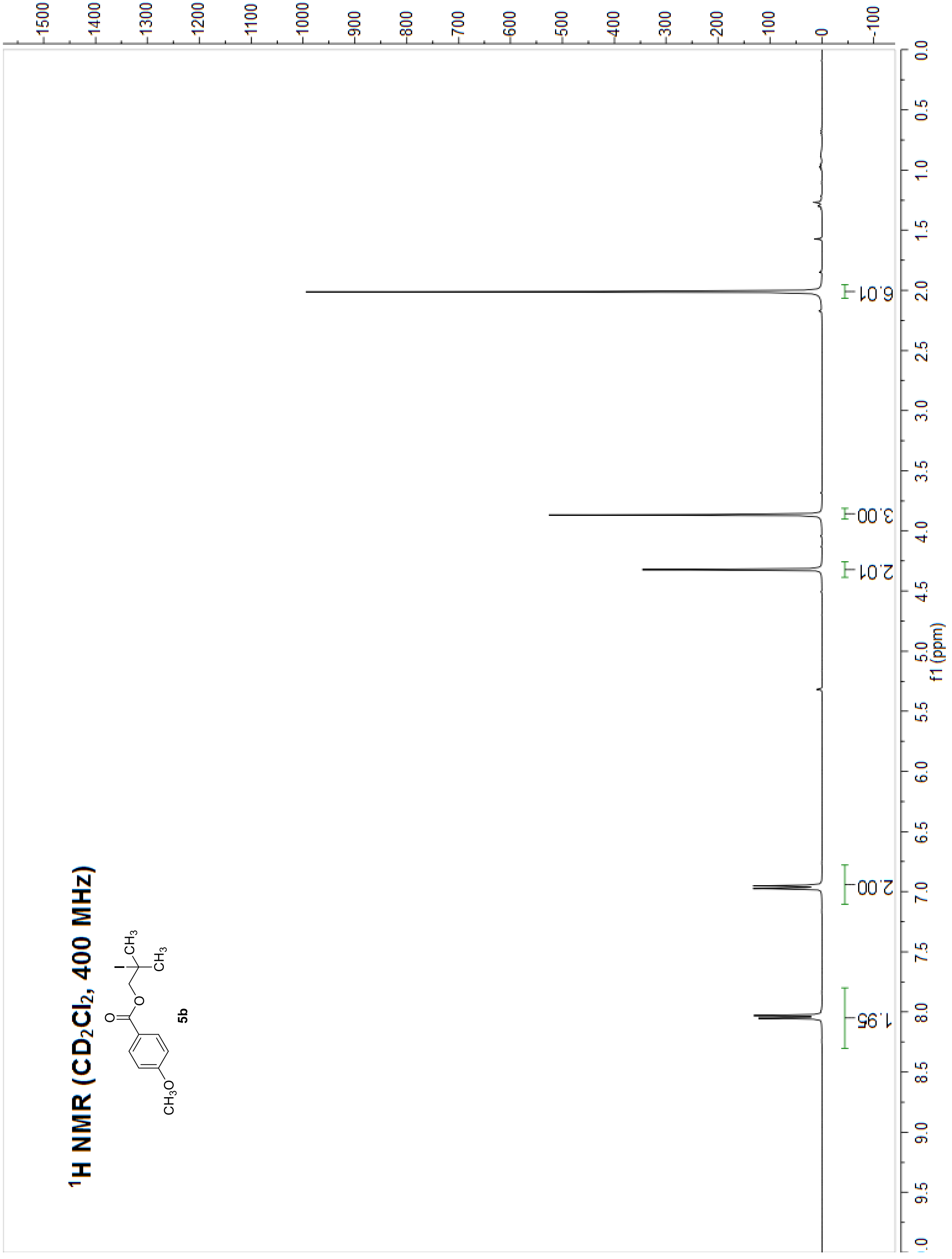
1H NMR (CD2Cl2, 400 MHz)
CH3O
O
O
I
CH3
CH3
5b
6.01
3.00
2.01
2.00
1.95
f1 (ppm)

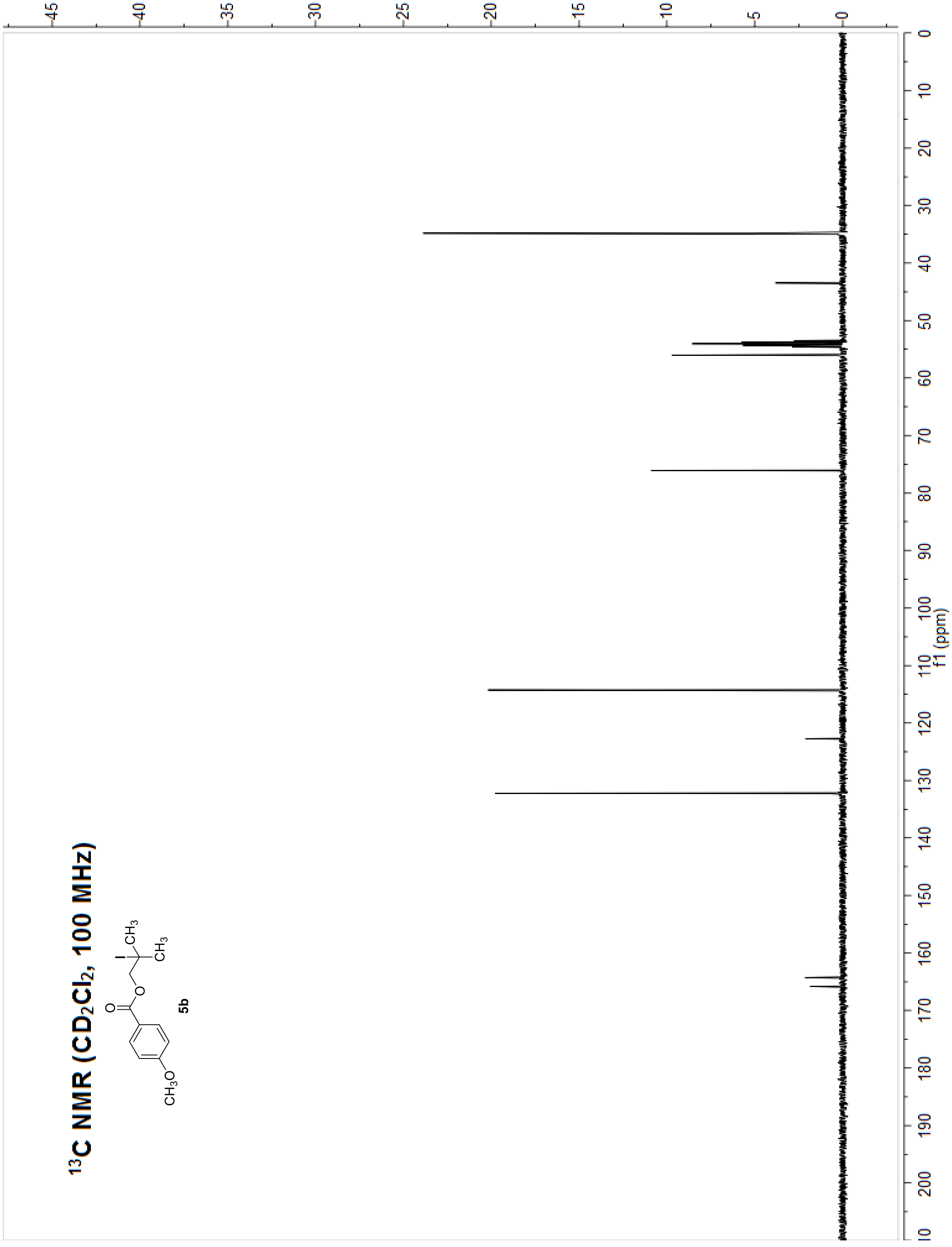

13C NMR (CD2Cl2, 100 MHz)
5b
f1 (ppm)

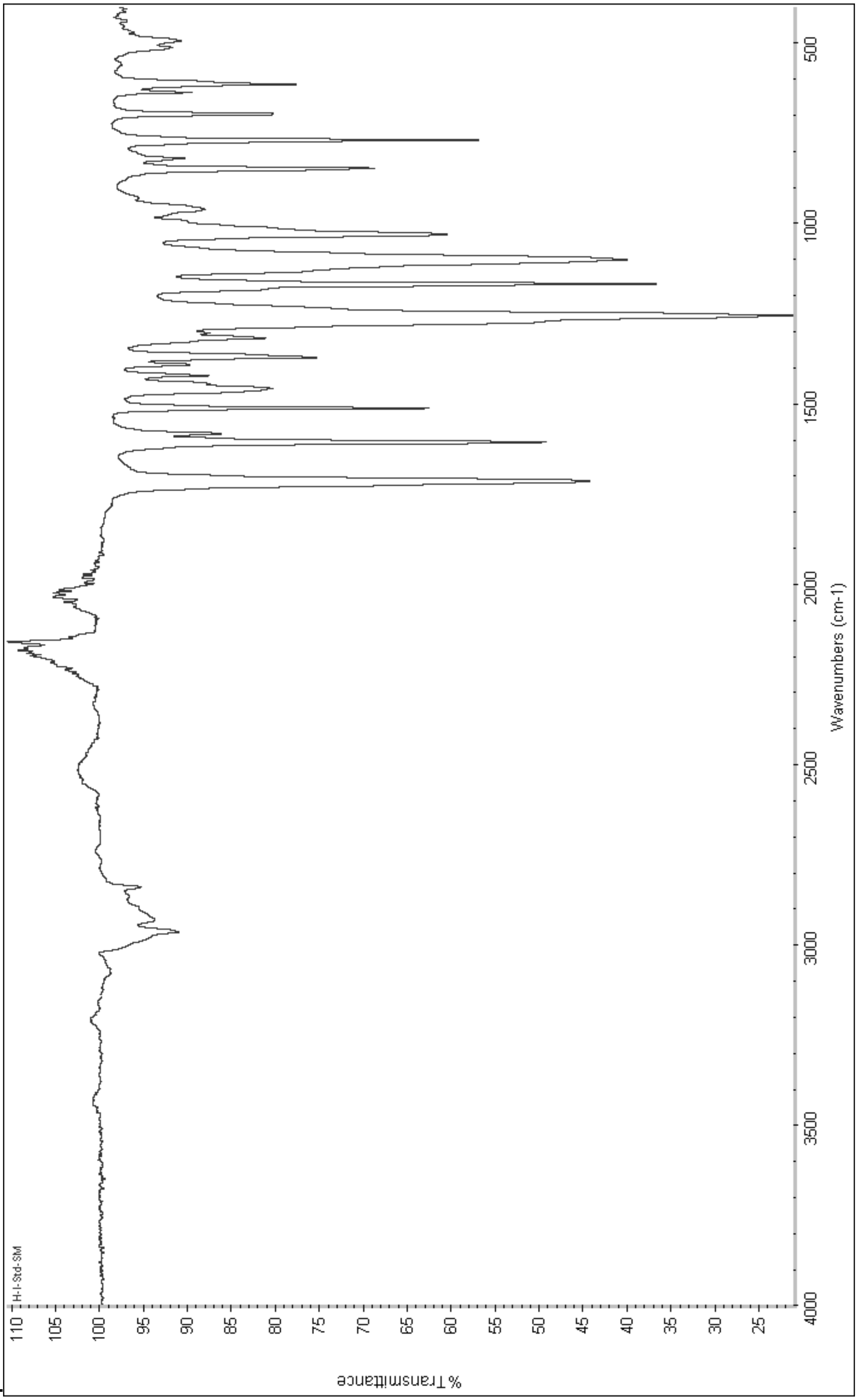
H-I-Std-SM
%Transmittance
Wavenumbers (cm-1)

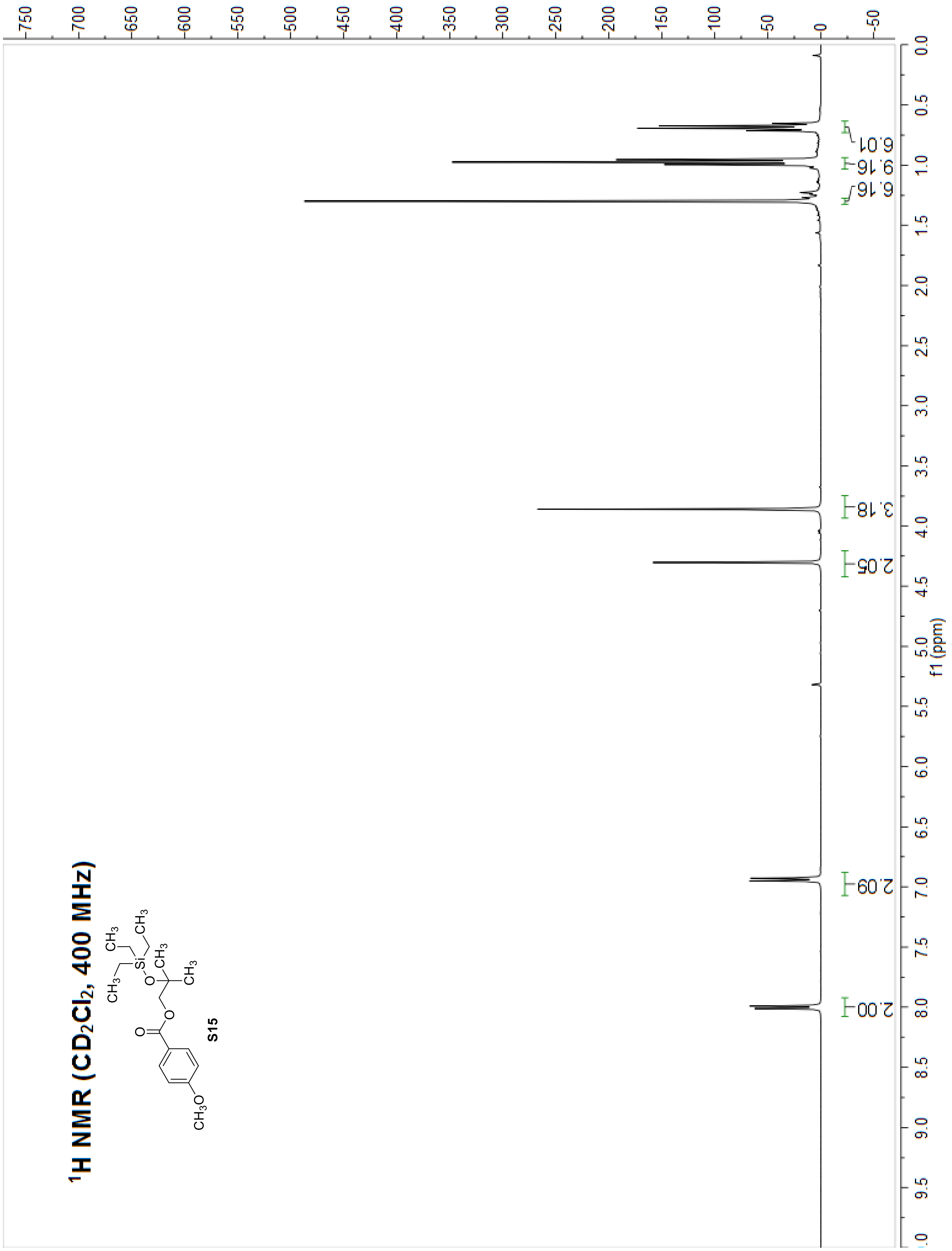

1H NMR (CD2Cl2, 400 MHz)
S15
f1 (ppm)

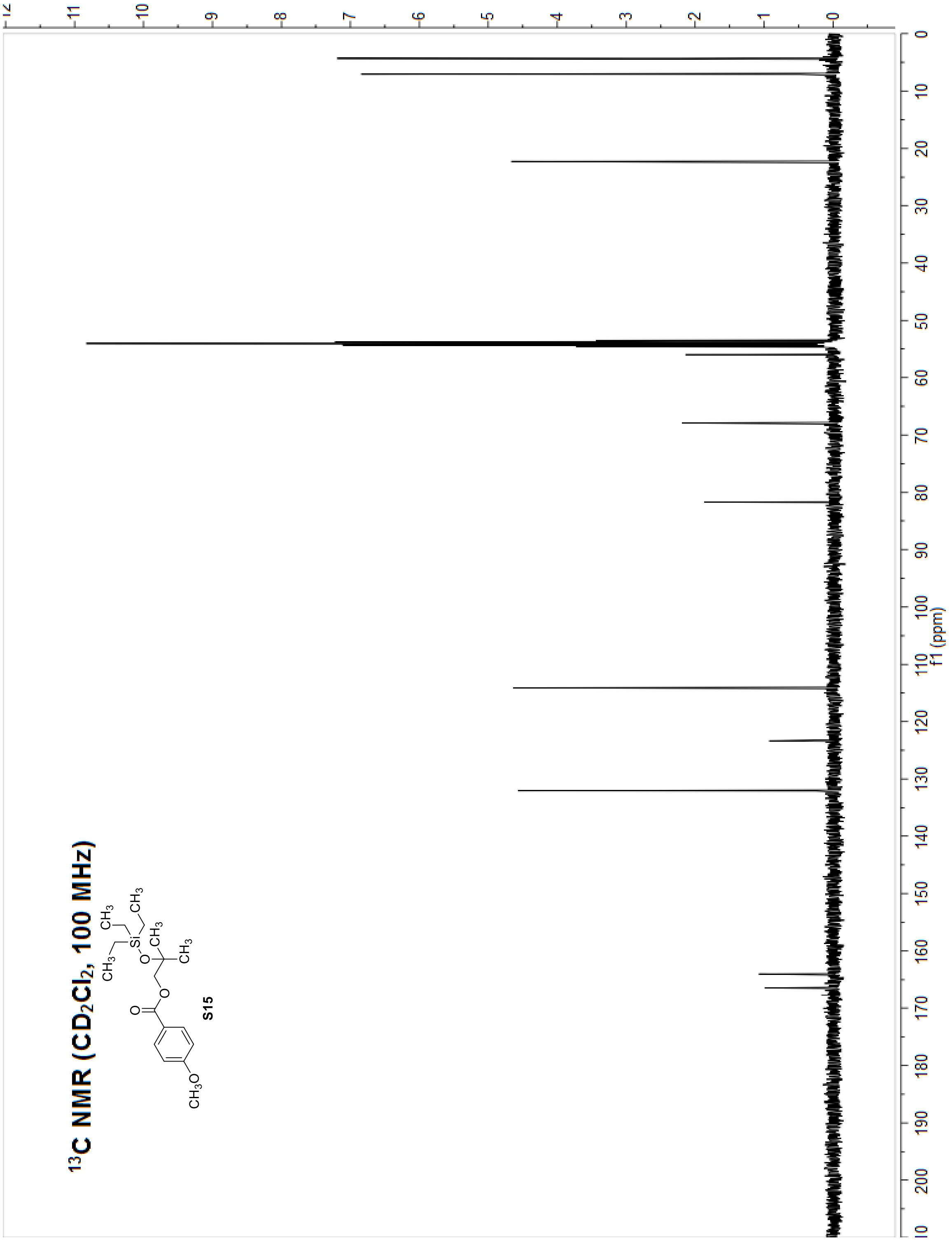

^{13}C NMR (CD$_2$Cl$_2$, 100 MHz)
S15
f1 (ppm)

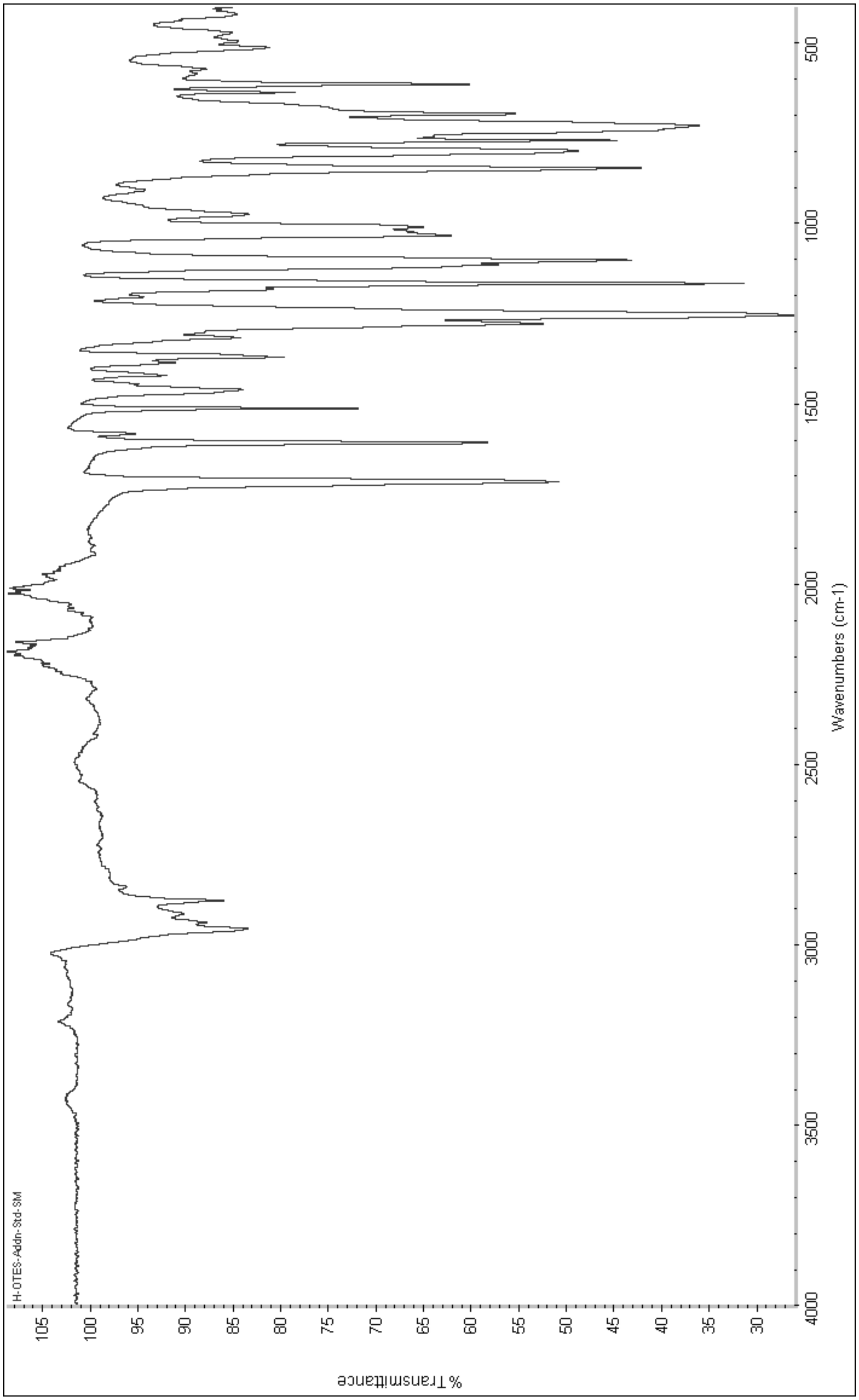
H-OTES-Addn-Std-SM
%Transmittance
Wavenumbers (cm-1)
4000
3500
3000
2500
2000
1500
1000
500
105
100
95
90
85
80
75
70
65
60
55
50
45
40
35
30

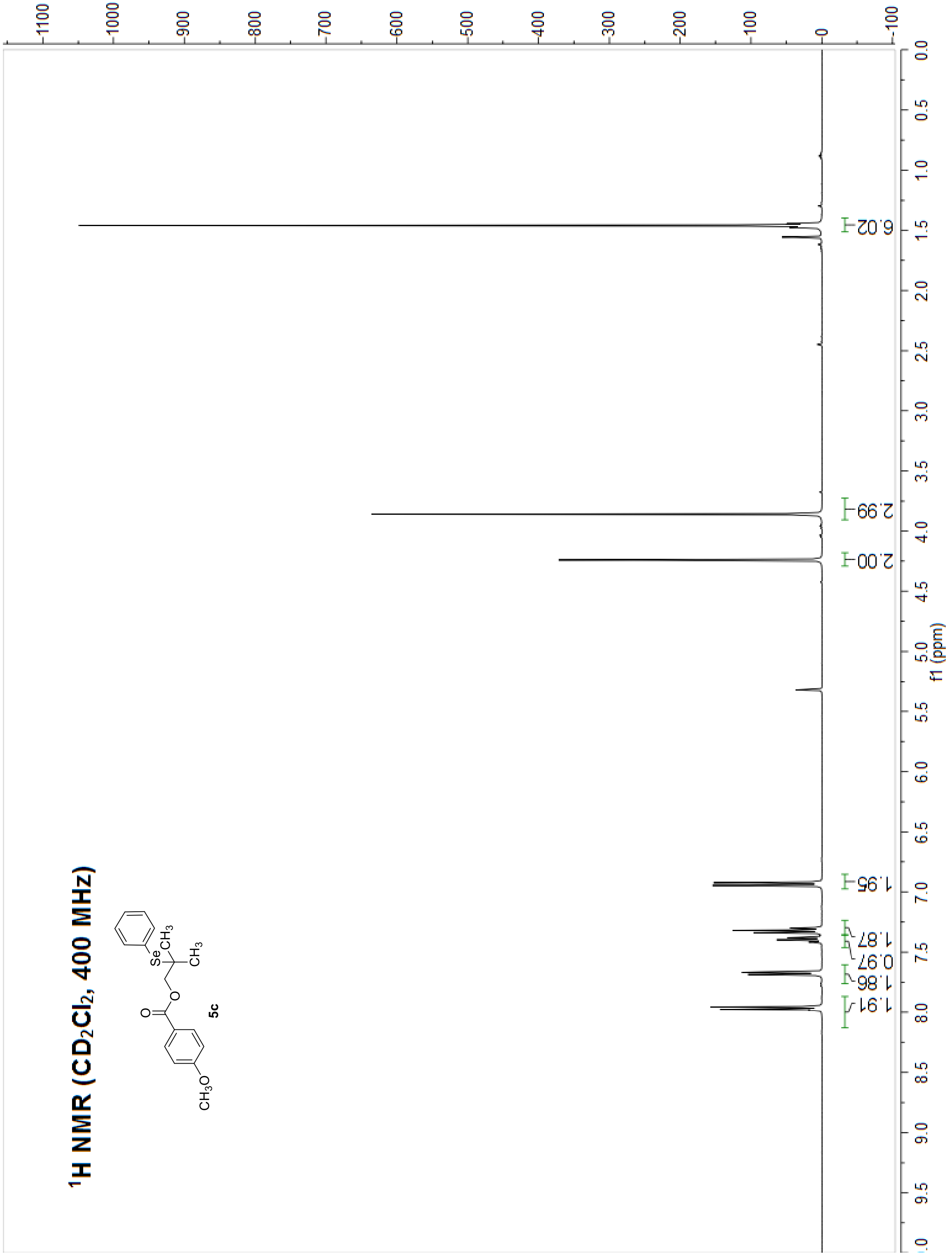
1H NMR (CD2Cl2, 400 MHz)
5c
f1 (ppm)
6.02
2.99
2.00
1.95
1.87
0.97
1.86
1.91

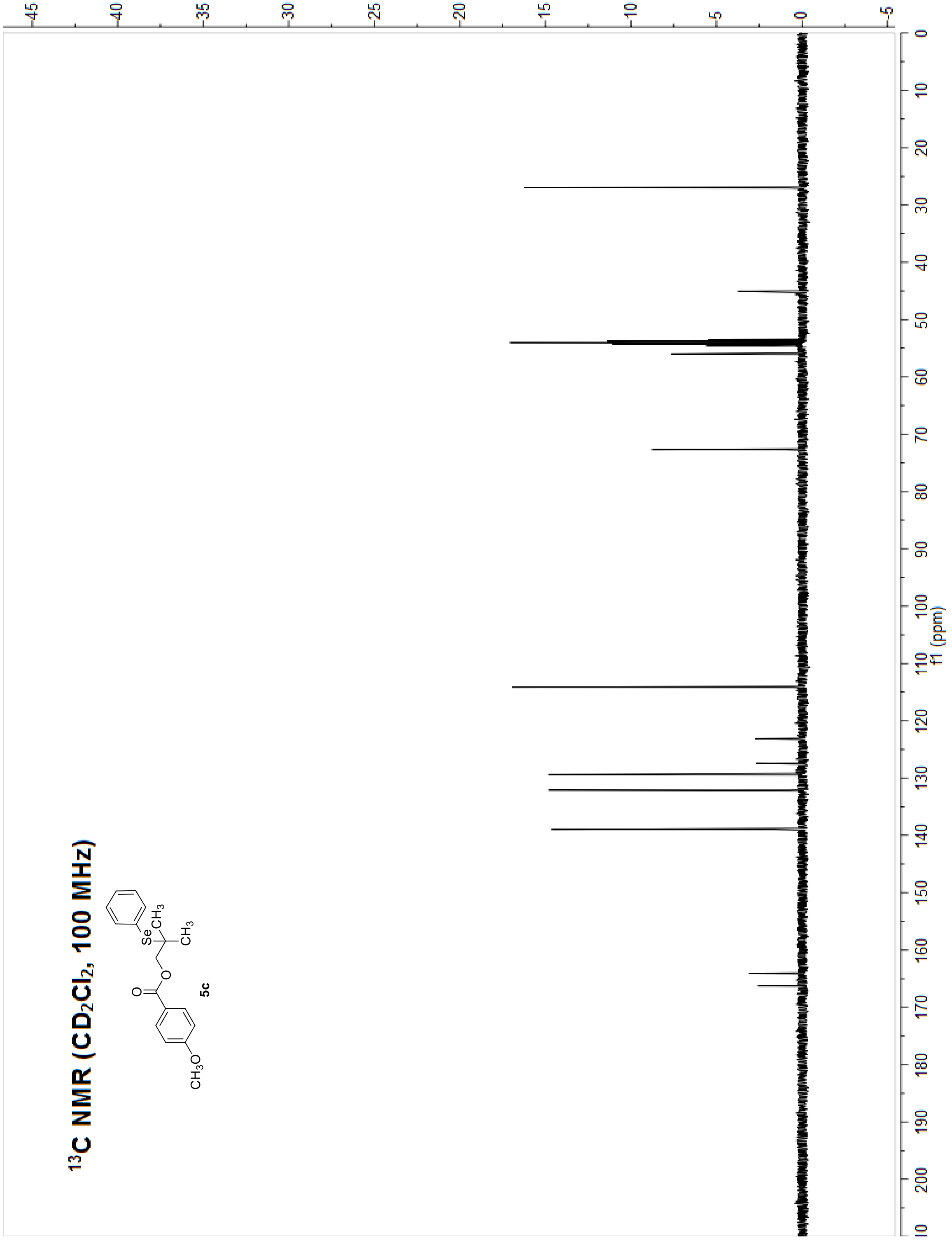

^{13}C NMR (CD_2Cl_2, 100 MHz)
5c
f1 (ppm)

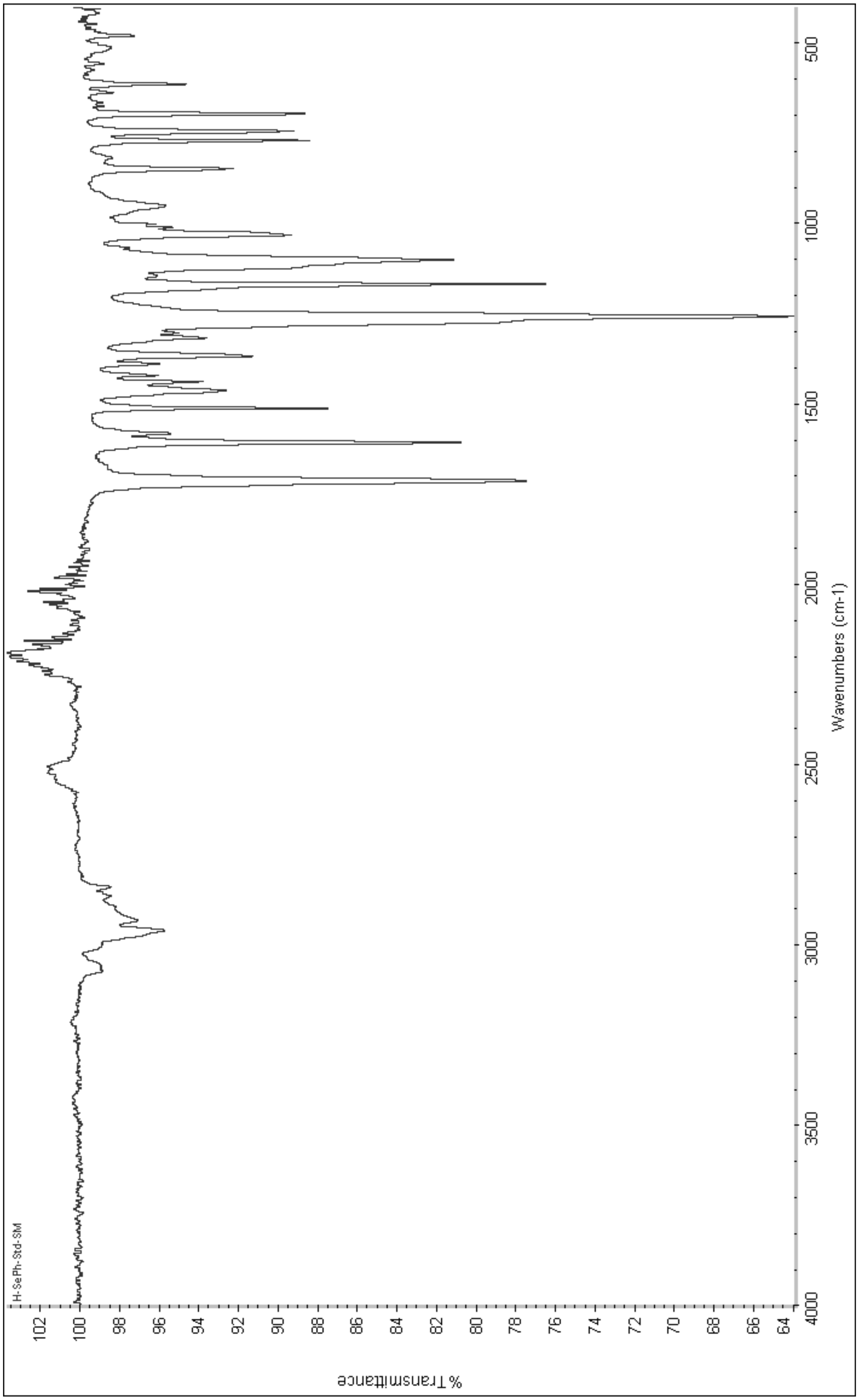
H-SePh-Std-SM
%Transmittance
Wavenumbers (cm-1)
4000
3500
3000
2500
2000
1500
1000
500
102
100
98
96
94
92
90
88
86
84
82
80
78
76
74
72
70
68
66
64

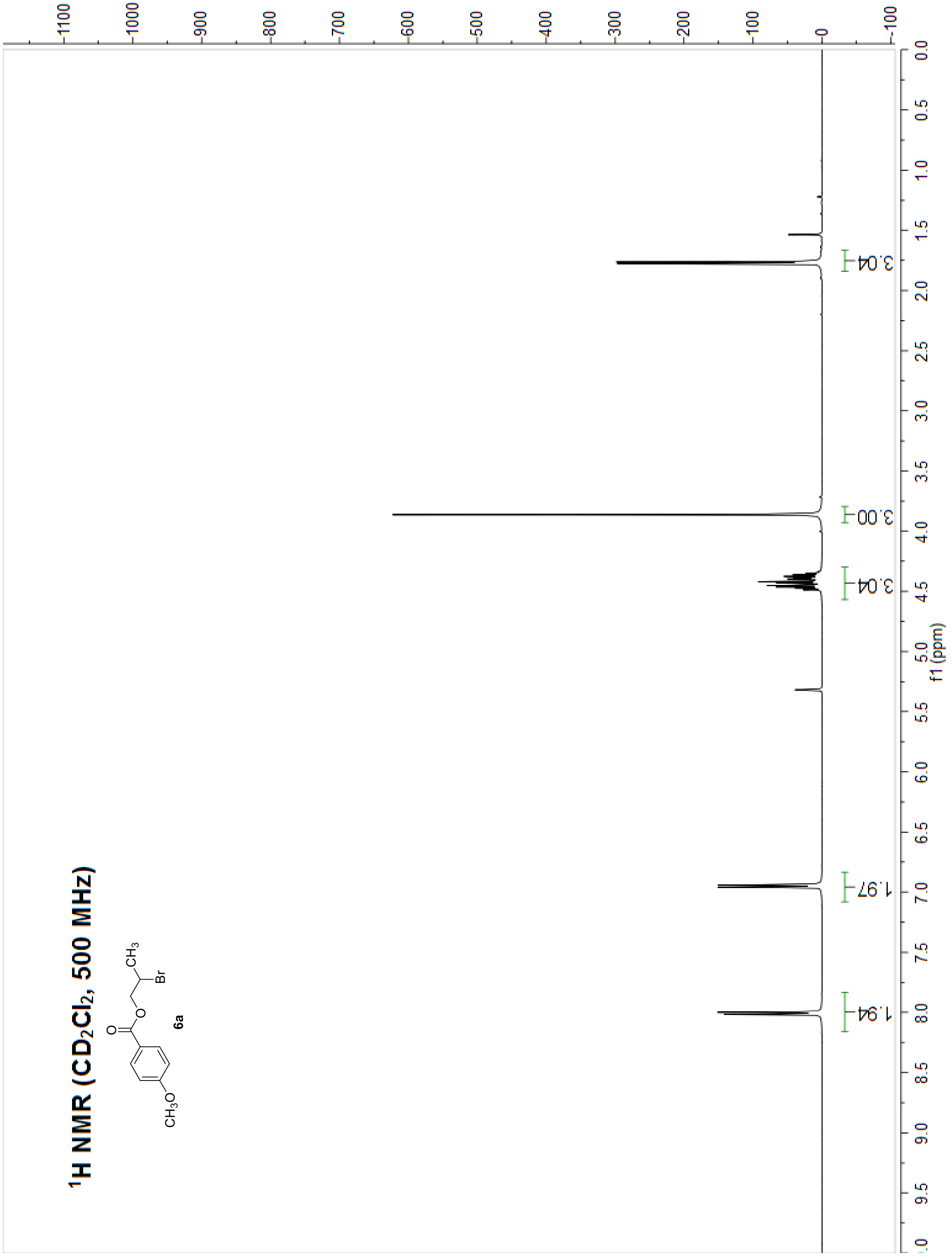
1H NMR (CD2Cl2, 500 MHz)
6a
f1 (ppm)

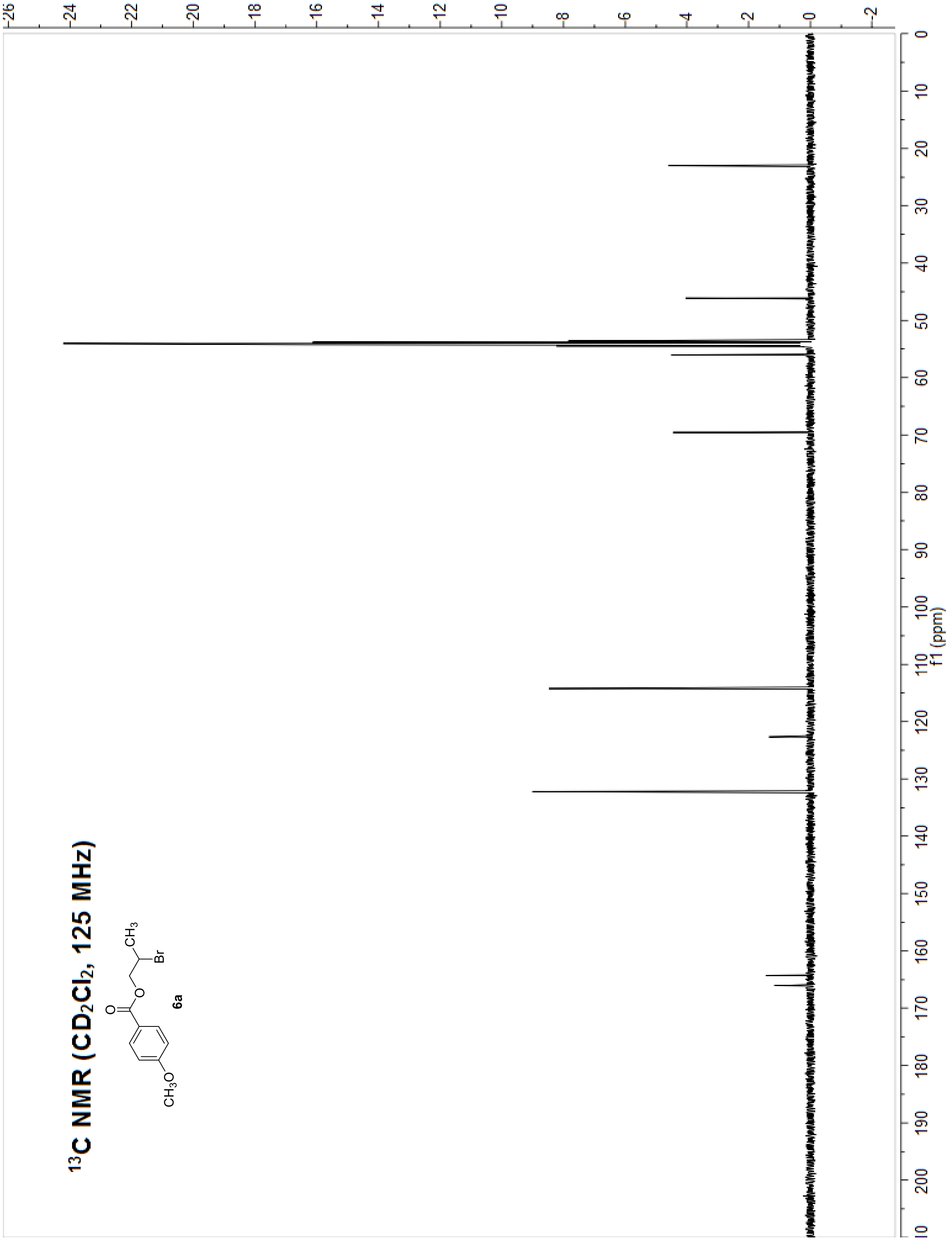

^{13}C NMR (CD_2Cl_2, 125 MHz)
CH_3O
O
O
CH_3
Br
6a
f1 (ppm)

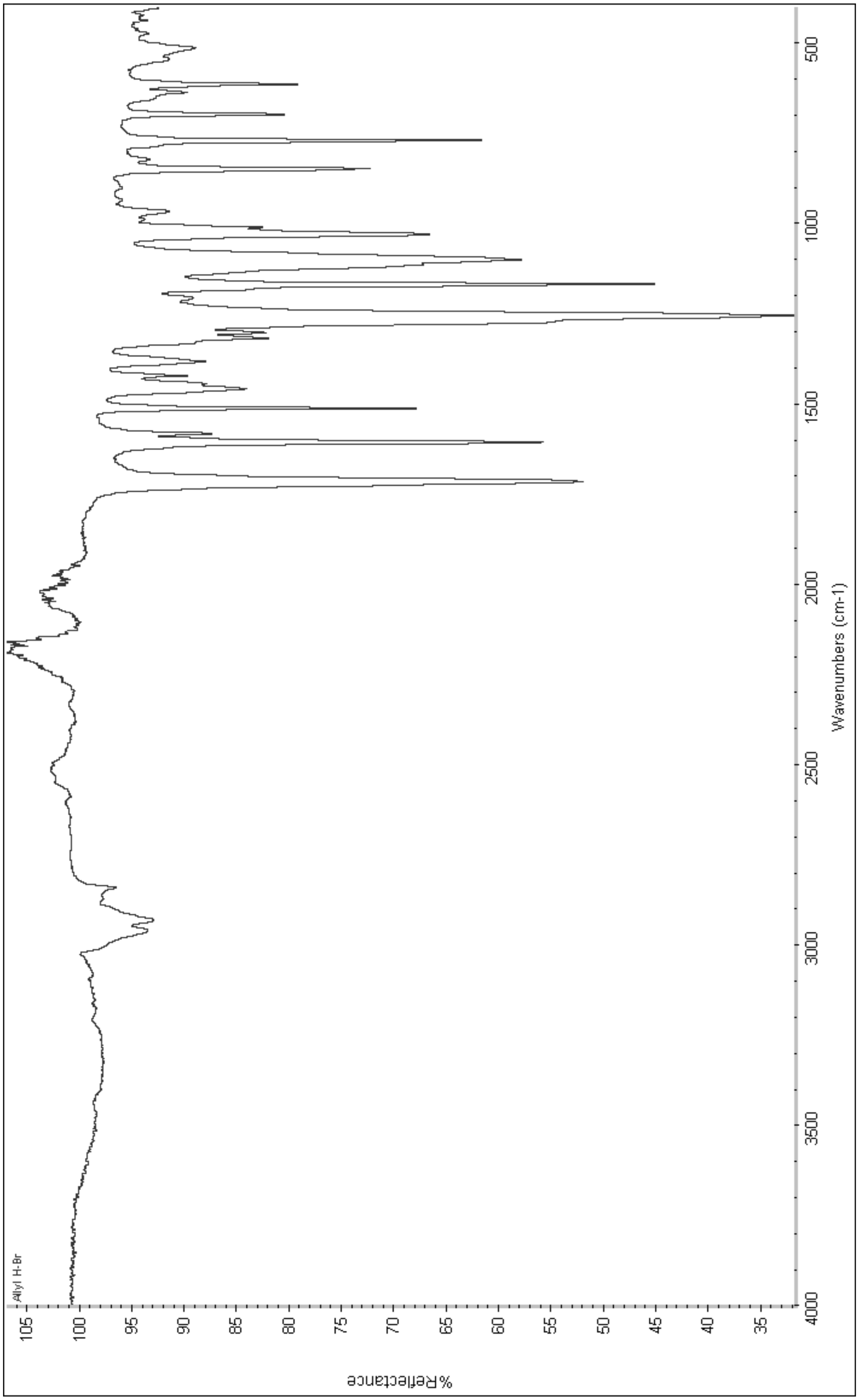
Allyl H-Br
%Reflectance
Wavenumbers (cm-1)

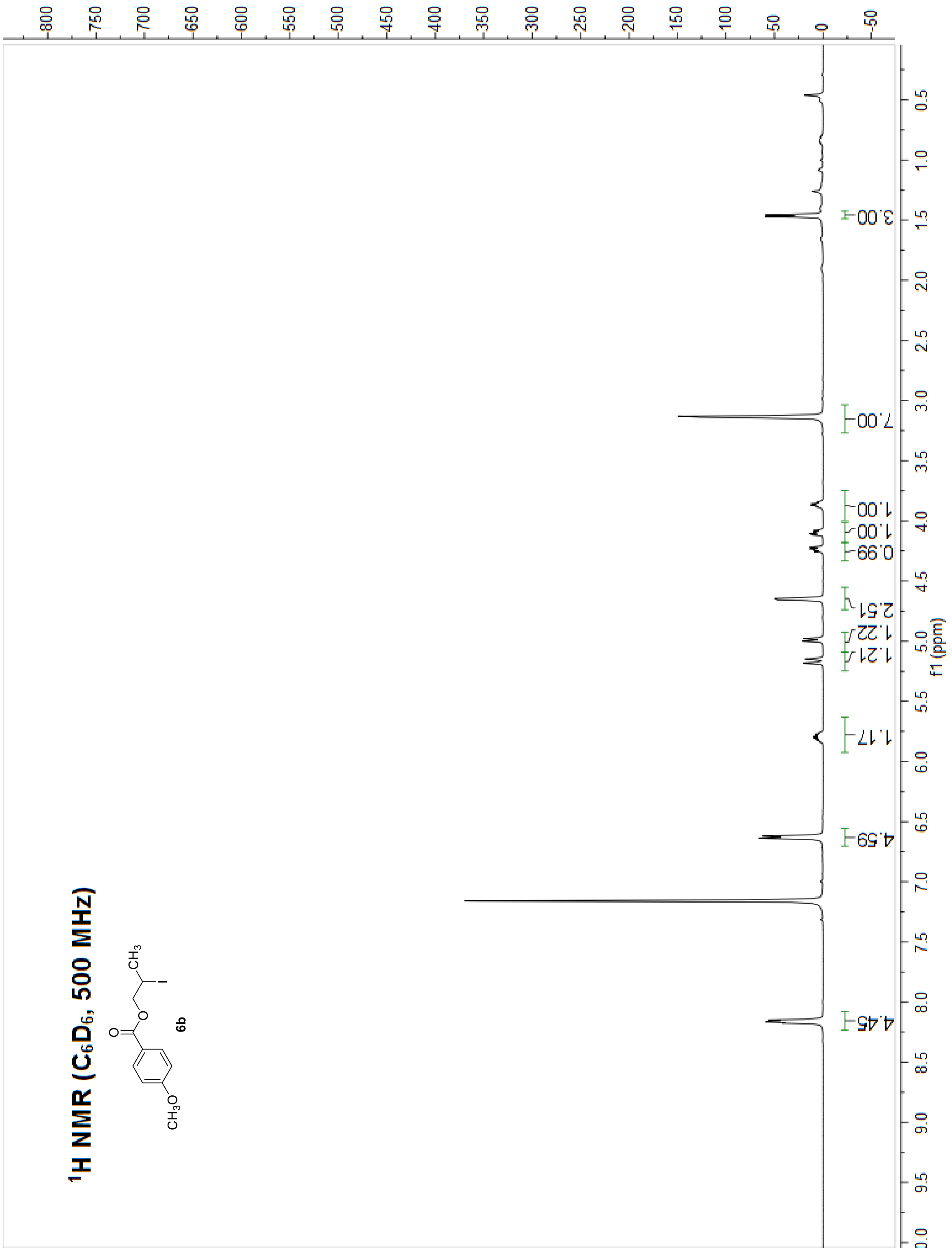

^1H NMR (C_6D_6, 500 MHz)
6b
f1 (ppm)

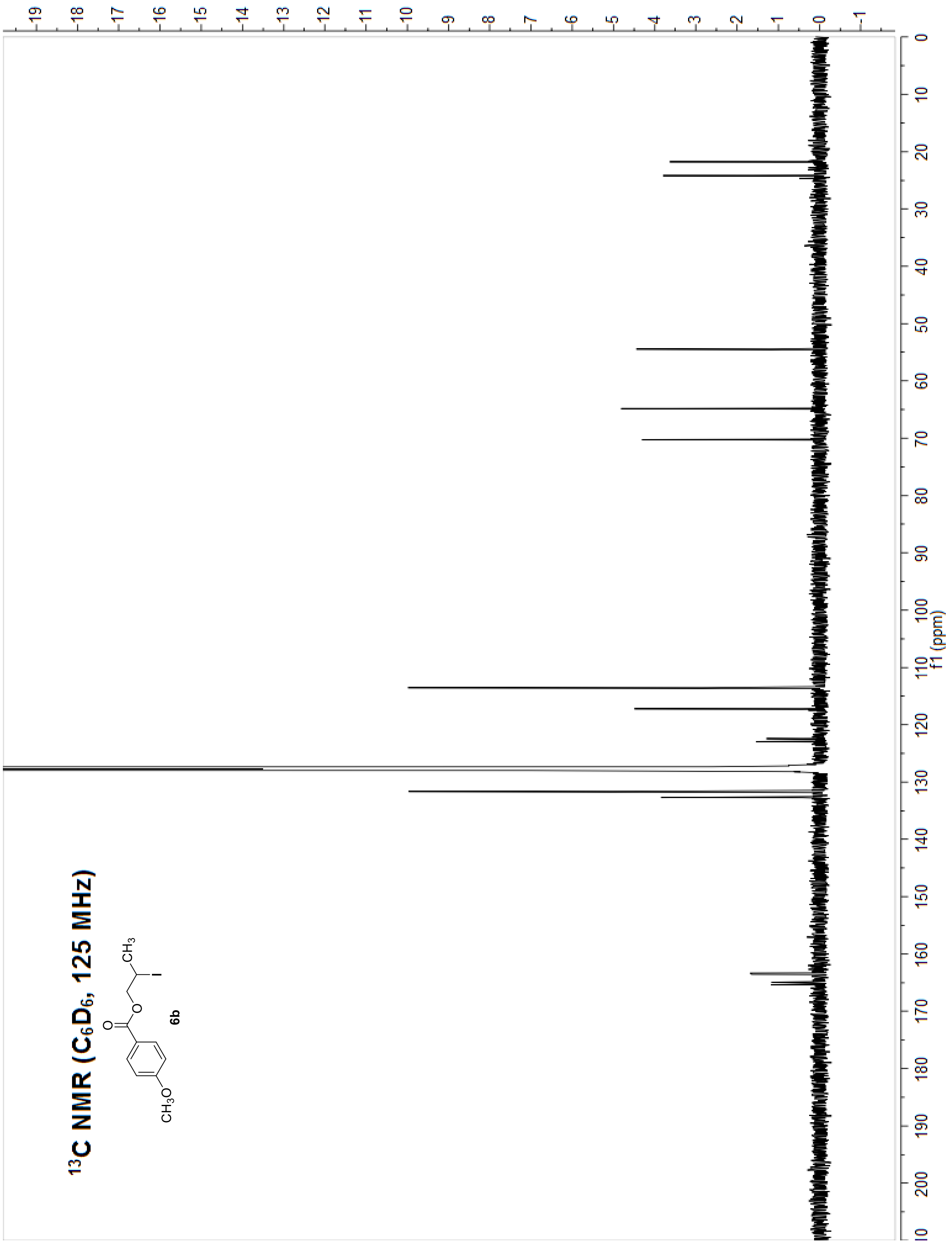
13C NMR (C6D6, 125 MHz)
6b
f1 (ppm)

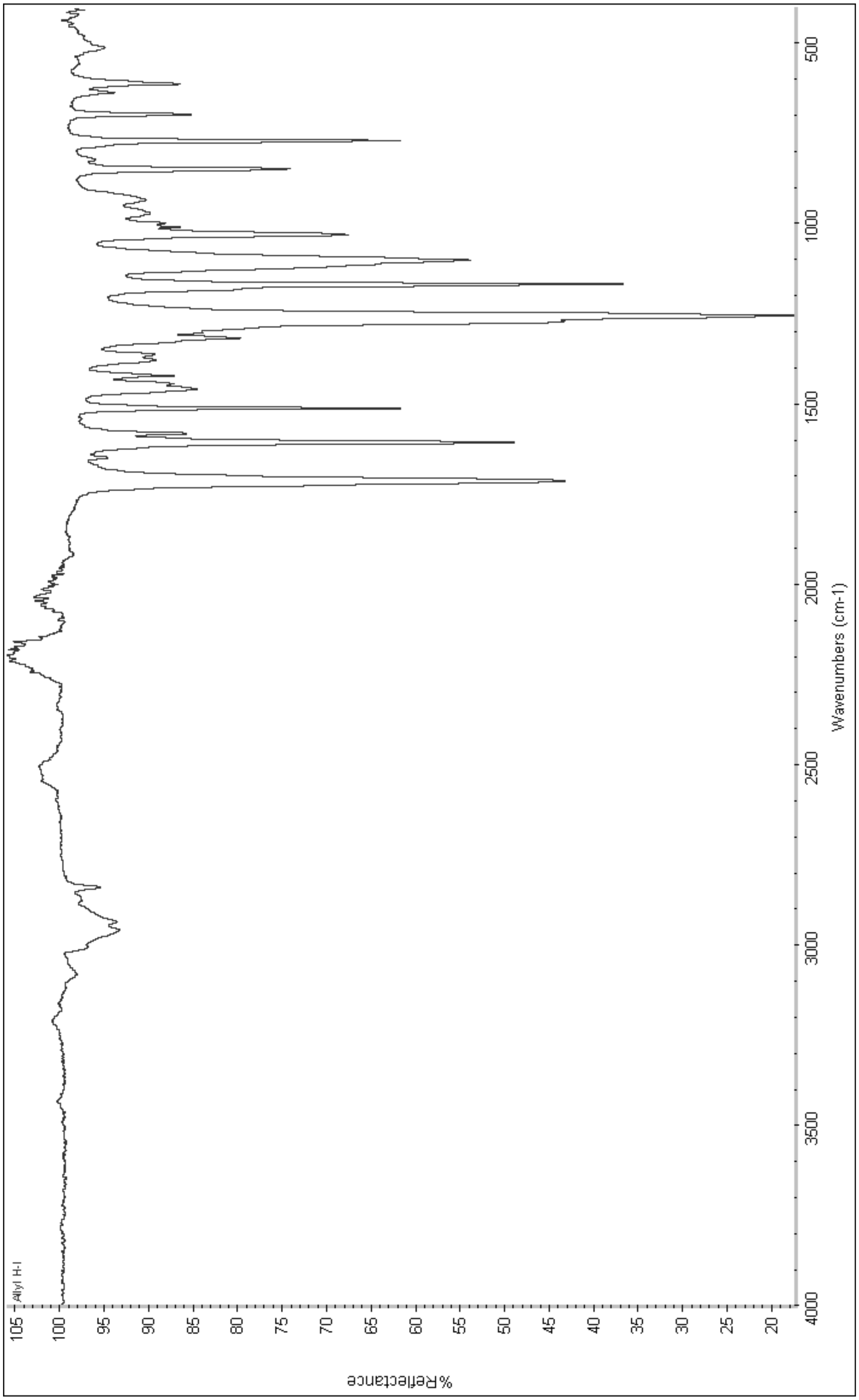
Allyl H-I
%Reflectance
Wavenumbers (cm-1)

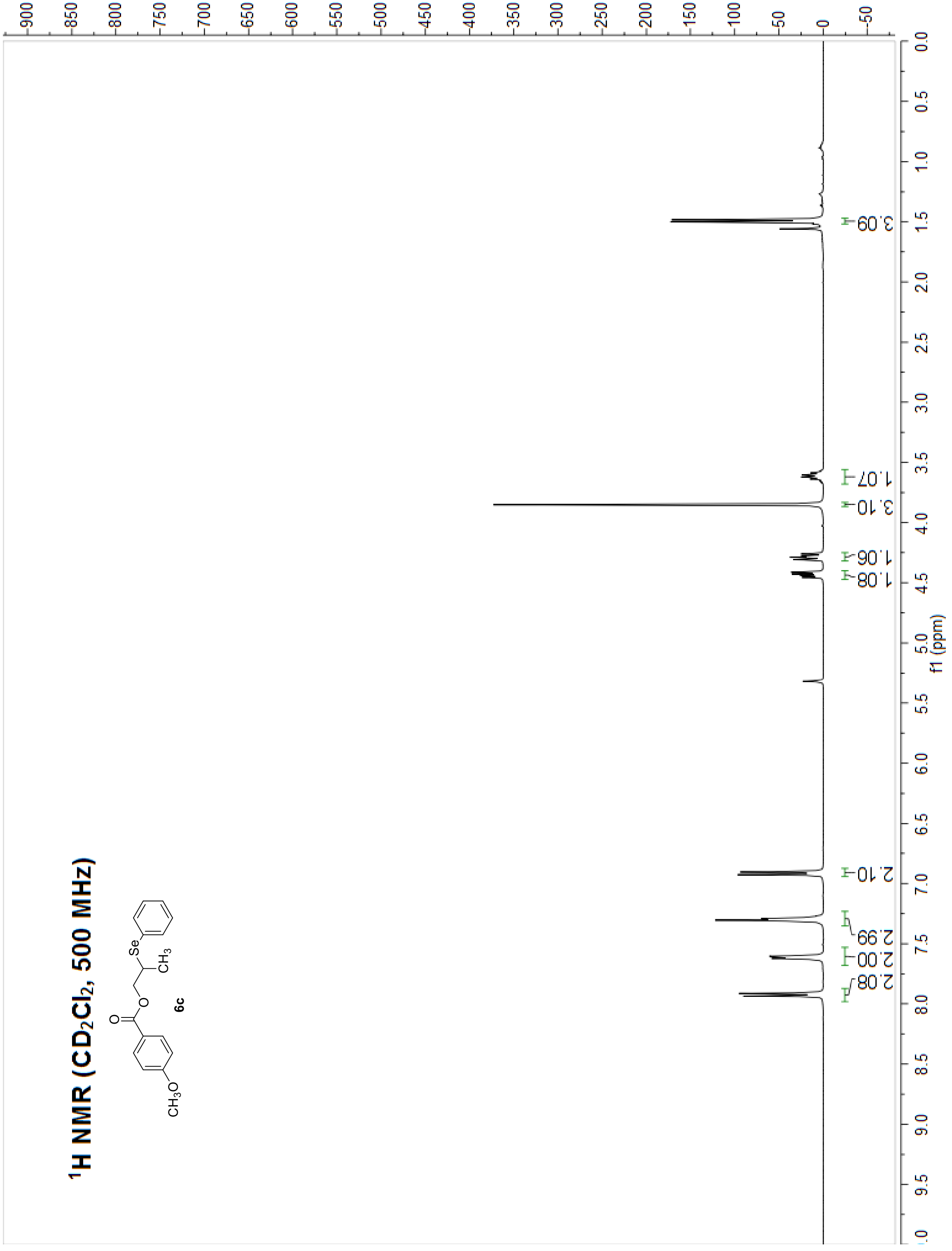
1H NMR (CD2Cl2, 500 MHz)
6c
f1 (ppm)
3.09
1.07
3.10
1.06
1.08
2.10
2.99
2.00
2.08

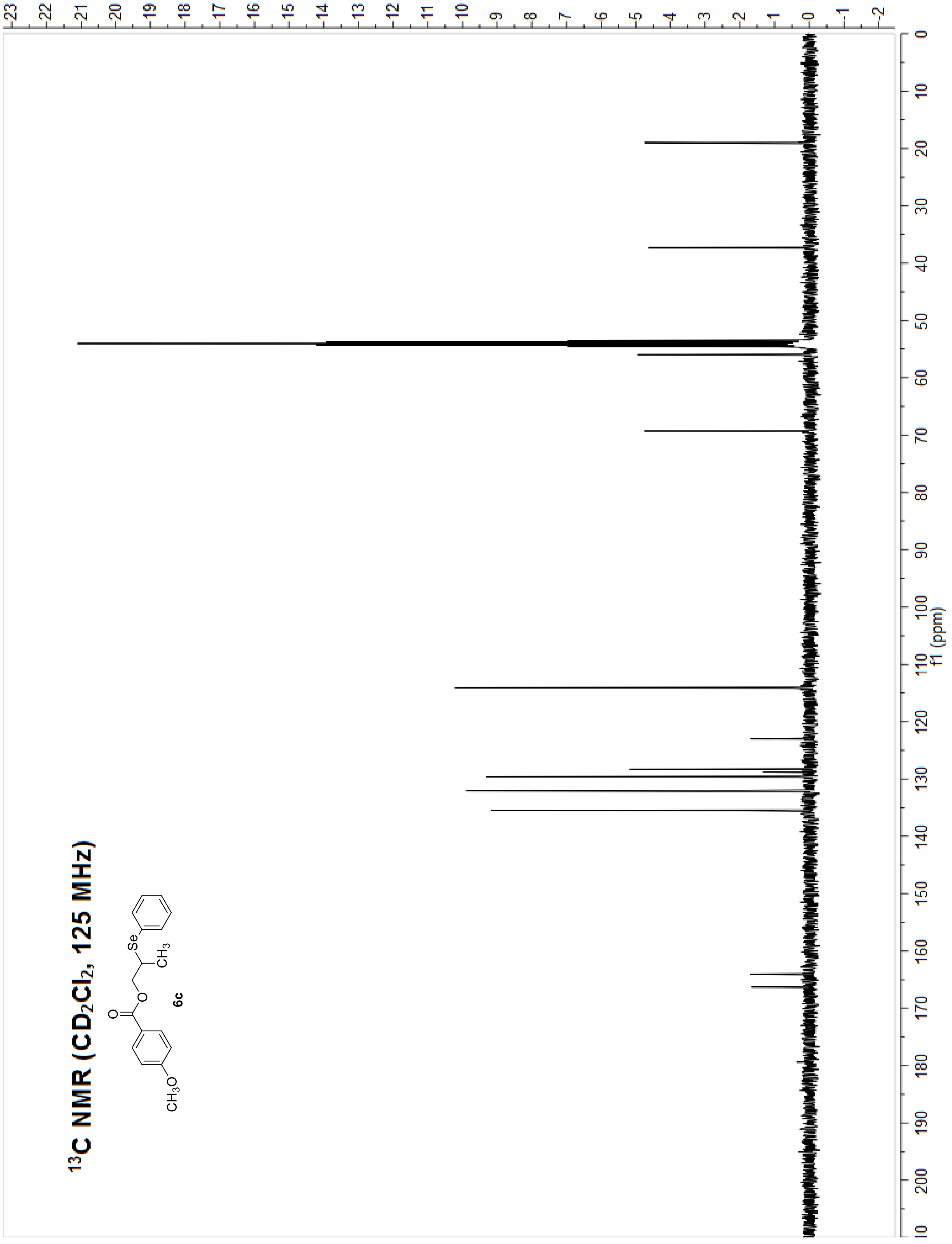

13C NMR (CD2Cl2, 125 MHz)
6c
f1 (ppm)

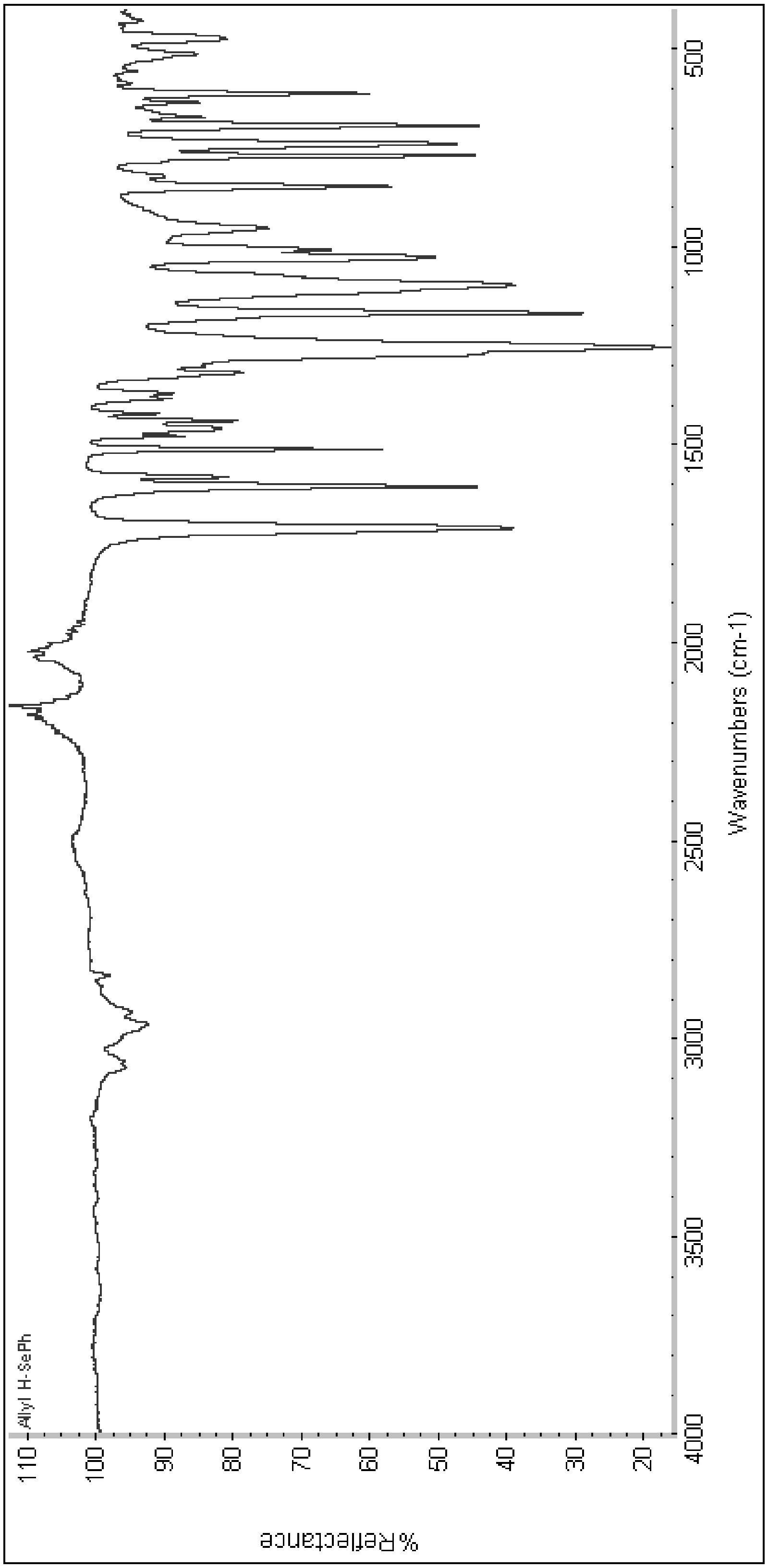
Allyl H-SePh
%Reflectance
Wavenumbers (cm-1)

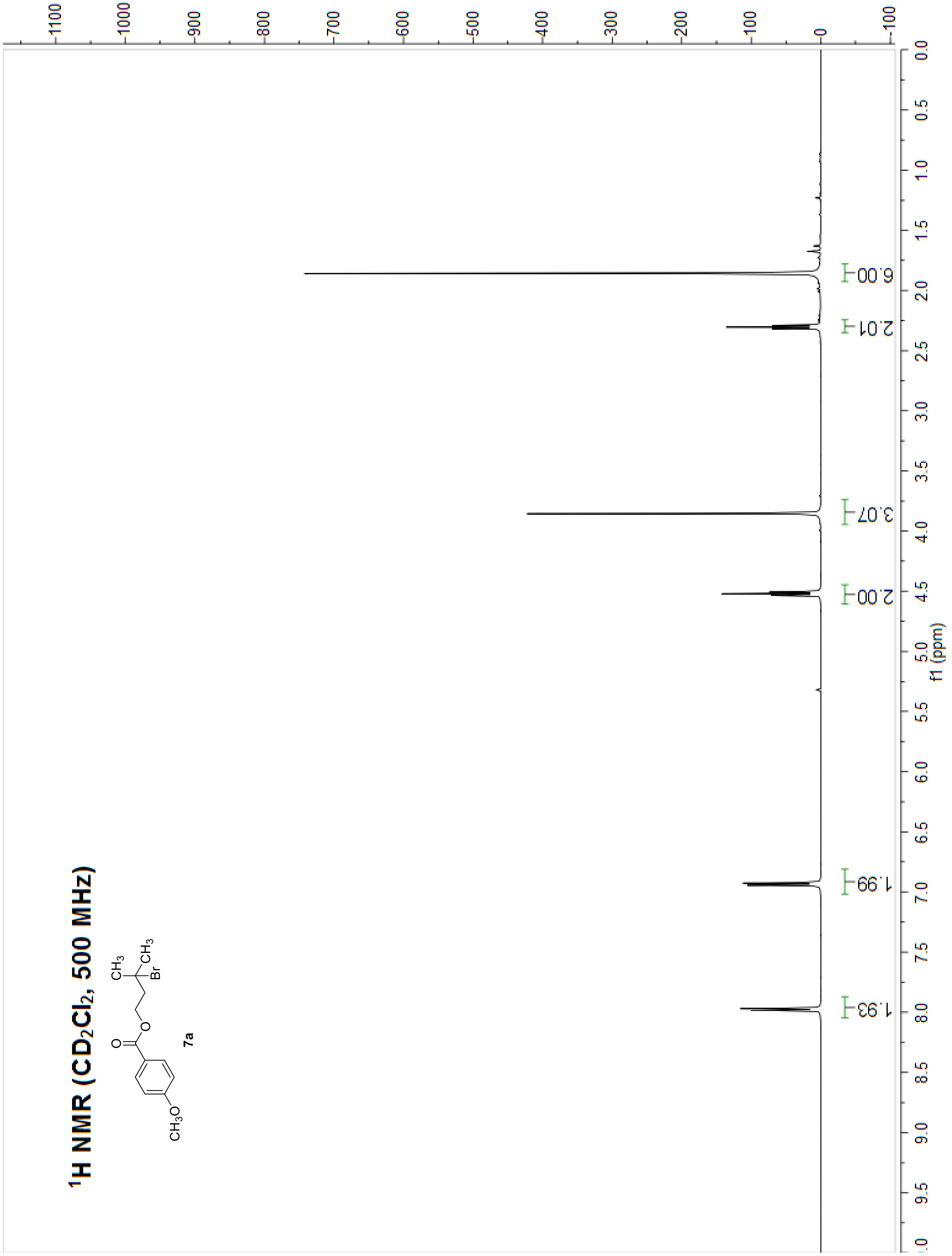
1H NMR (CD2Cl2, 500 MHz)
7a
6.00
2.01
3.07
2.00
1.99
1.93
f1 (ppm)

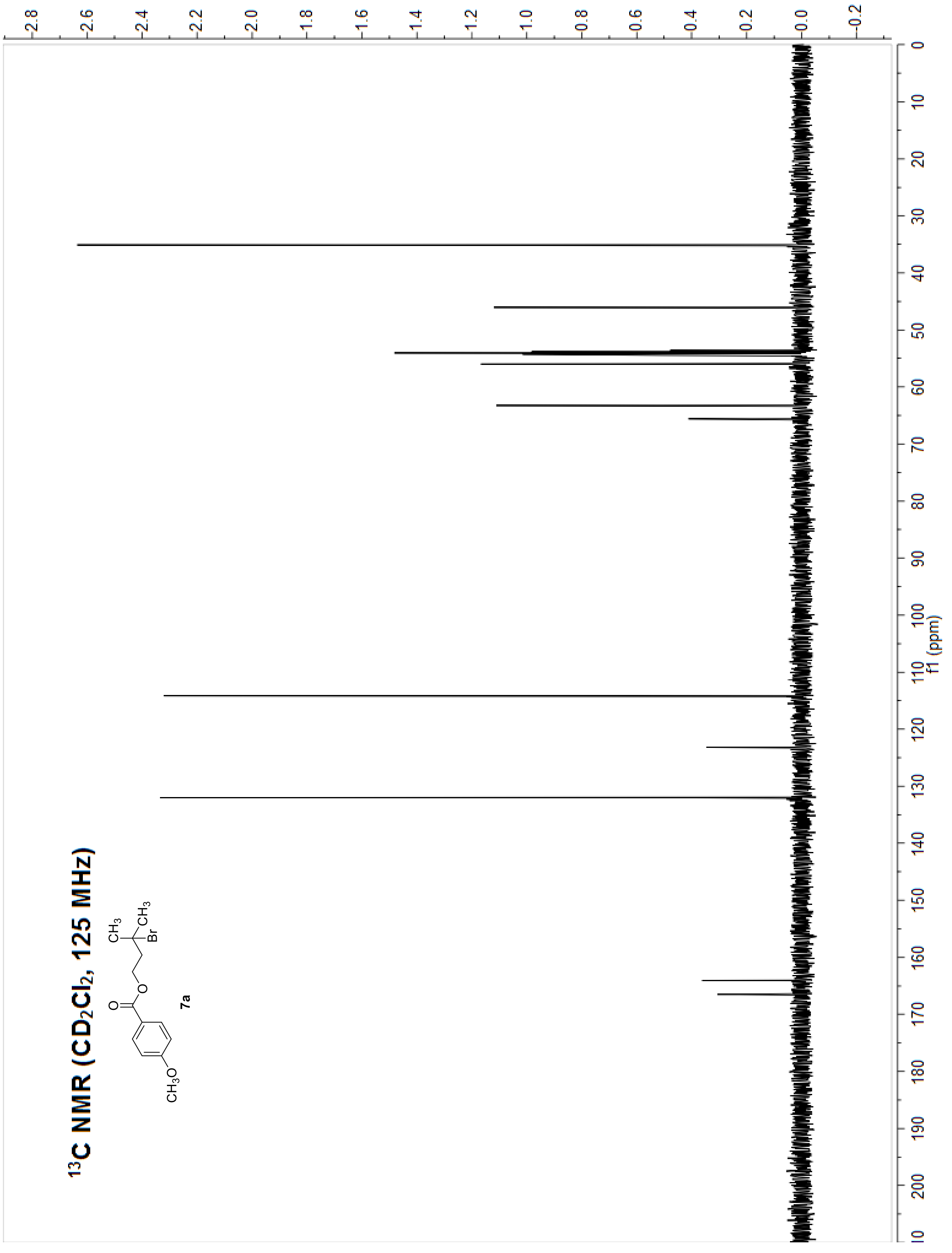
13C NMR (CD2Cl2, 125 MHz)
7a
CH3O
O
O
CH3
CH3
Br
f1 (ppm)

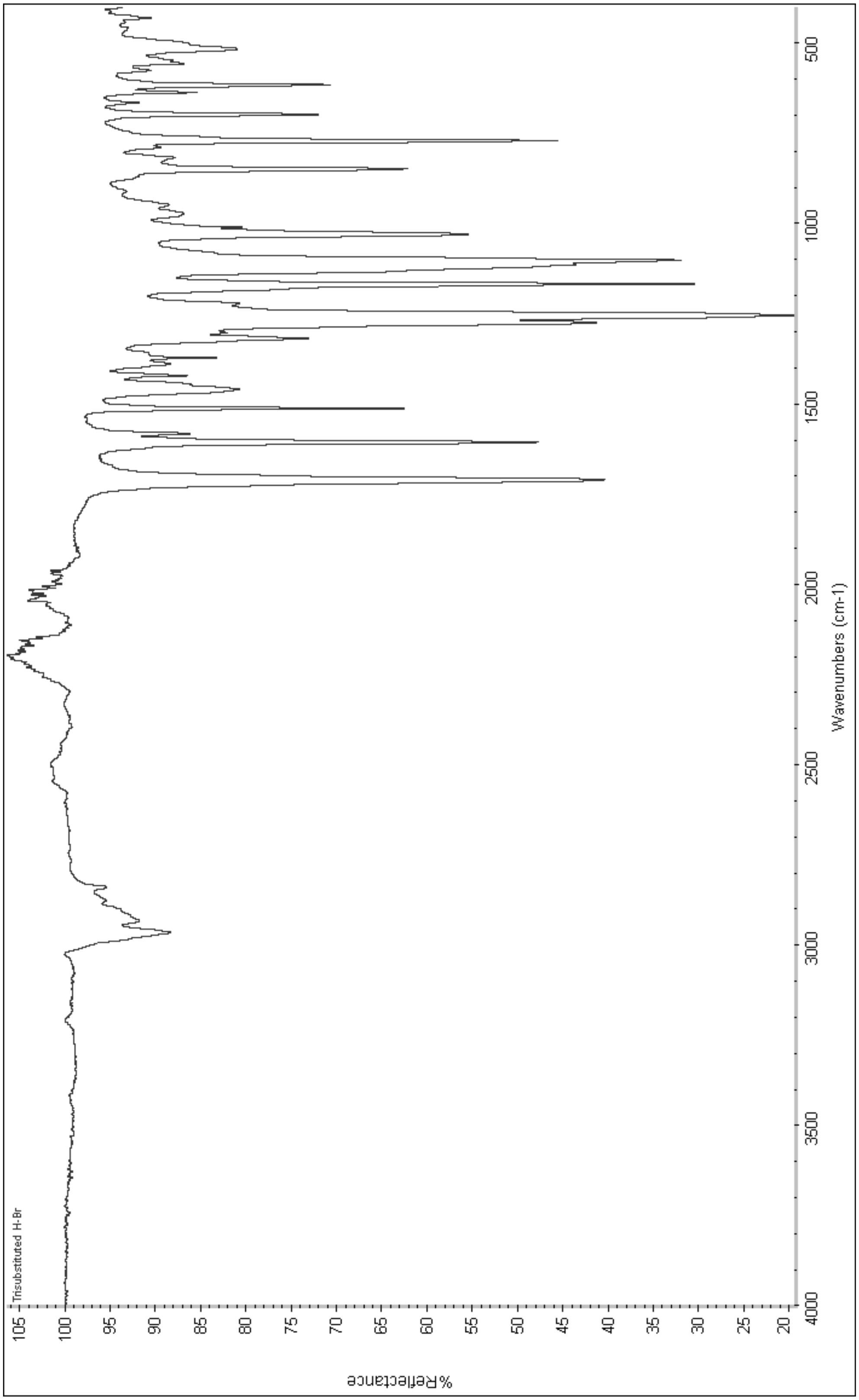
Trisubstituted H-Br
%Reflectance
Wavenumbers (cm-1)

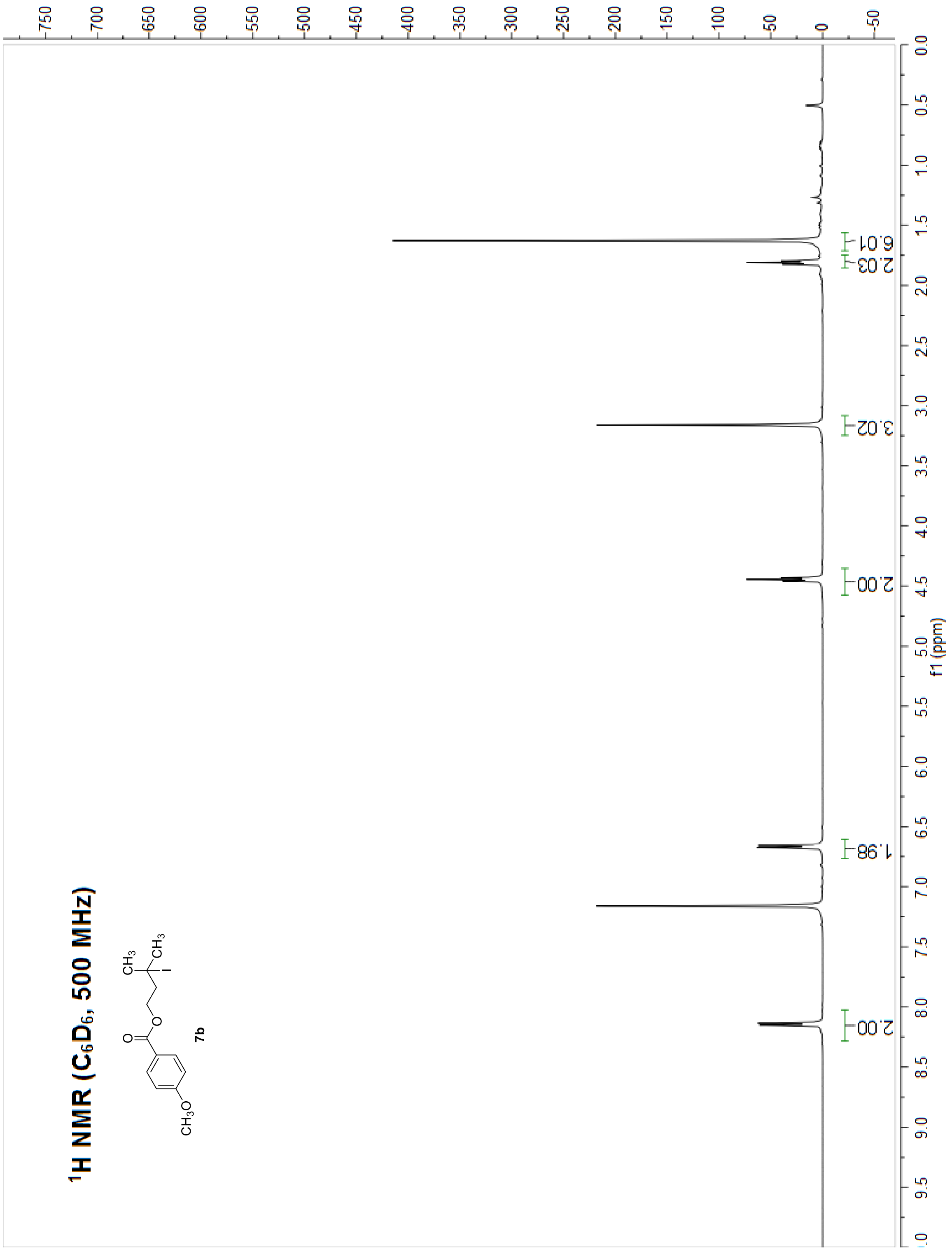
1H NMR (C6D6, 500 MHz)
7b
f1 (ppm)
6.01
2.03
3.02
2.00
1.98
2.00

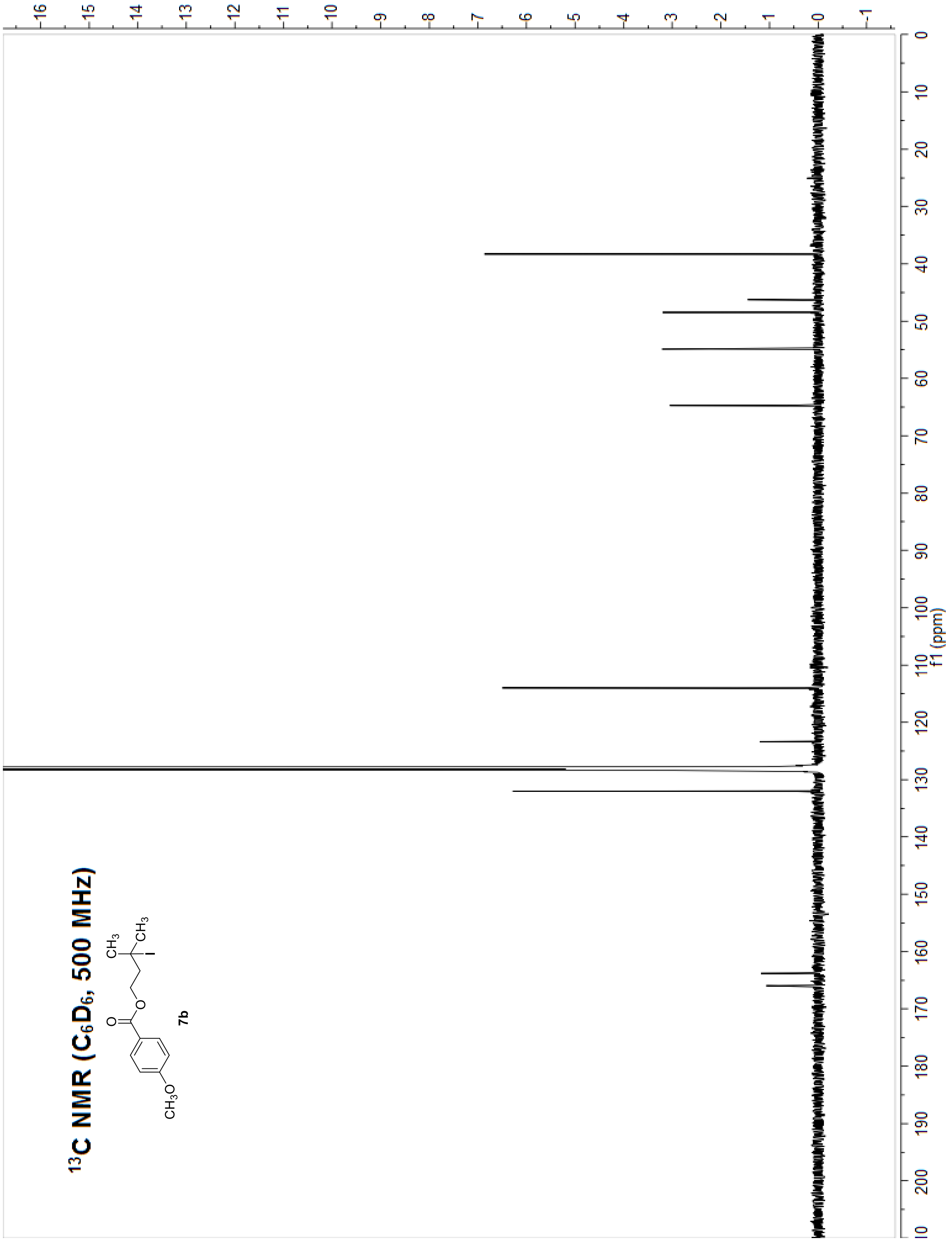

^{13}C NMR (C_6D_6, 500 MHz)
7b
f1 (ppm)

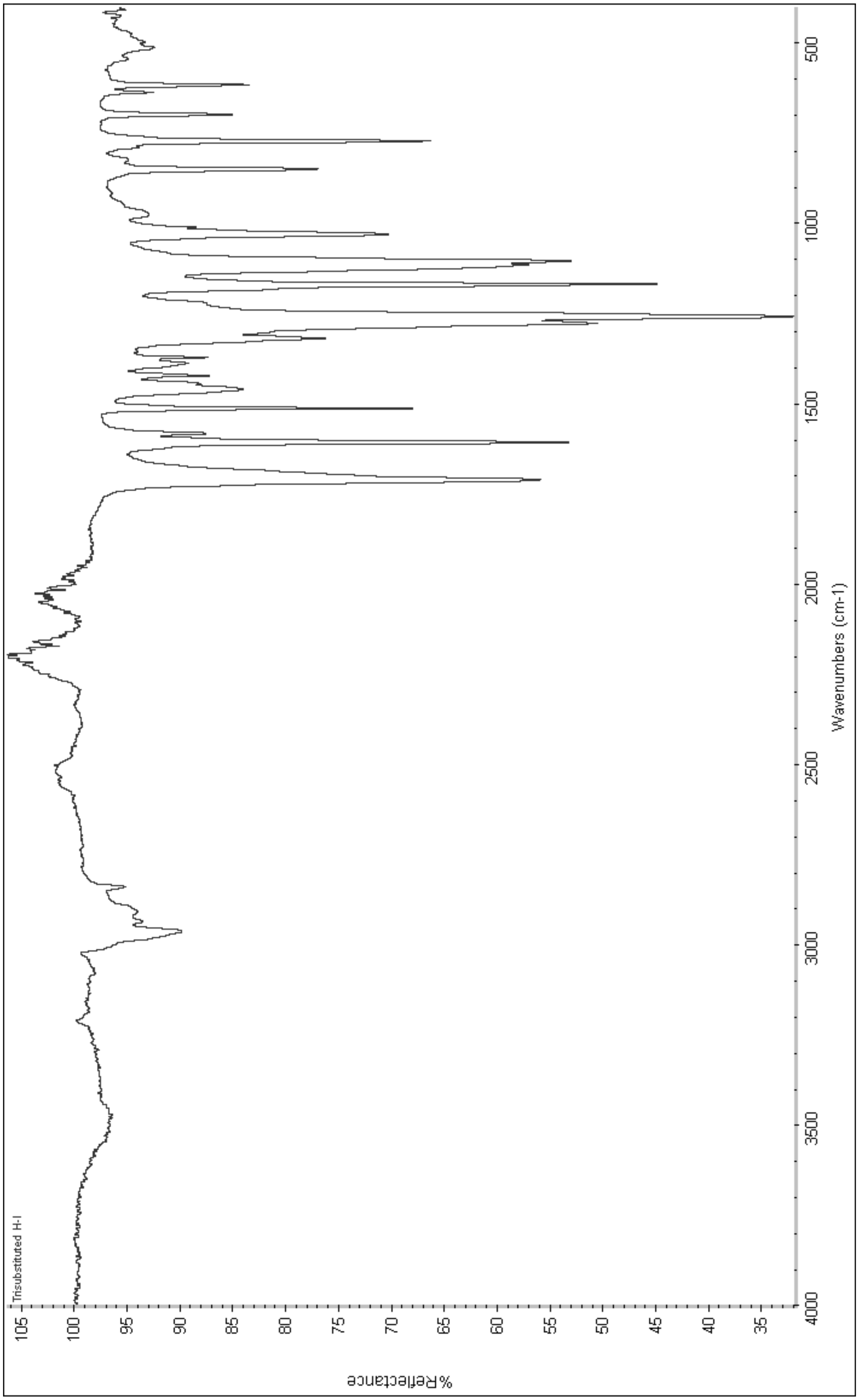
Trisubstituted H-I
%Reflectance
Wavenumbers (cm-1)

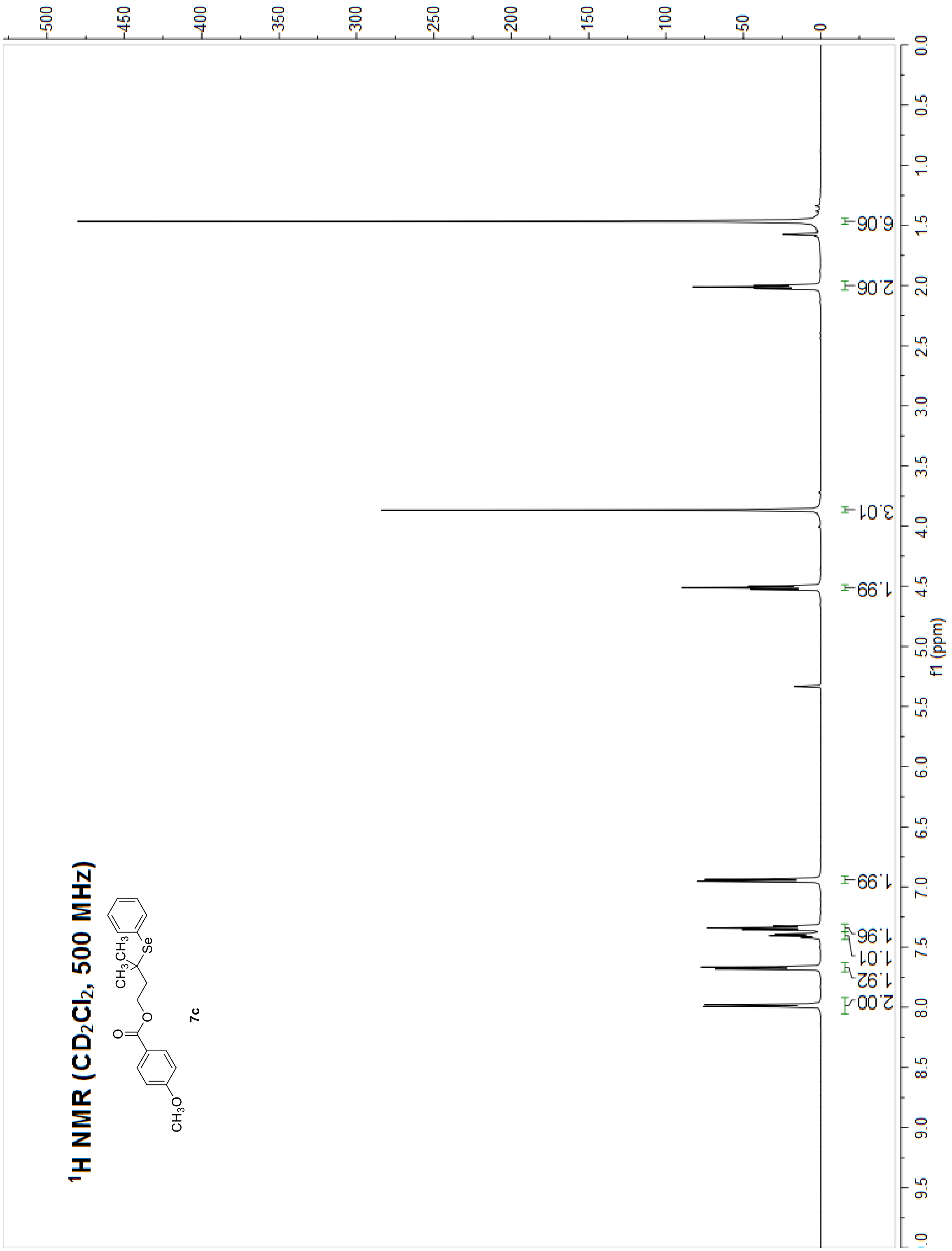

1H NMR (CD2Cl2, 500 MHz)
7c
f1 (ppm)

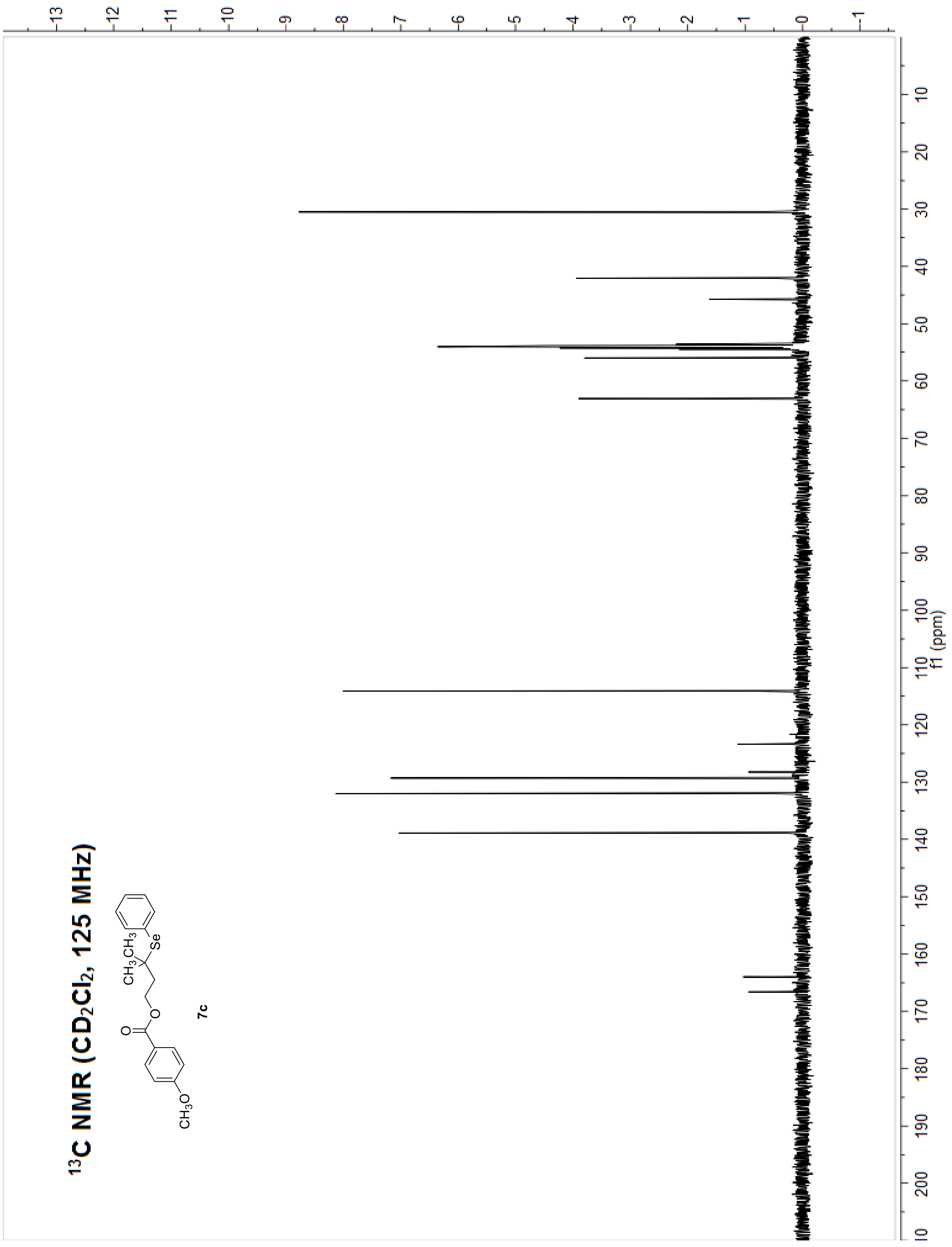

13C NMR (CD2Cl2, 125 MHz)
7c
f1 (ppm)

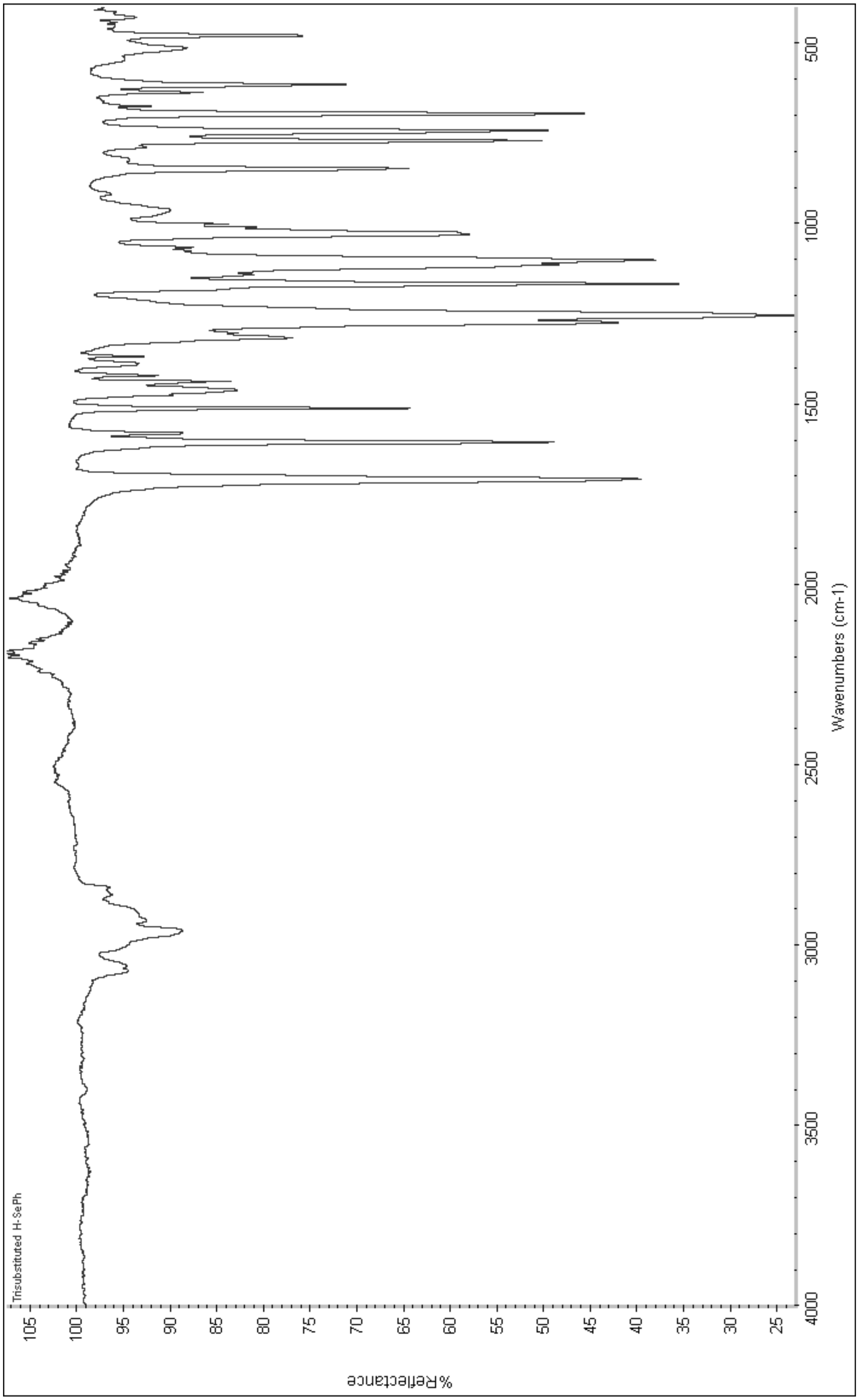
Trisubstituted H-SePh
%Reflectance
Wavenumbers (cm-1)

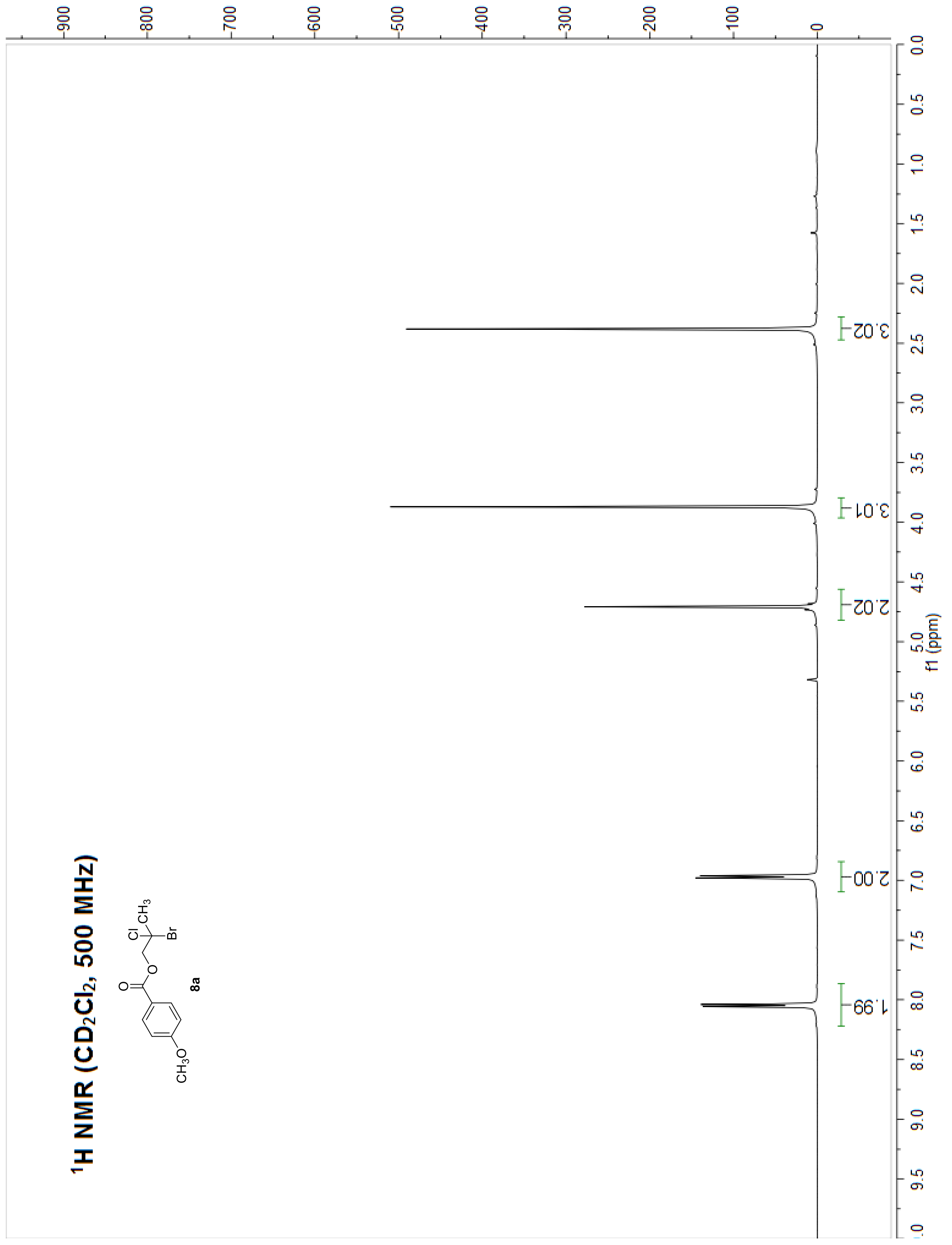

^{1}H NMR (CD_2Cl_2, 500 MHz)
8a
3.02
3.01
2.02
2.00
1.99
f1 (ppm)

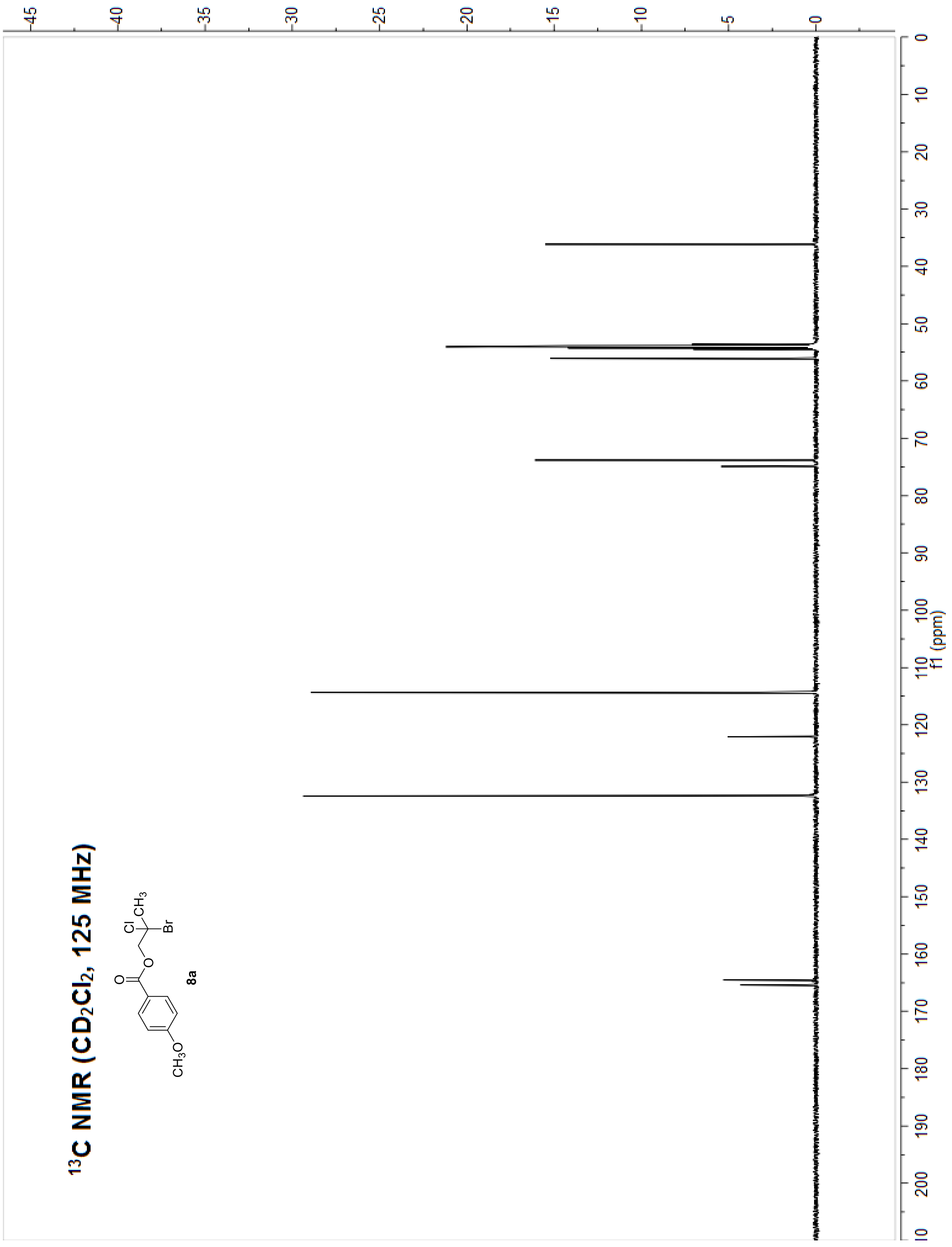
13C NMR (CD2Cl2, 125 MHz)
8a
f1 (ppm)

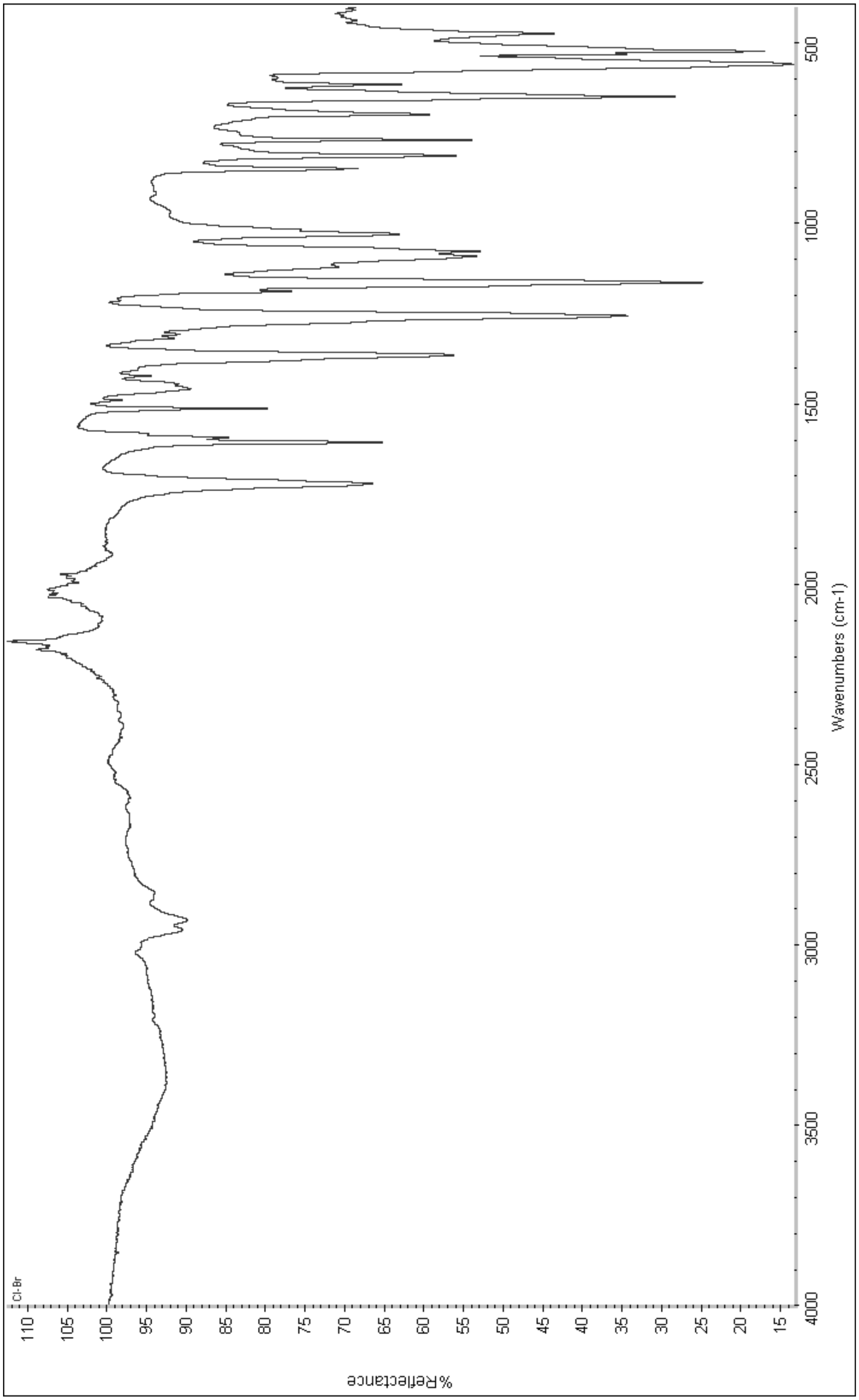
%Reflectance
Wavenumbers (cm-1)
Cl-Br

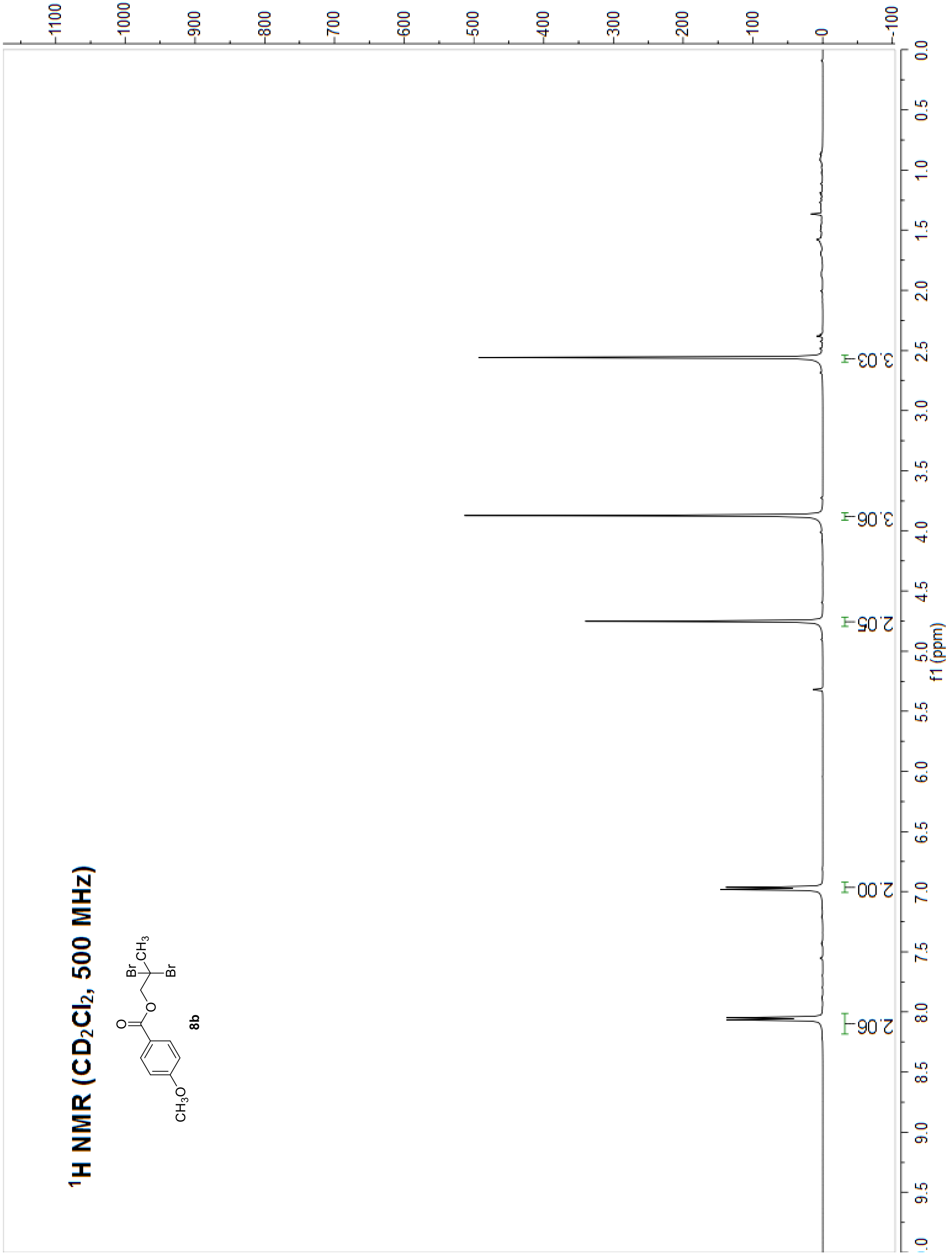

1H NMR (CD2Cl2, 500 MHz)
CH3O
O
O
Br
CH3
Br
8b
3.03
3.06
2.05
2.00
2.06
f1 (ppm)

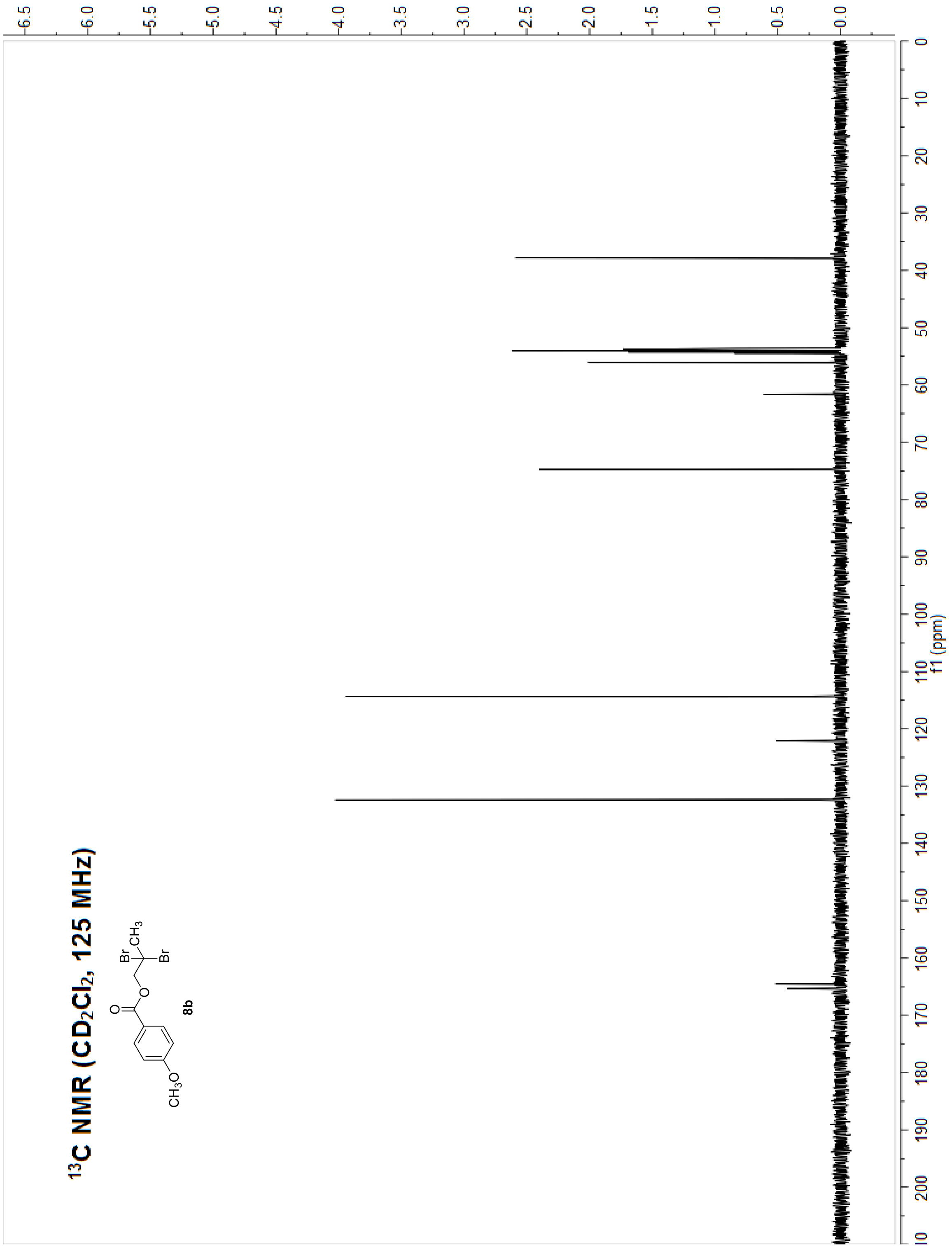
13C NMR (CD2Cl2, 125 MHz)
8b
f1 (ppm)

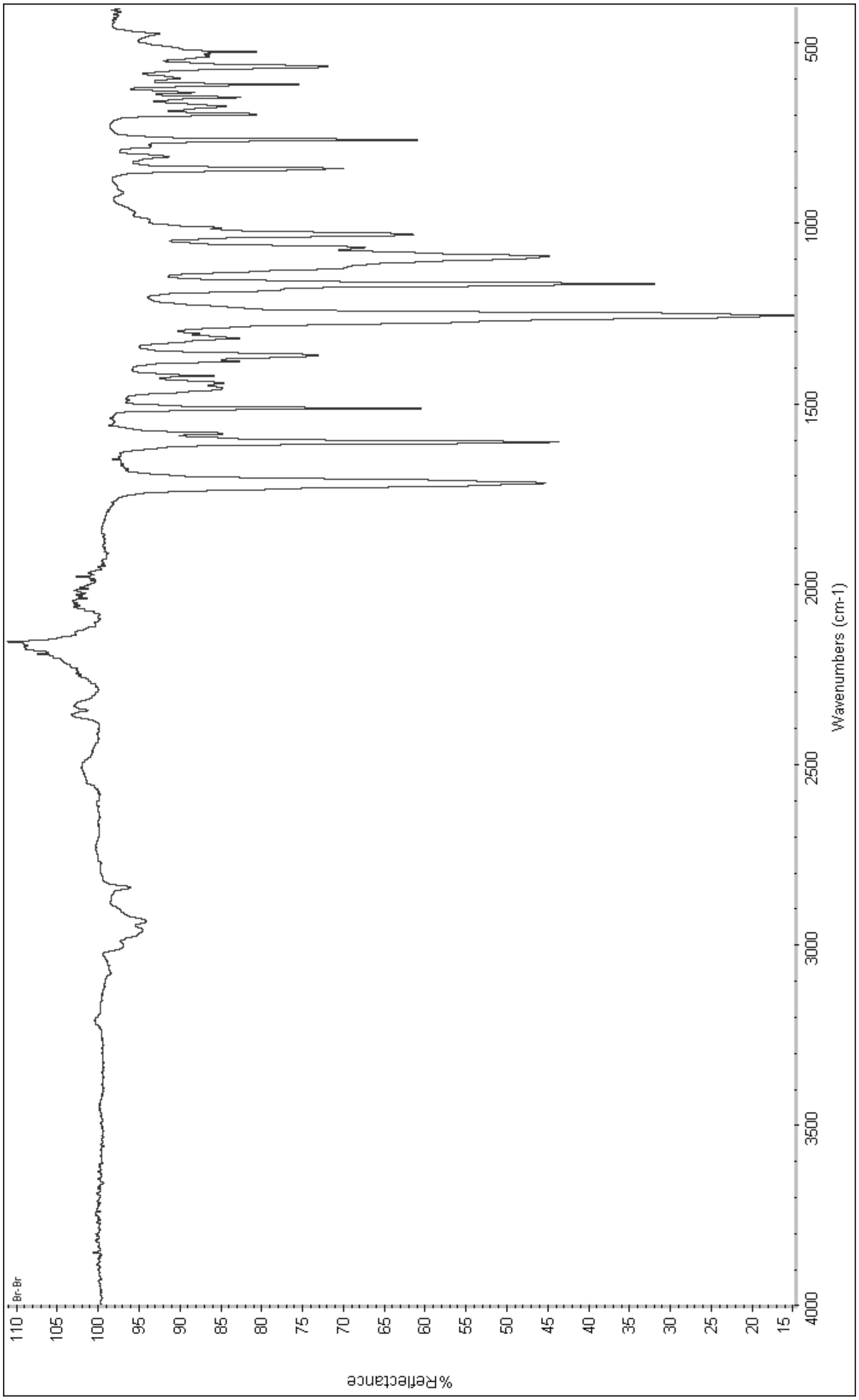
Br-Br
%Reflectance
Wavenumbers (cm-1)
110
105
100
95
90
85
80
75
70
65
60
55
50
45
40
35
30
25
20
15
4000
3500
3000
2500
2000
1500
1000
500

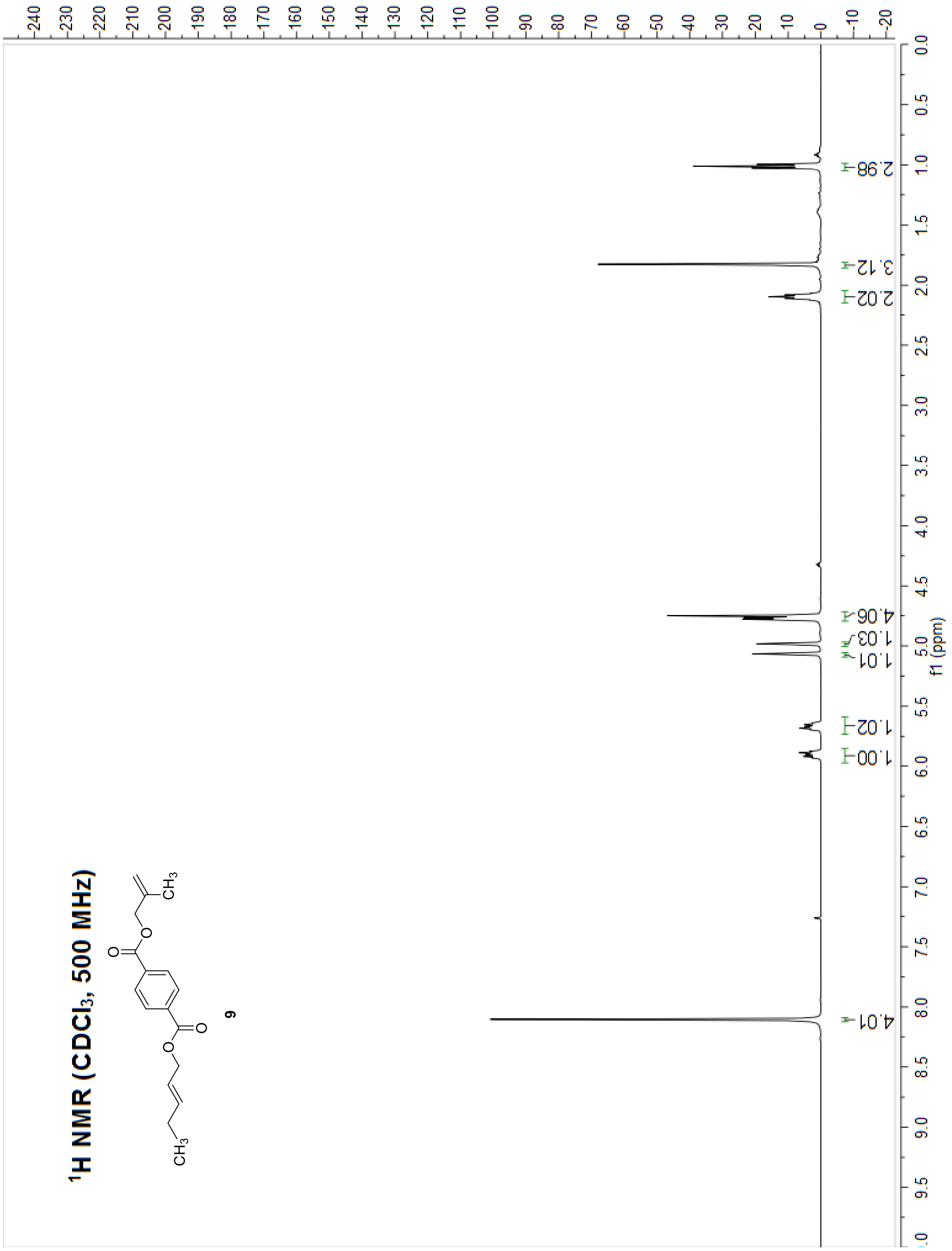
1H NMR (CDCl3, 500 MHz)
9
f1 (ppm)

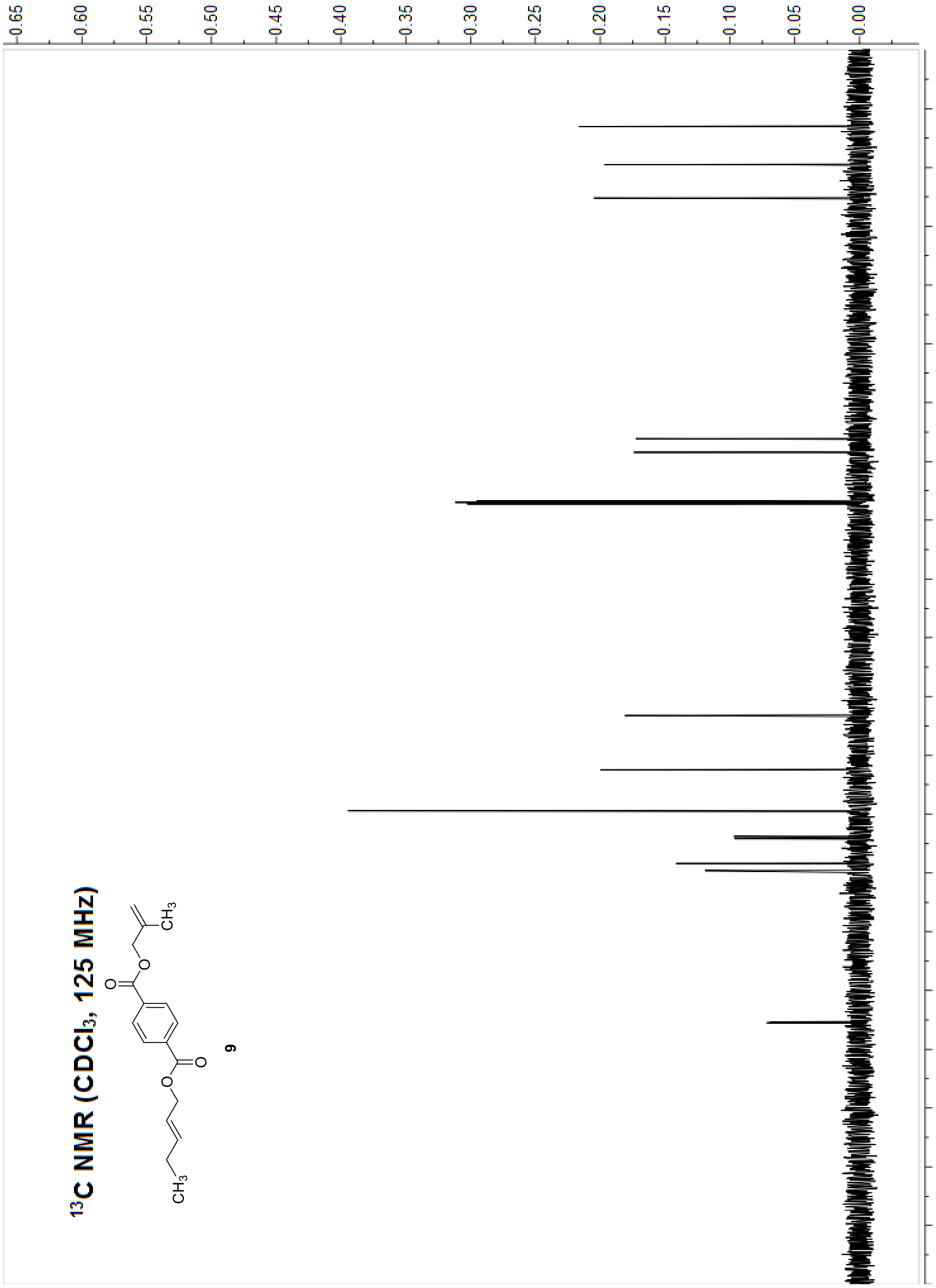

^{13}C NMR (CDCl$_3$, 125 MHz)
9
f1 (ppm)

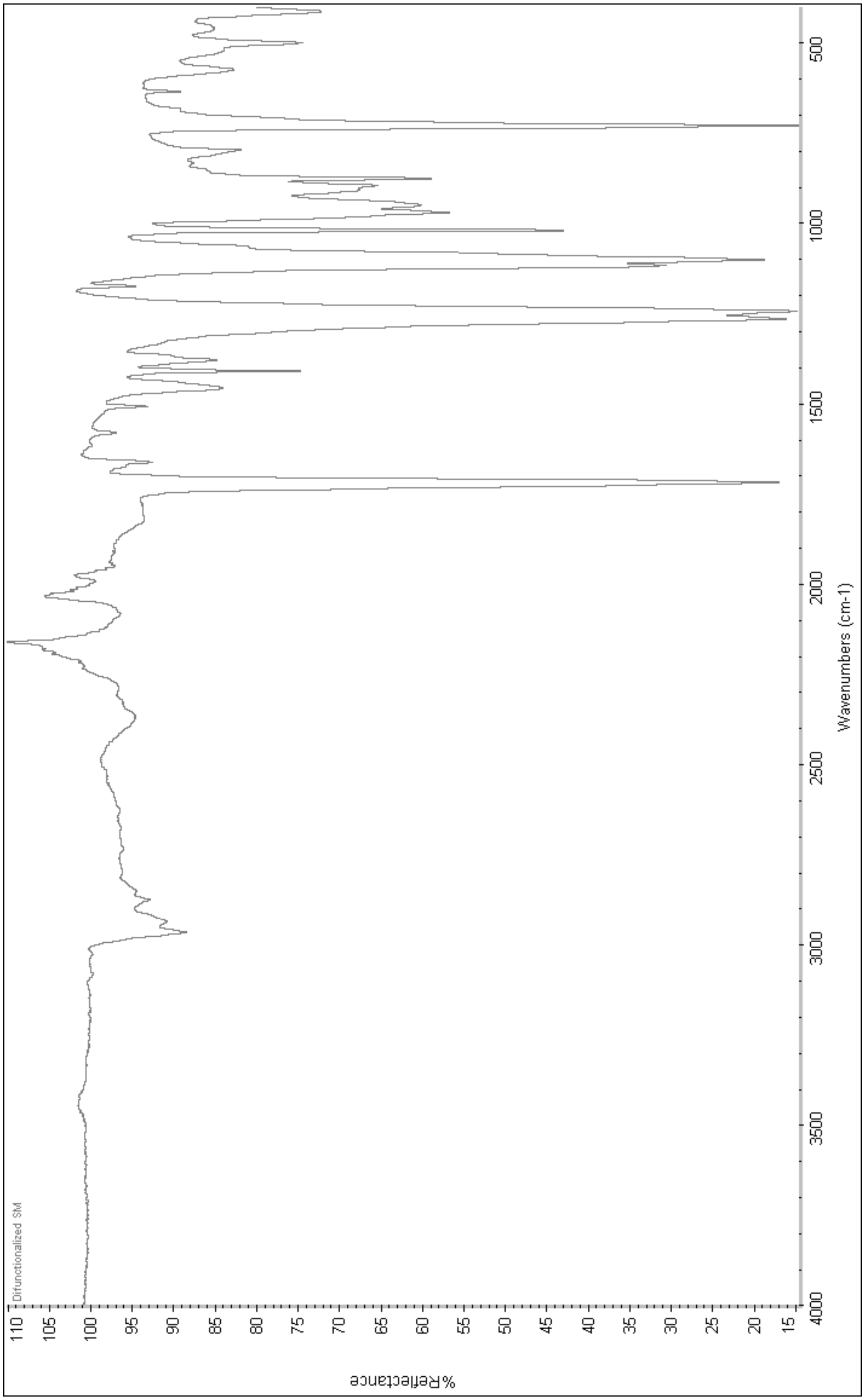
Difunctionalized SM
%Reflectance
Wavenumbers (cm-1)

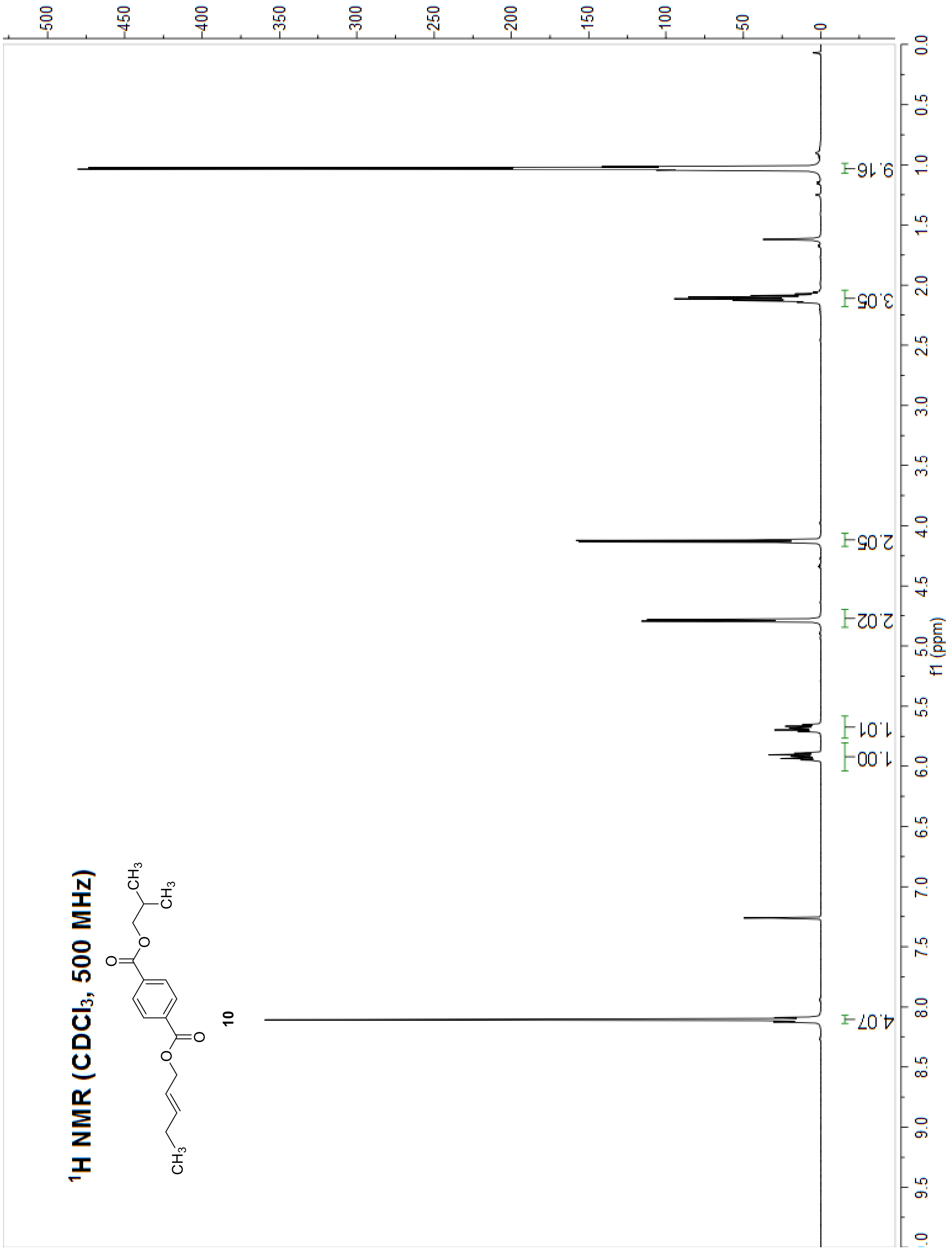
1H NMR (CDCl3, 500 MHz)
10
9.16
3.05
2.05
2.02
1.01
1.00
4.07
f1 (ppm)

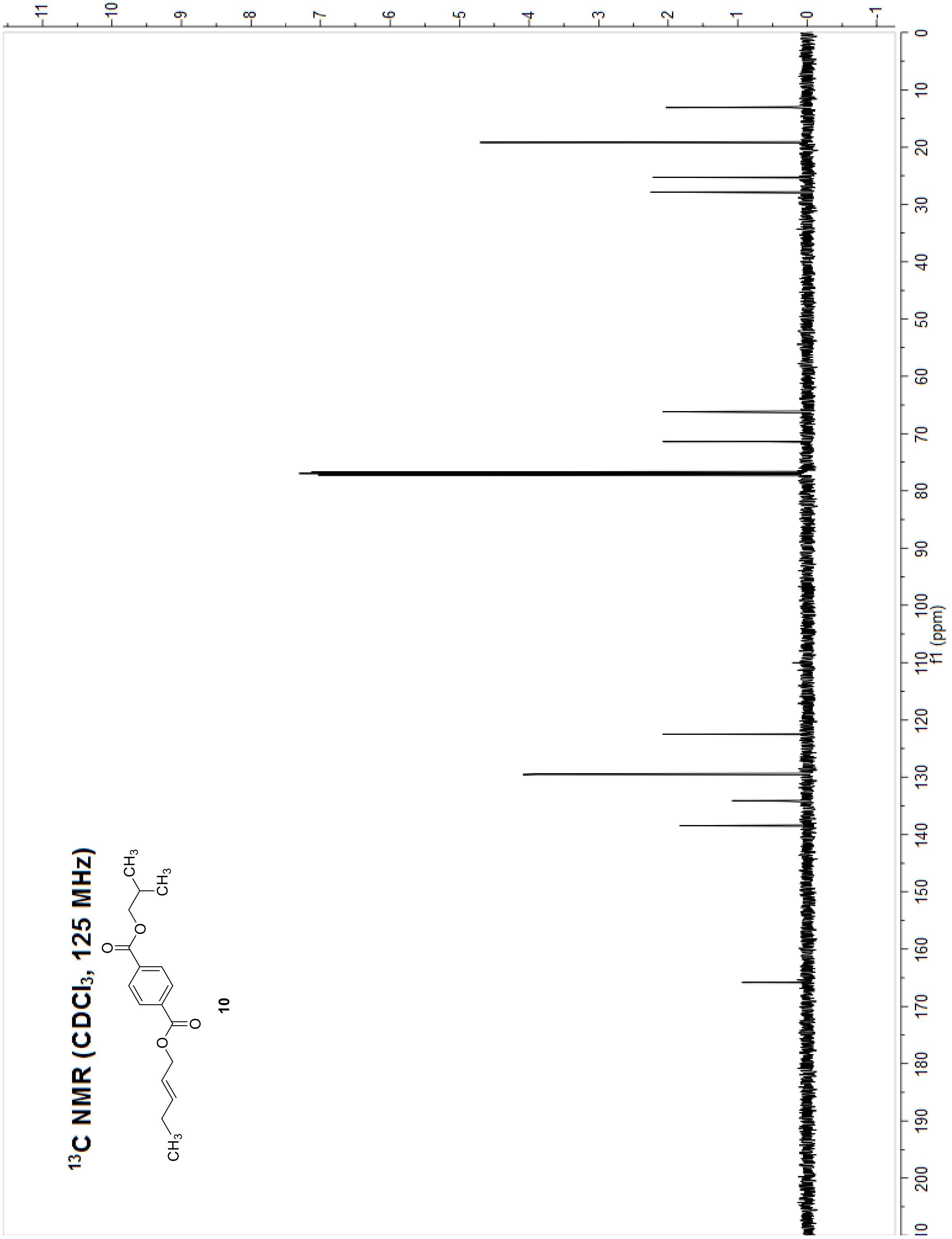

13C NMR (CDCl3, 125 MHz)
10
f1 (ppm)

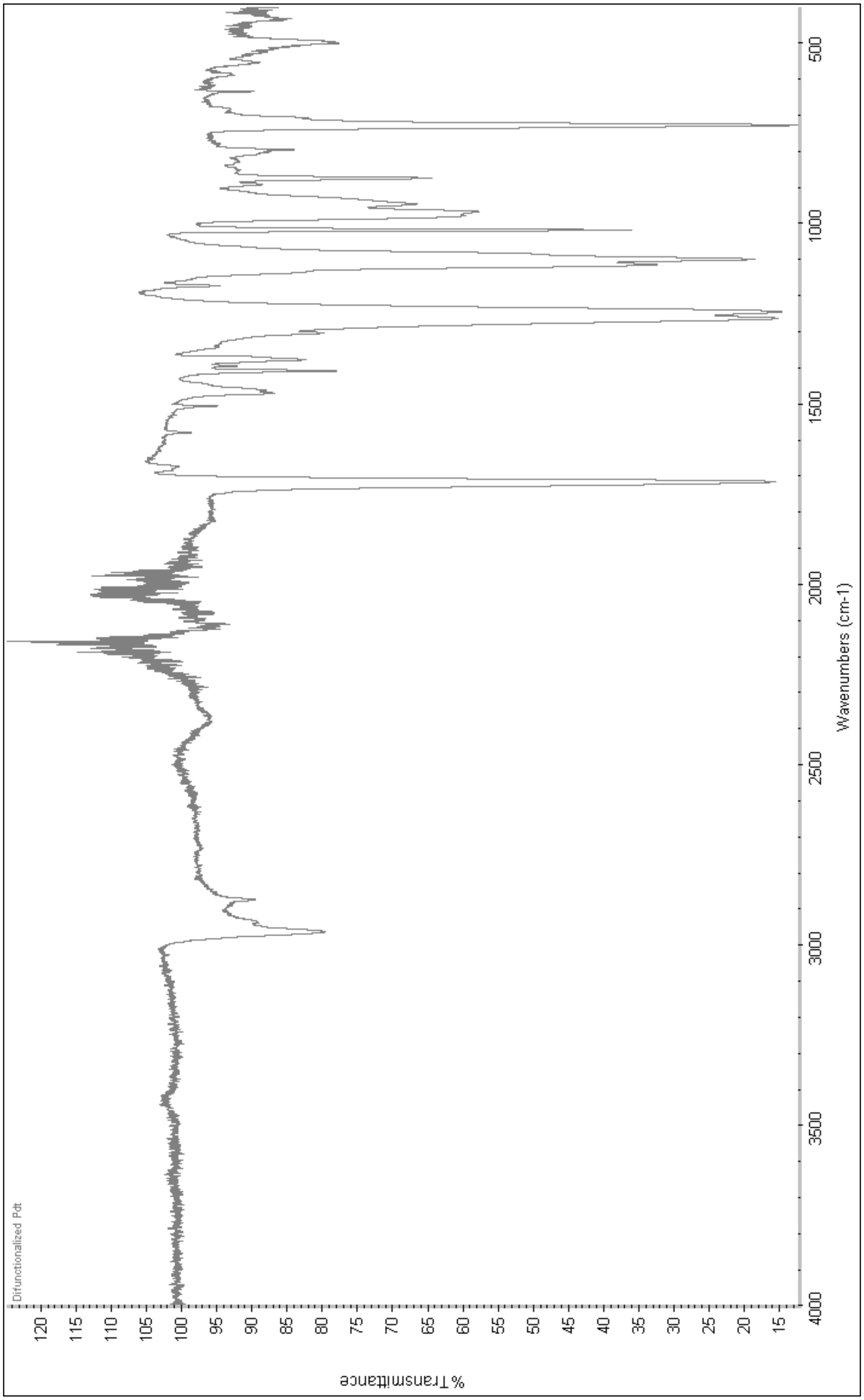
Difunctionalized Pdt
%Transmittance
Wavenumbers (cm-1)

^{1}H NMR Spectra of Unpurified Product Mixtures Containing Added Internal Standard

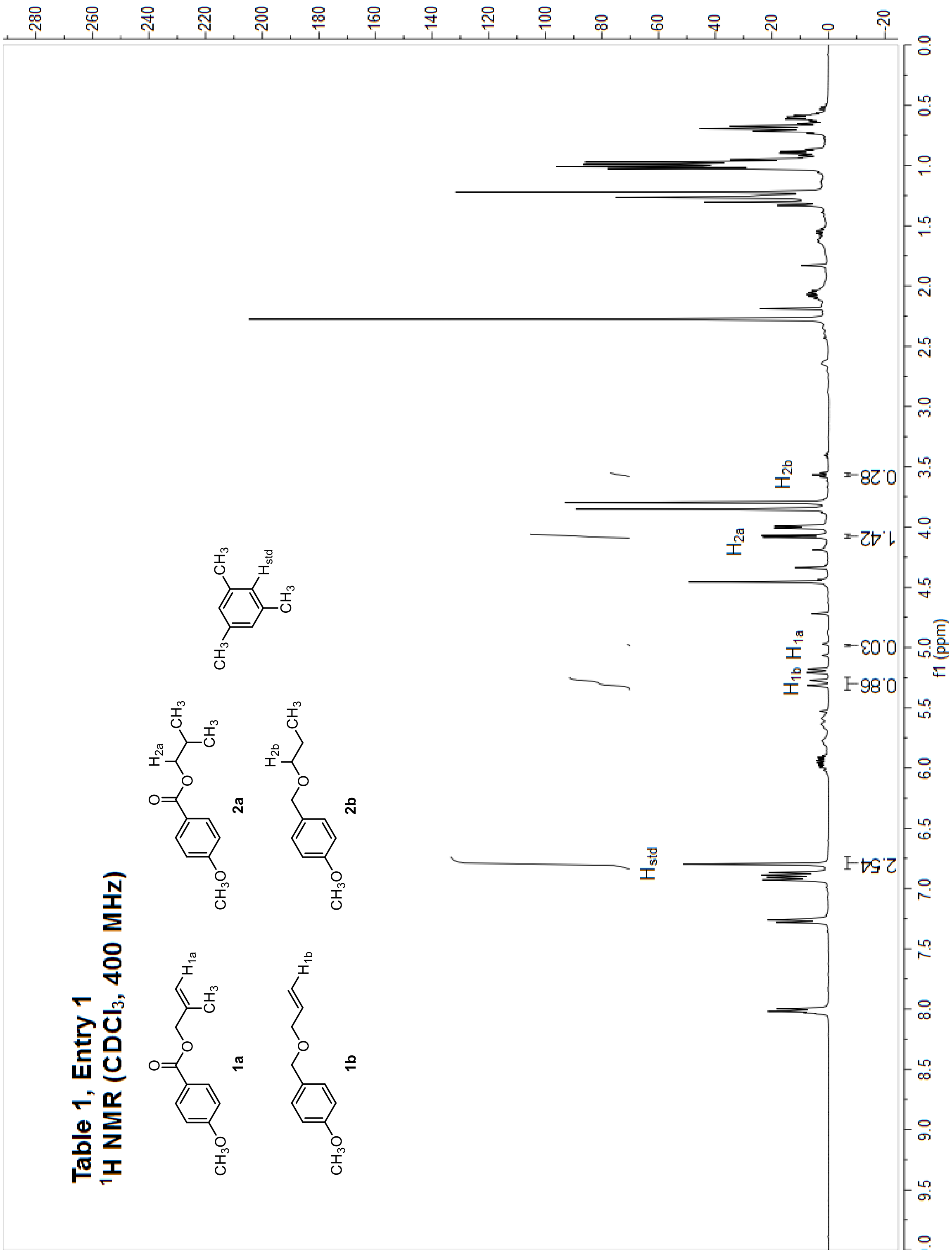

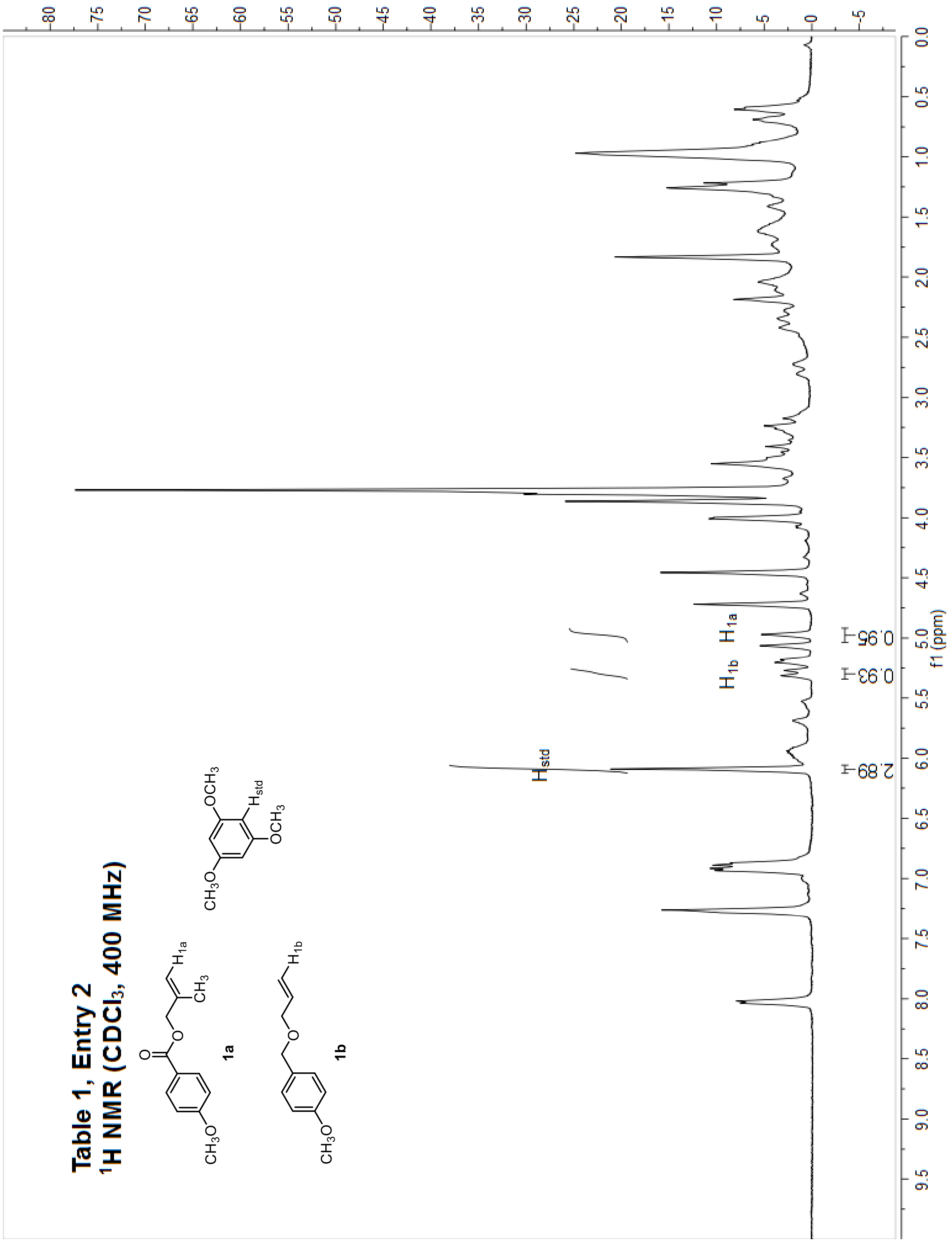
Table 1, Entry 2
1H NMR (CDCl3, 400 MHz)
1a
1b
H1a
H1b
Hstd
0.95
0.93
2.89
f1 (ppm)

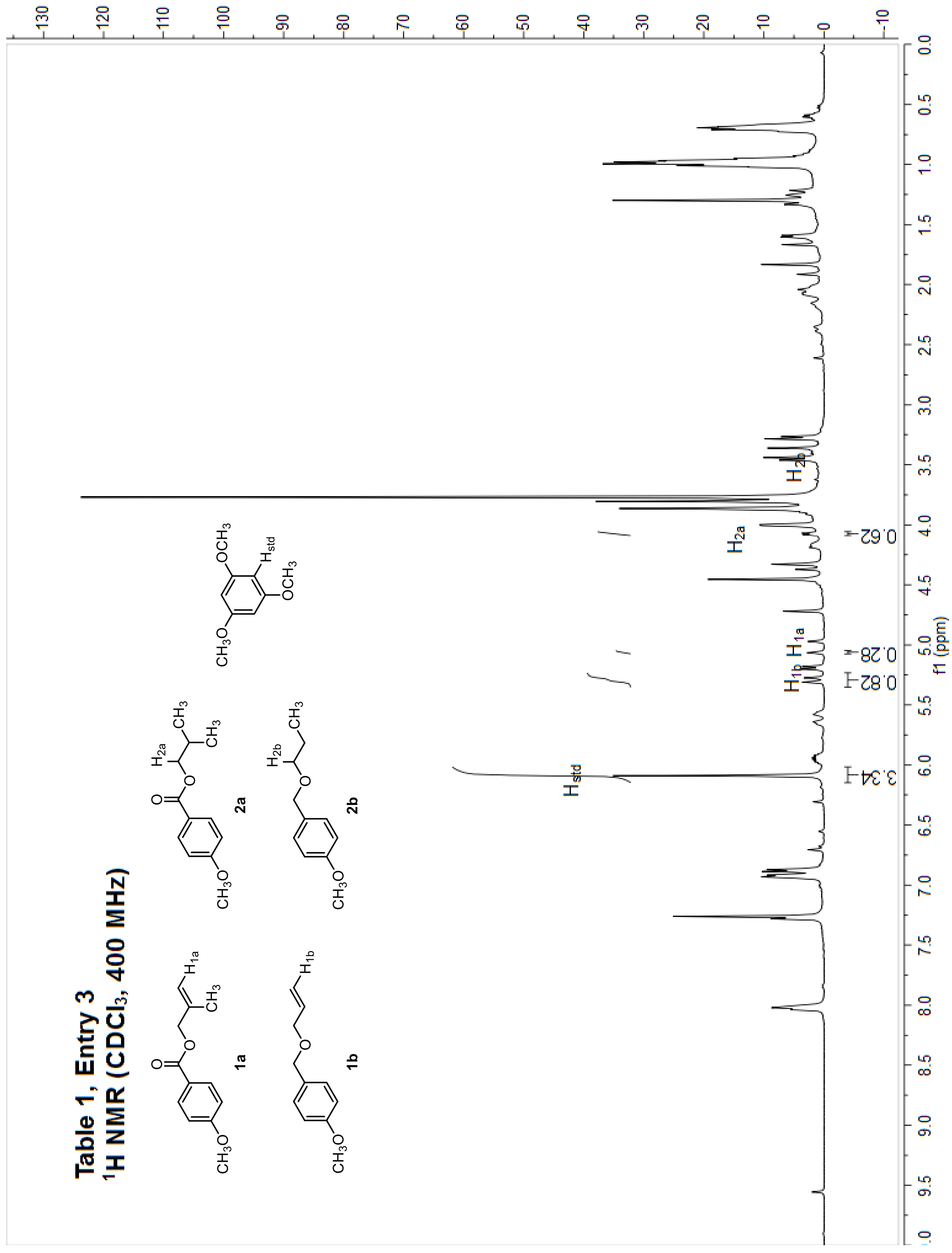
Table 1, Entry 3
1H NMR (CDCl3, 400 MHz)
1a
1b
2a
2b
H1a
H1b
H2a
H2b
Hstd
0.62
0.28
0.82
3.34
f1 (ppm)

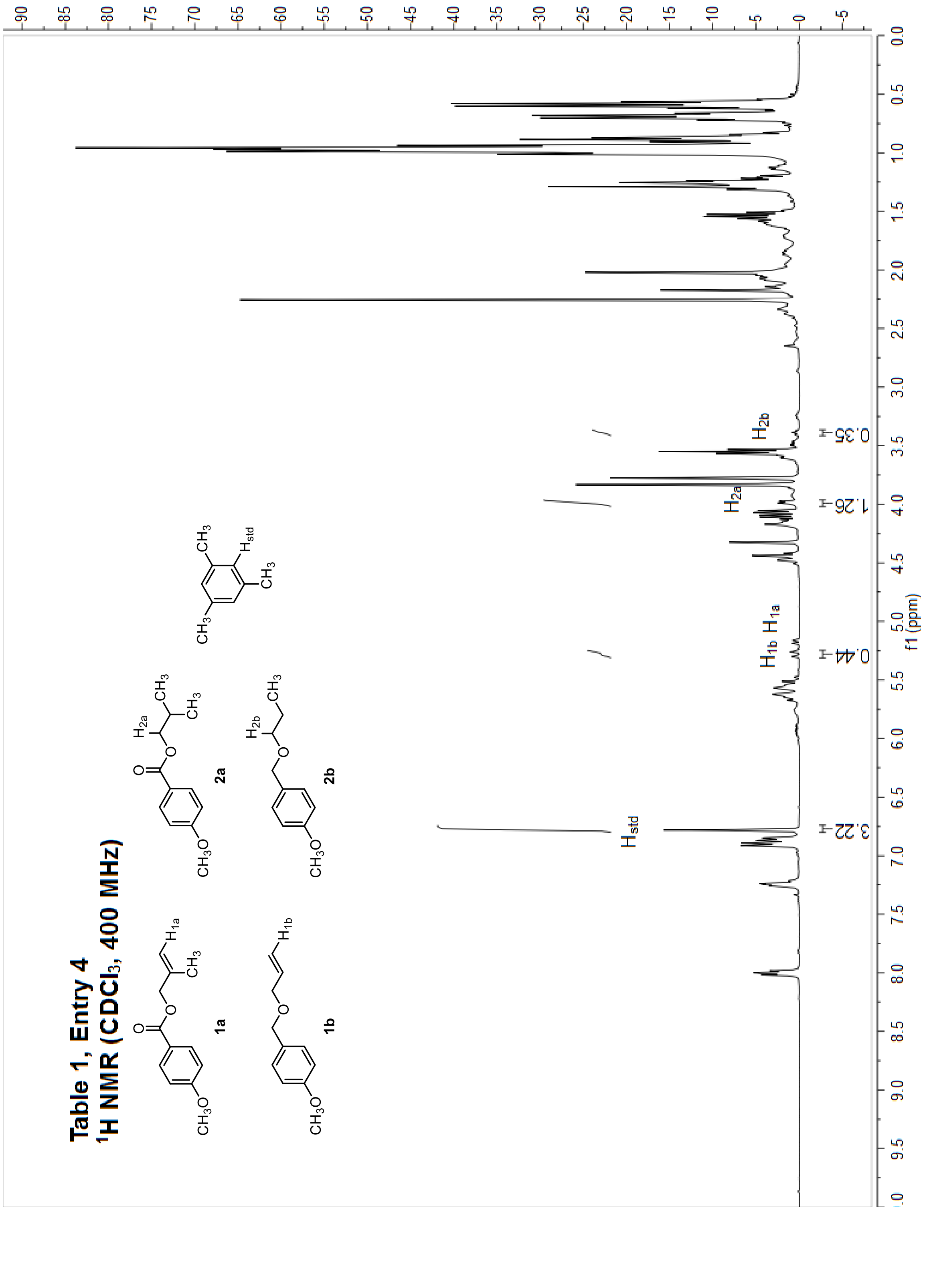
Table 1, Entry 4
1H NMR (CDCl3, 400 MHz)
1a
1b
2a
2b
Hstd
H2a
H2b
H1b H1a
3.22
0.44
1.26
0.35
f1 (ppm)

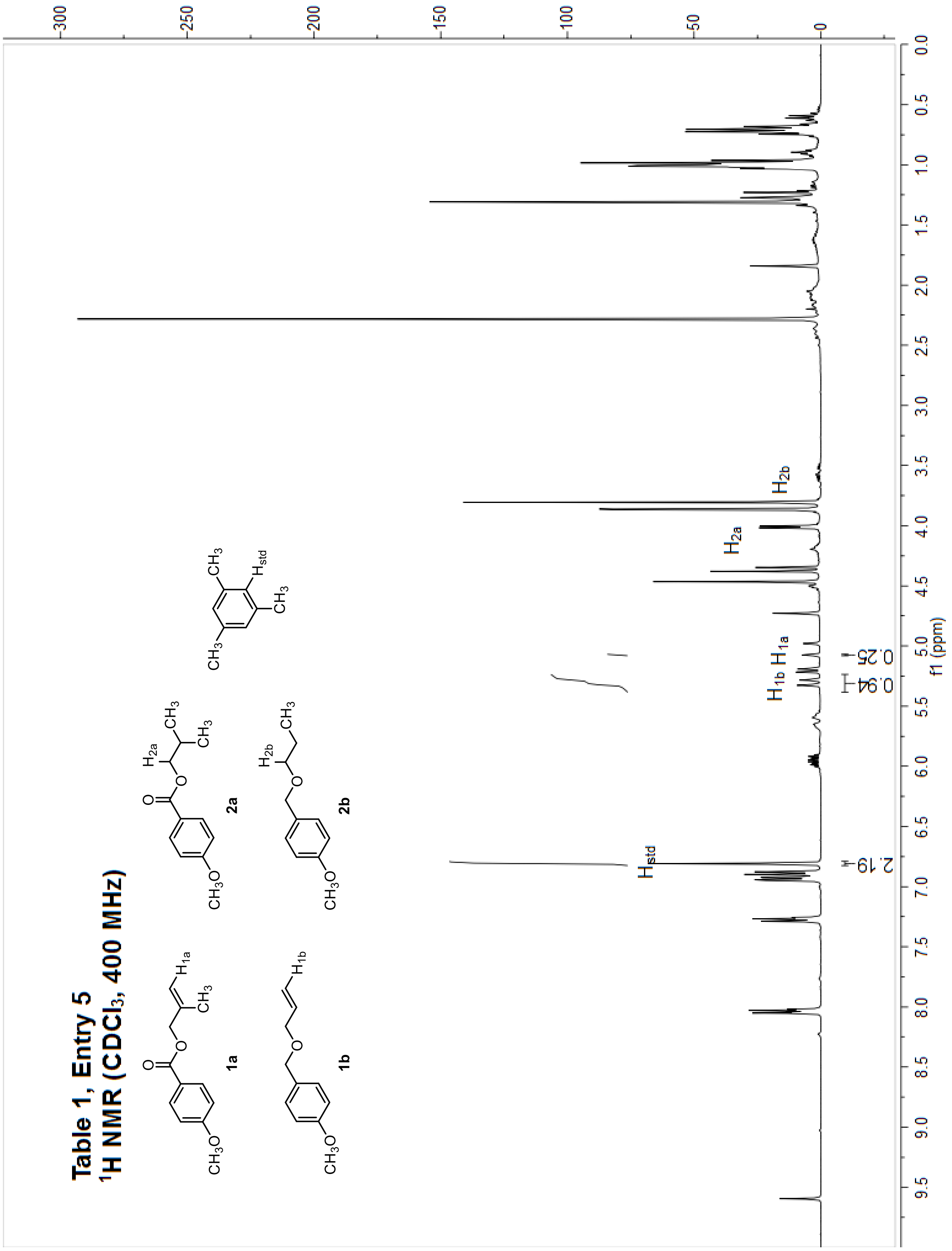
Table 1, Entry 5
1H NMR (CDCl3, 400 MHz)
1a
1b
2a
2b
Hstd
H2a
H2b
H1b H1a
0.25
0.94
2.19
f1 (ppm)

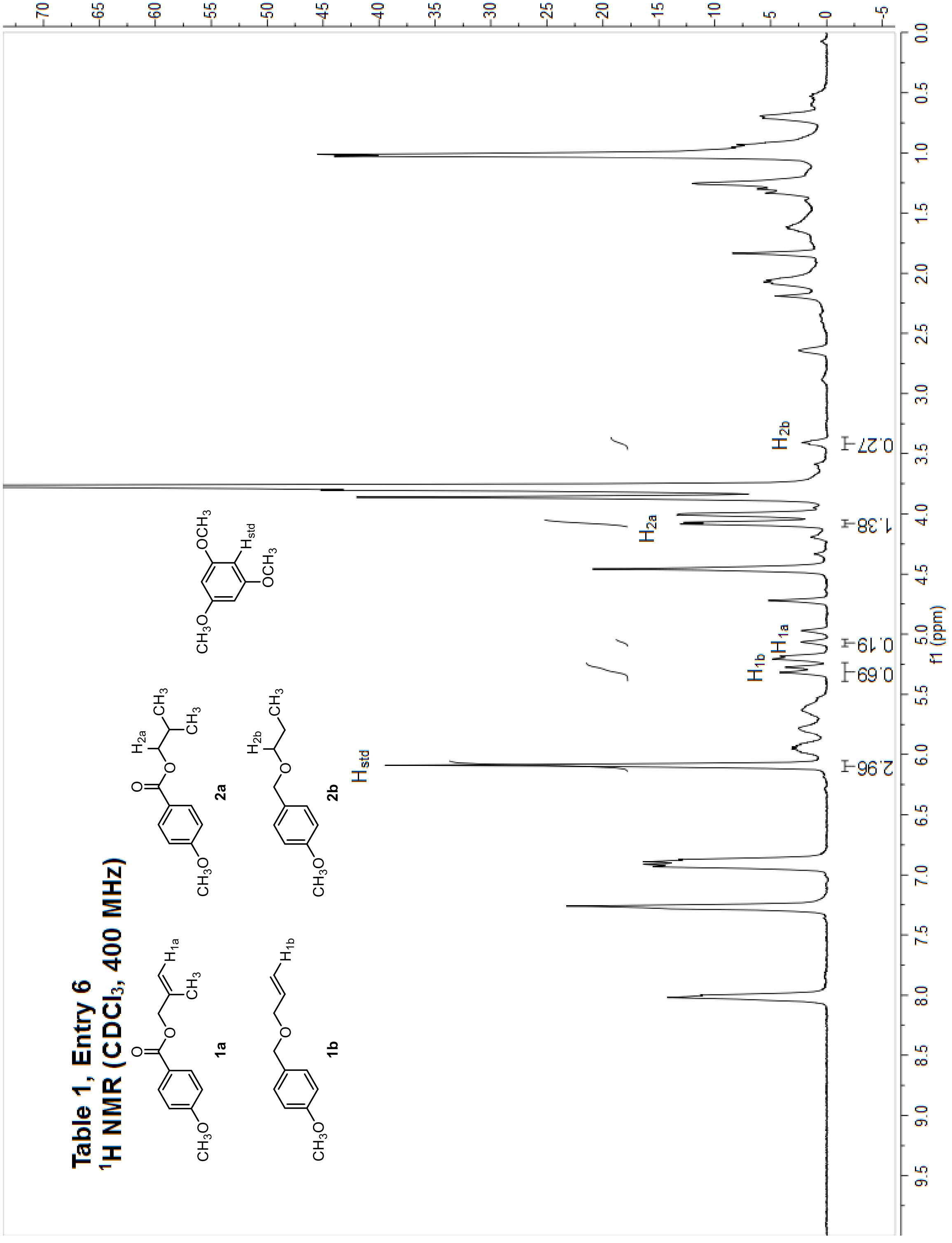

Table 1, Entry 6
1H NMR (CDCl3, 400 MHz)
1a
1b
2a
2b
H1a
H1b
H2a
H2b
Hstd
0.27
1.38
0.19
0.69
2.96
f1 (ppm)

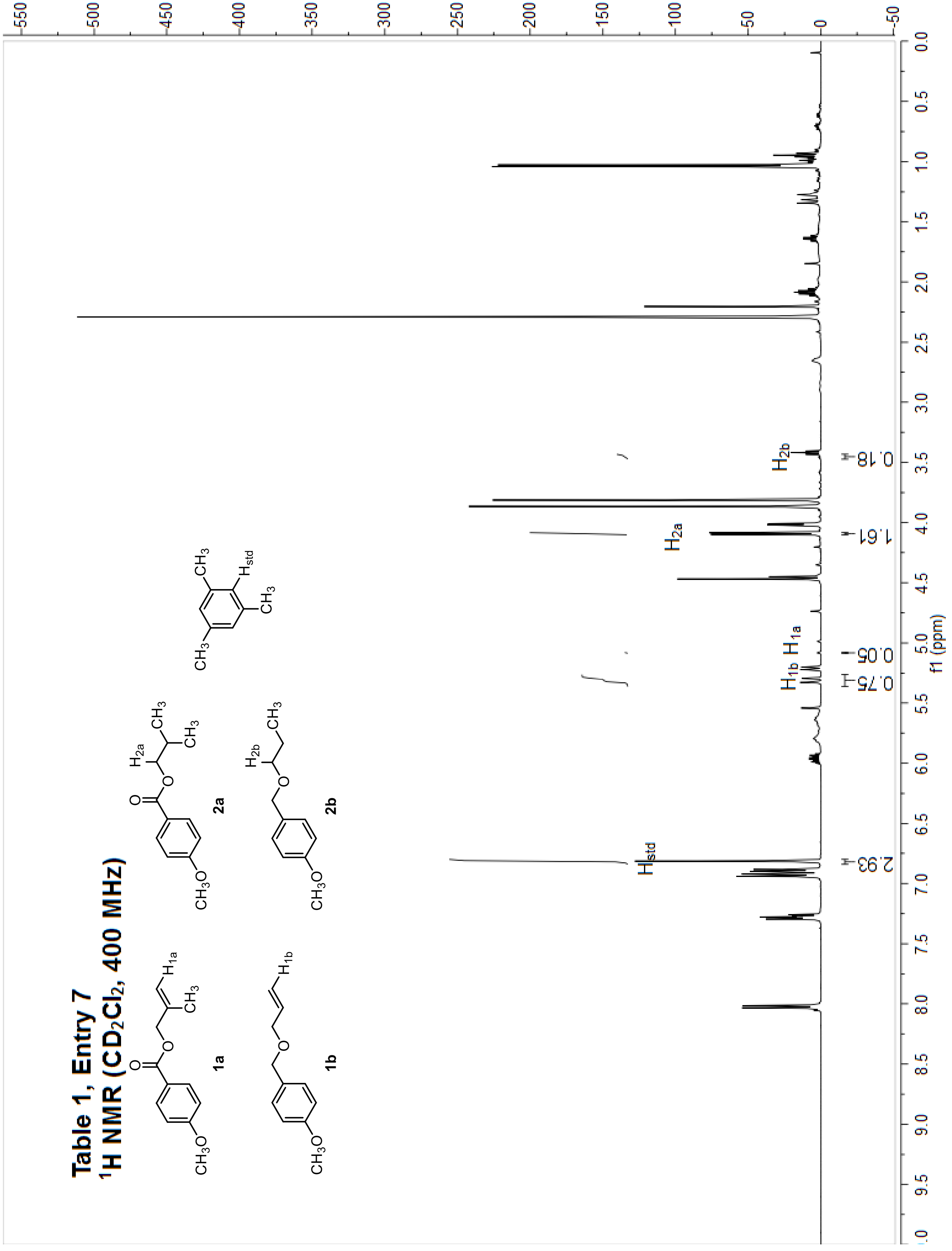
Table 1, Entry 7
1H NMR (CD2Cl2, 400 MHz)
1a
1b
2a
2b
H2b
H2a
H1b H1a
Hstd
0.18
1.61
0.05
0.75
2.93
f1 (ppm)

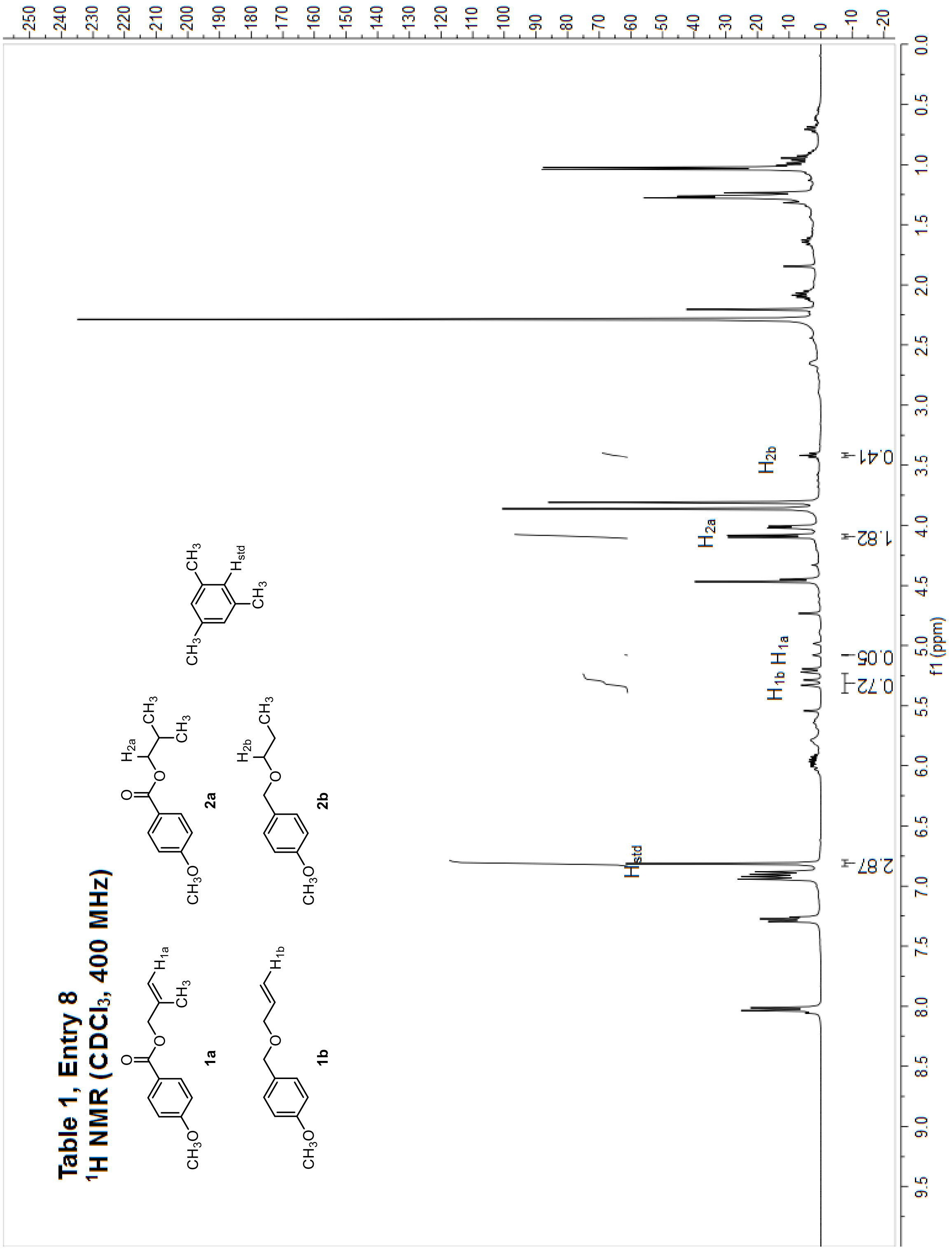
Table 1, Entry 8
1H NMR (CDCl3, 400 MHz)
1a
1b
2a
2b
H2b
H2a
H1b H1a
Hstd
0.41
1.82
0.05
0.72
2.87
f1 (ppm)

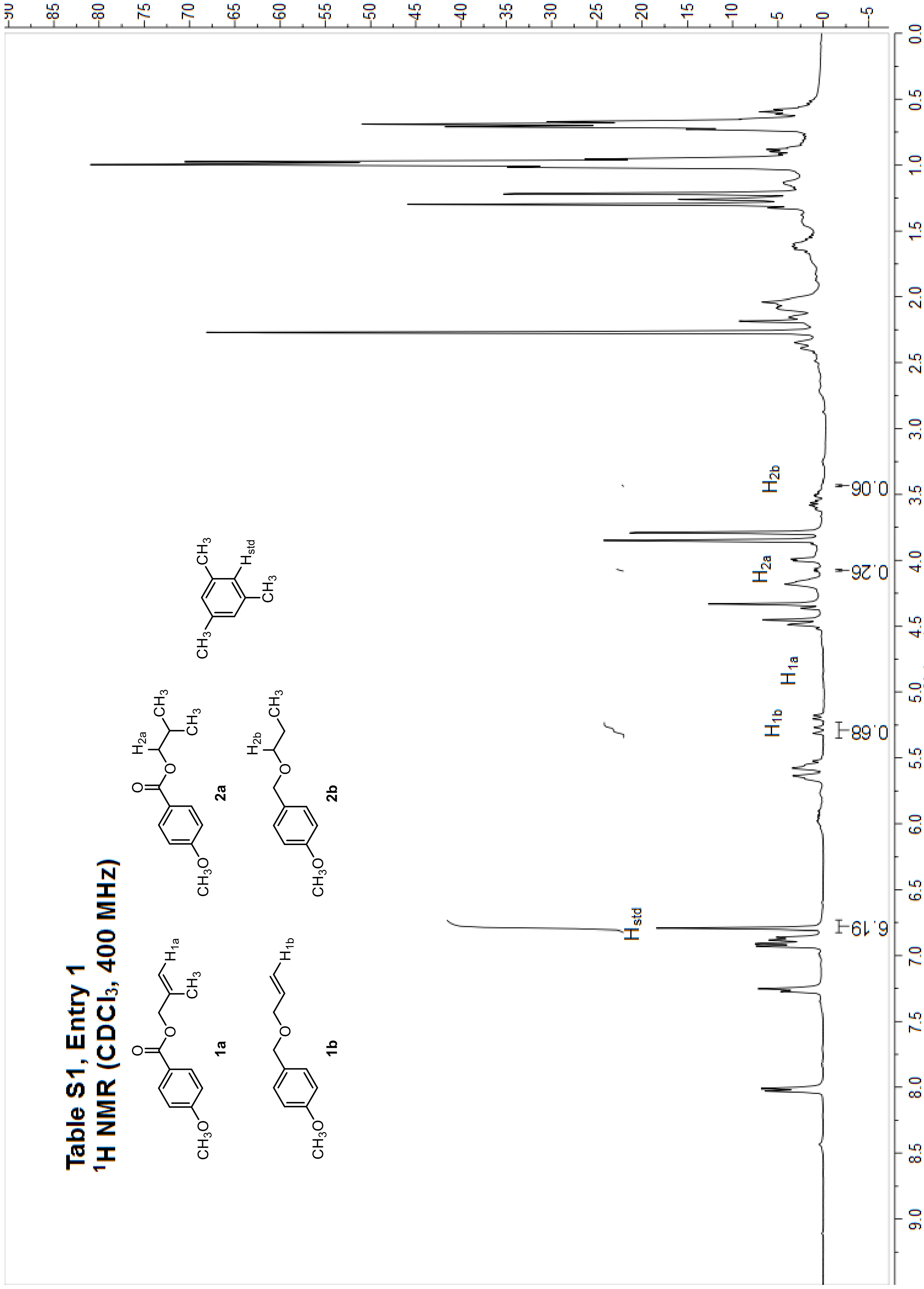
Table S1, Entry 1
1H NMR (CDCl3, 400 MHz)
1a
1b
2a
2b
H1a
H1b
H2a
H2b
Hstd
0.06
0.26
0.68
6.19
f1 (ppm)

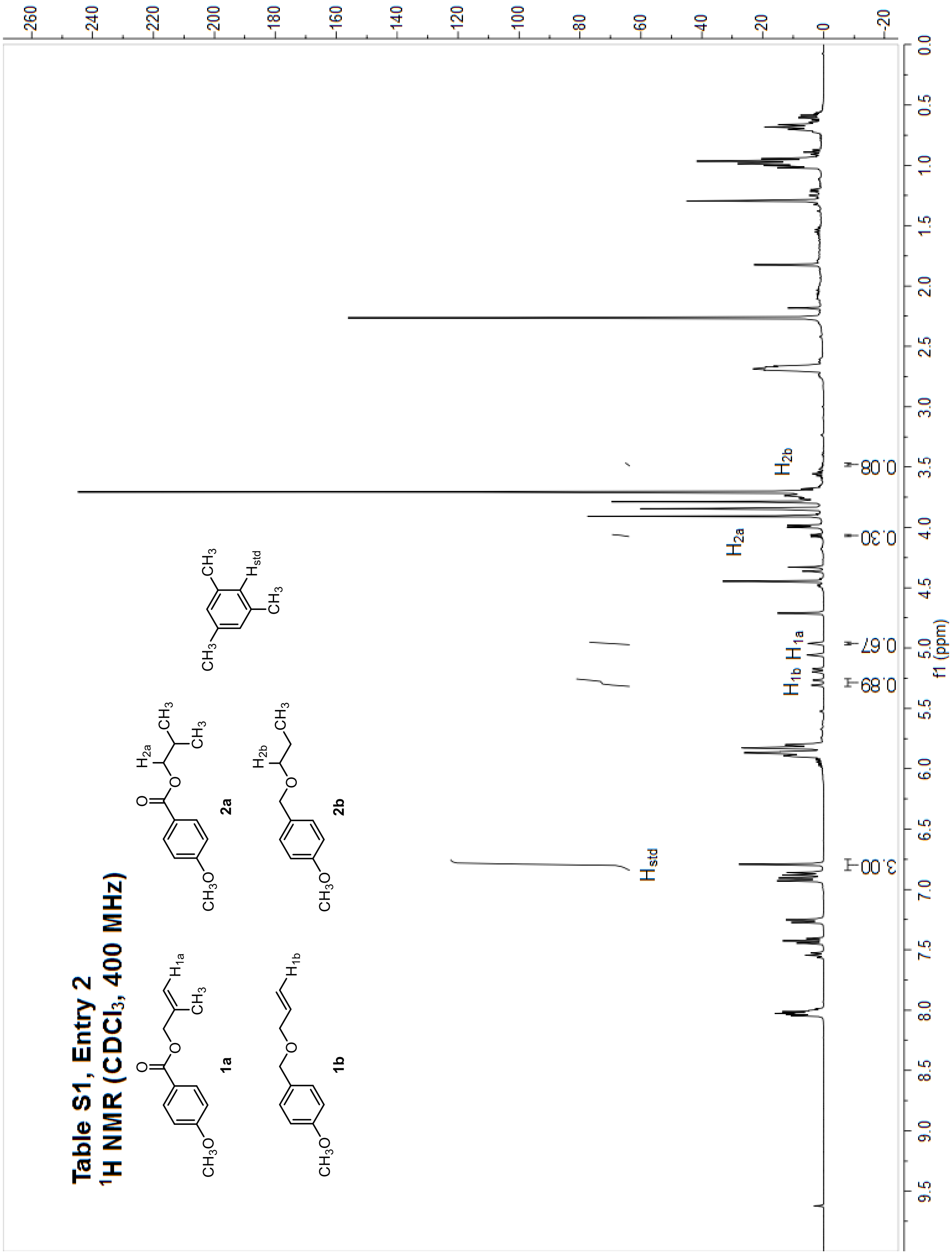
Table S1, Entry 2
^1H NMR ($CDCl_3$, 400 MHz)
1a
1b
2a
2b
H_{1a}
H_{1b}
H_{2a}
H_{2b}
H_{std}
CH_3O
CH_3
0.08
0.30
0.67
0.89
3.00
f1 (ppm)

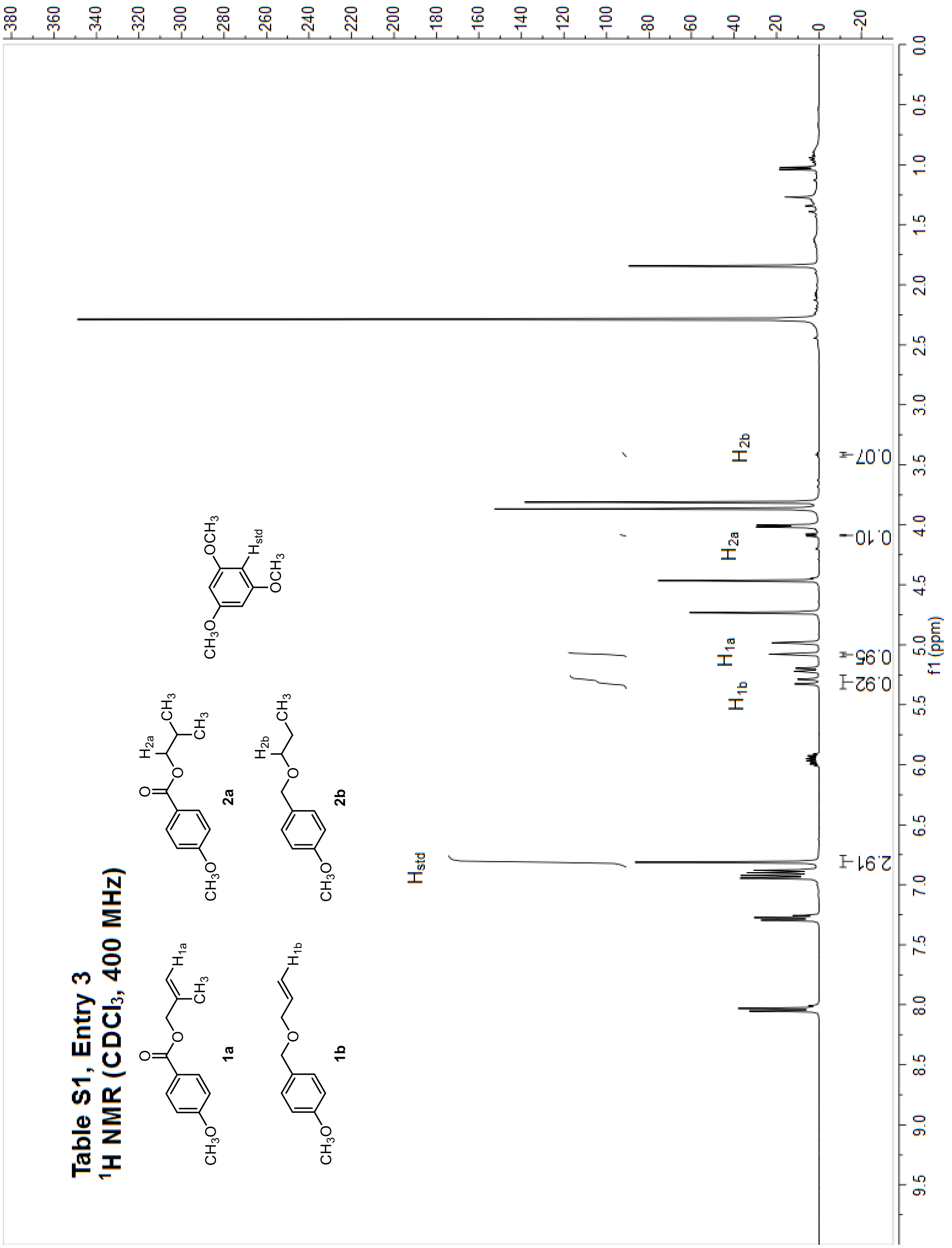
Table S1, Entry 3
1H NMR (CDCl3, 400 MHz)
1a
1b
2a
2b
Hstd
H2b
H2a
H1a
H1b
0.07
0.10
0.95
0.92
2.91
f1 (ppm)

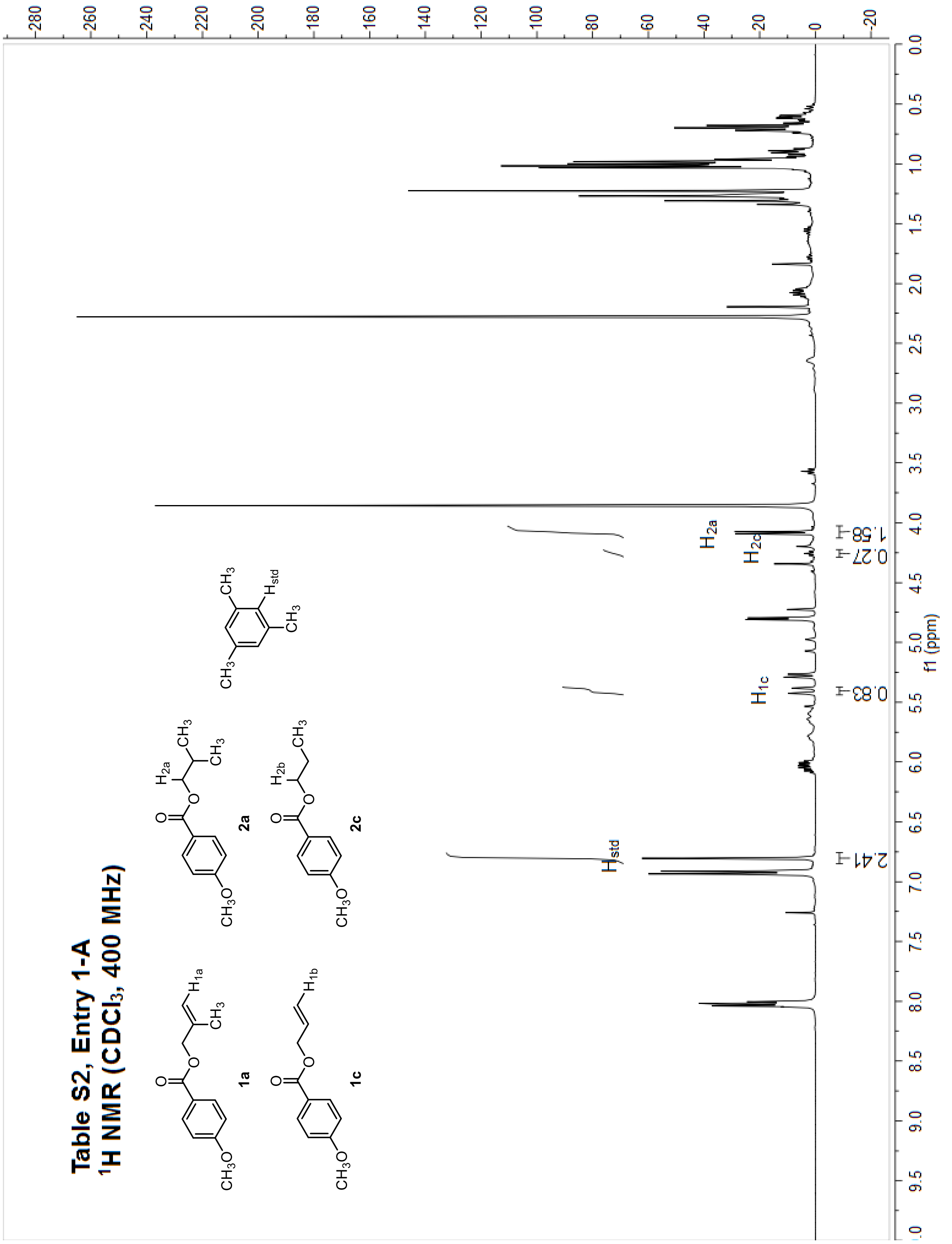
Table S2, Entry 1-A
1H NMR (CDCl3, 400 MHz)
1a
1c
2a
2c
H1a
H1b
H2a
H2b
H2c
H1c
Hstd
CH3
CH3O
O
1.58
0.27
0.83
2.41
f1 (ppm)

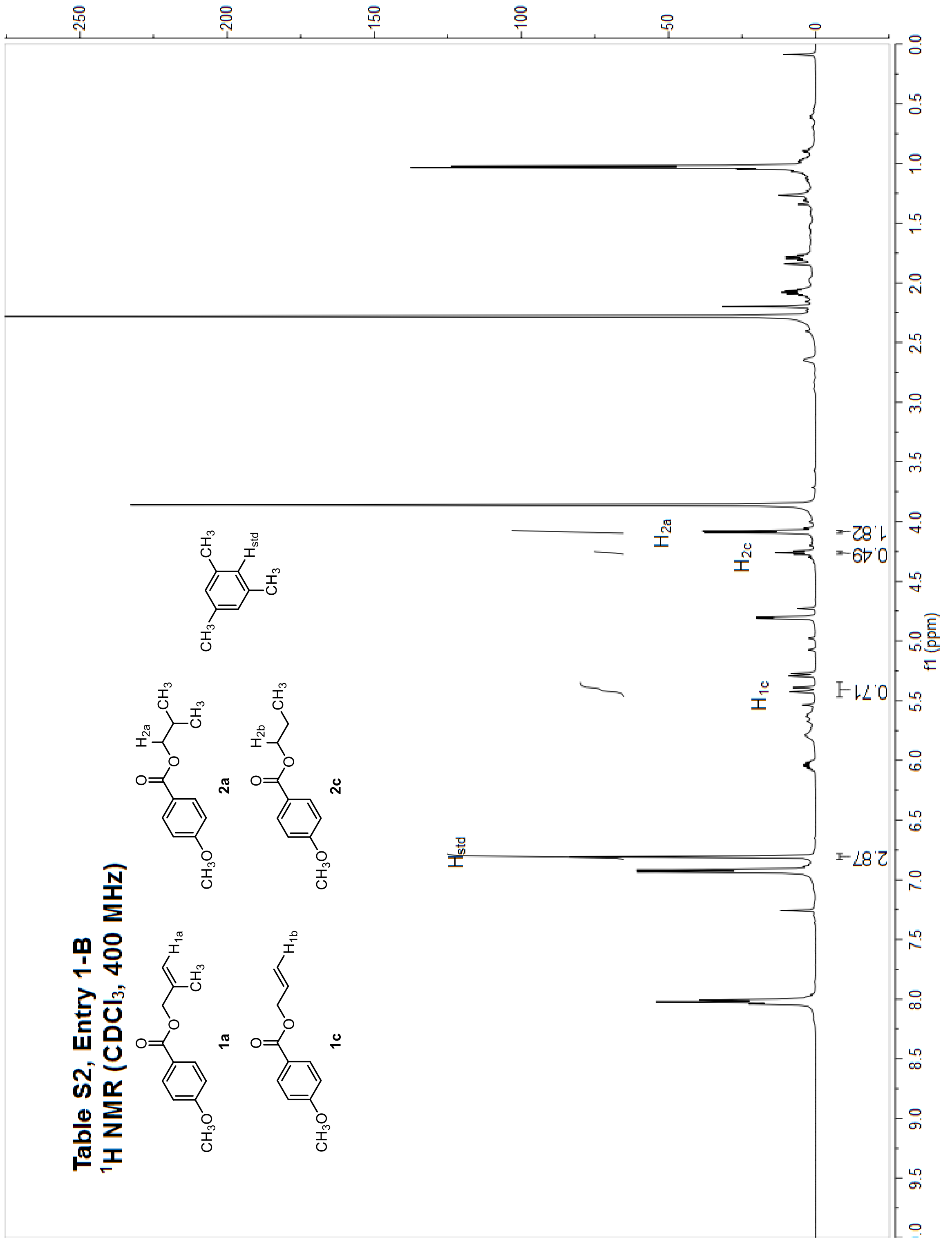
Table S2, Entry 1-B
1H NMR (CDCl3, 400 MHz)
1a
1c
2a
2c
H2a
H2c
H1c
Hstd
1.82
0.49
0.71
2.87
f1 (ppm)

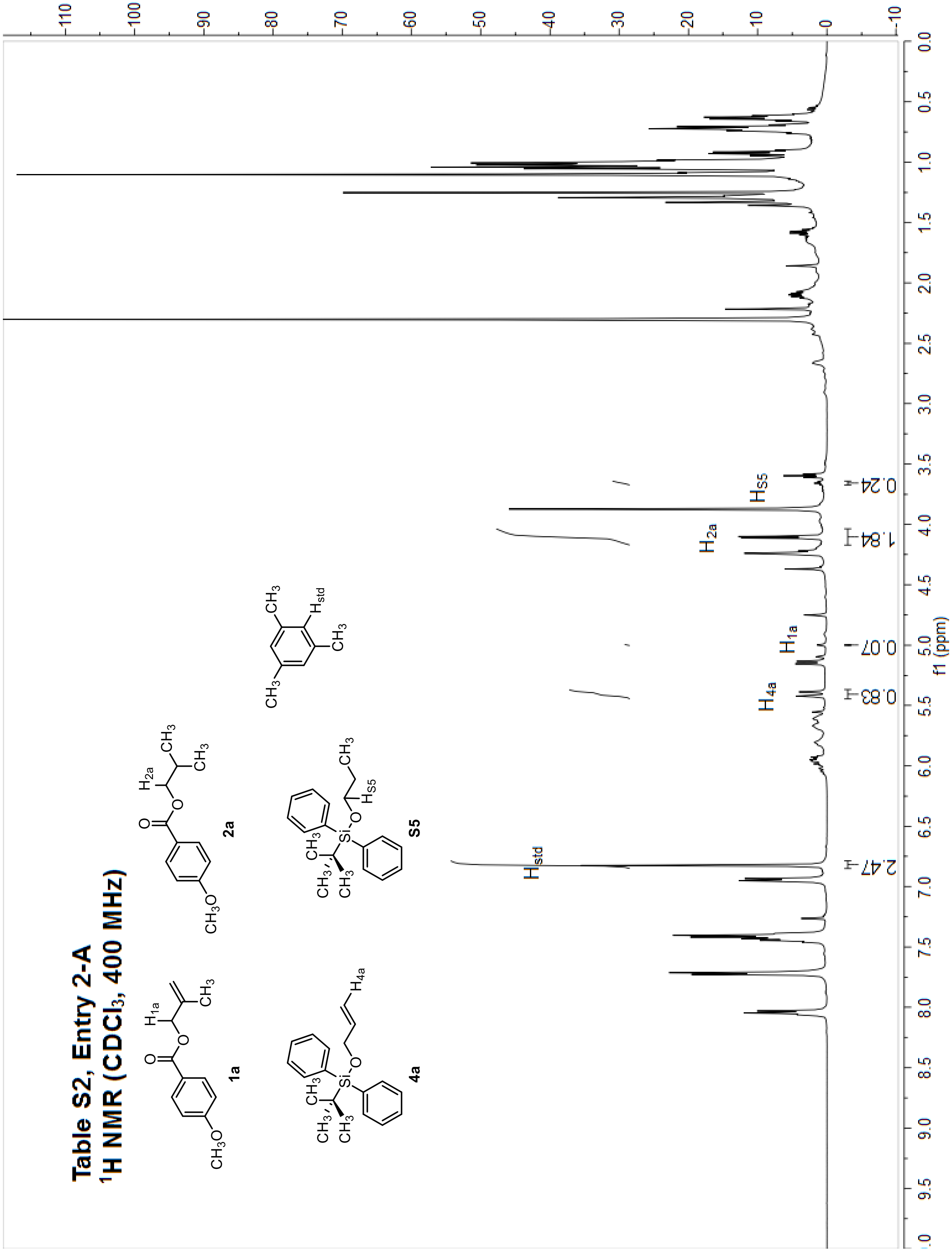

Table S2, Entry 2-A
^{1}H NMR ($CDCl_3$, 400 MHz)
1a
2a
4a
S5
H_{1a}
H_{2a}
H_{4a}
H_{S5}
H_{std}
0.24
1.84
0.07
0.83
2.47
f1 (ppm)

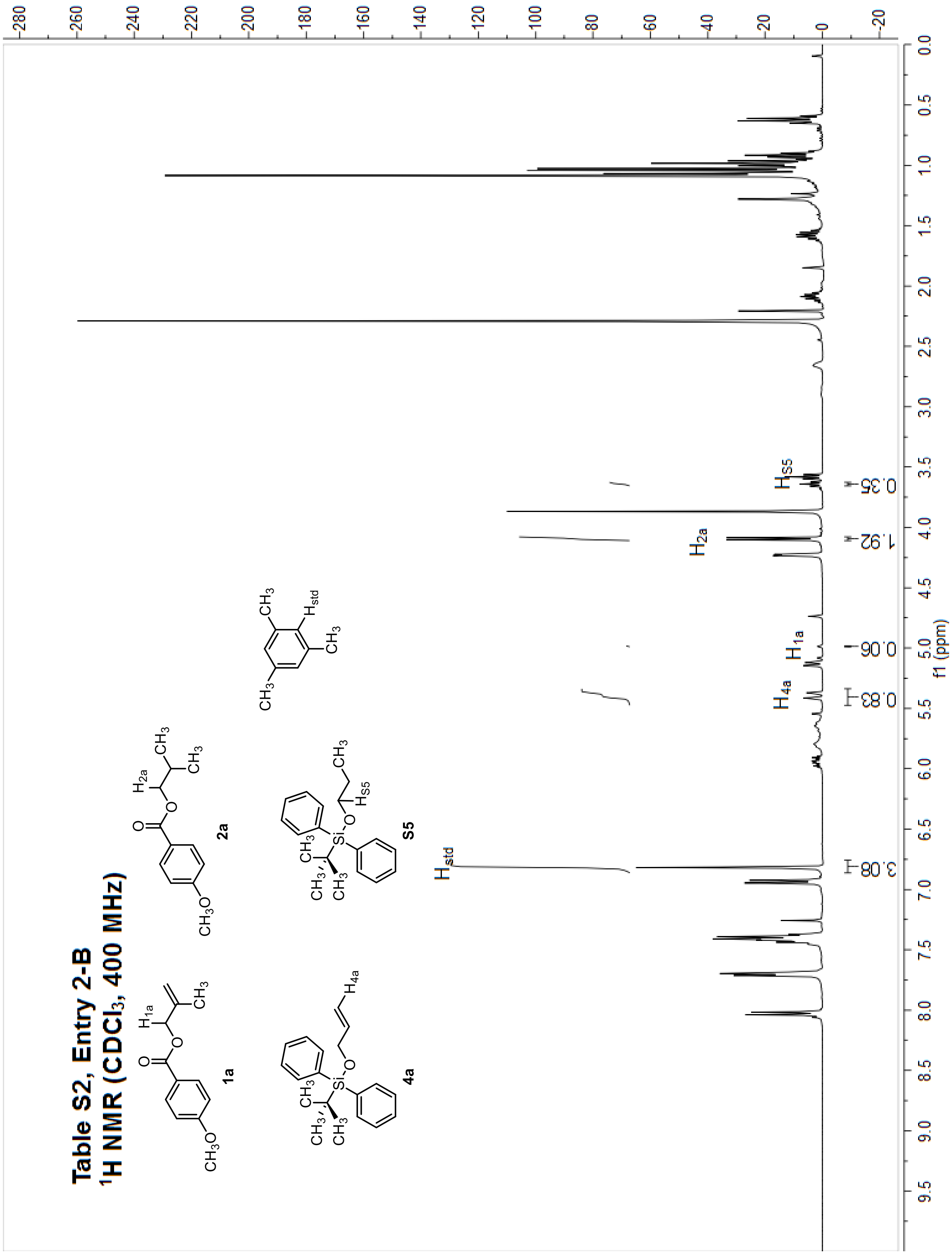
Table S2, Entry 2-B
¹H NMR (CDCl₃, 400 MHz)
1a
2a
4a
S5
H1a
H2a
H4a
HS5
Hstd
0.35
1.92
0.06
0.83
3.08
f1 (ppm)

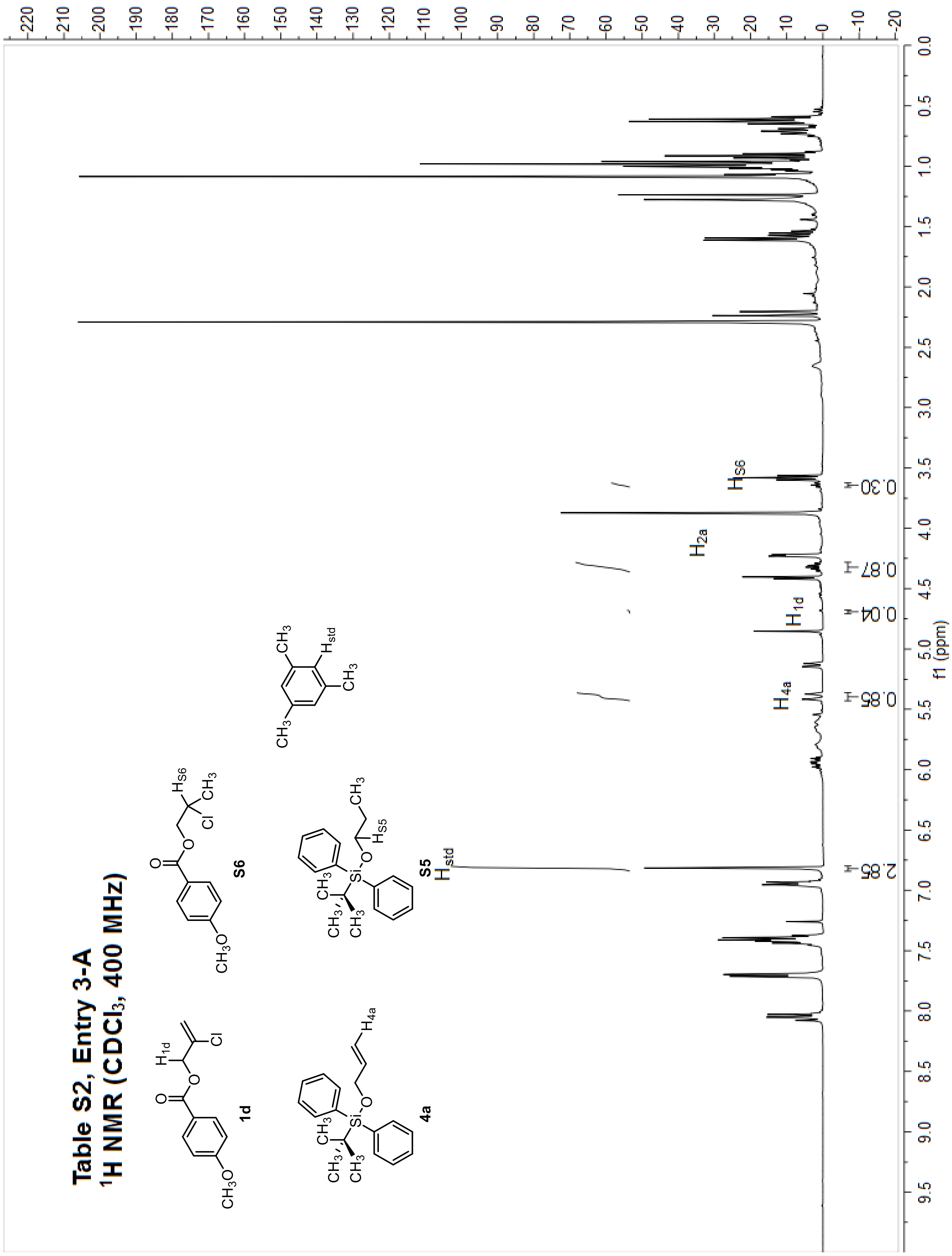
Table S2, Entry 3-A
1H NMR (CDCl3, 400 MHz)
1d
4a
S6
S5
H1d
H4a
H2a
HS6
Hstd
0.30
0.87
0.04
0.85
2.85
f1 (ppm)

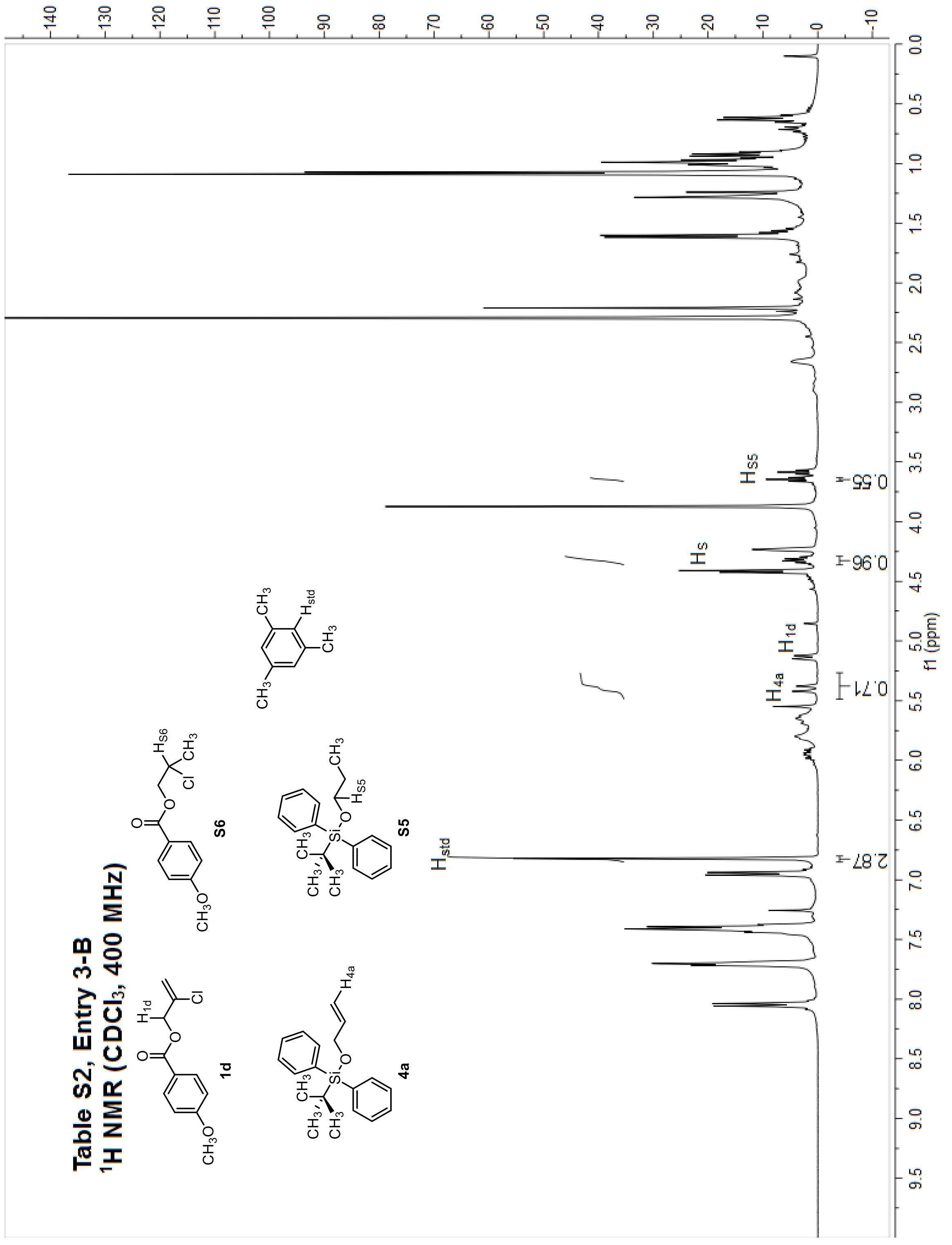
Table S2, Entry 3-B
¹H NMR (CDCl₃, 400 MHz)
1d
4a
S6
S5
H1d
H4a
HS5
HS6
Hstd
HS
0.55
0.96
0.71
2.87
f1 (ppm)

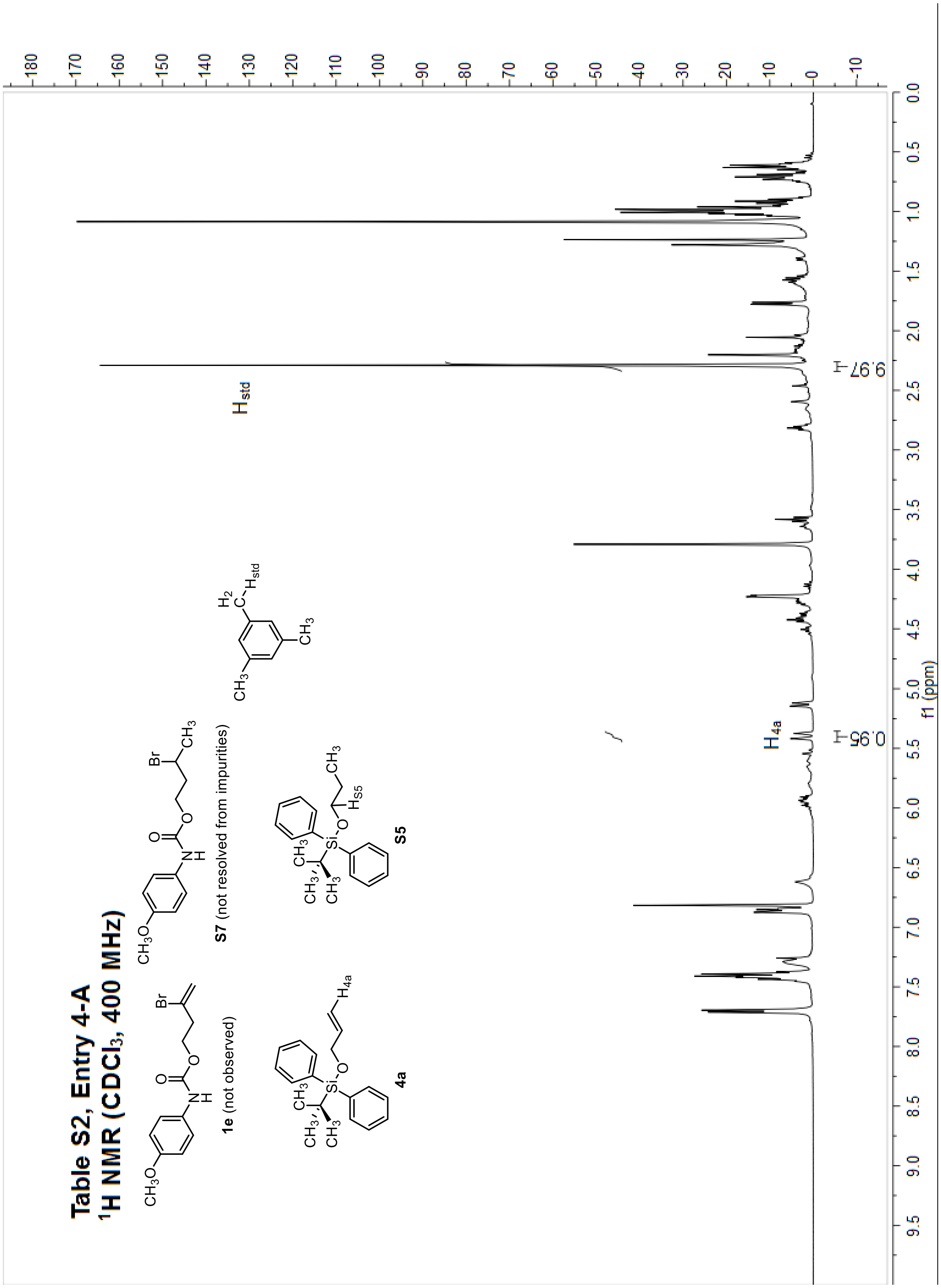

Table S2, Entry 4-A
1H NMR (CDCl3, 400 MHz)
1e (not observed)
S7 (not resolved from impurities)
4a
S5
H4a
Hstd
0.95
9.97
f1 (ppm)

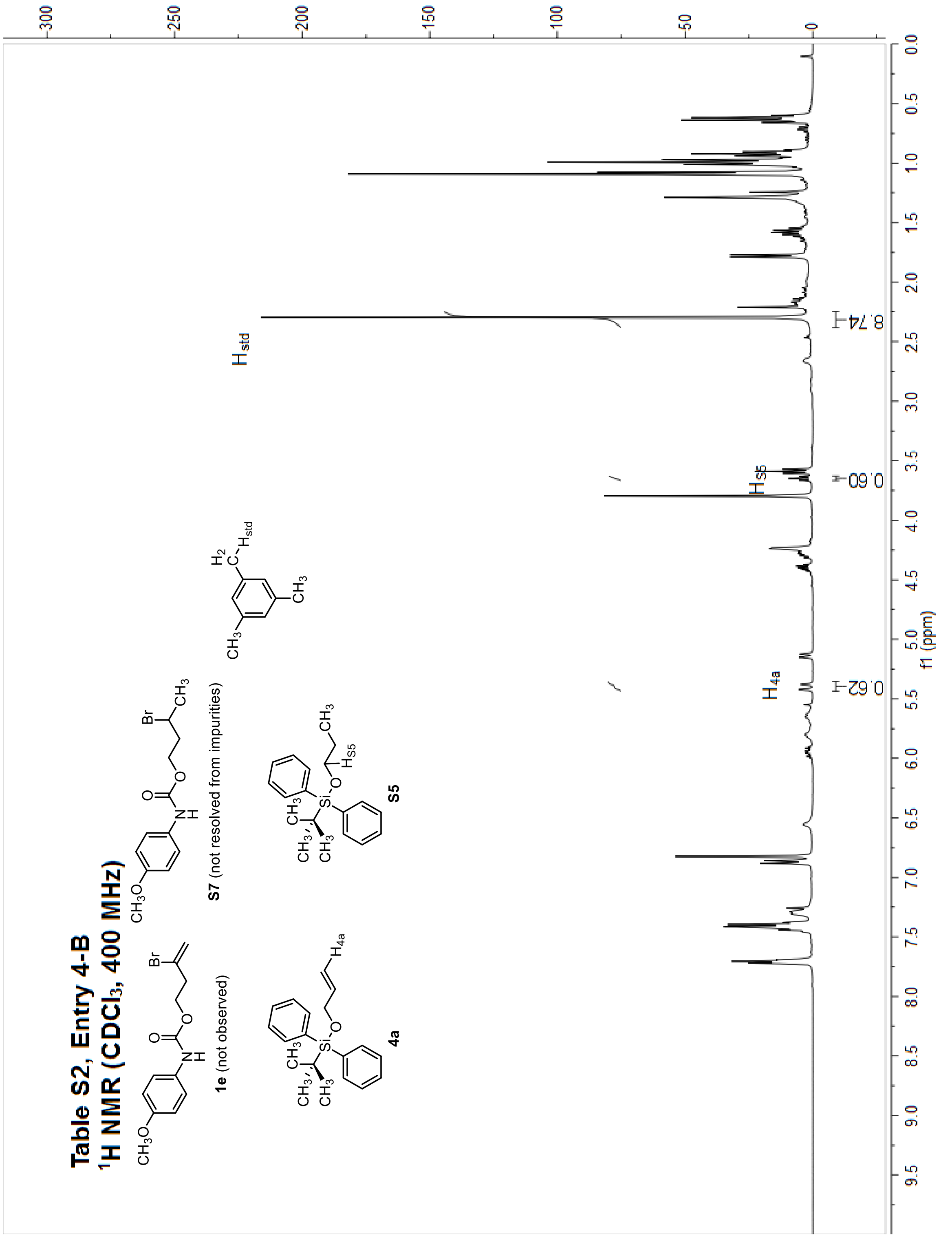
Table S2, Entry 4-B
1H NMR (CDCl3, 400 MHz)
1e (not observed)
S7 (not resolved from impurities)
4a
S5
Hstd
HS5
H4a
8.74
0.60
0.62
f1 (ppm)

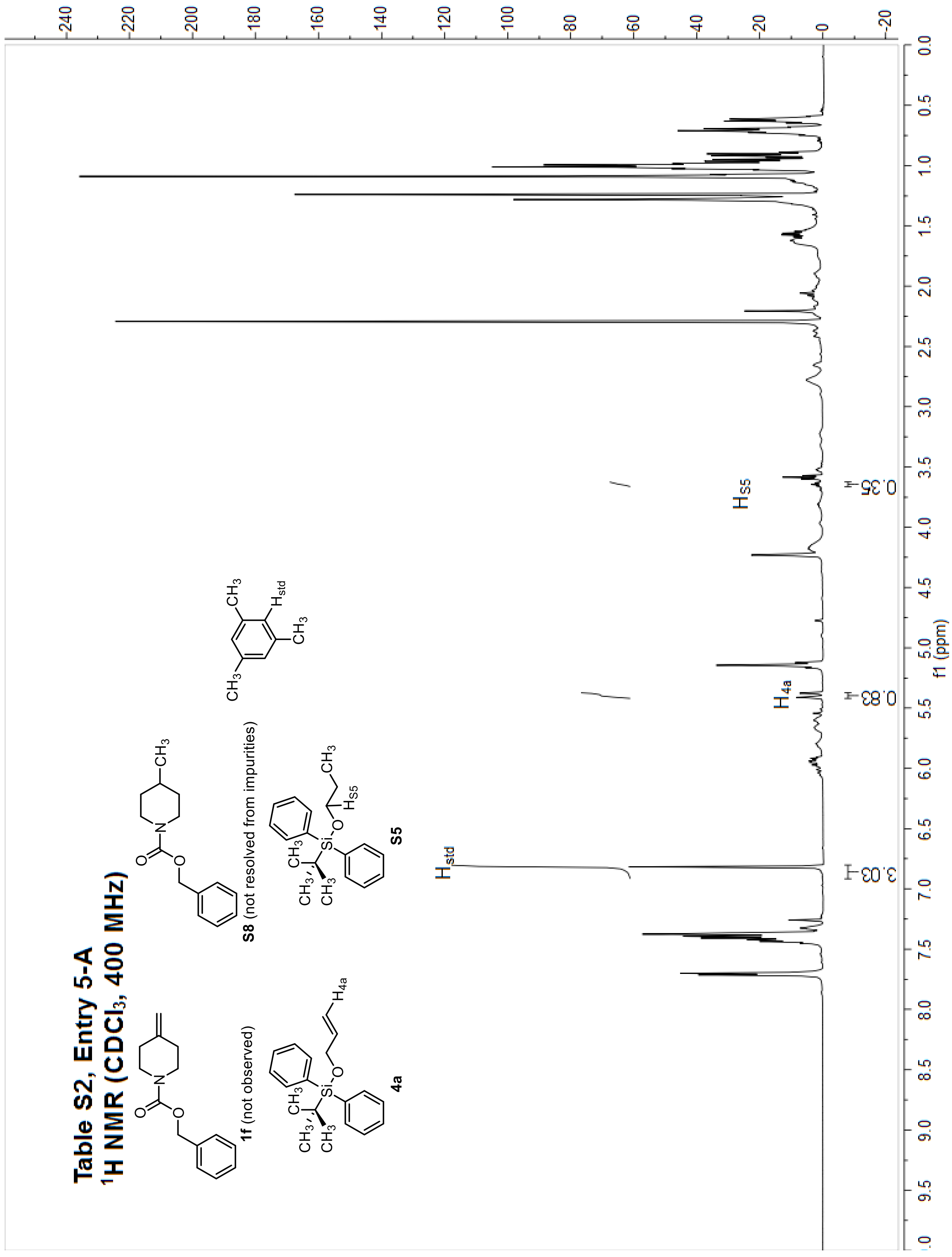

Table S2, Entry 5-A
1H NMR (CDCl3, 400 MHz)
1f (not observed)
S8 (not resolved from impurities)
4a
S5
H4a
HS5
Hstd
0.35
0.83
3.03
f1 (ppm)

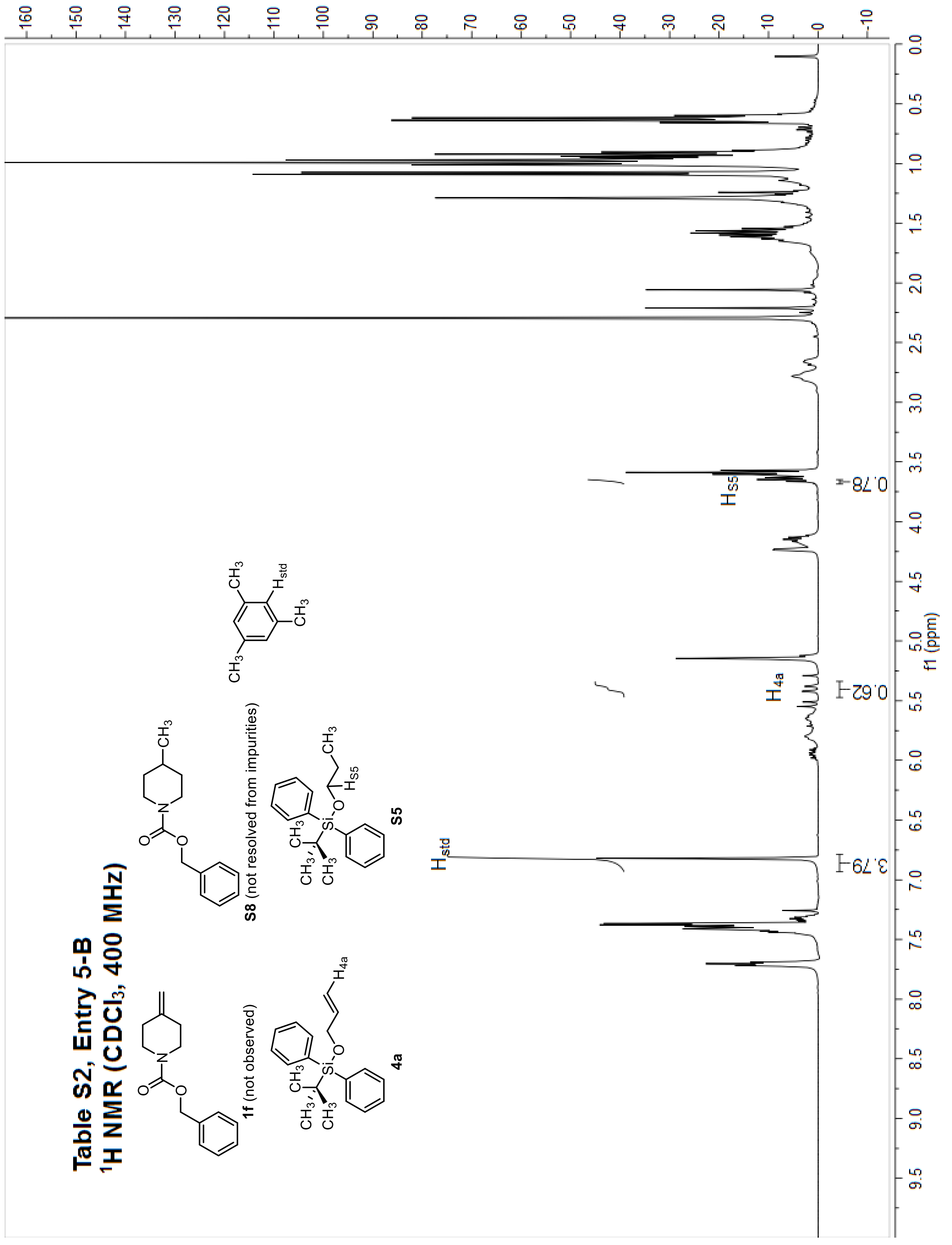
Table S2, Entry 5-B
^{1}H NMR ($CDCl_3$, 400 MHz)
1f (not observed)
S8 (not resolved from impurities)
4a
S5
H_{4a}
H_{S5}
H_{std}
0.78
0.62
3.79
f1 (ppm)

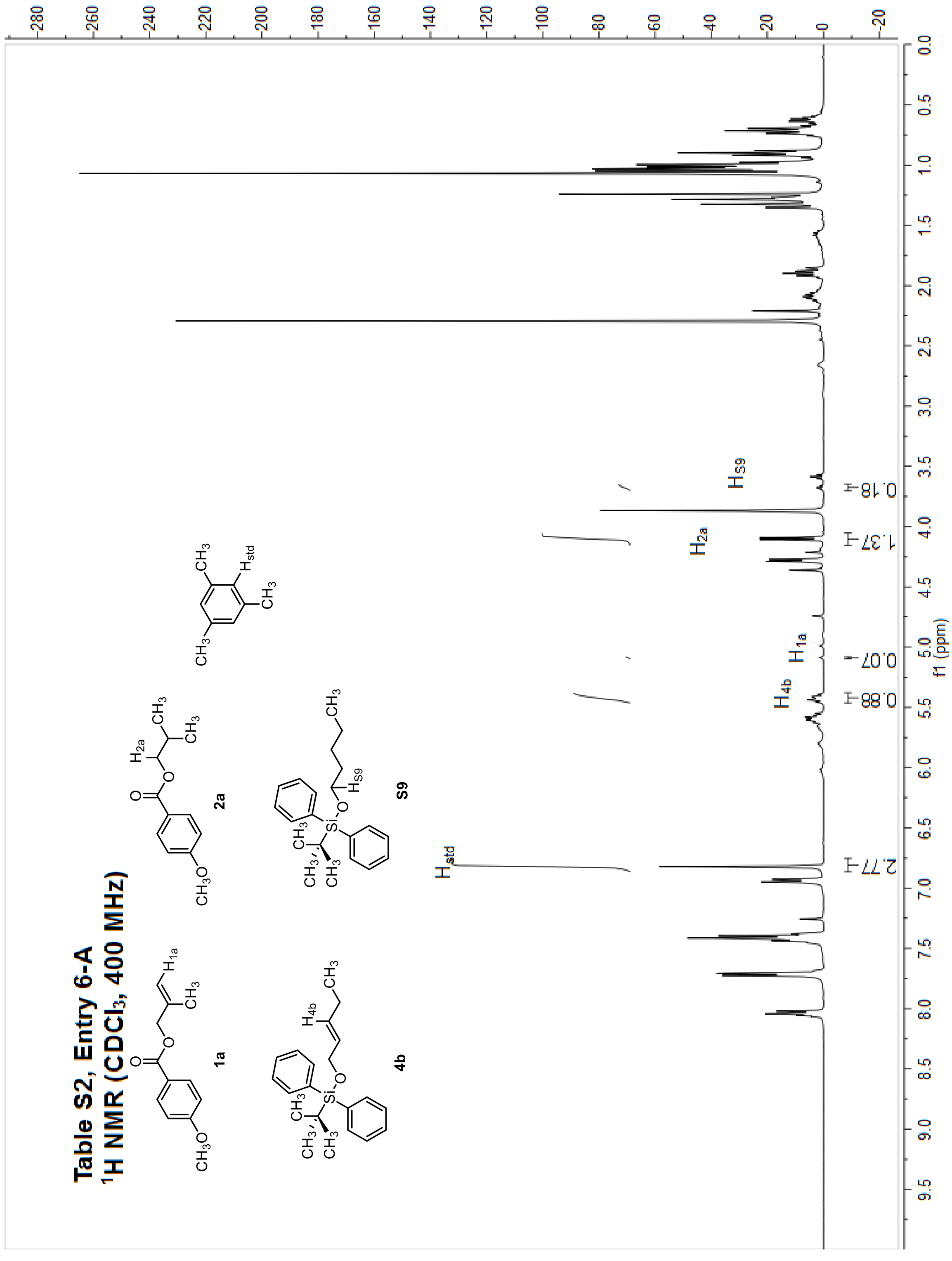
Table S2, Entry 6-A
1H NMR (CDCl3, 400 MHz)
1a
2a
4b
S9
Hstd
HS9
H2a
H1a
H4b
0.18
1.37
0.07
0.88
2.77
f1 (ppm)

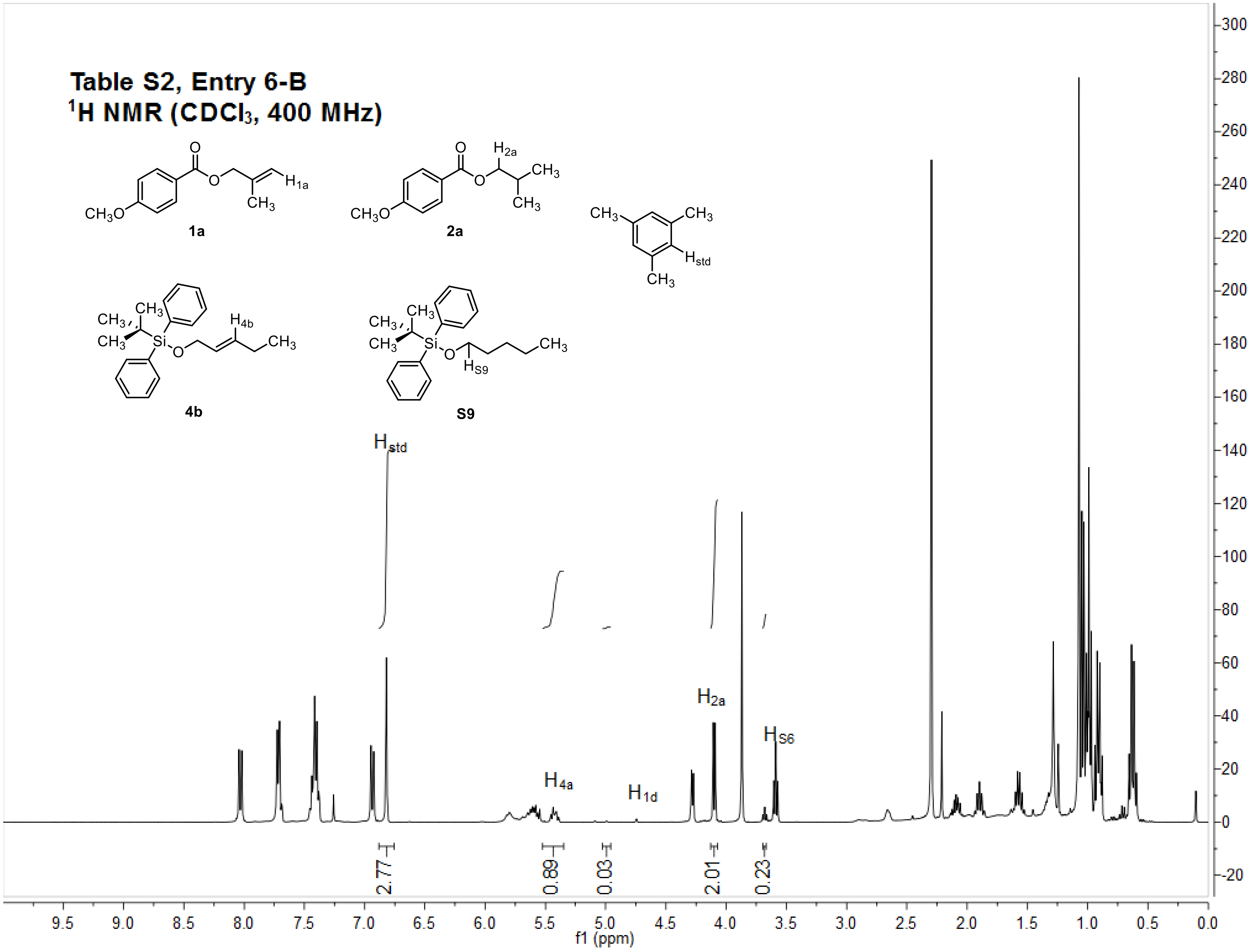
Table S2, Entry 6-B
^{1}H NMR ($CDCl_3$, 400 MHz)
1a
2a
4b
S9
H1a
H2a
H4b
HS9
Hstd
H4a
H1d
HS6
2.77
0.89
0.03
2.01
0.23
f1 (ppm)

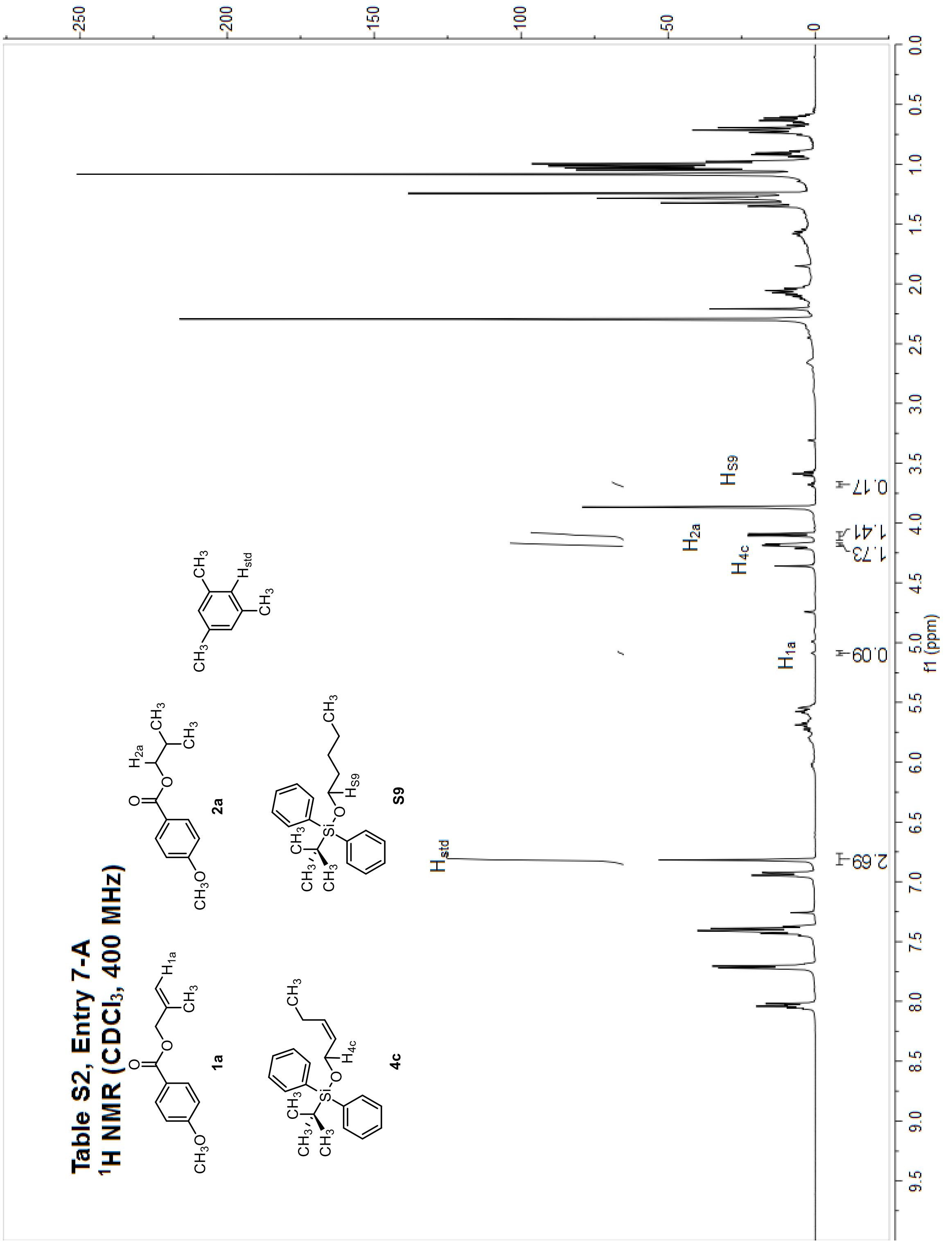
Table S2, Entry 7-A
^{1}H NMR (CDCl$_3$, 400 MHz)
1a
2a
4c
S9
H$_{1a}$
H$_{2a}$
H$_{4c}$
H$_{S9}$
H$_{std}$
0.17
1.41
1.73
0.09
2.69
f1 (ppm)

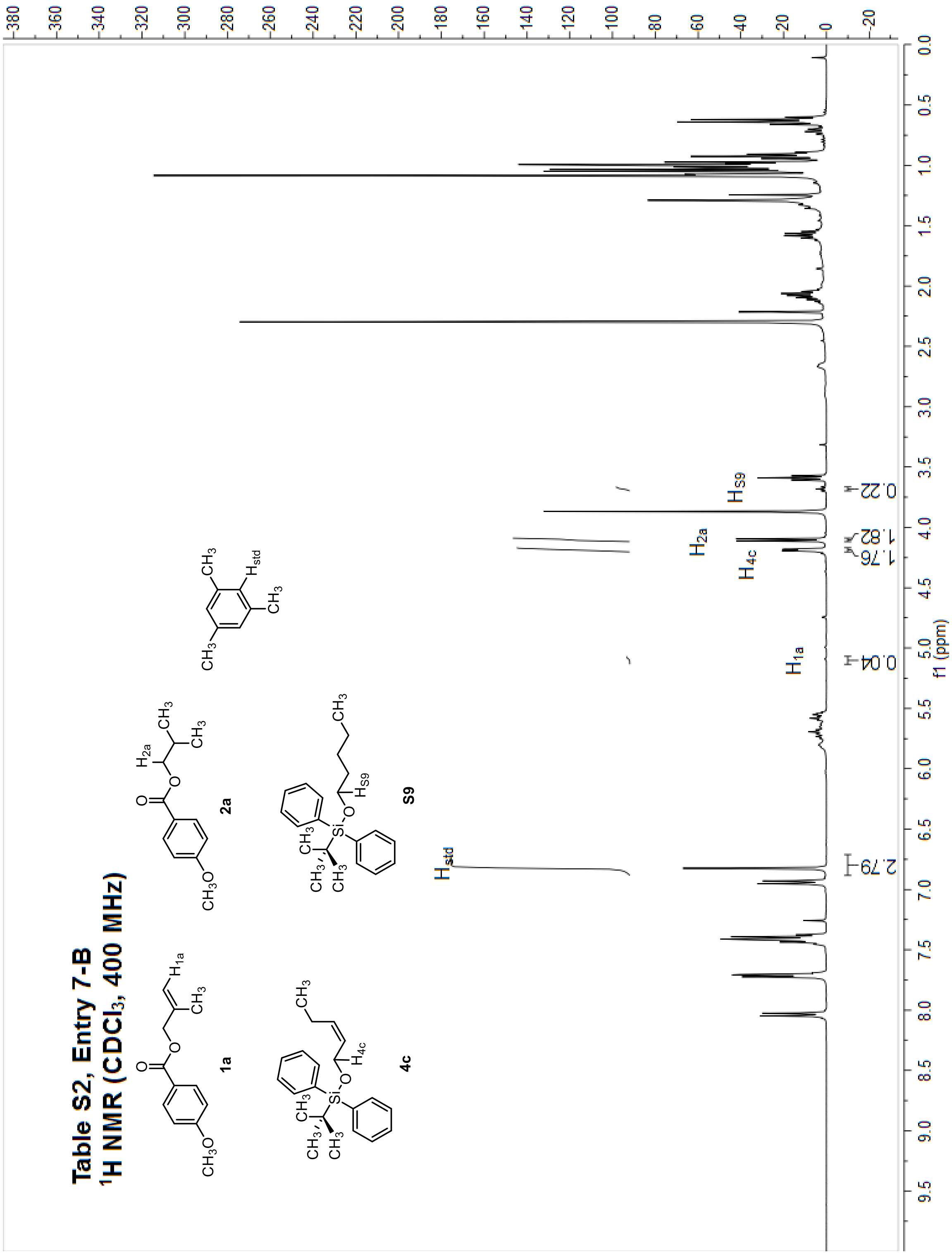
Table S2, Entry 7-B
1H NMR (CDCl3, 400 MHz)
1a
2a
4c
S9
H1a
H2a
H4c
HS9
Hstd
0.22
1.82
1.76
0.04
2.79
f1 (ppm)

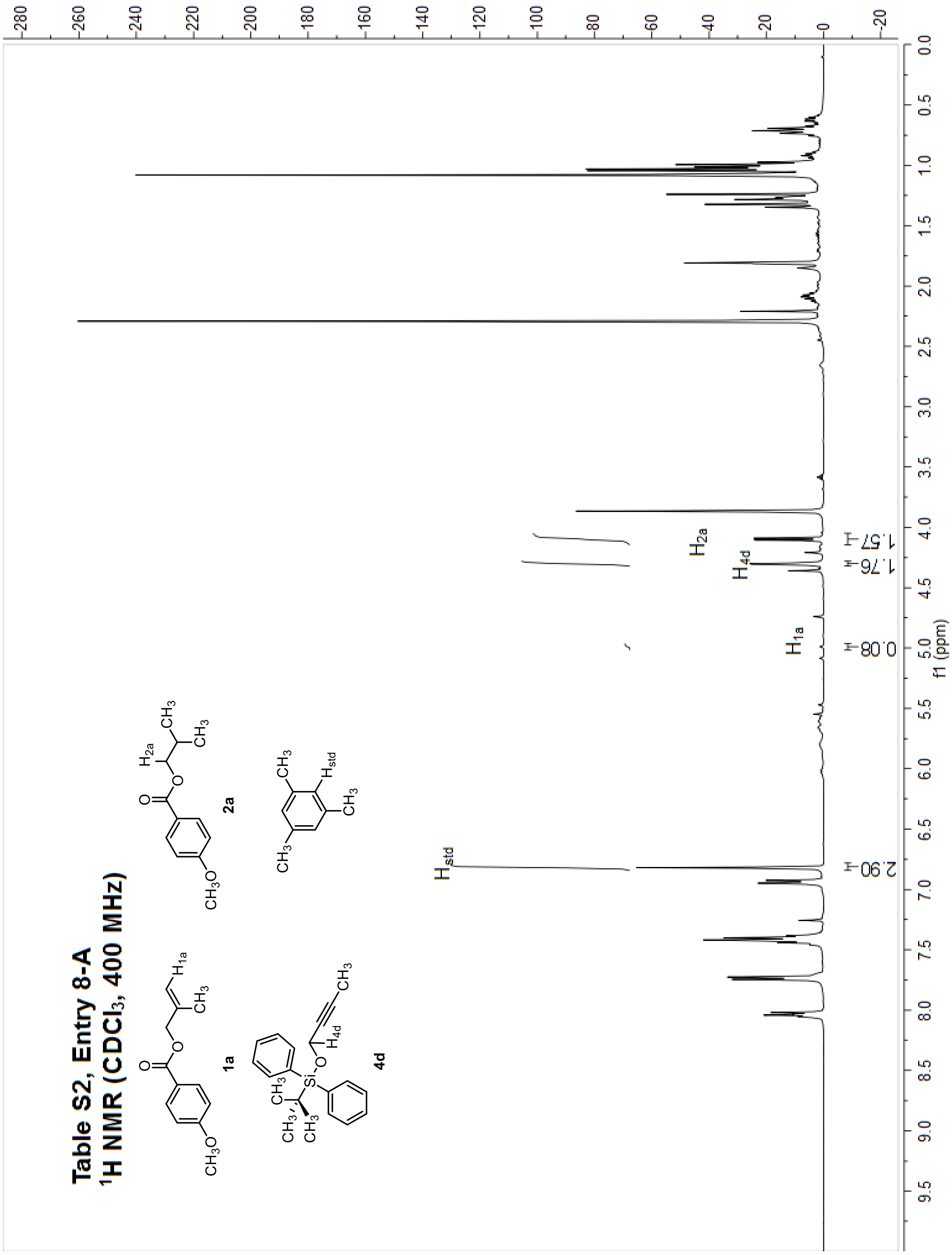

Table S2, Entry 8-A
1H NMR (CDCl3, 400 MHz)
1a
2a
4d
H1a
H2a
H4d
Hstd
f1 (ppm)

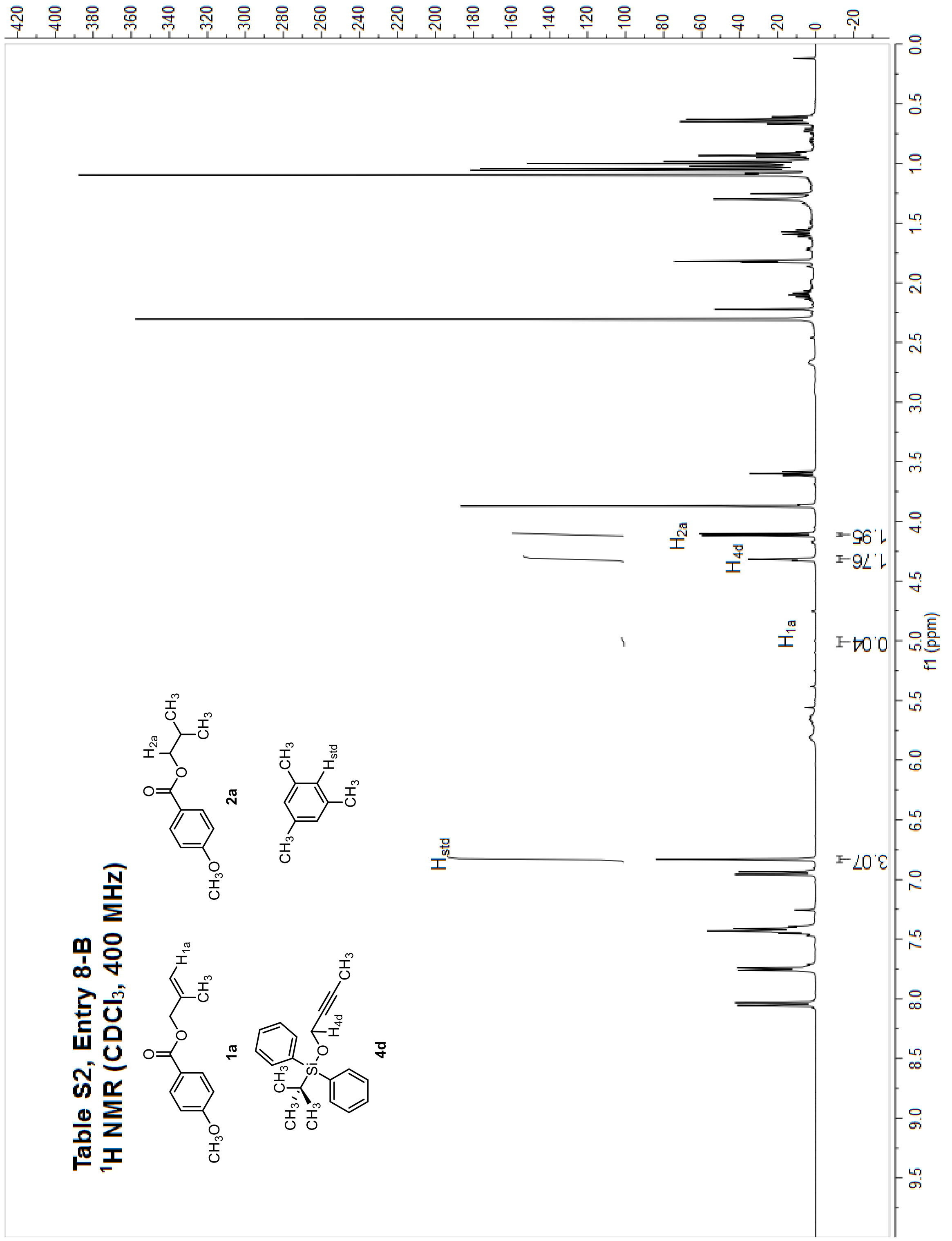
Table S2, Entry 8-B
1H NMR (CDCl3, 400 MHz)
1a
2a
4d
H2a
H4d
H1a
Hstd
1.95
1.76
0.04
3.07
f1 (ppm)

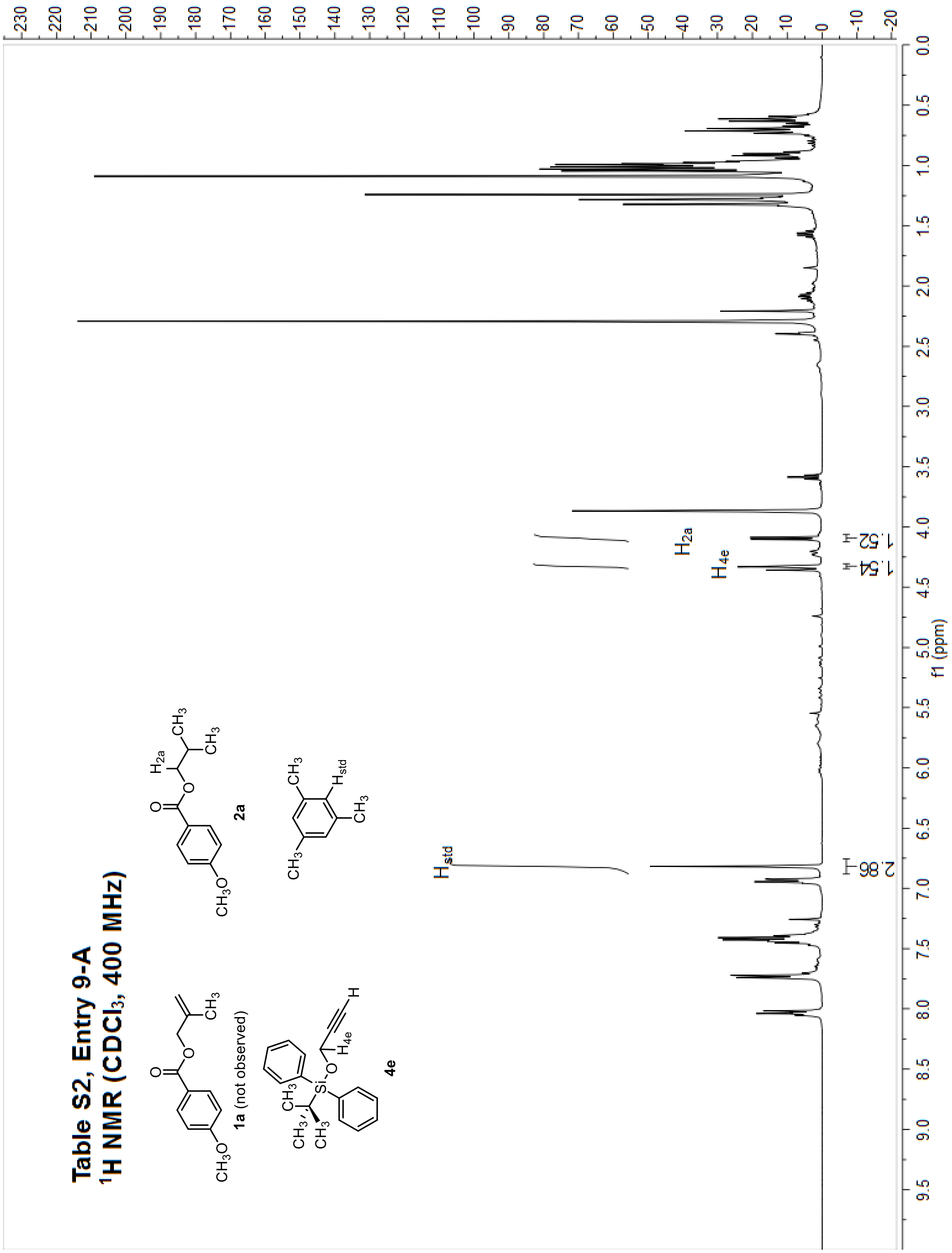
Table S2, Entry 9-A
¹H NMR (CDCl₃, 400 MHz)
1a (not observed)
2a
4e
H2a
H4e
Hstd
1.52
1.54
2.86
f1 (ppm)

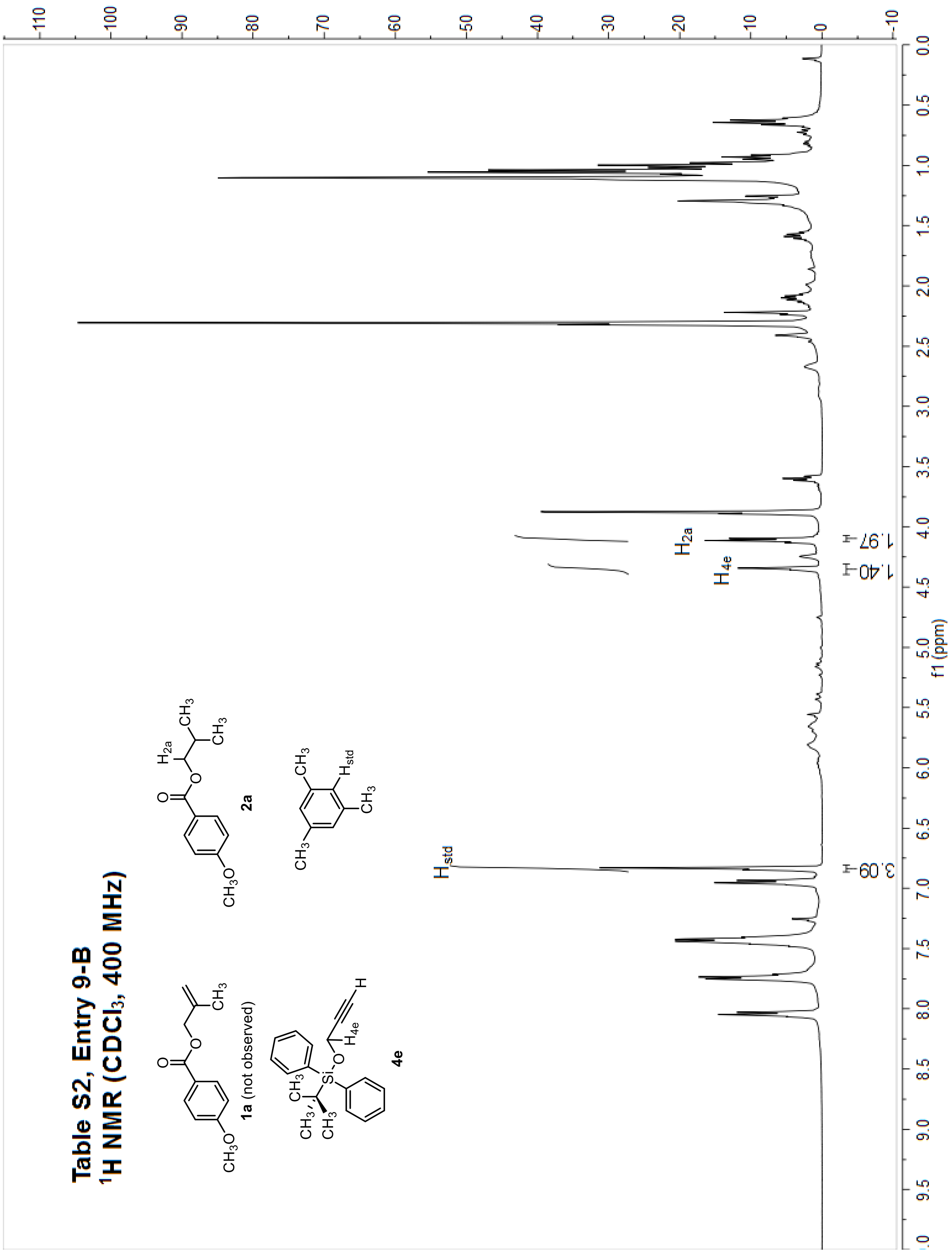
Table S2, Entry 9-B
1H NMR (CDCl3, 400 MHz)
1a (not observed)
2a
4e
H2a
H4e
Hstd
1.97
1.40
3.09
f1 (ppm)

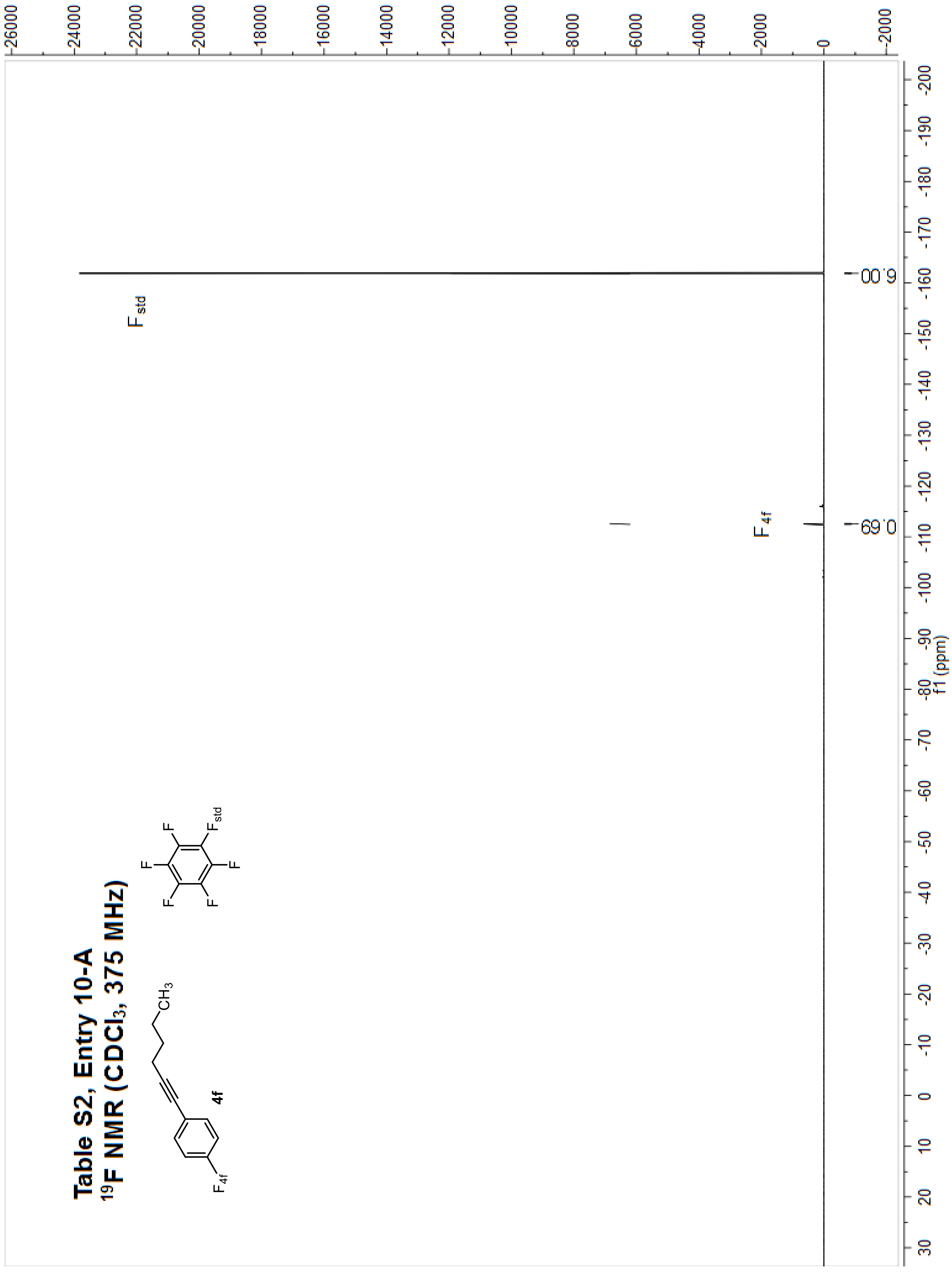

Table S2, Entry 10-A
19F NMR (CDCl3, 375 MHz)
4f
F4f
Fstd
6.00
0.69
f1 (ppm)

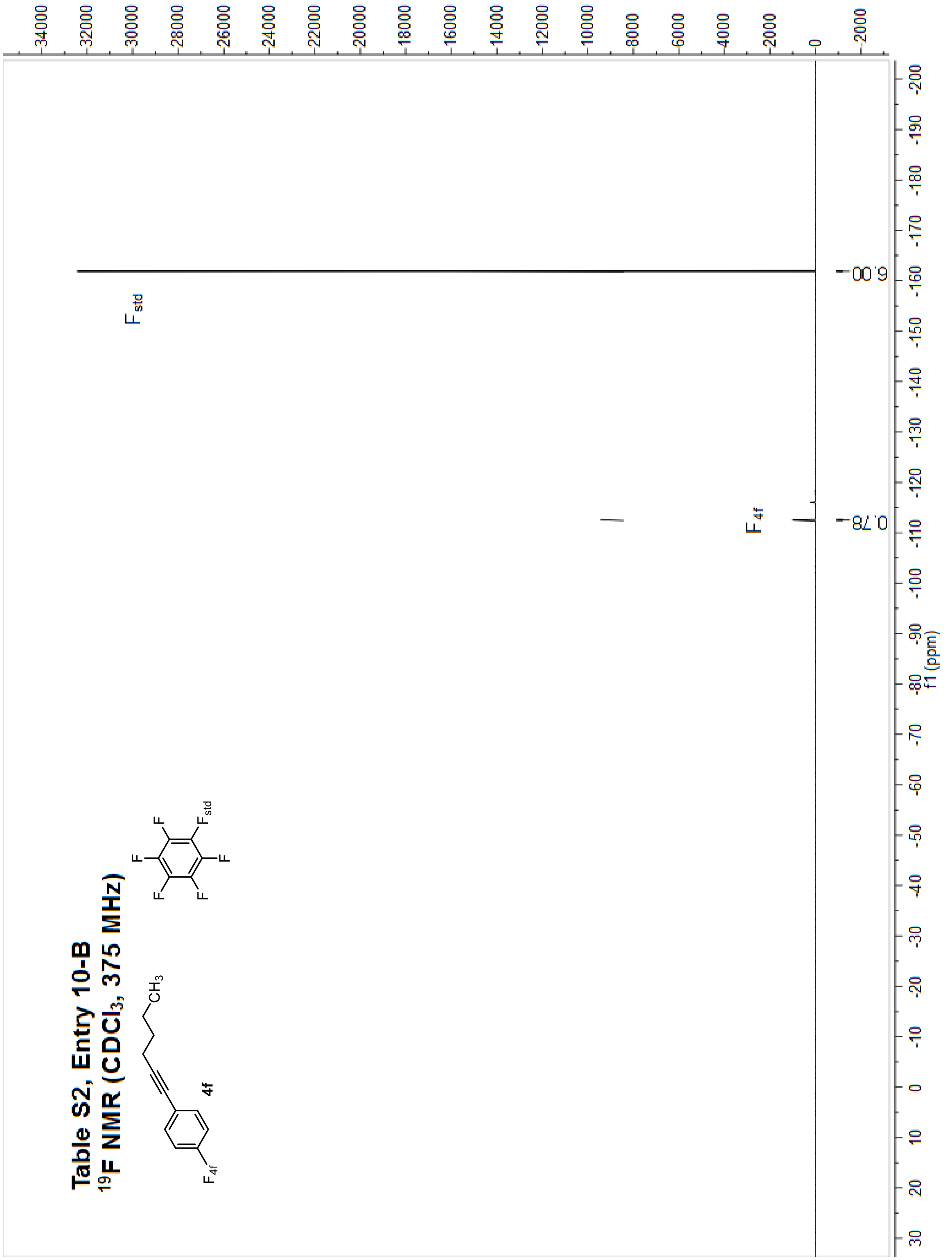
Table S2, Entry 10-B
19F NMR (CDCl3, 375 MHz)
4f
CH3
F4f
Fstd
F4f
6.00
0.78
f1 (ppm)

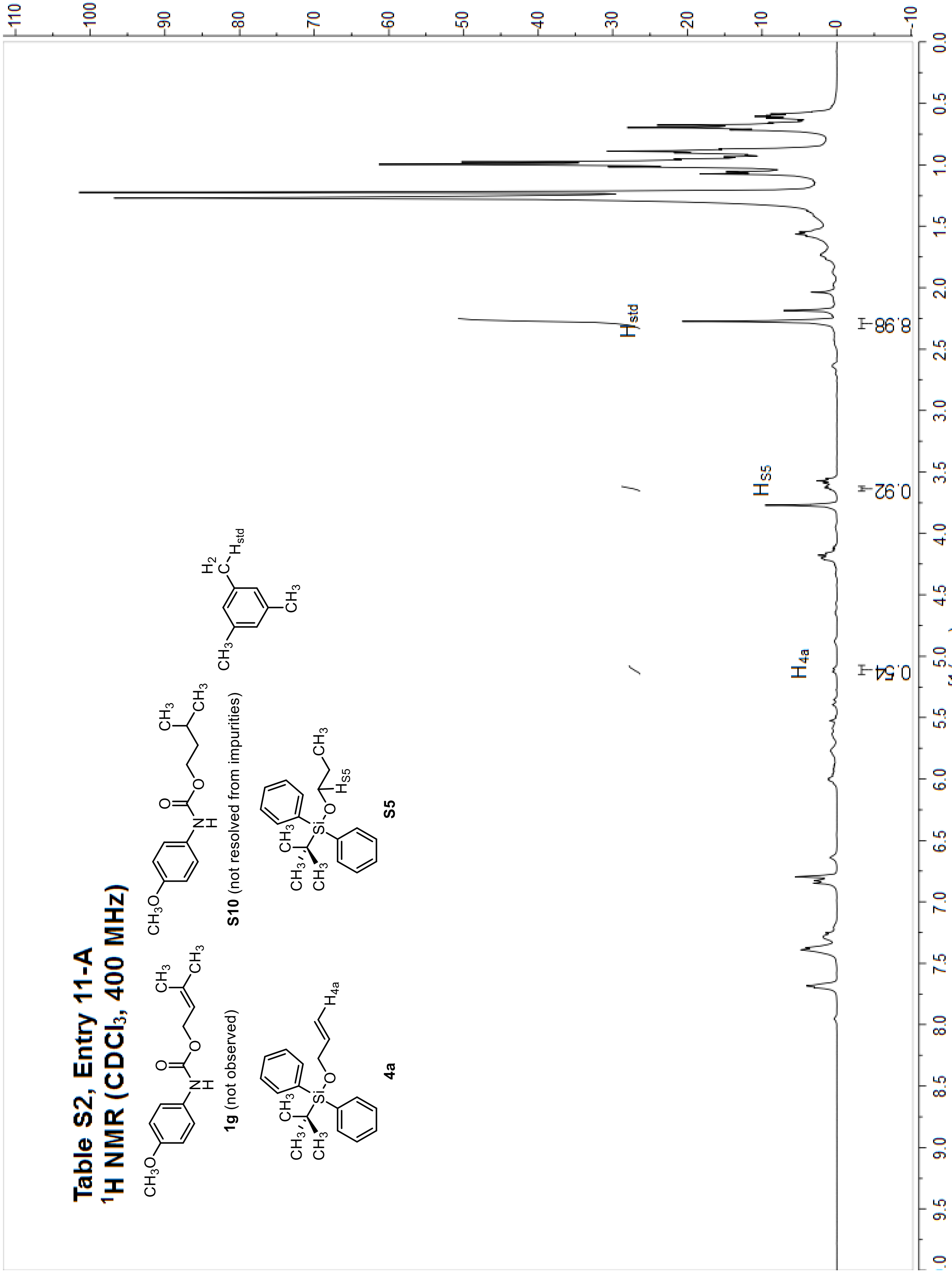

Table S2, Entry 11-A
^{1}H NMR ($CDCl_3$, 400 MHz)
1g (not observed)
S10 (not resolved from impurities)
4a
S5
H_{std}
H_{S5}
H_{4a}
0.98
0.92
0.54
f1 (ppm)

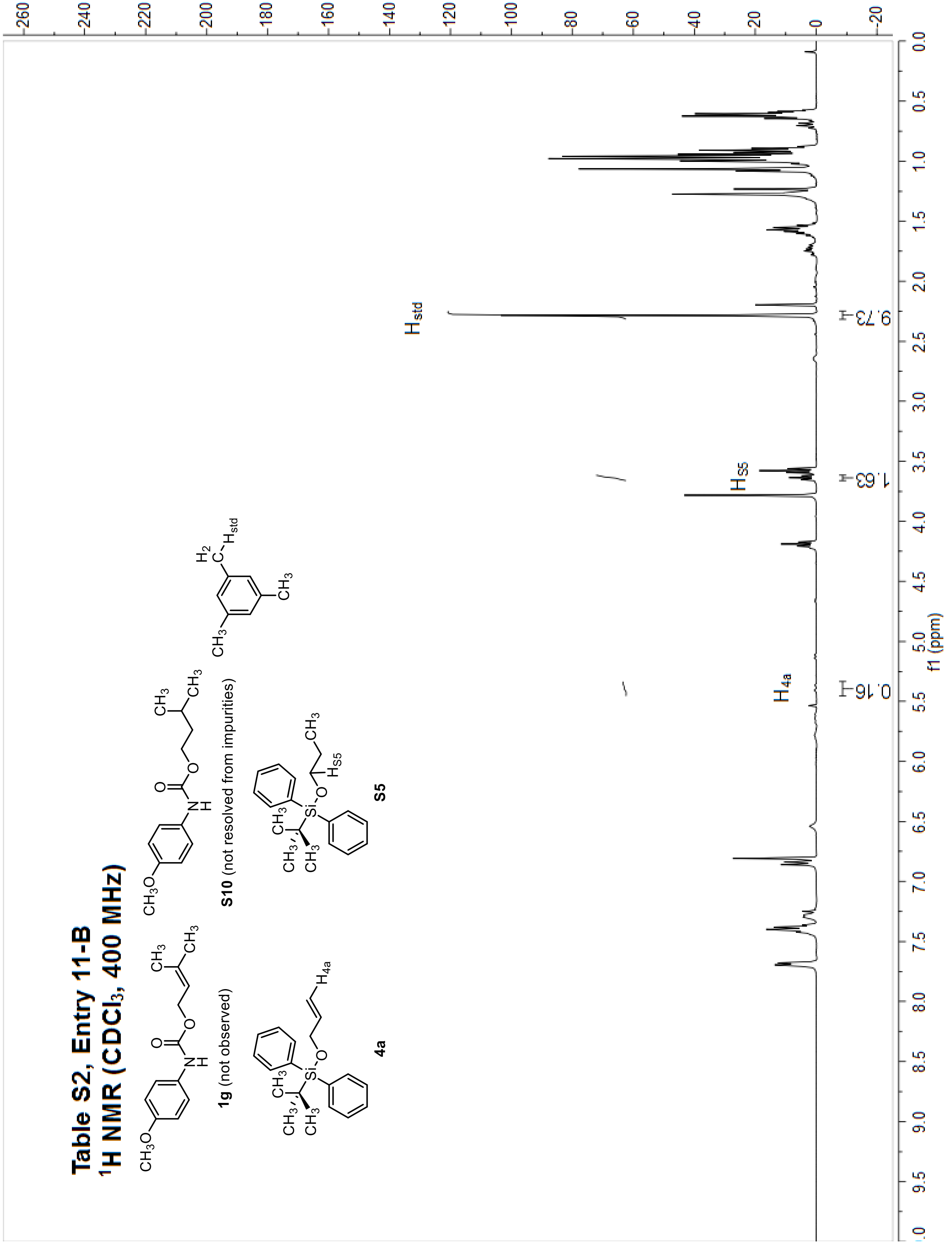
Table S2, Entry 11-B
^{1}H NMR (CDCl$_3$, 400 MHz)
1g (not observed)
S10 (not resolved from impurities)
4a
S5
H$_{std}$
H$_{S5}$
H$_{4a}$
9.73
1.63
0.16
f1 (ppm)

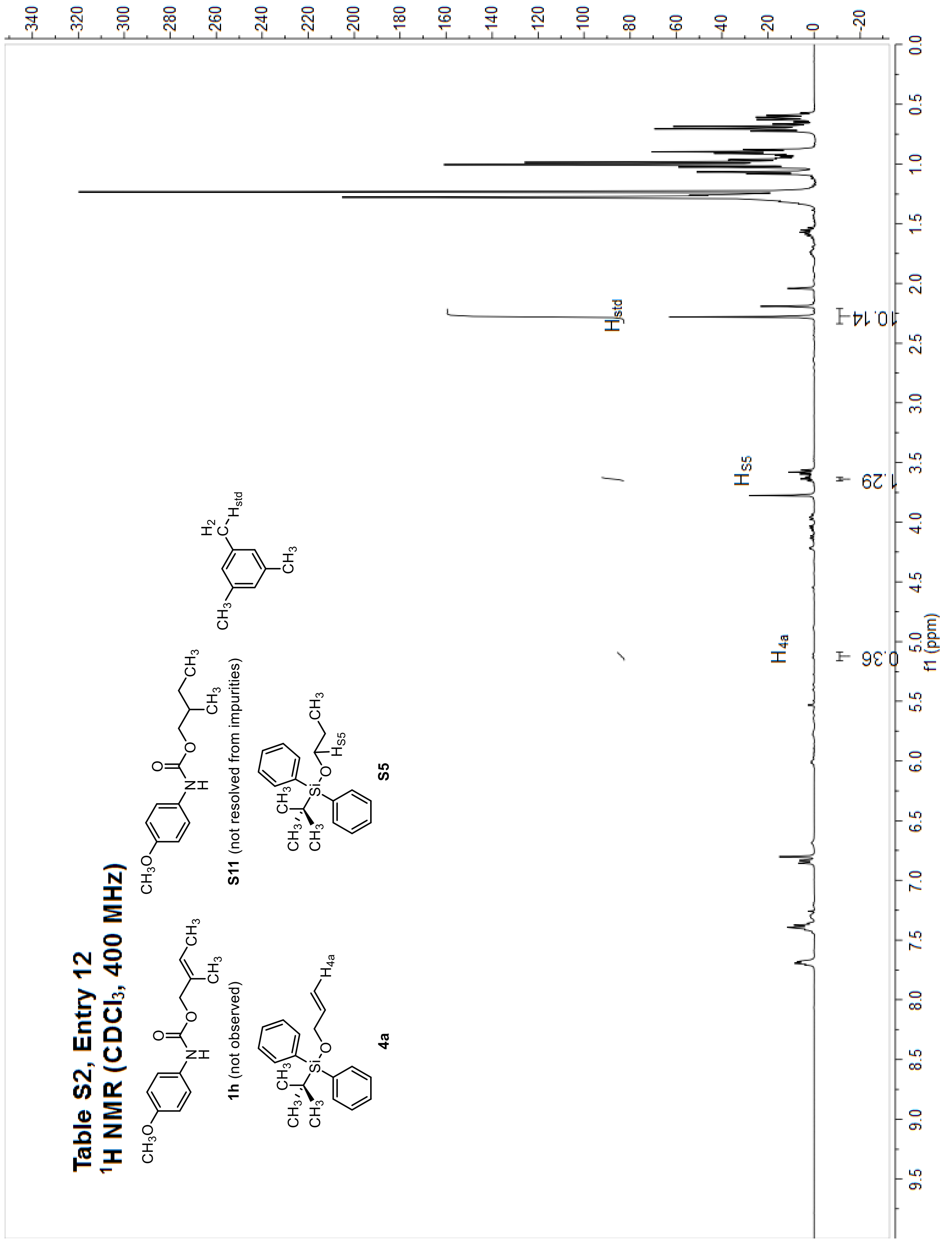

Table S2, Entry 12
^{1}H NMR ($CDCl_3$, 400 MHz)
1h (not observed)
S11 (not resolved from impurities)
4a
S5
H_{4a}
H_{S5}
H_{std}
10.14
1.29
0.36
f1 (ppm)

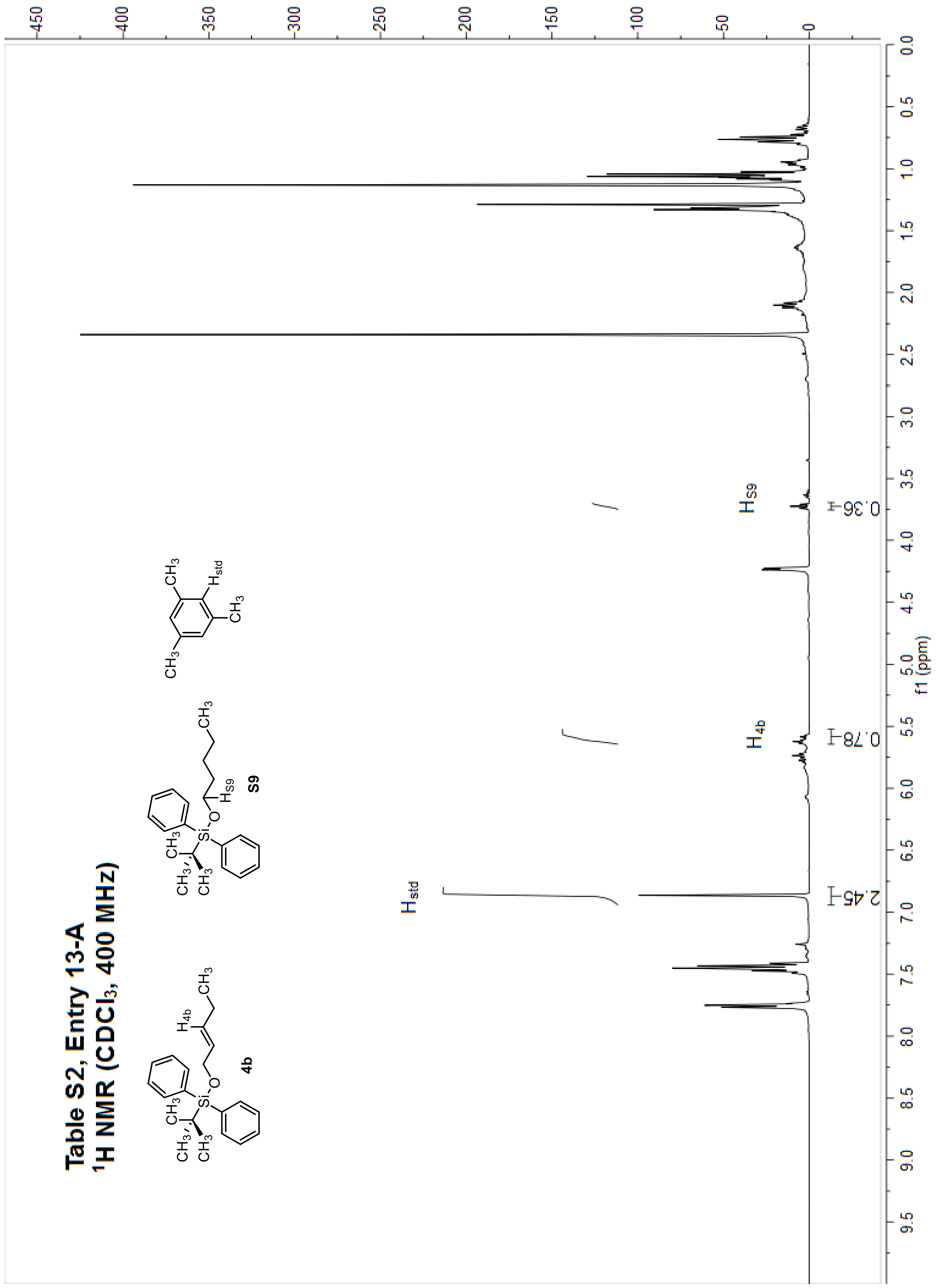
Table S2, Entry 13-A
1H NMR (CDCl3, 400 MHz)
4b
S9
H4b
HS9
Hstd
2.45
0.78
0.36
f1 (ppm)

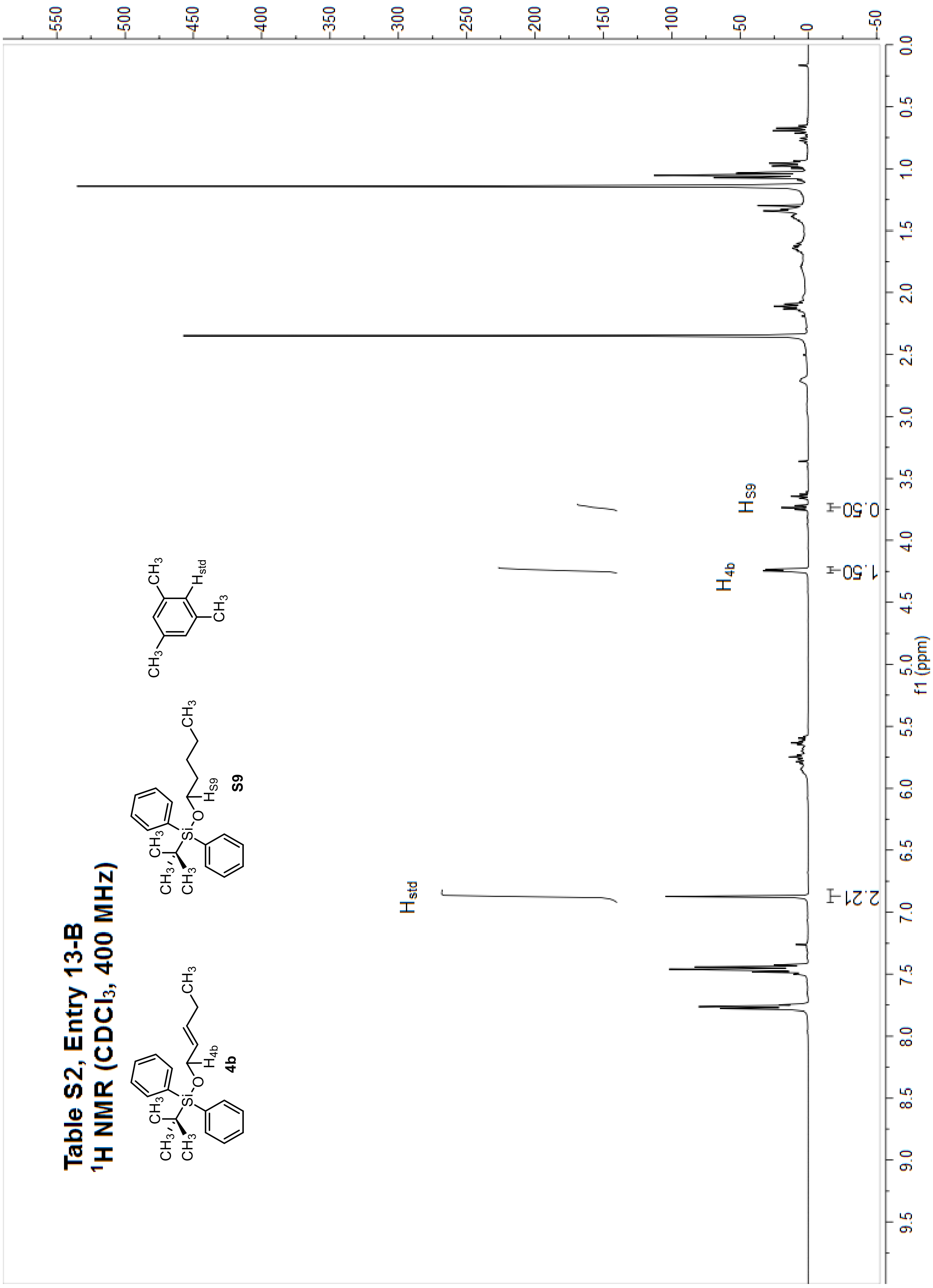
Table S2, Entry 13-B
^{1}H NMR (CDCl$_3$, 400 MHz)
4b
S9
H$_{4b}$
H$_{S9}$
H$_{std}$
0.50
1.50
2.21
f1 (ppm)

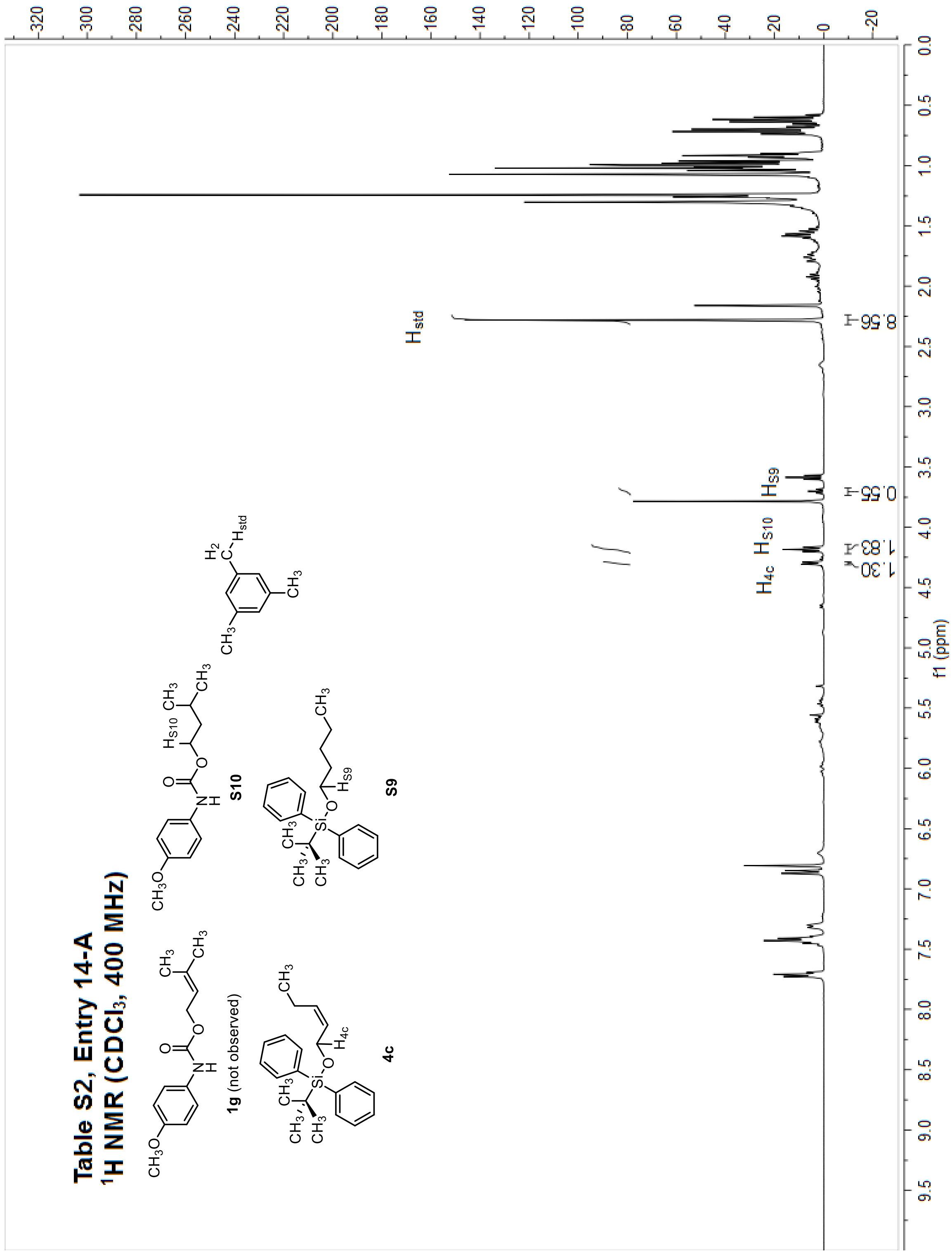
Table S2, Entry 14-A
^{1}H NMR ($CDCl_3$, 400 MHz)
1g (not observed)
S10
S9
4c
H_{std}
H_{S9}
H_{S10}
H_{4c}
8.56
0.55
1.83
1.30
f1 (ppm)

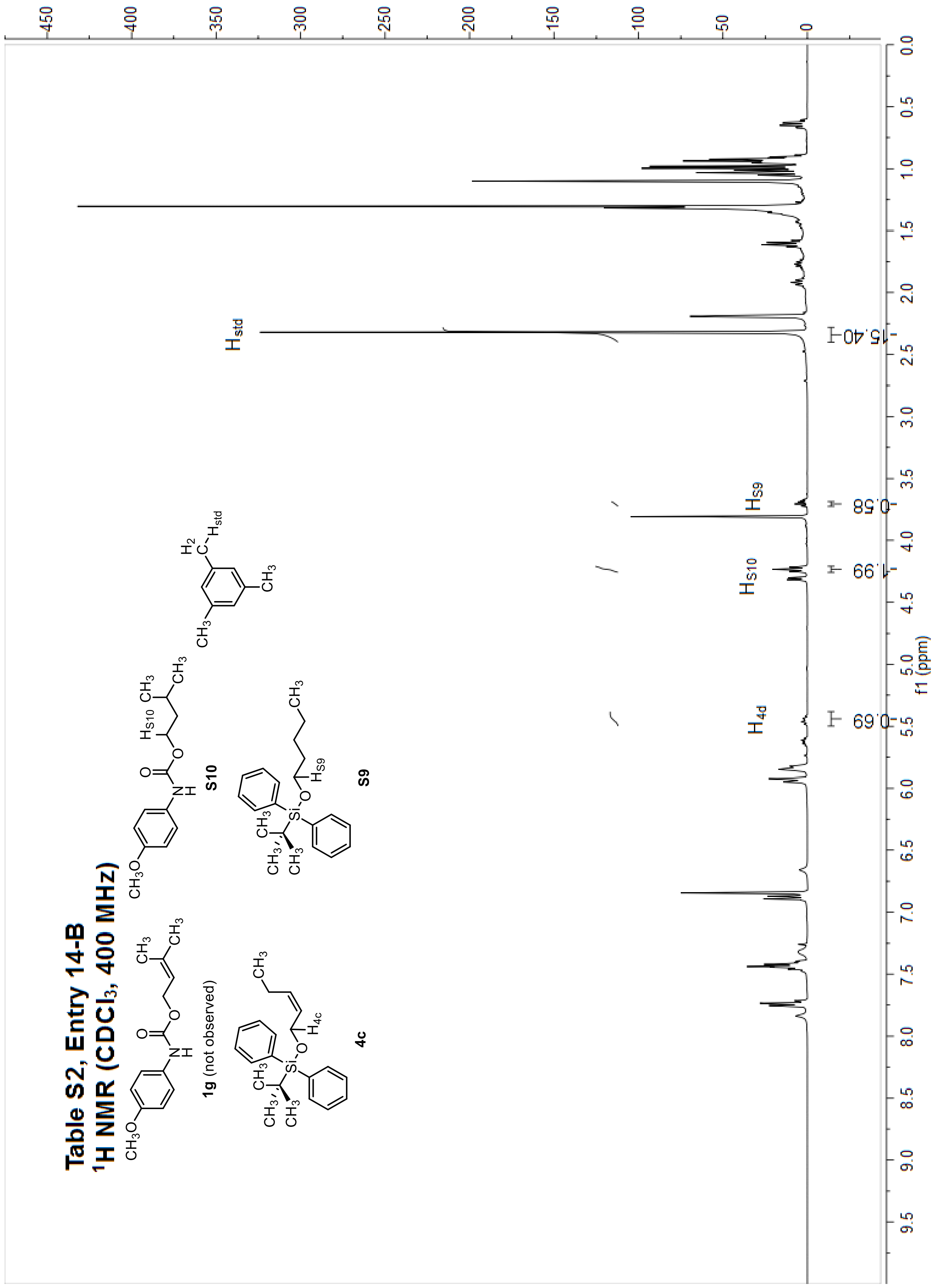

Table S2, Entry 14-B
1H NMR (CDCl3, 400 MHz)
1g (not observed)
4c
S10
S9
Hstd
HS9
HS10
H4d
15.40
0.58
1.99
0.69
f1 (ppm)

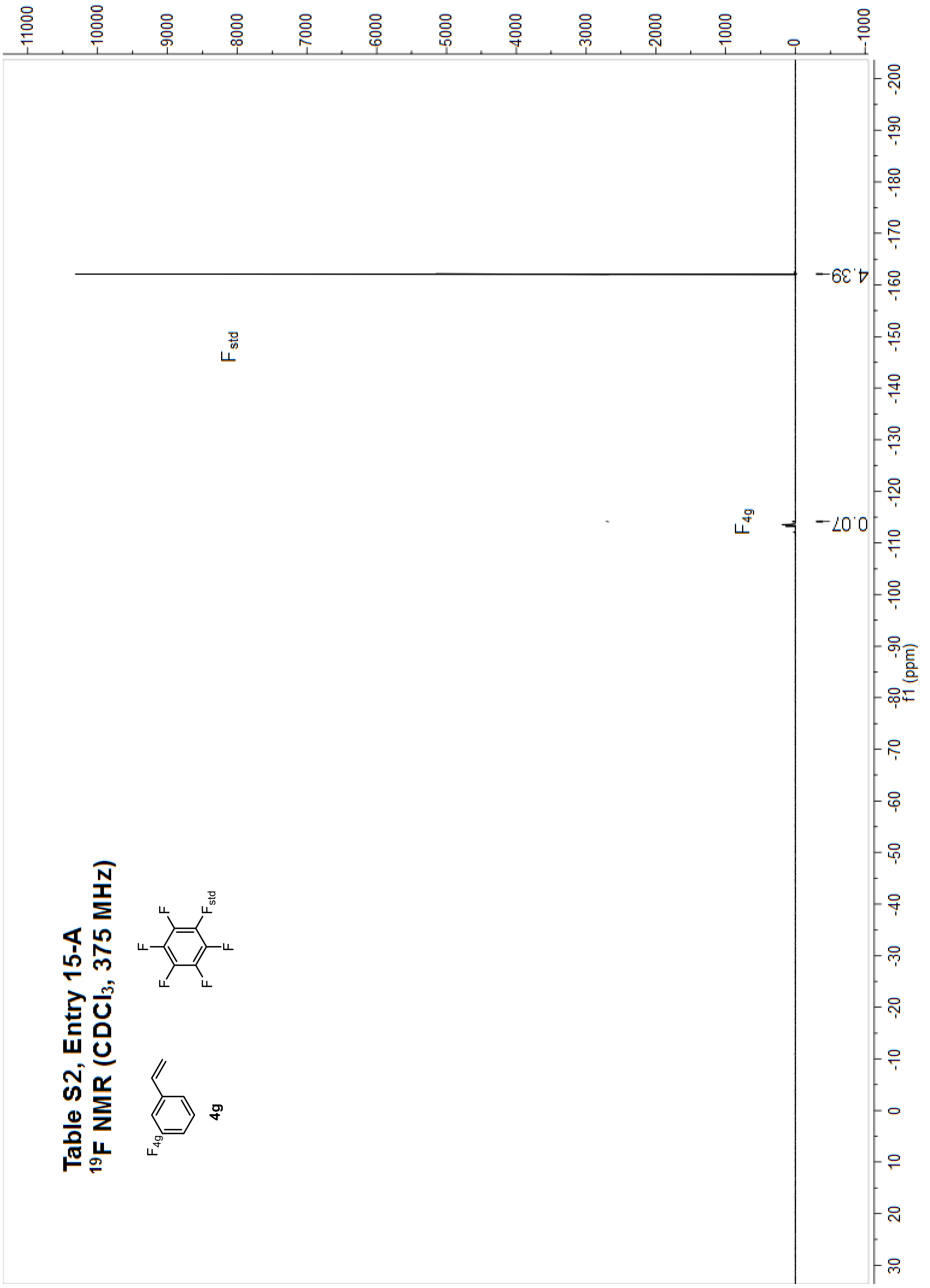
Table S2, Entry 15-A
19F NMR (CDCl3, 375 MHz)
F4g
4g
Fstd
4.39
0.07
f1 (ppm)

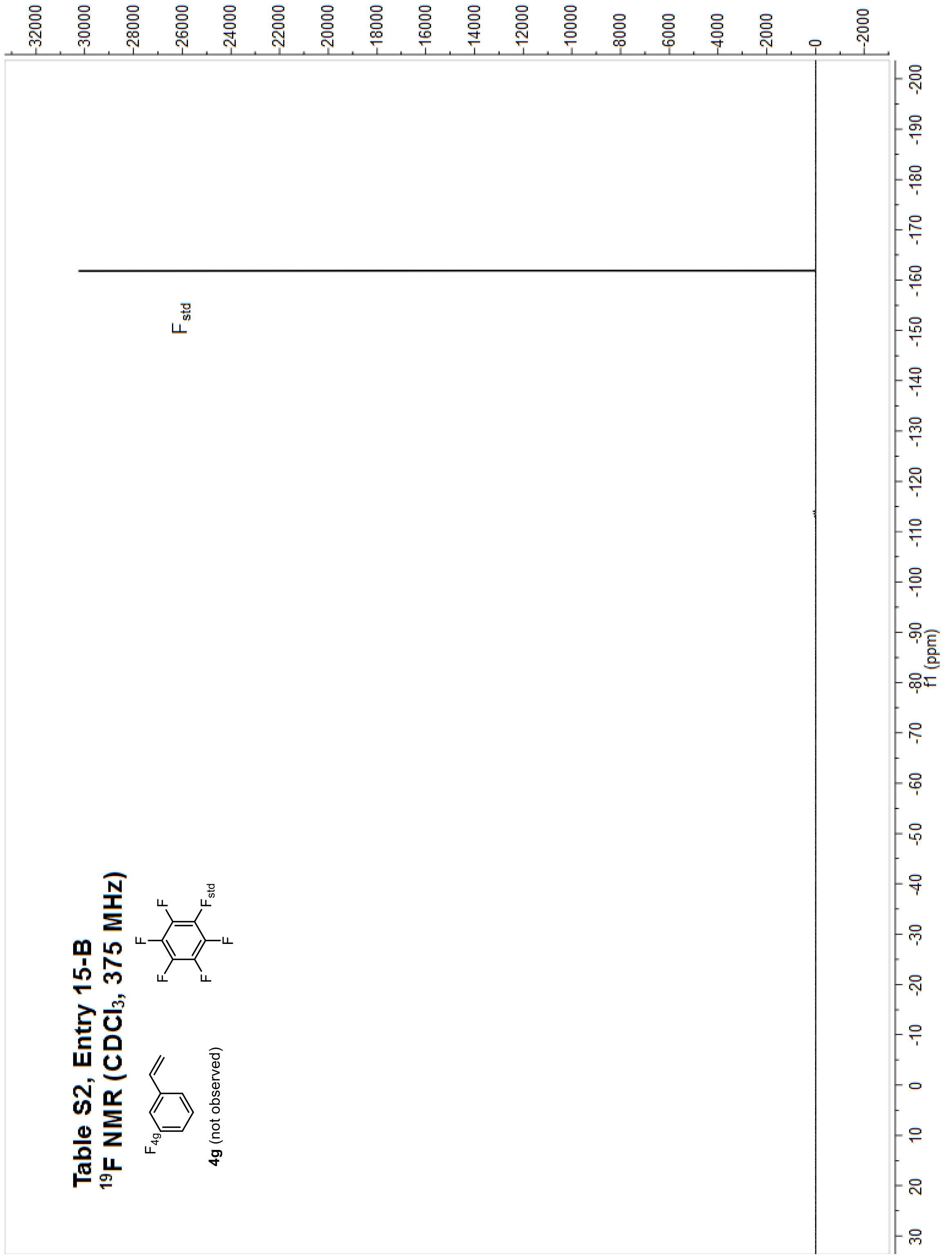
Table S2, Entry 15-B
19F NMR (CDCl3, 375 MHz)
F4g
4g (not observed)
Fstd
f1 (ppm)

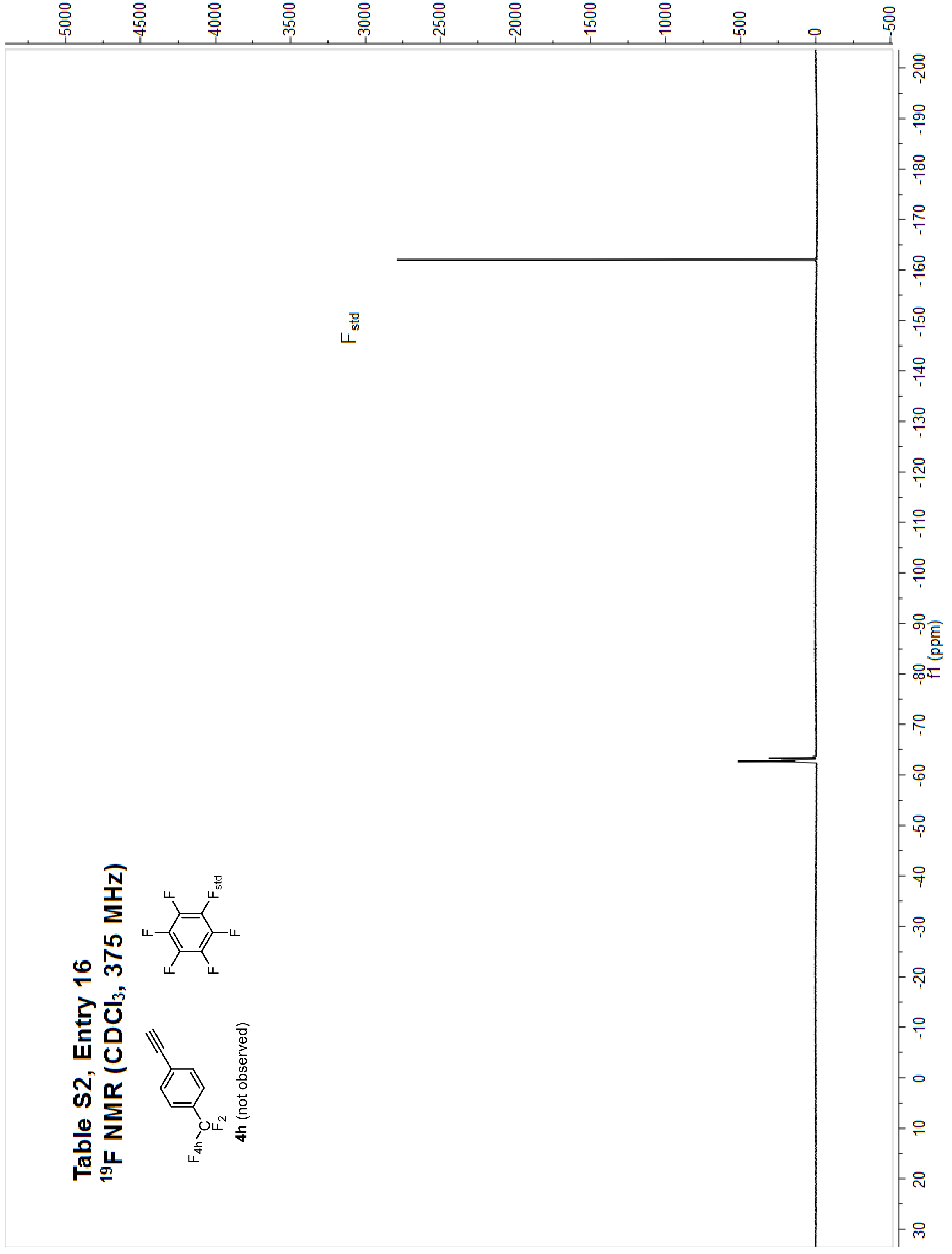
Table S2, Entry 16
^{19}F NMR ($CDCl_3$, 375 MHz)
F_{4h}
F_2
4h (not observed)
F_{std}
F_{std}
f1 (ppm)

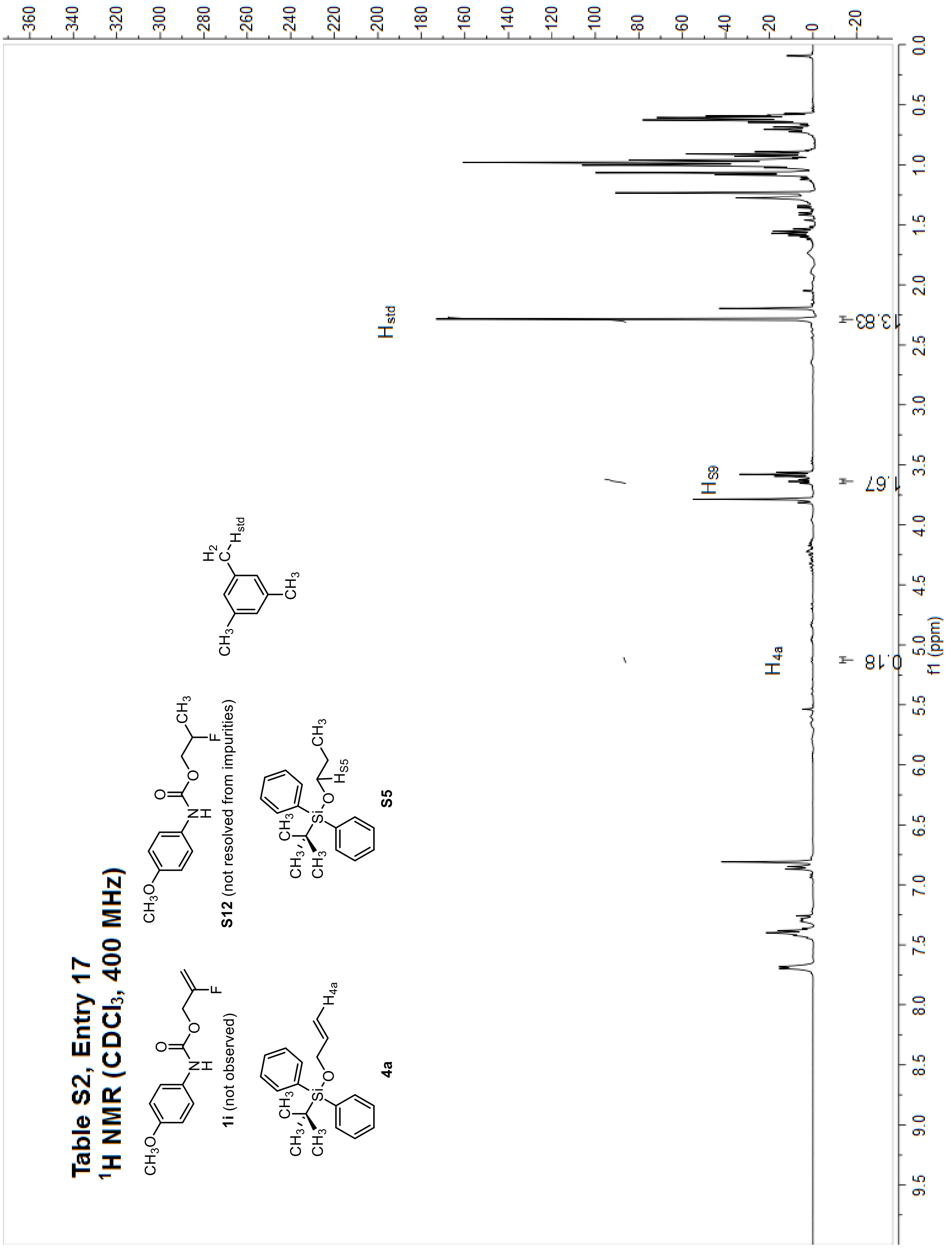

Table S2, Entry 17
^{1}H NMR ($CDCl_3$, 400 MHz)

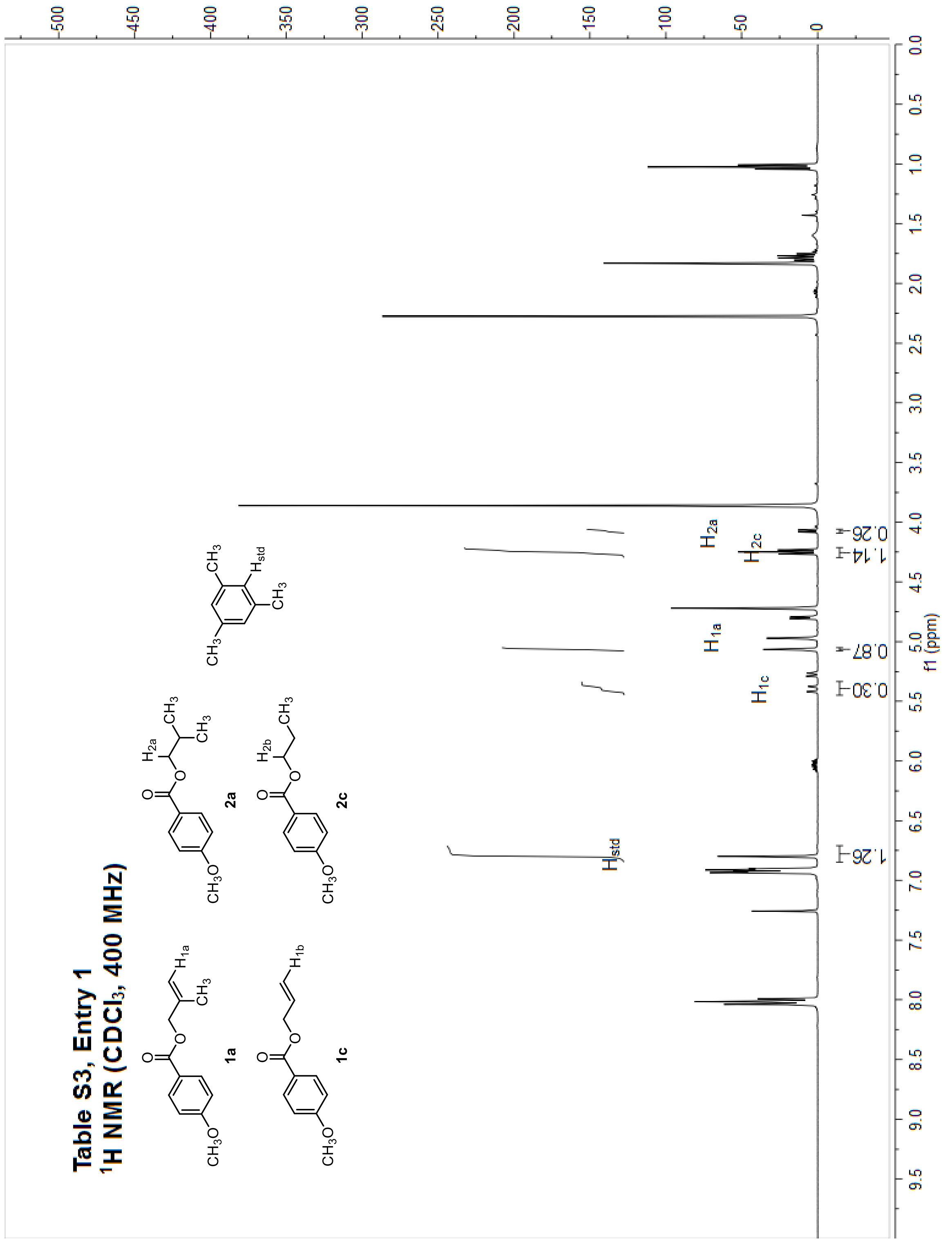
Table S3, Entry 1
1H NMR (CDCl3, 400 MHz)
1a
1c
2a
2c
H1a
H1b
H2a
H2b
H2c
H1c
Hstd
0.26
1.14
0.87
0.30
1.26
f1 (ppm)

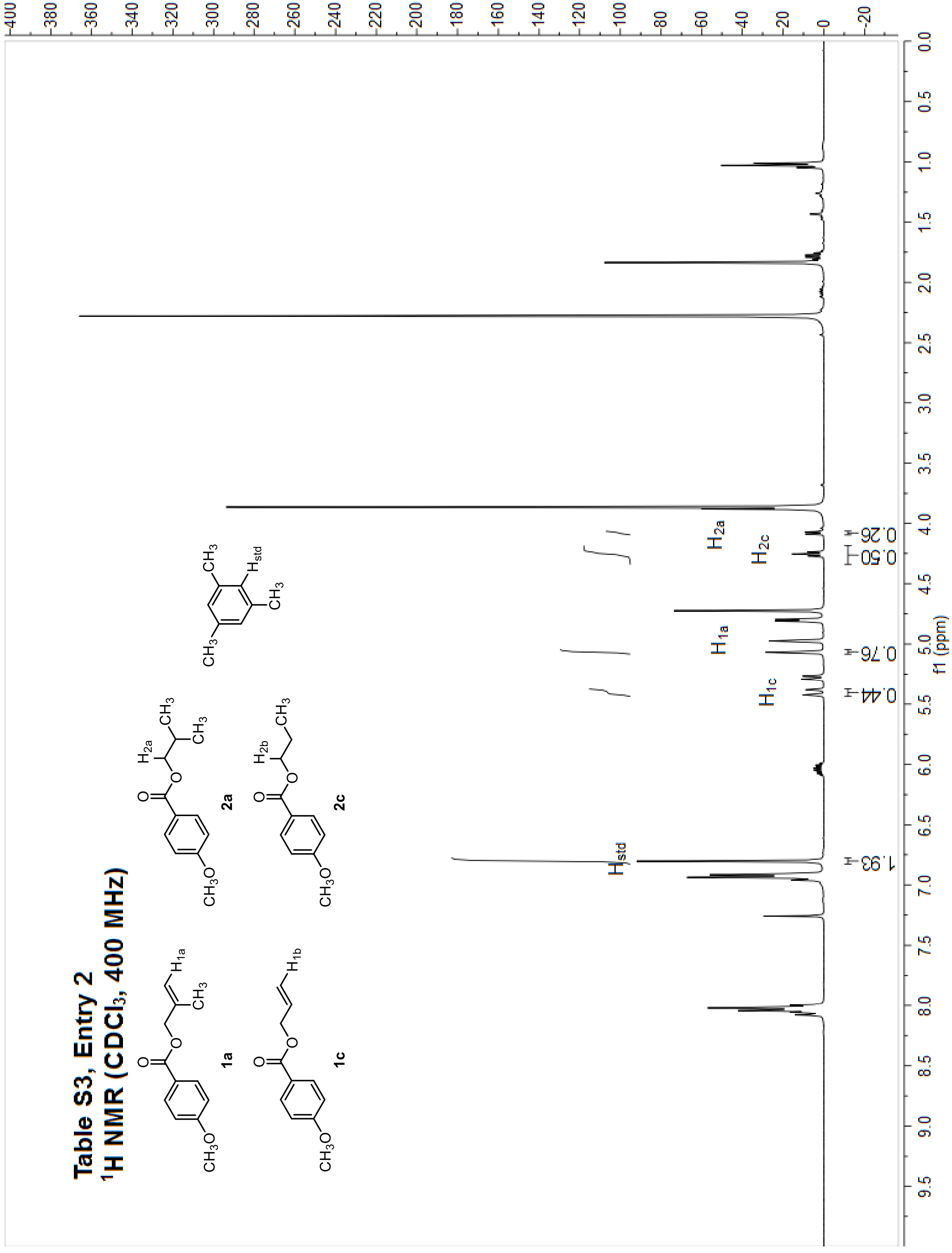

Table S3, Entry 2
^{1}H NMR ($CDCl_3$, 400 MHz)
1a
1c
2a
2c
H2a
H2c
H1a
H1c
Hstd
0.26
0.50
0.76
0.44
1.98
f1 (ppm)

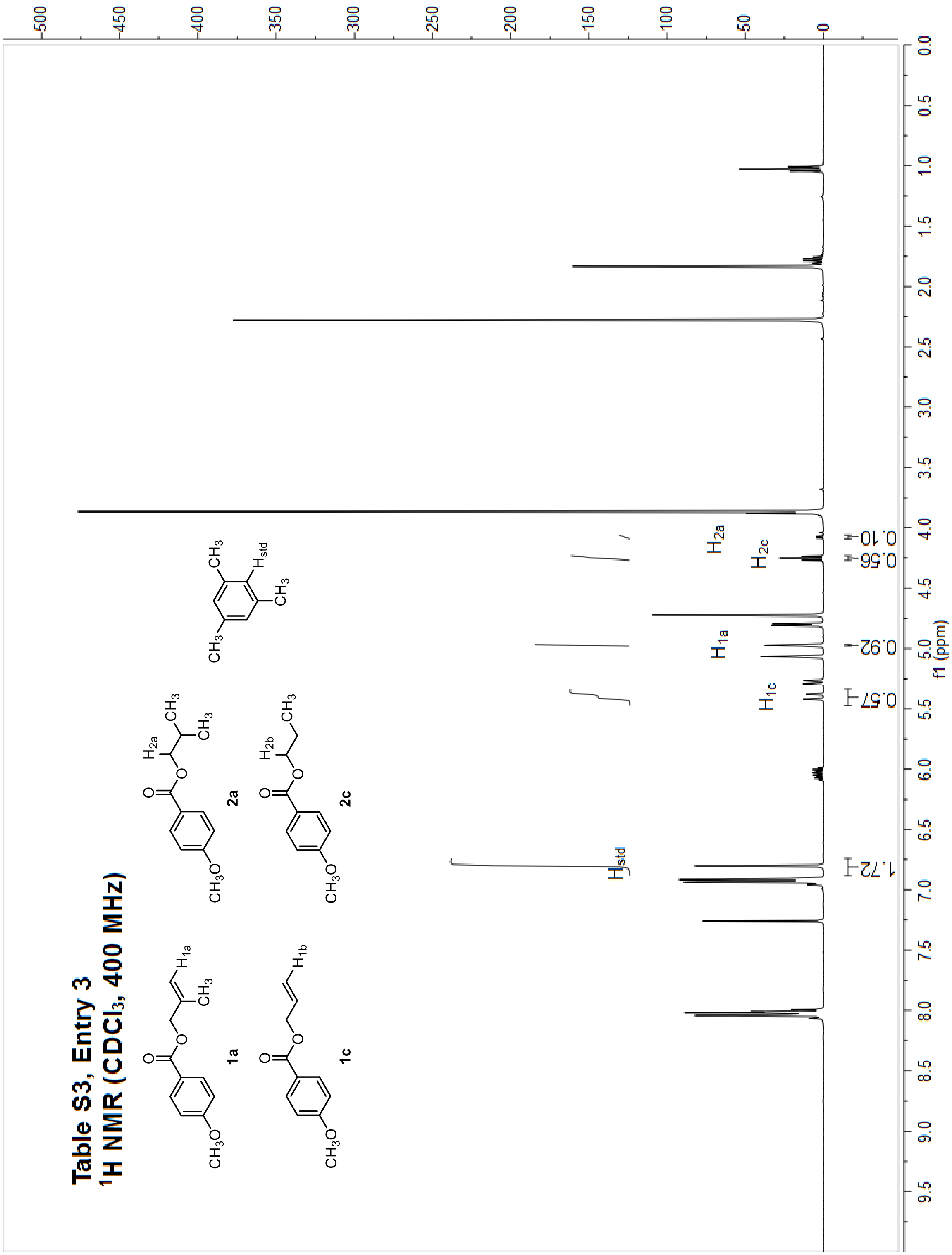

Table S3, Entry 3
1H NMR (CDCl3, 400 MHz)
1a
1c
2a
2c
H2a
H2c
H1a
H1c
Hstd
0.10
0.56
0.92
0.57
1.72
f1 (ppm)

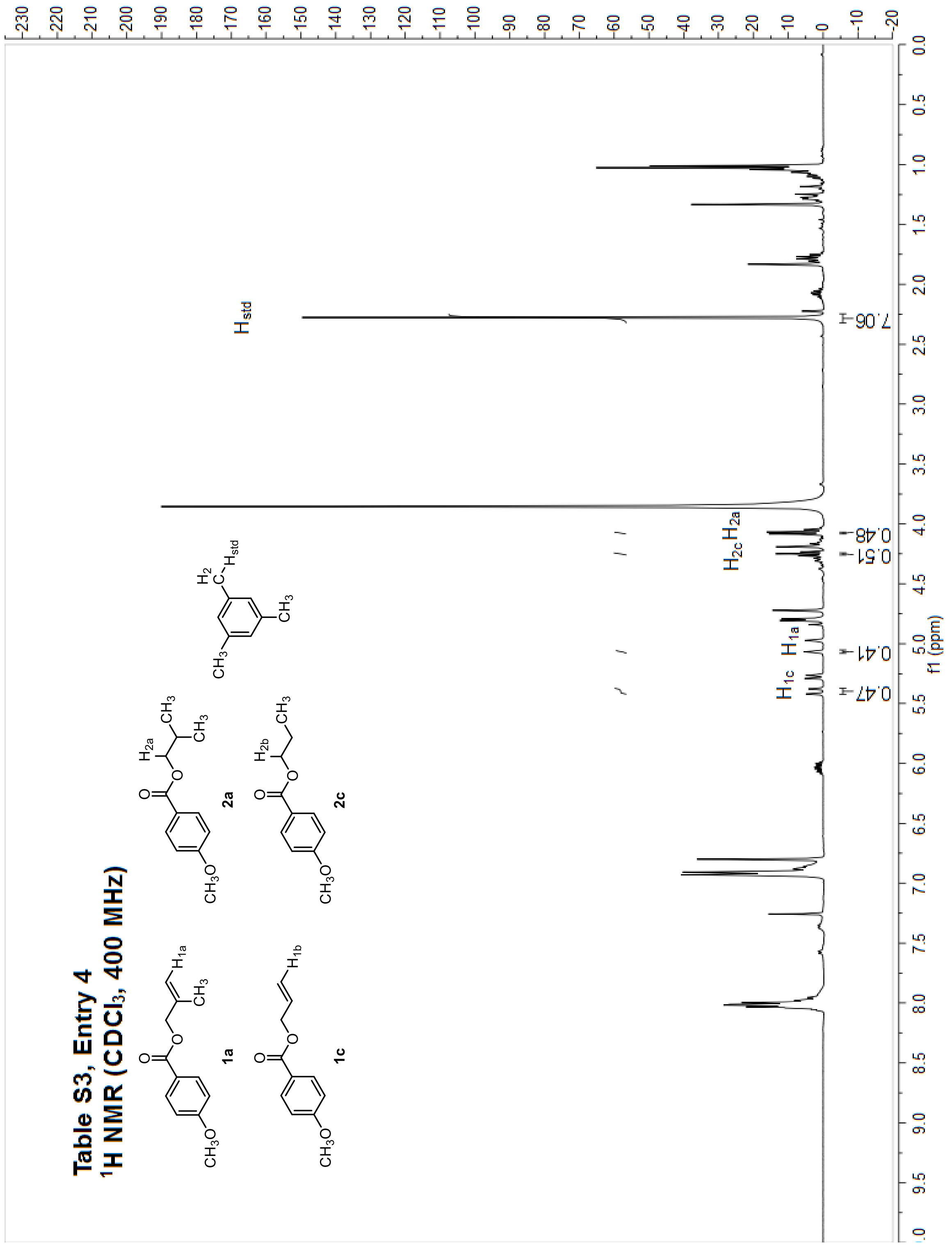

Table S3, Entry 4
1H NMR (CDCl3, 400 MHz)
1a
1c
2a
2c
Hstd
f1 (ppm)

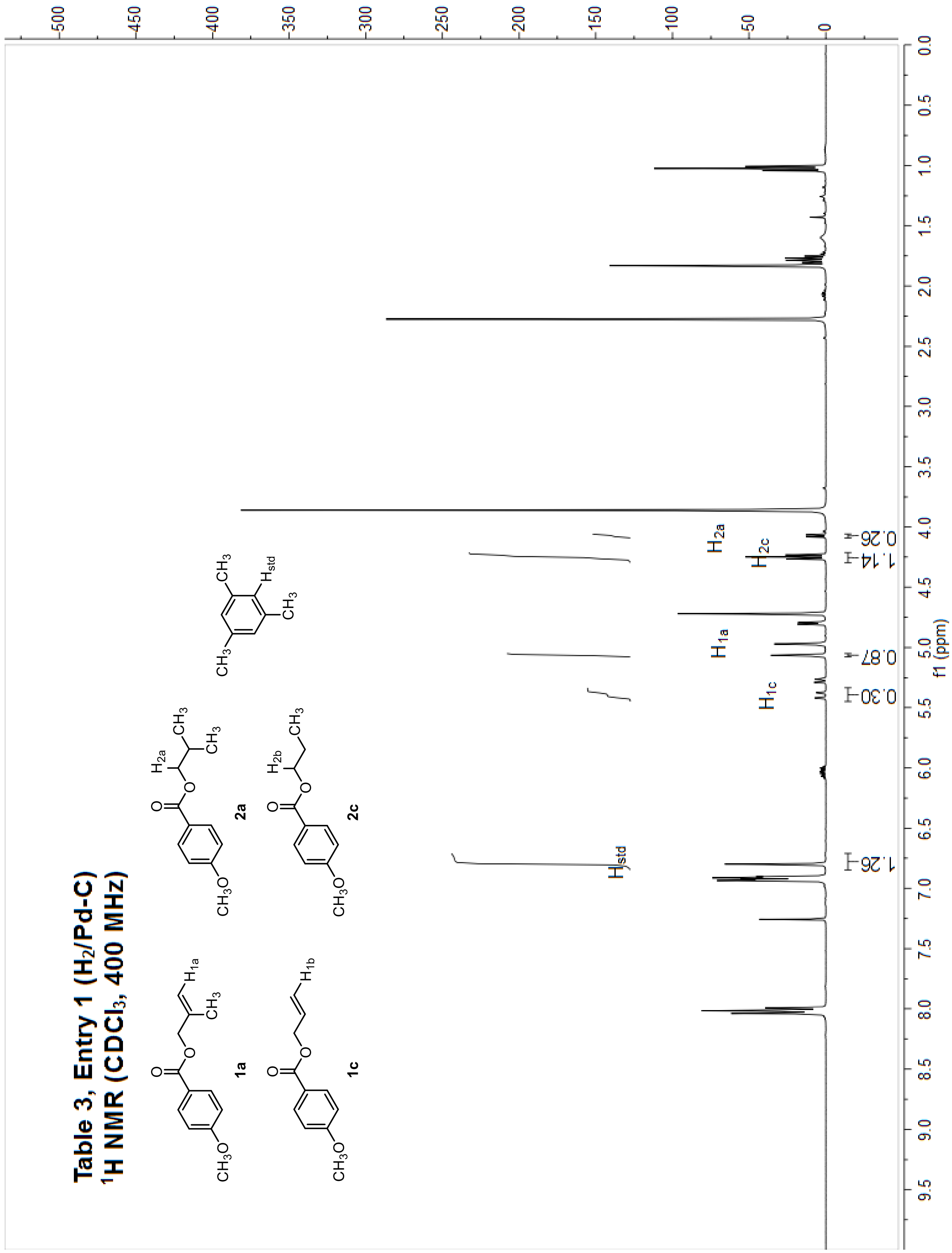
Table 3, Entry 1 (H_2/Pd-C)
^{1}H NMR ($CDCl_3$, 400 MHz)
1a
1c
2a
2c
H_{1a}
H_{1b}
H_{1c}
H_{2a}
H_{2b}
H_{2c}
H_{std}
CH_3O
CH_3
0.26
1.14
0.87
0.30
1.26
f1 (ppm)

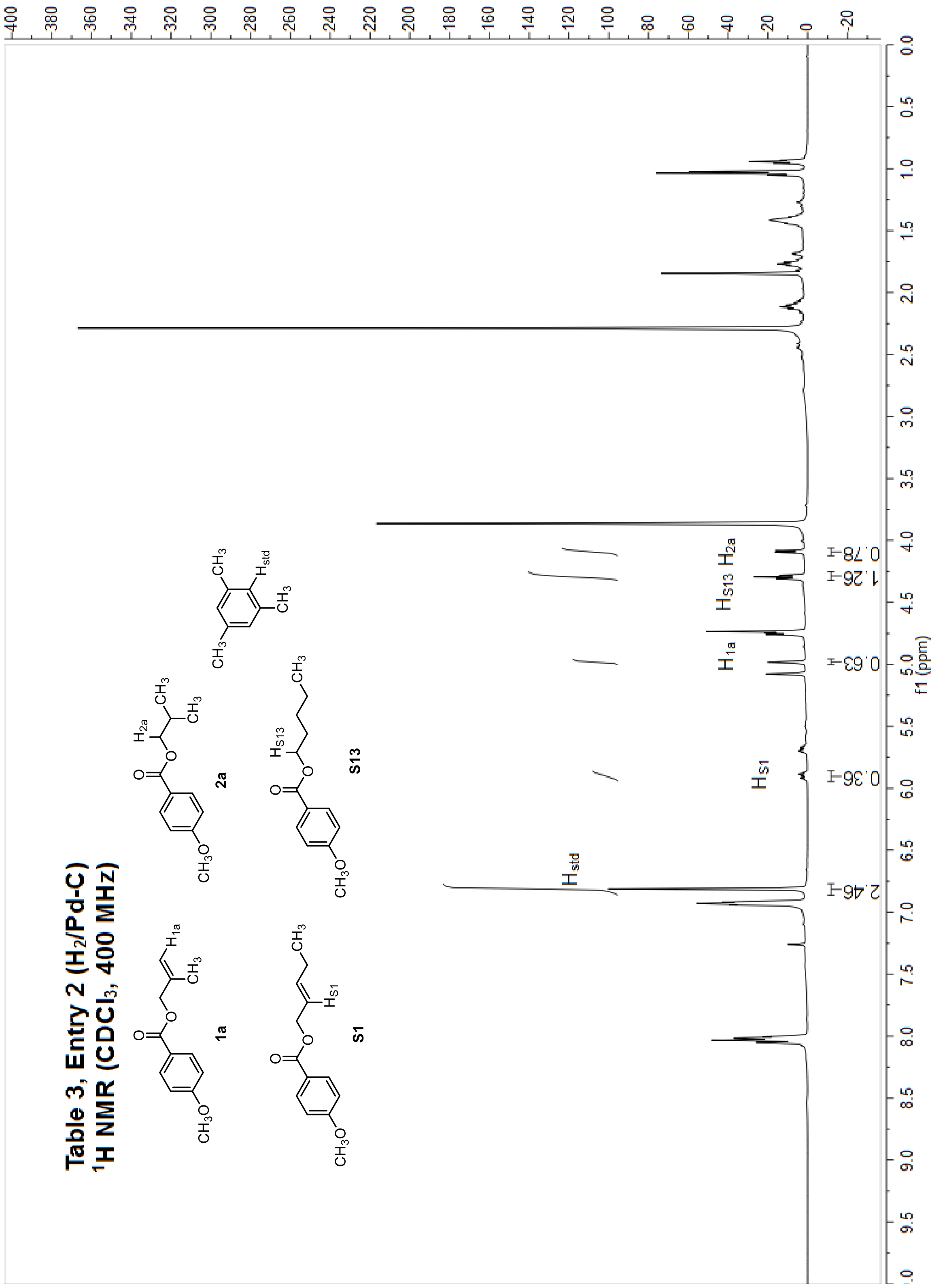
Table 3, Entry 2 (H_2/Pd-C)
^{1}H NMR ($CDCl_3$, 400 MHz)
1a
2a
S1
S13
H_{1a}
H_{2a}
H_{S1}
H_{S13}
H_{std}
0.78
1.26
0.63
0.36
2.46
f1 (ppm)

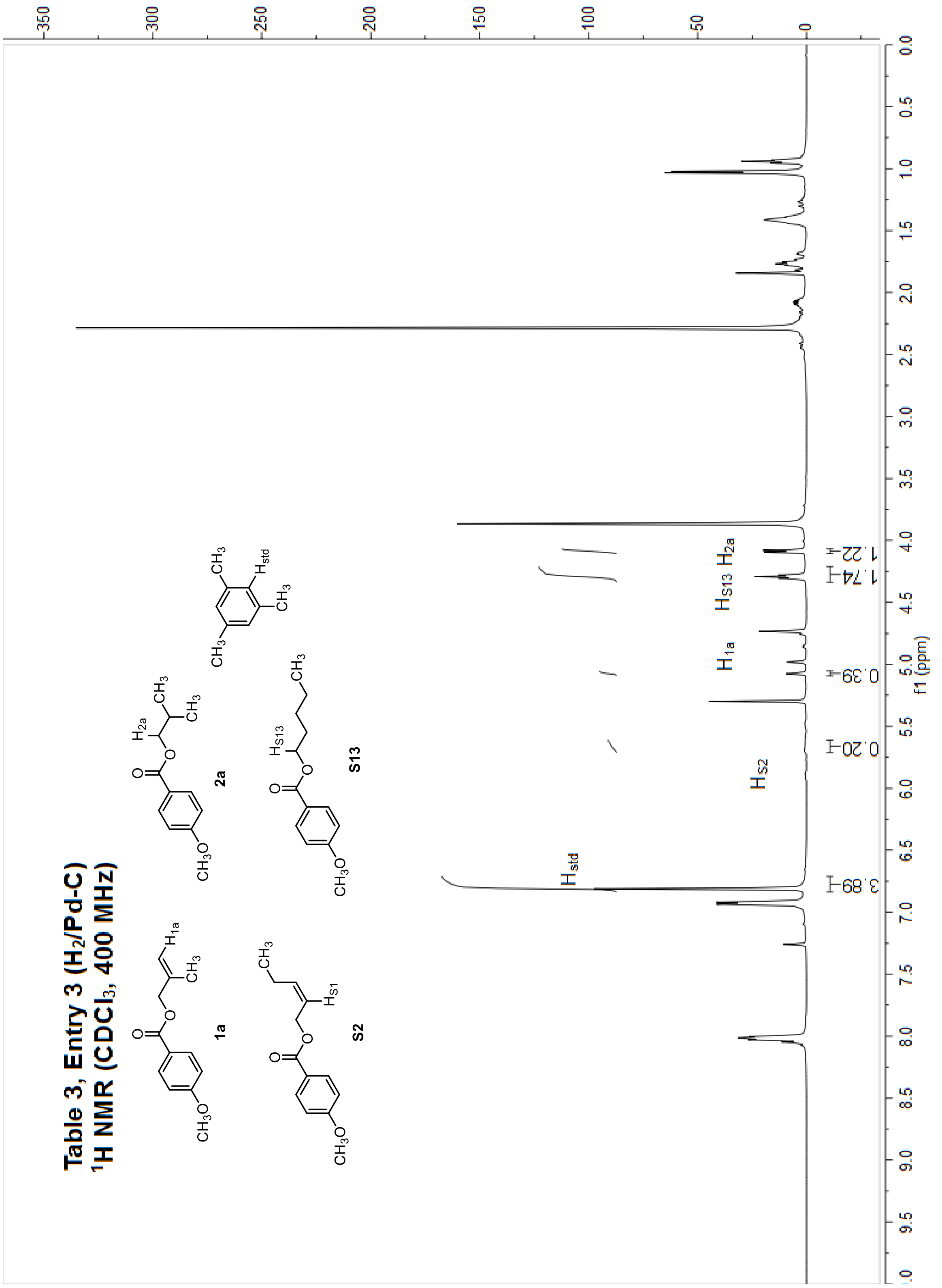
Table 3, Entry 3 (H_2/Pd-C)
^1H NMR ($CDCl_3$, 400 MHz)
1a
2a
S2
S13
H_{std}
H_{1a}
H_{S13} H_{2a}
H_{S2}
f1 (ppm)

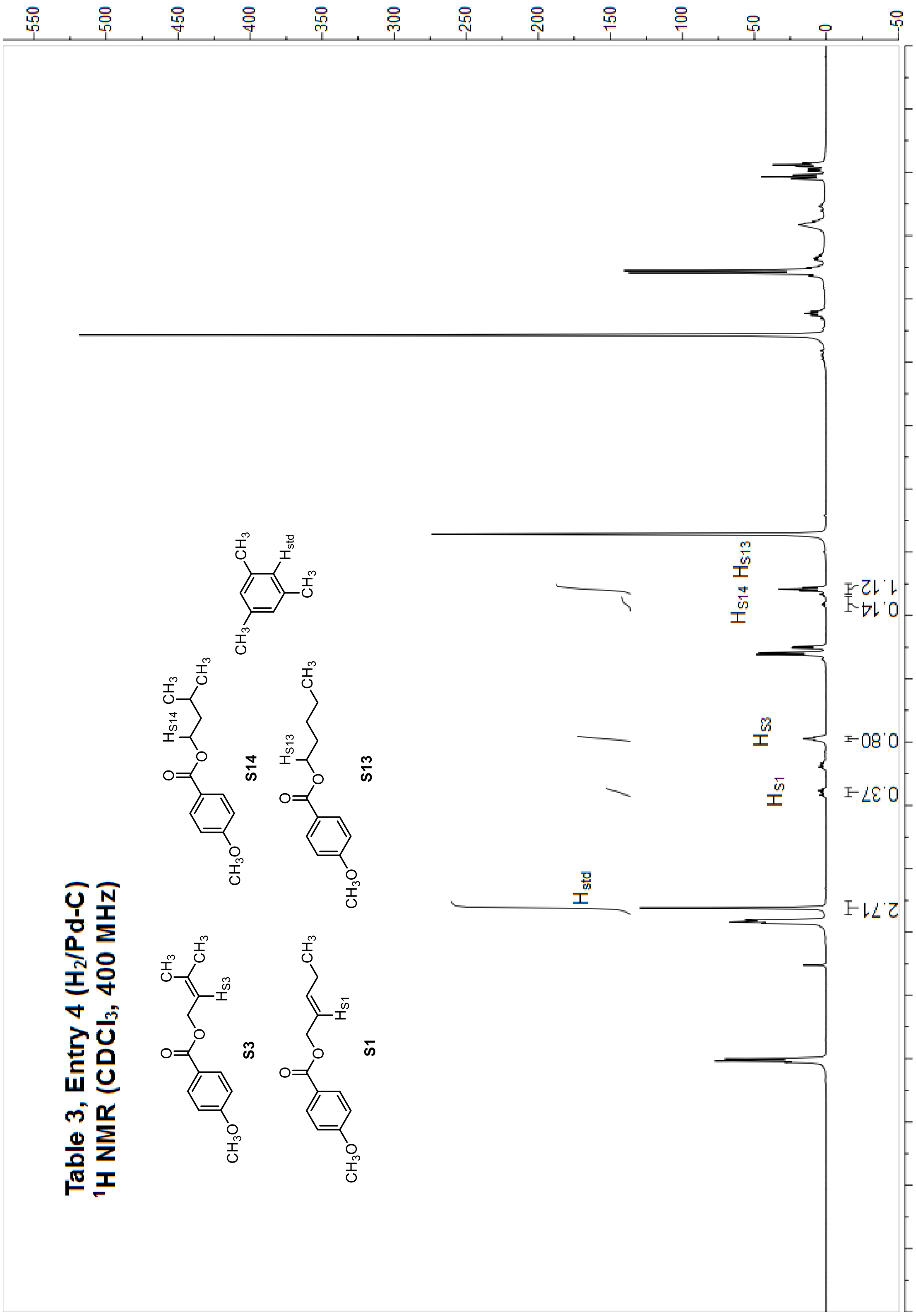
Table 3, Entry 4 (H2/Pd-C)
1H NMR (CDCl3, 400 MHz)
S3
S1
S14
S13
Hstd
HS14 HS13
HS3
HS1
2.71
0.37
0.80
0.14
1.12
f1 (ppm)

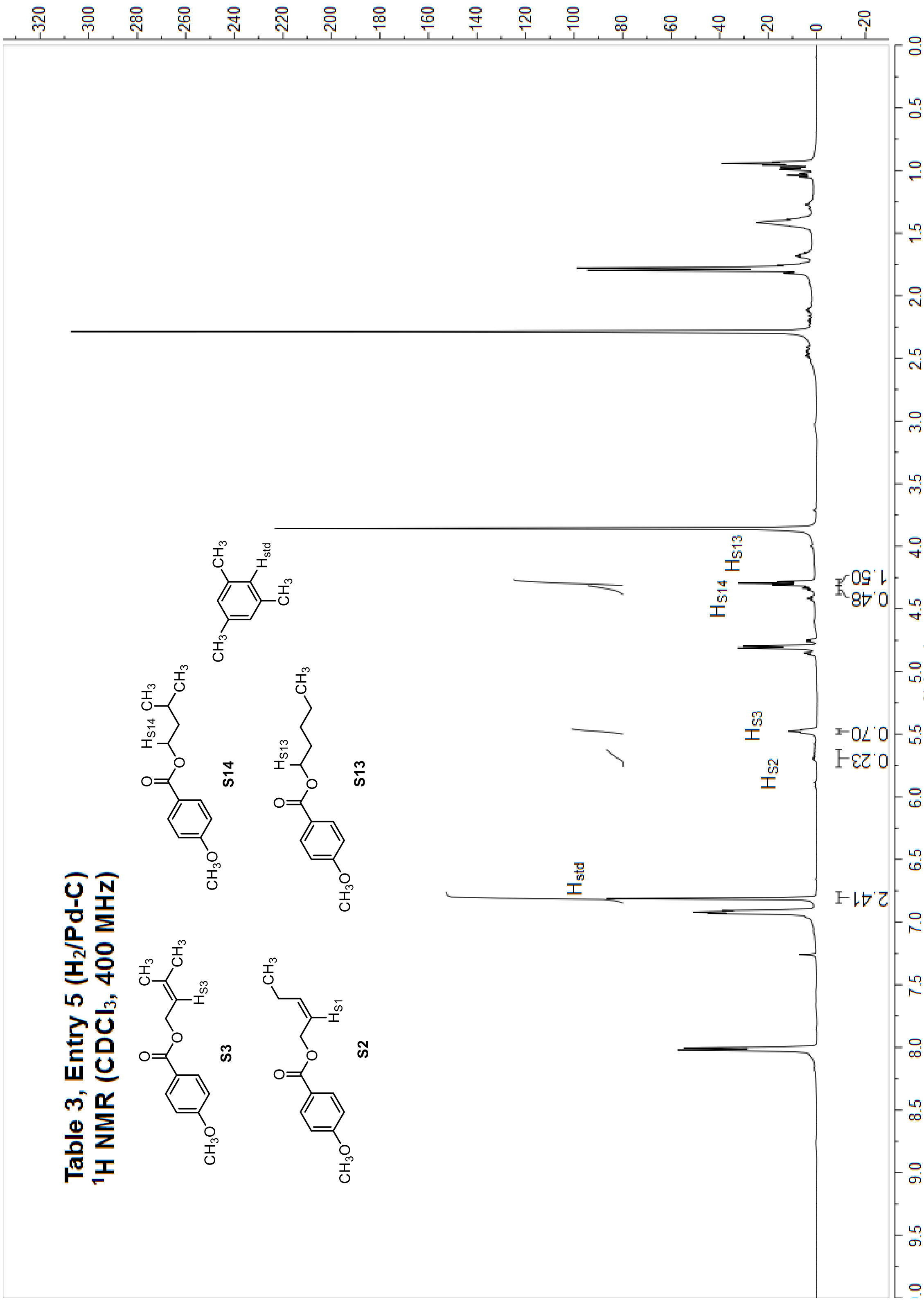

Table 3, Entry 5 (H_2/Pd-C)
^{1}H NMR ($CDCl_3$, 400 MHz)
S3
S2
S14
S13
H_{S3}
H_{S1}
H_{S14}
H_{S13}
H_{std}
H_{S2}
2.41
0.23
0.70
0.48
1.50
f1 (ppm)

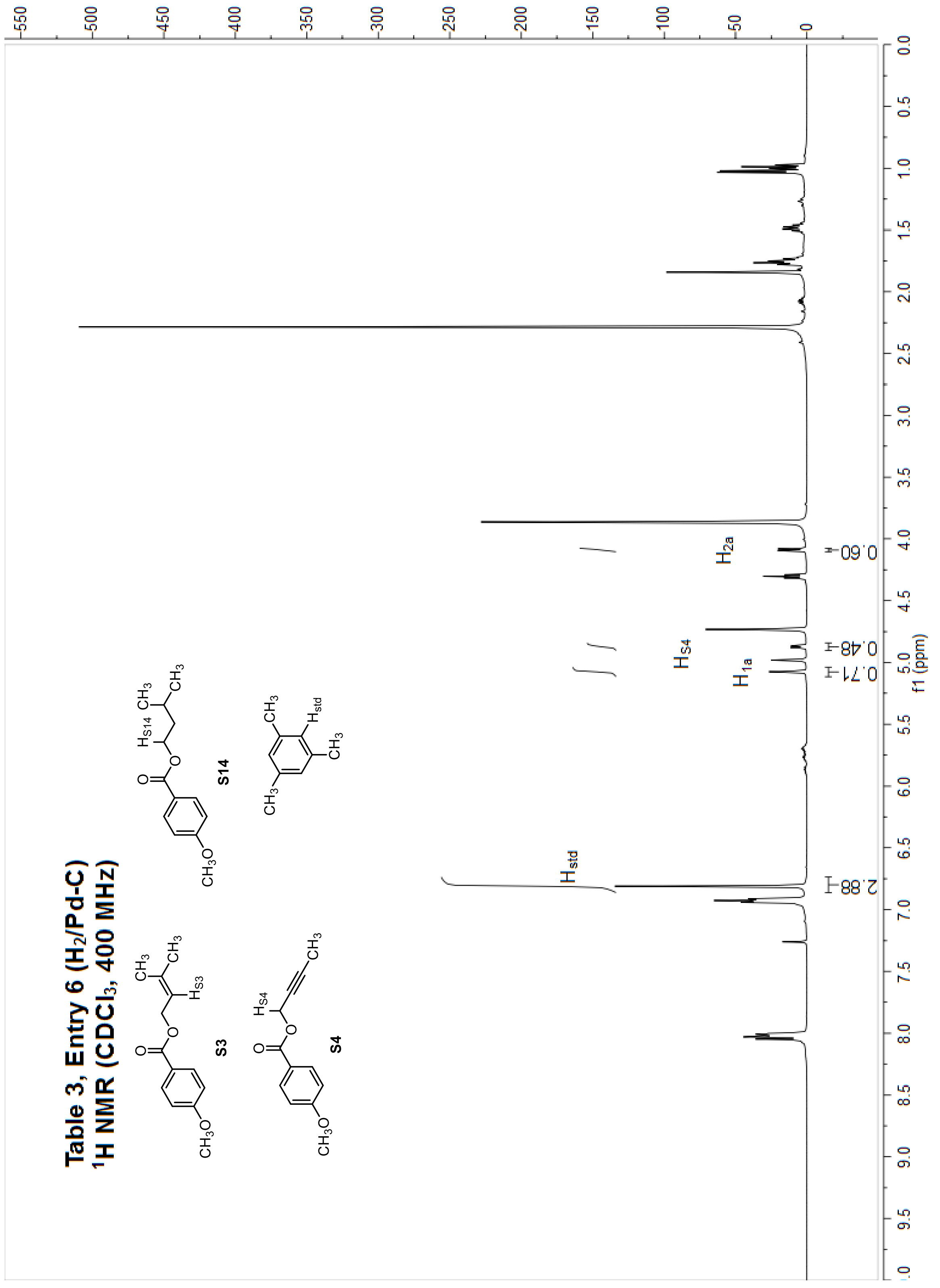
Table 3, Entry 6 (H2/Pd-C)
1H NMR (CDCl3, 400 MHz)
S3
S4
S14
Hstd
HS4
H1a
H2a
f1 (ppm)

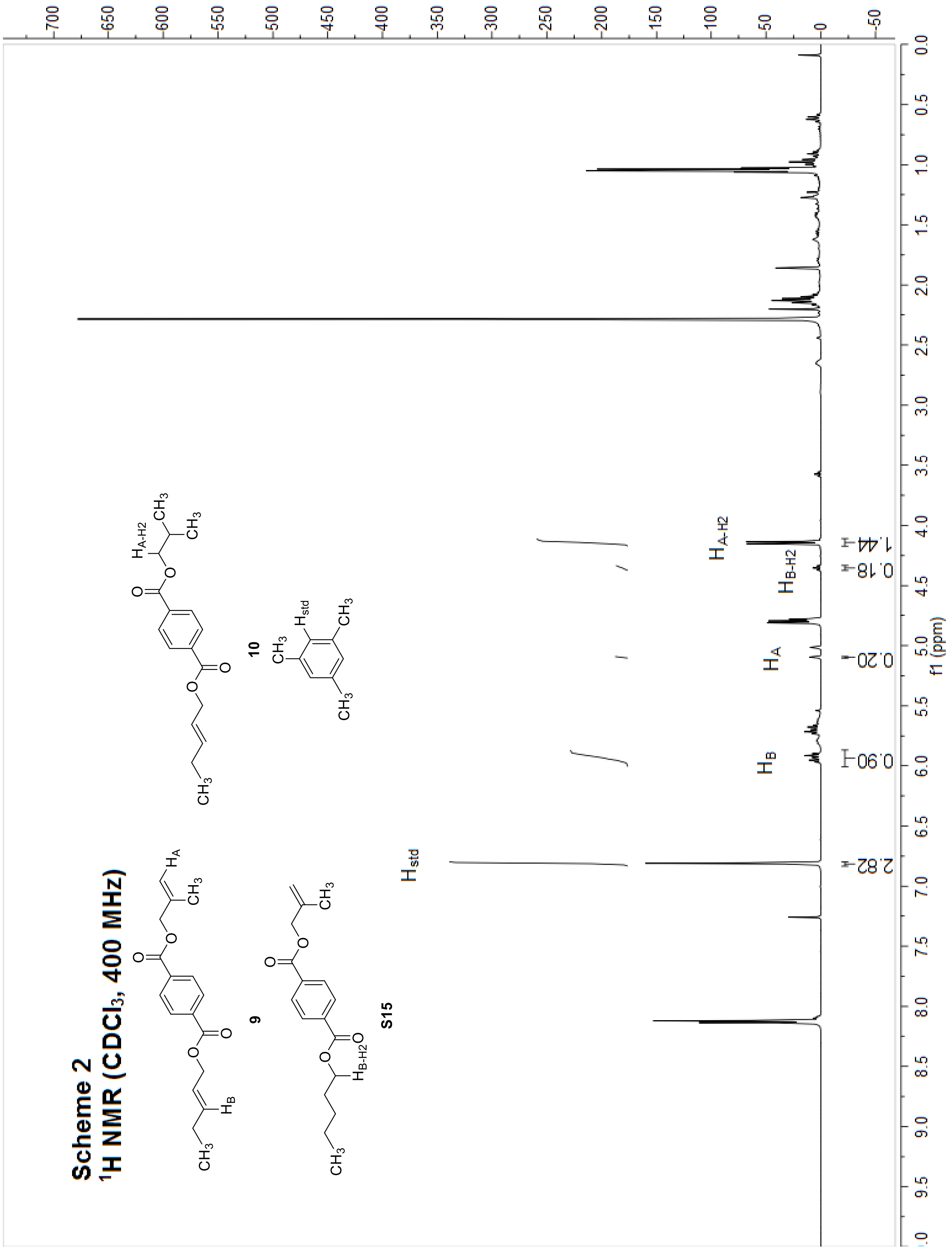
Scheme 2
1H NMR (CDCl3, 400 MHz)
9
S15
10
HA
HB
HB-H2
HA-H2
Hstd
2.82
0.90
0.20
0.18
1.44
f1 (ppm)

Bibliography:

[1] A. B. Pangborn, M. A. Giardello, R. H. Grubbs, R. K. Rosen, F. J. Timmers, *Organometallics* **1996**, *15*, 1518-1520.

[2] S. B. Daval, C. Valant, D. Bonnet, E. Kellenberger, M. Hibert, J.-L. Galzi, B. Ilien, *J. Med. Chem.* **2012**, *55*, 2125-2143.

[3] R. K. Boeckman, J. E. Pero, D. J. Boehmler, *J. Am. Chem. Soc.* **2006**, *128*, 11032-11033.

[4] M. J. O'Mahony, R. A. More O'Ferrall, D. R. Boyd, C. M. Lam, A. C. O'Donoghue, *J. Phys. Org. Chem.* **2013**, *26*, 989-996.

[5] K. Iwasaki, K. K. Wan, A. Oppedisano, S. W. M. Crossley, R. A. Shenvi, *J. Am. Chem. Soc.* **2014**, *136*, 1300-1303.

[6] A. C. Poshkus, J. E. Herweh, F. A. Magnotta, *J. Org. Chem.* **1963**, *28*, 2766-2769.

[7] G. L. Edwards, C. A. Muldoon, D. J. Sinclair, *Tetrahedron* **1996**, *52*, 7779-7788.

[8] D.-W. Chen, Z.-C. Chen, *Tetrahedron Lett.* **1994**, *35*, 7637-7638.

[9] B. Gaspar, E. M. Carreira, *J. Am. Chem. Soc.* **2009**, *131*, 13214-13215.

[10] W. G. Kofron, L. M. Baclawski, *J. Org. Chem.* **1976**, *41*, 1879-1880.

[11] E. J. Corey, A. Guzman-Perez, M. C. Noe, *J. Am. Chem. Soc.* **1995**, *117*, 10805-10816.

[12] R. Chênevert, M. Dasser, *J. Org. Chem.* **2000**, *65*, 4529-4531.

[13] T. Debnar, T. Wang, D. Menche, *Org. Lett.* **2013**, *15*, 2774-2777.

[14] S. M. King, X. Ma, S. B. Herzon, *J. Am. Chem. Soc.* **2014**, *136*, 6884-6887.

[15] L. O. Ofori, T. A. Hilimire, R. P. Bennett, N. W. Brown, H. C. Smith, B. L. Miller, *J. Med. Chem.* **2014**, *57*, 723-732.

[16] J. Waser, H. Nambu, E. M. Carreira, *J. Am. Chem. Soc.* **2005**, *127*, 8294-8295.

[17] S.-r. Choi, M. Breugst, K. N. Houk, C. D. Poulter, *J. Org. Chem.* **2014**, *79*, 3572-3580.

[18] B. Lu, C. Li, L. Zhang, *J. Am. Chem. Soc.* **2010**, *132*, 14070-14072.

[19] J.-I. Lee, *Bull. Korean Chem. Soc.* **2011**, *32*, 1765.

[20] S. J. Aitken, G. Grogan, C. S.-Y. Chow, N. J. Turner, S. L. Flitsch, *J. Chem. Soc., Perkin Trans. 1* **1998**, 3365-3370.

[21] K. Iwasaki, K. K. Wan, A. Oppedisano, S. W. M. Crossley, R. A. Shenvi, *J. Am. Chem. Soc.* **2014**, *136*, 1300-1303.
